# Supplementary figures and images for: Layered patterns in nature, medicine, and materials: quantifying anisotropic structures and cyclicity (part 1 of 4)
Source: PeerJ. 2019 Oct 14;7:e7813. doi: 10.7717/peerj.7813 (PMC6797002; doi:10.7717/peerj.7813)

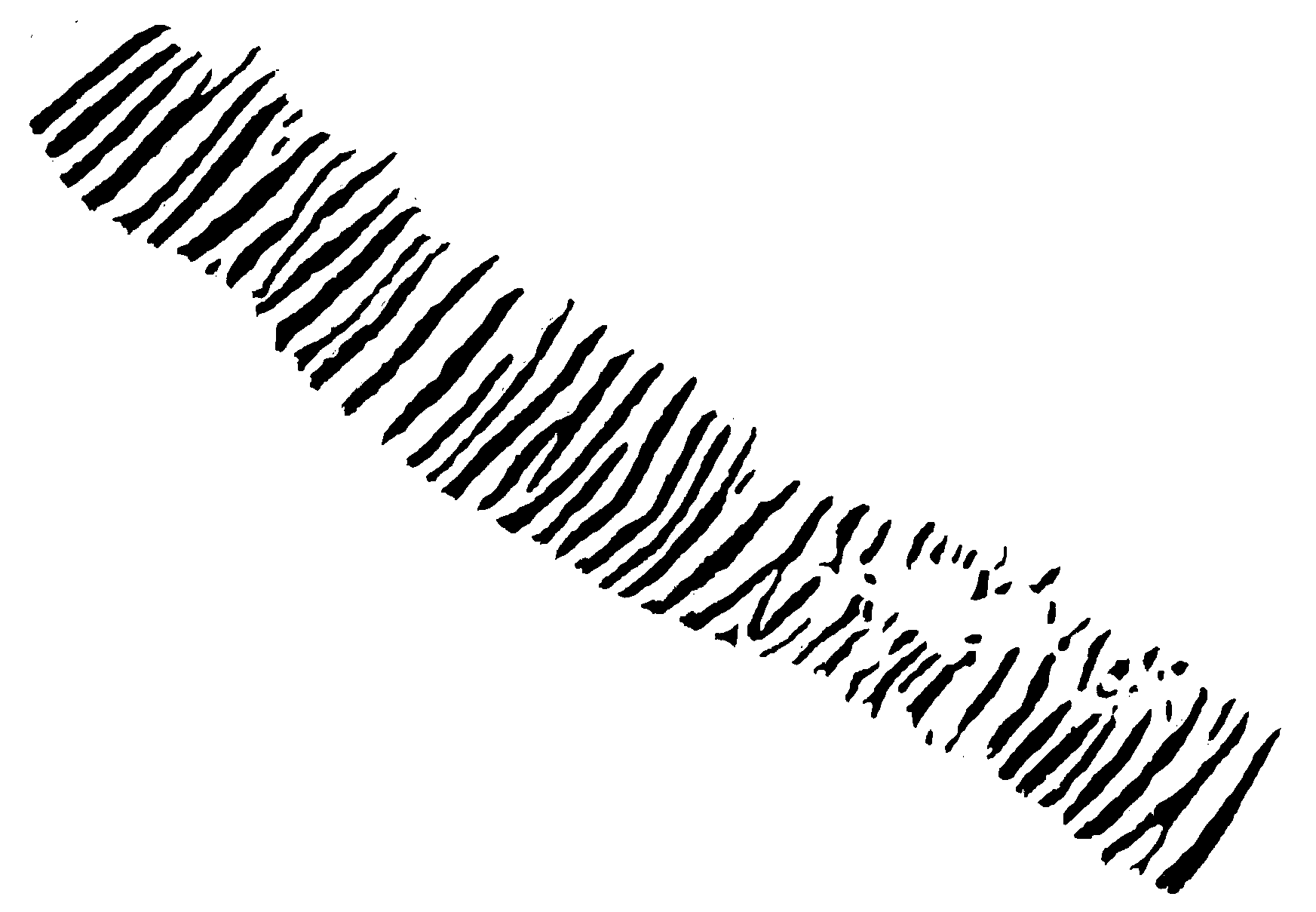

Supplement: Supplemental Information 2 [file peerj-07-7813-s002.zip › Supplemental2/Fig06A_MarsAnom.bmp]

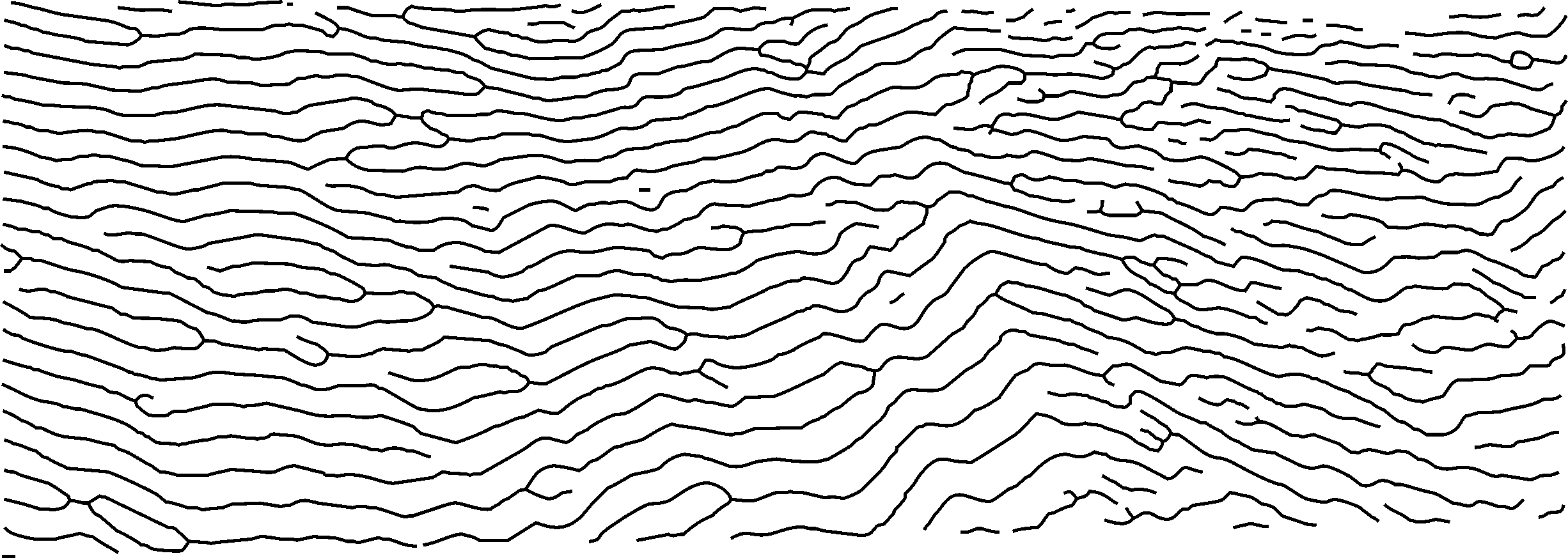

Supplement: Supplemental Information 2 [file peerj-07-7813-s002.zip › Supplemental2/Fig06B_Blue.bmp]

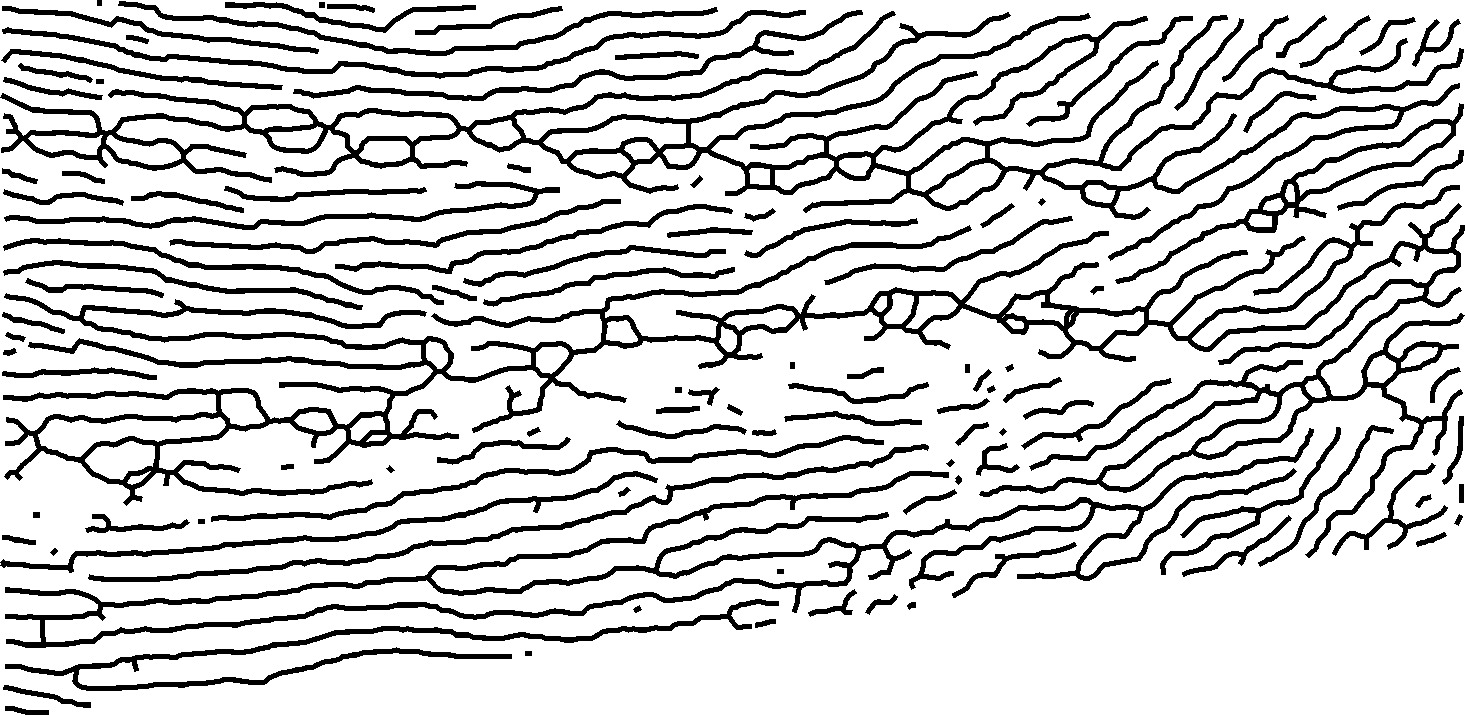

Supplement: Supplemental Information 2 [file peerj-07-7813-s002.zip › Supplemental2/Fig06B_Red.bmp]

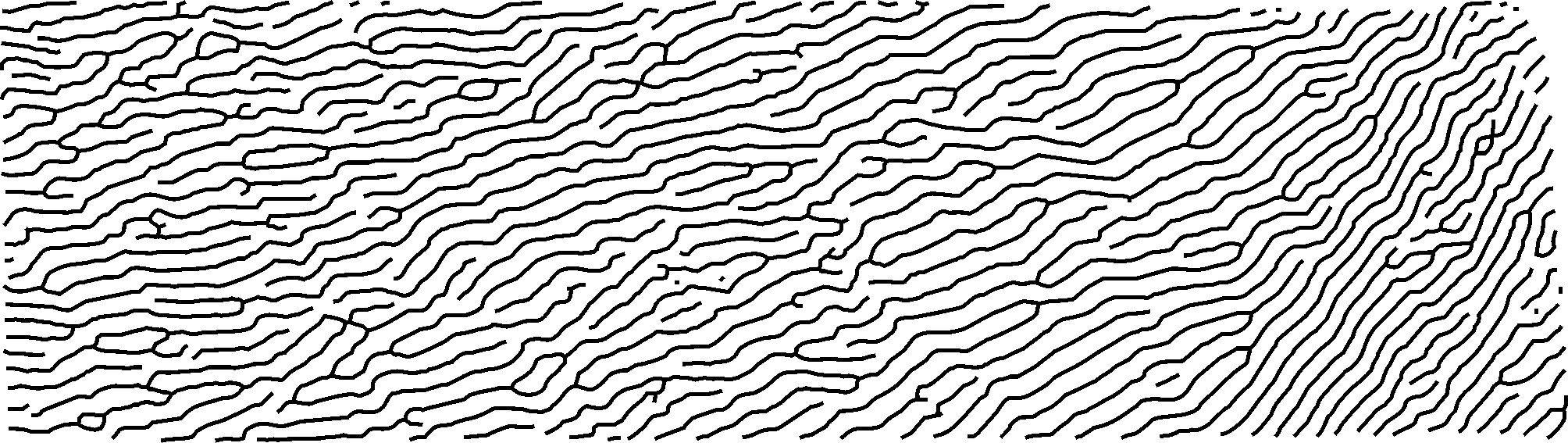

Supplement: Supplemental Information 2 [file peerj-07-7813-s002.zip › Supplemental2/Fig06B_Yellow.bmp]

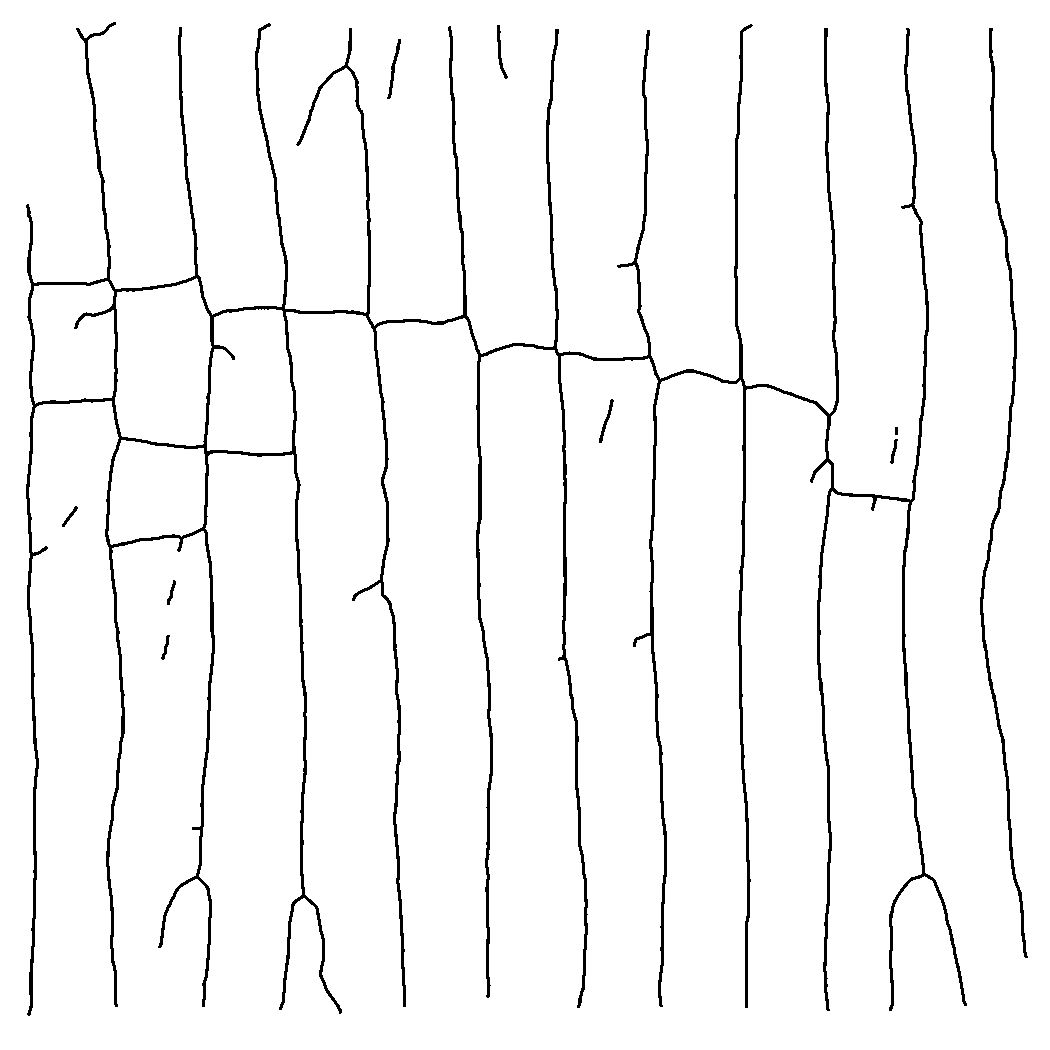

Supplement: Supplemental Information 2 [file peerj-07-7813-s002.zip › Supplemental2/Fig07-Blue.bmp]

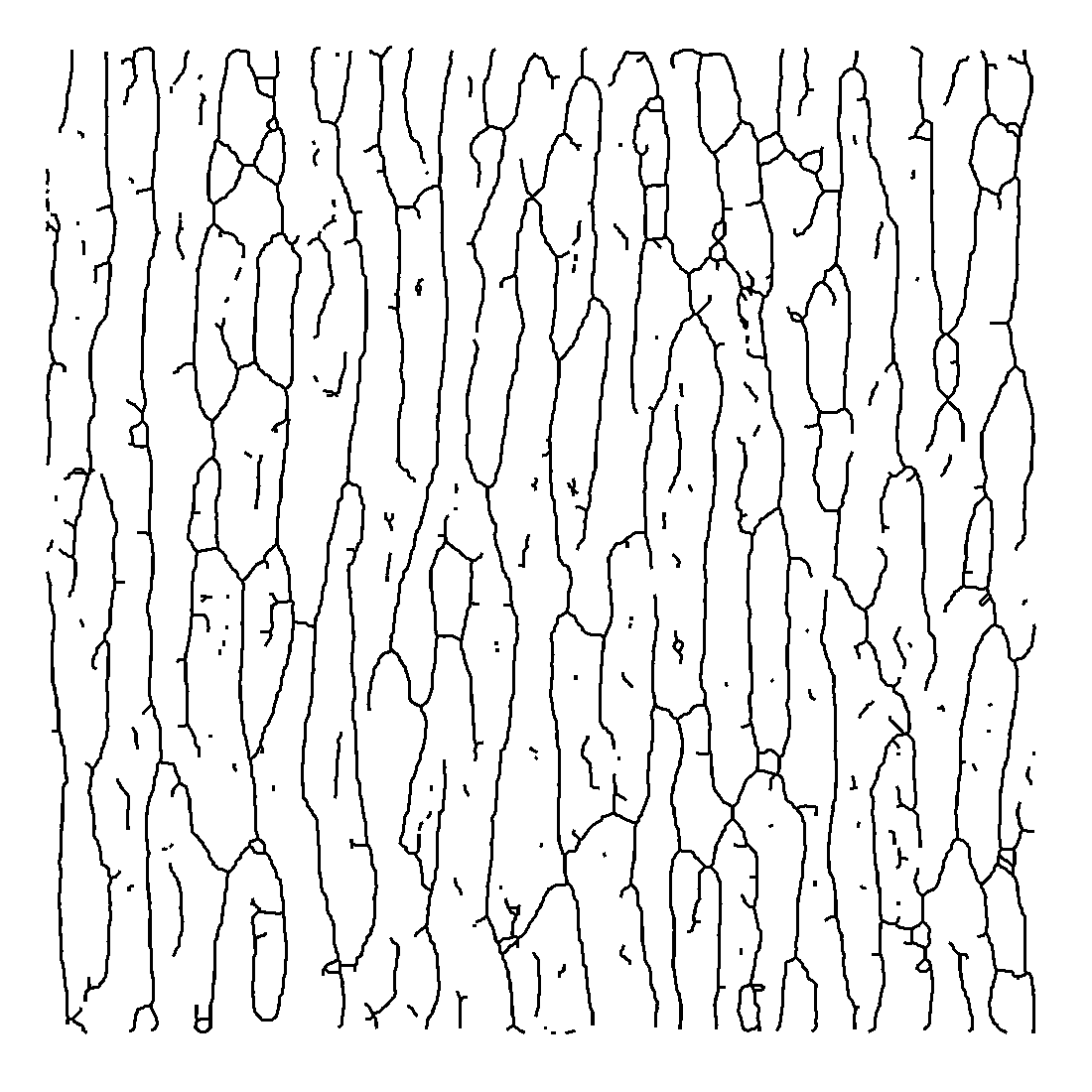

Supplement: Supplemental Information 2 [file peerj-07-7813-s002.zip › Supplemental2/Fig07-Green.bmp]

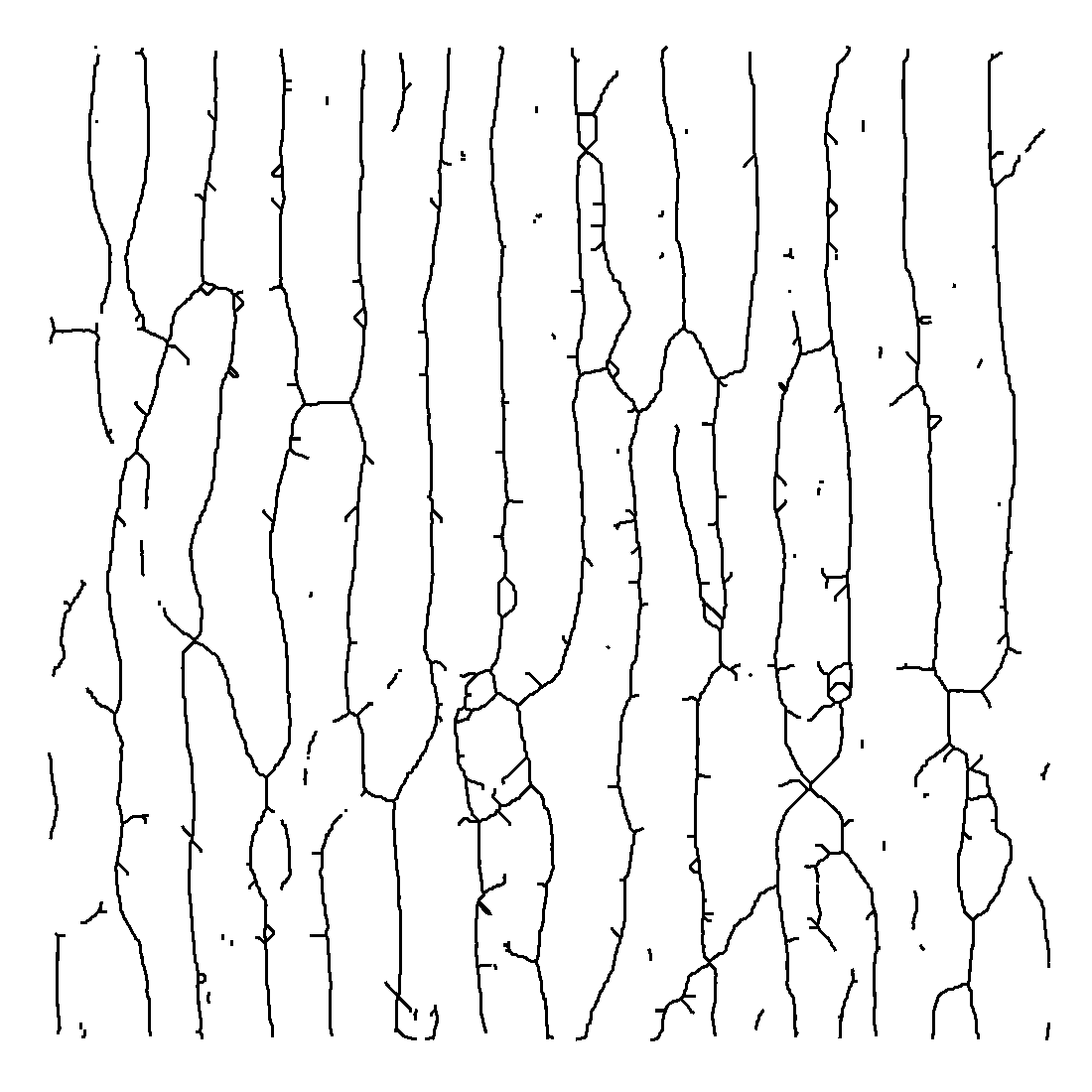

Supplement: Supplemental Information 2 [file peerj-07-7813-s002.zip › Supplemental2/Fig07-Red.bmp]

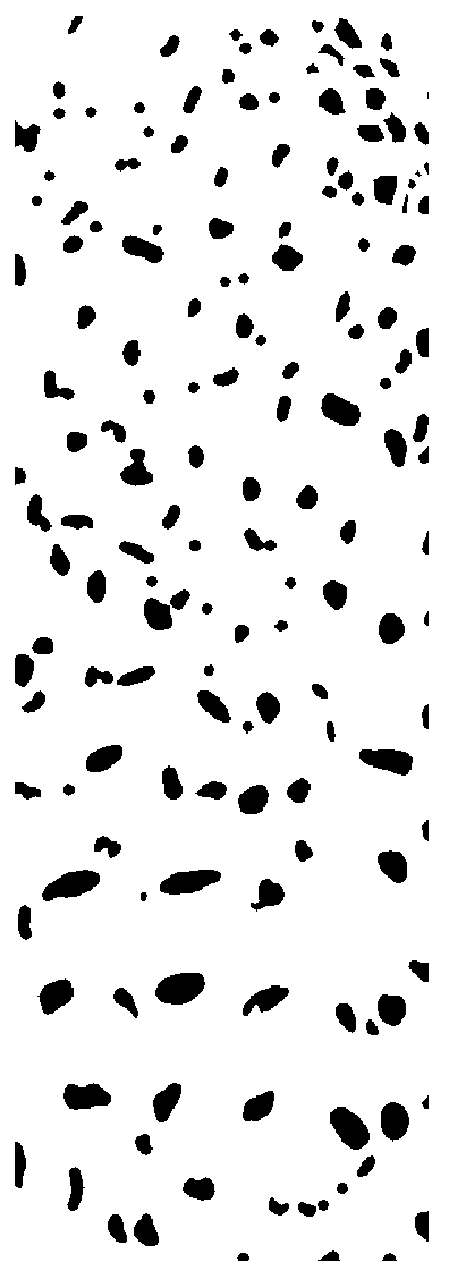

Supplement: Supplemental Information 2 [file peerj-07-7813-s002.zip › Supplemental2/Fig09A_GiantKingFisher.bmp]

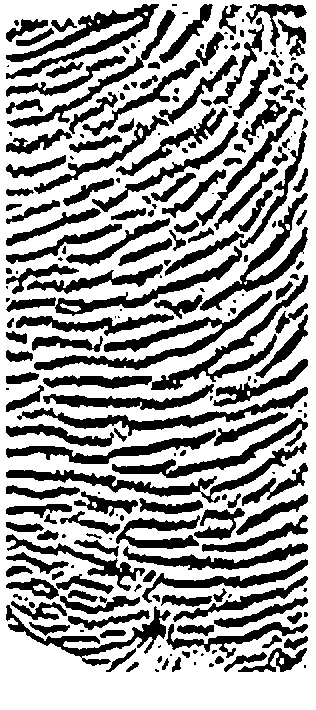

Supplement: Supplemental Information 2 [file peerj-07-7813-s002.zip › Supplemental2/Fig09B_Pitta.bmp]

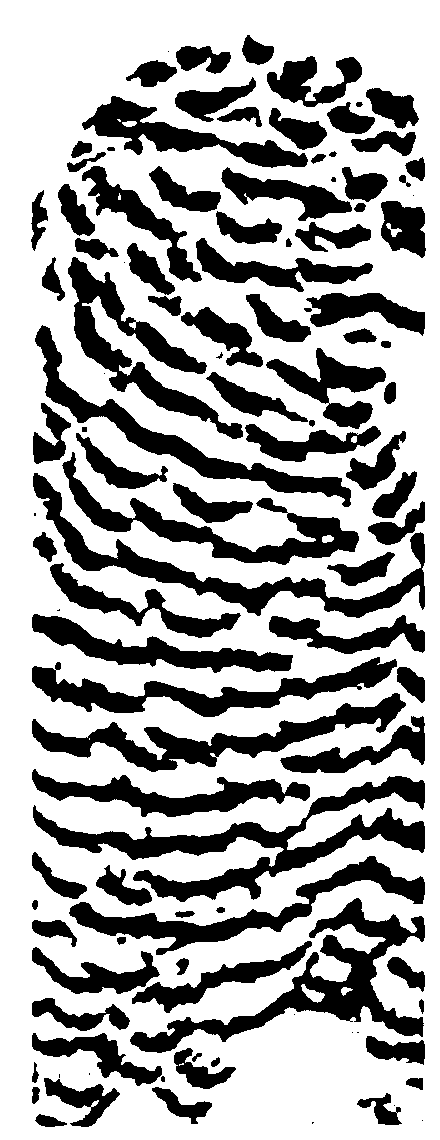

Supplement: Supplemental Information 2 [file peerj-07-7813-s002.zip › Supplemental2/Fig09C_Owl.bmp]

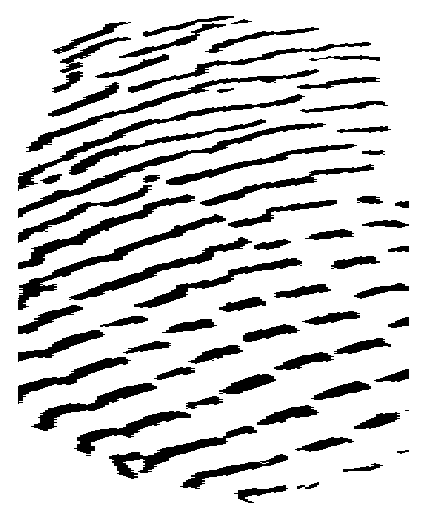

Supplement: Supplemental Information 2 [file peerj-07-7813-s002.zip › Supplemental2/Fig09D_Harpactes1.bmp]

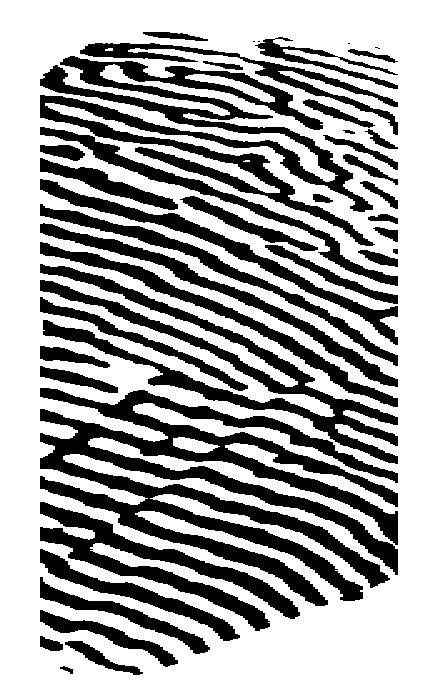

Supplement: Supplemental Information 2 [file peerj-07-7813-s002.zip › Supplemental2/Fig09E_Harpactes2.bmp]

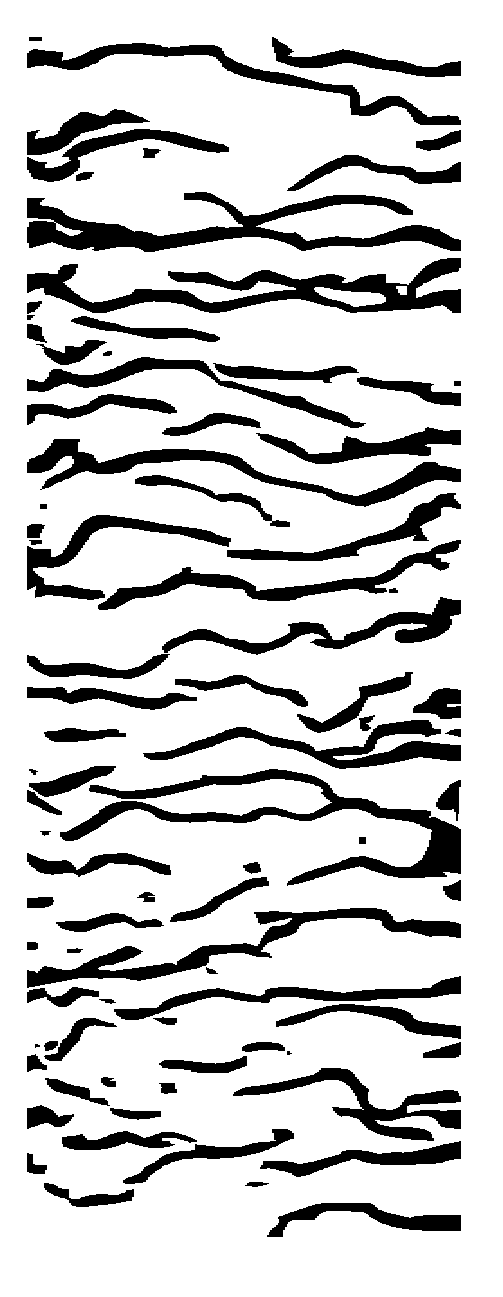

Supplement: Supplemental Information 2 [file peerj-07-7813-s002.zip › Supplemental2/Fig10_Hair.bmp]

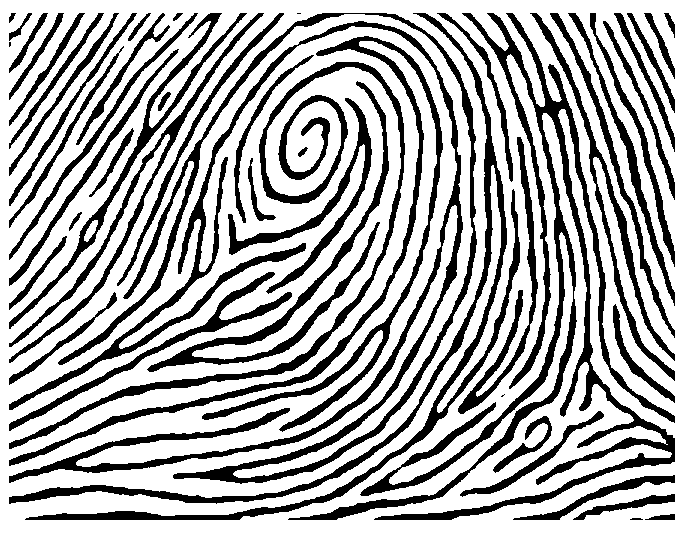

Supplement: Supplemental Information 2 [file peerj-07-7813-s002.zip › Supplemental2/Fig11_CentPocketLoop.bmp]

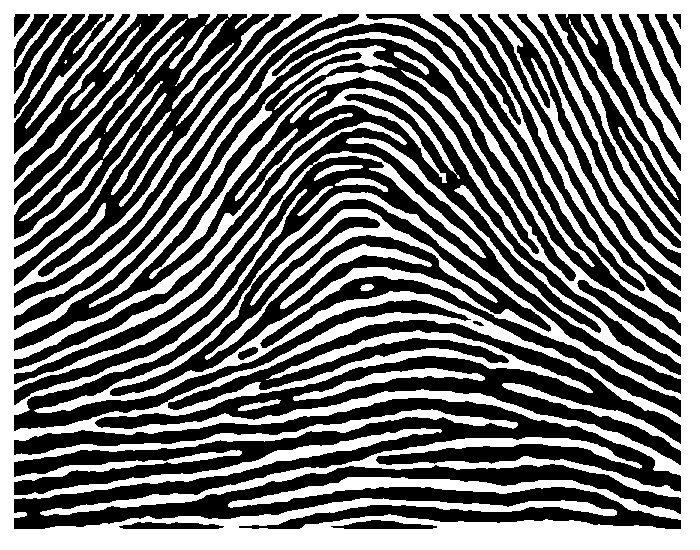

Supplement: Supplemental Information 2 [file peerj-07-7813-s002.zip › Supplemental2/Fig11_PlainArch.bmp]

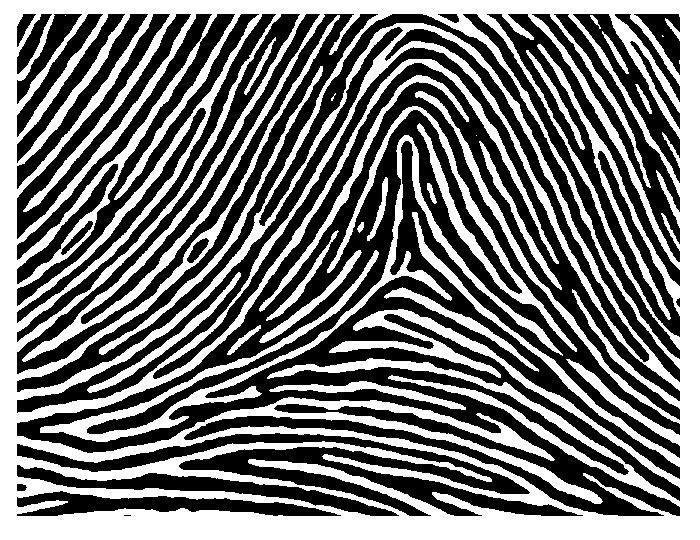

Supplement: Supplemental Information 2 [file peerj-07-7813-s002.zip › Supplemental2/Fig11_TentedArch.bmp]

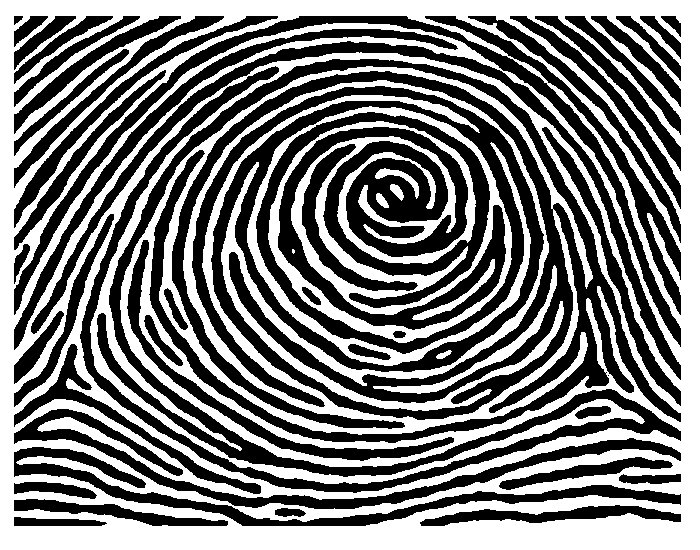

Supplement: Supplemental Information 2 [file peerj-07-7813-s002.zip › Supplemental2/Fig11_Whorl.bmp]

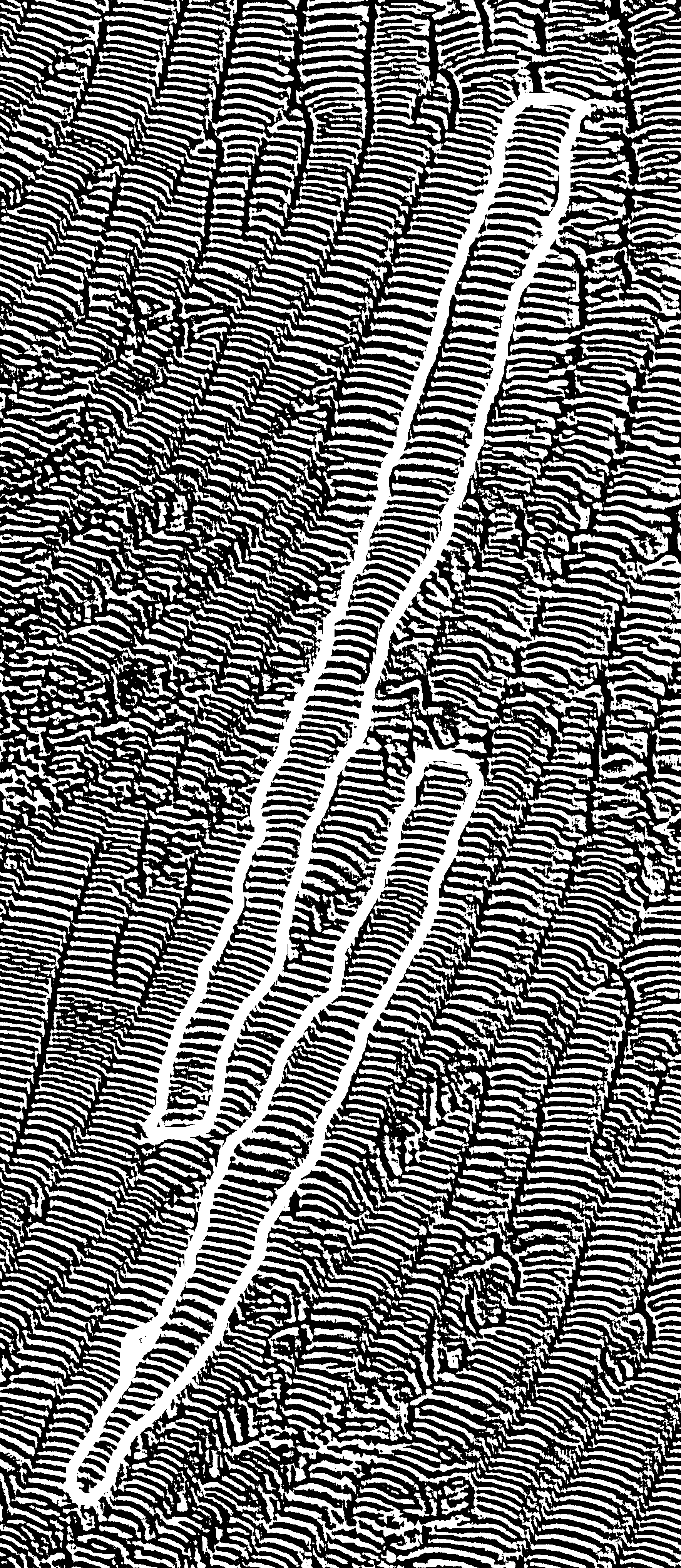

Supplement: Supplemental Information 2 [file peerj-07-7813-s002.zip › Supplemental2/Fig12_Mars.bmp]

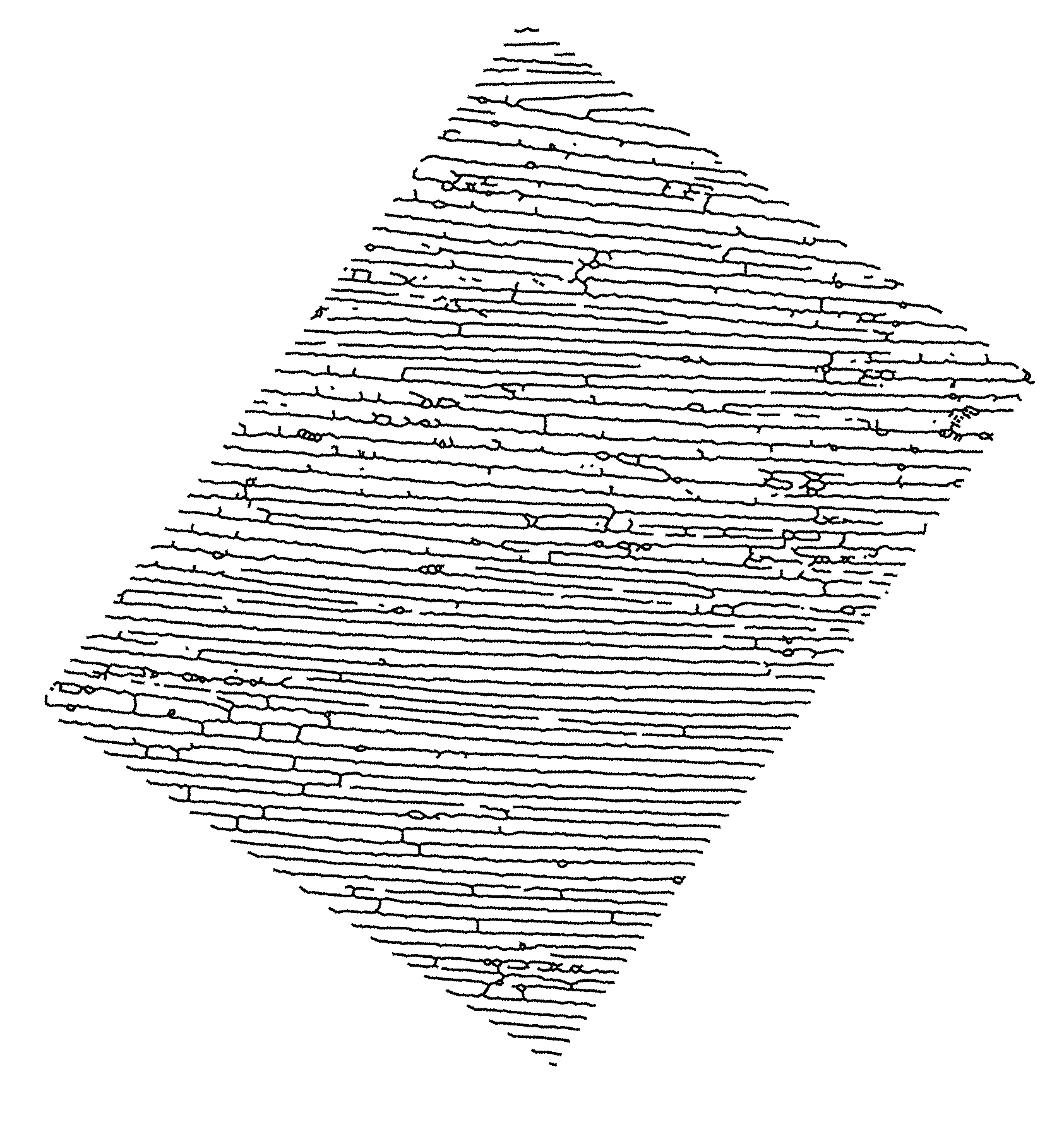

Supplement: Supplemental Information 2 [file peerj-07-7813-s002.zip › Supplemental2/Fig13_Material.bmp]

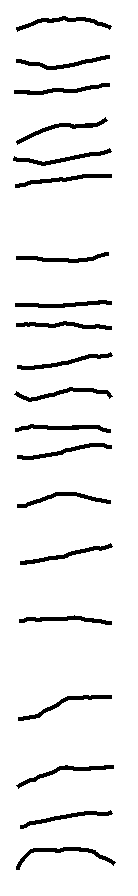

Supplement: Supplemental Information 2 [file peerj-07-7813-s002.zip › Supplemental2/Fig14_Corpuscle.bmp]

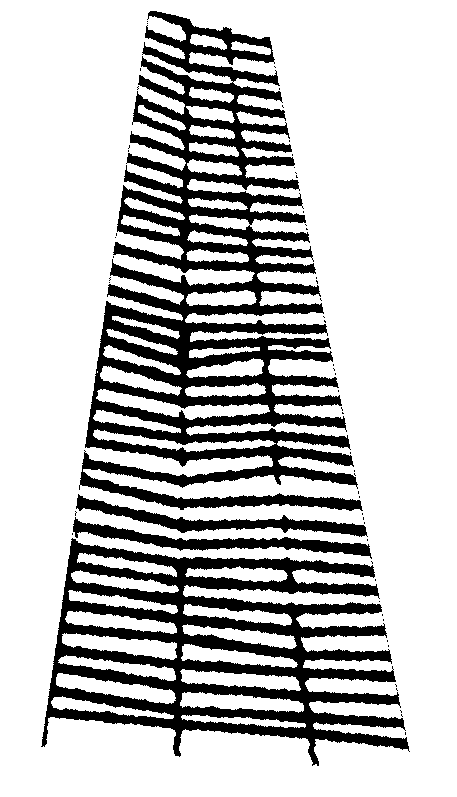

Supplement: Supplemental Information 2 [file peerj-07-7813-s002.zip › Supplemental2/Fig16_SpiderWeb.png]

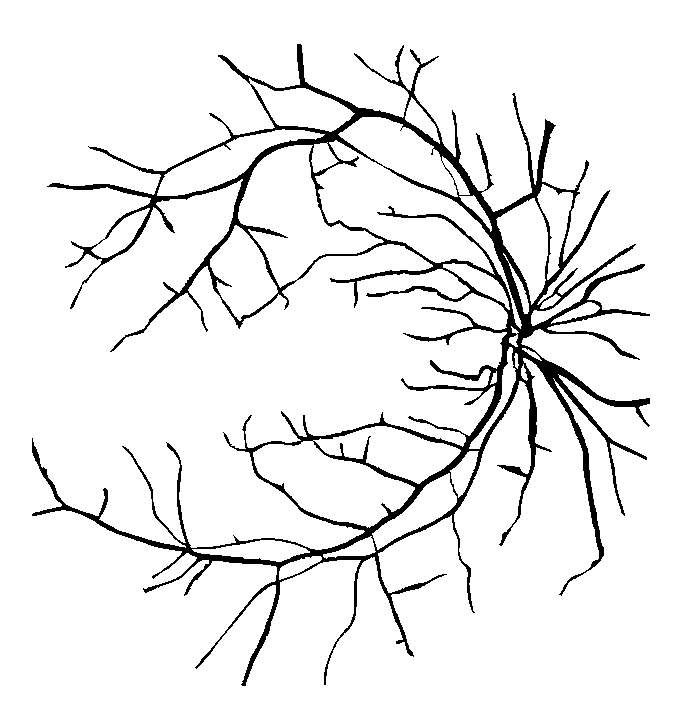

Supplement: Supplemental Information 2 [file peerj-07-7813-s002.zip › Supplemental2/Fig21A_BeforeChanges.bmp]

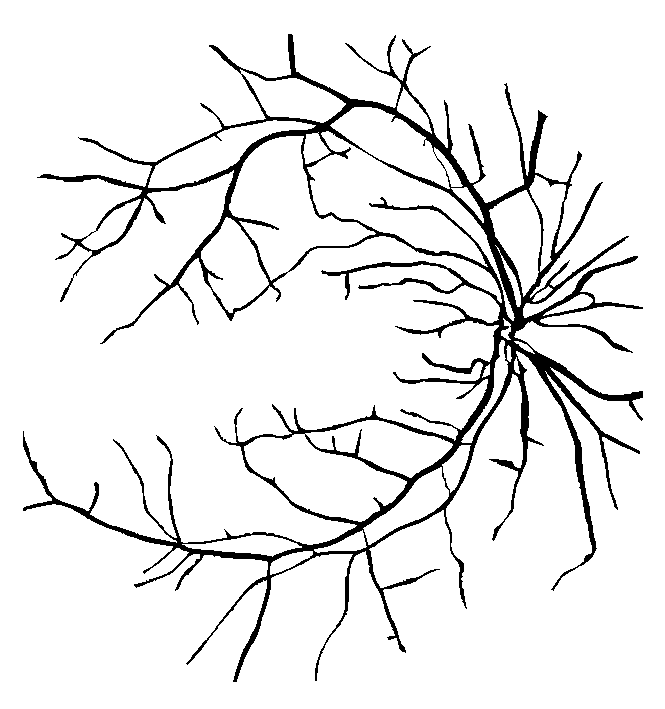

Supplement: Supplemental Information 2 [file peerj-07-7813-s002.zip › Supplemental2/Fig21B_AfterChanges.bmp]

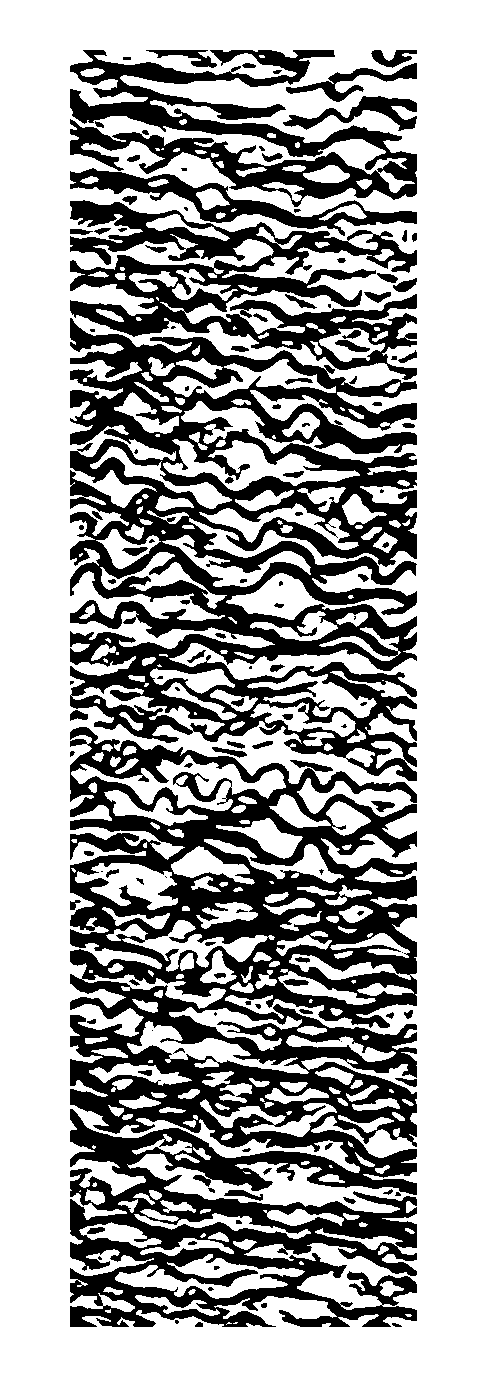

Supplement: Supplemental Information 2 [file peerj-07-7813-s002.zip › Supplemental2/Fig8A.bmp]

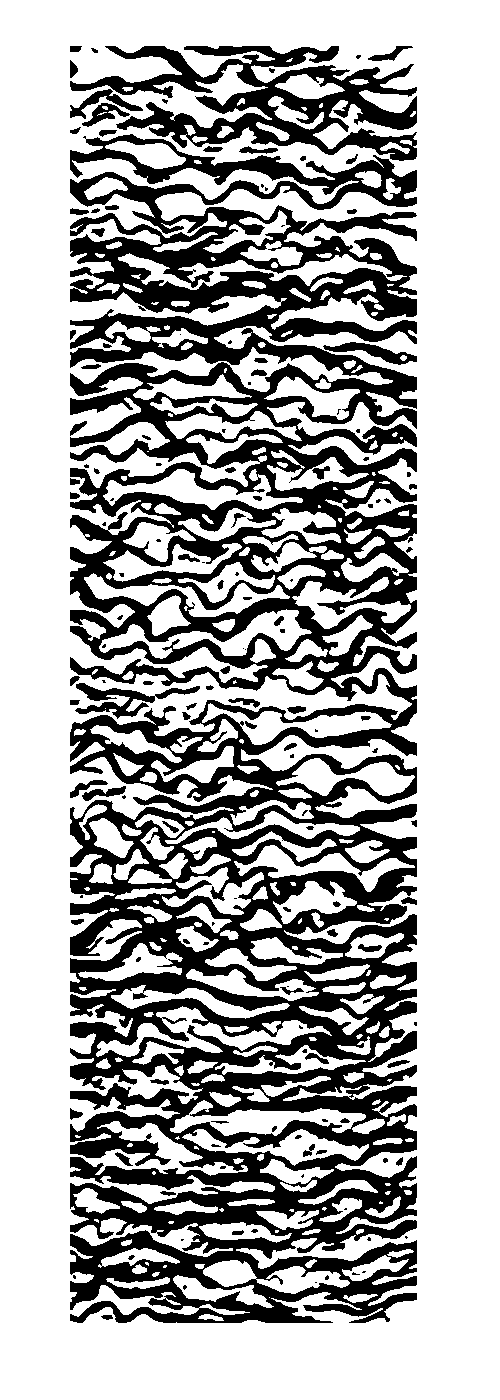

Supplement: Supplemental Information 2 [file peerj-07-7813-s002.zip › Supplemental2/Fig8B.bmp]

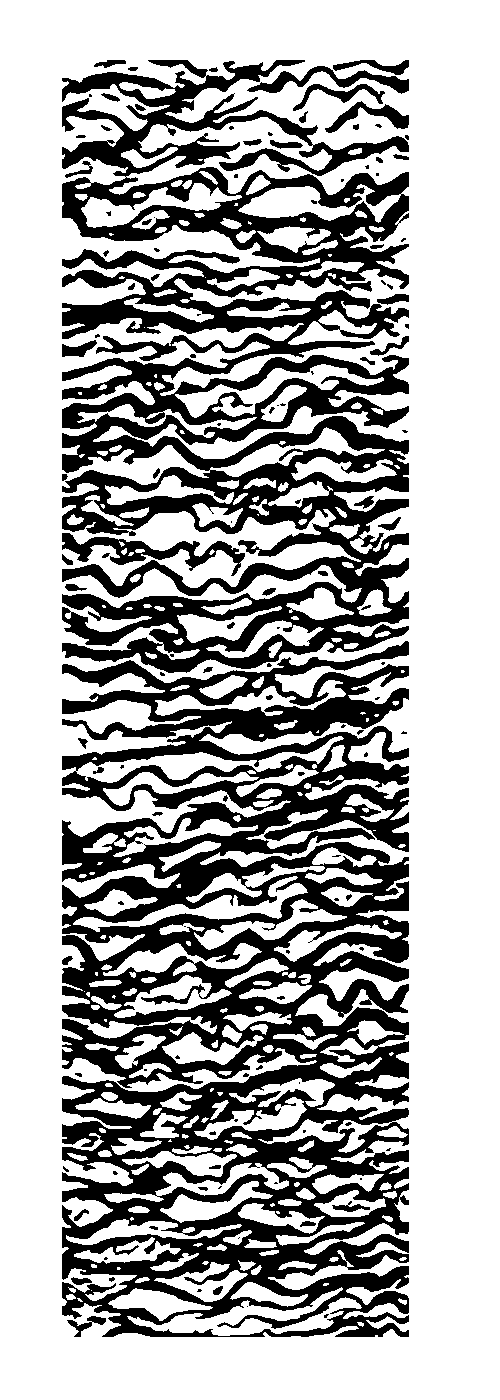

Supplement: Supplemental Information 2 [file peerj-07-7813-s002.zip › Supplemental2/Fig8C.bmp]

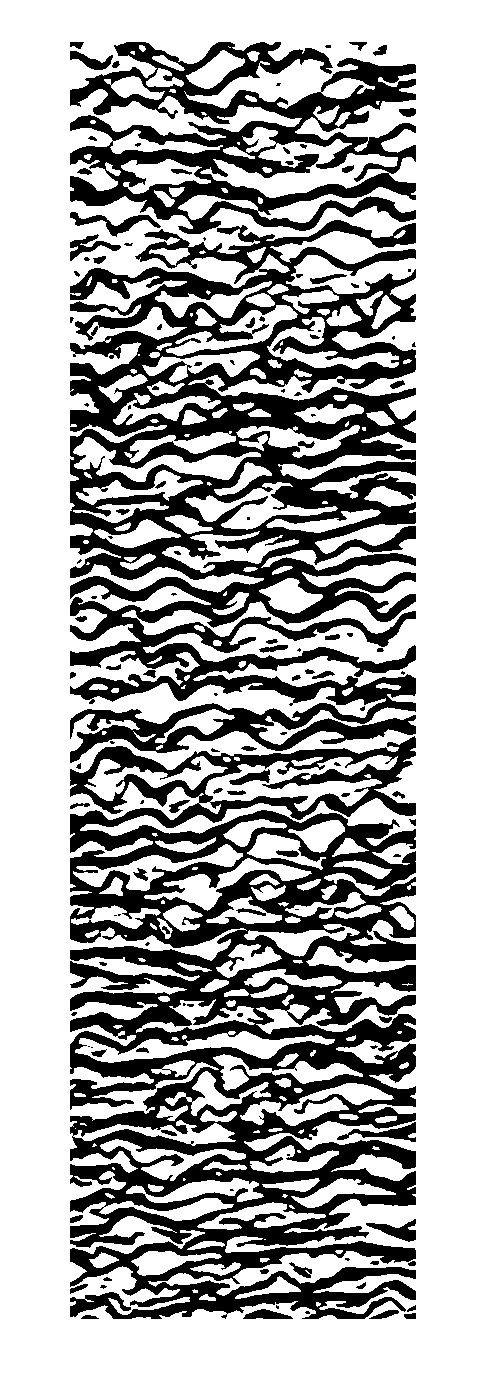

Supplement: Supplemental Information 2 [file peerj-07-7813-s002.zip › Supplemental2/Fig8D.bmp]

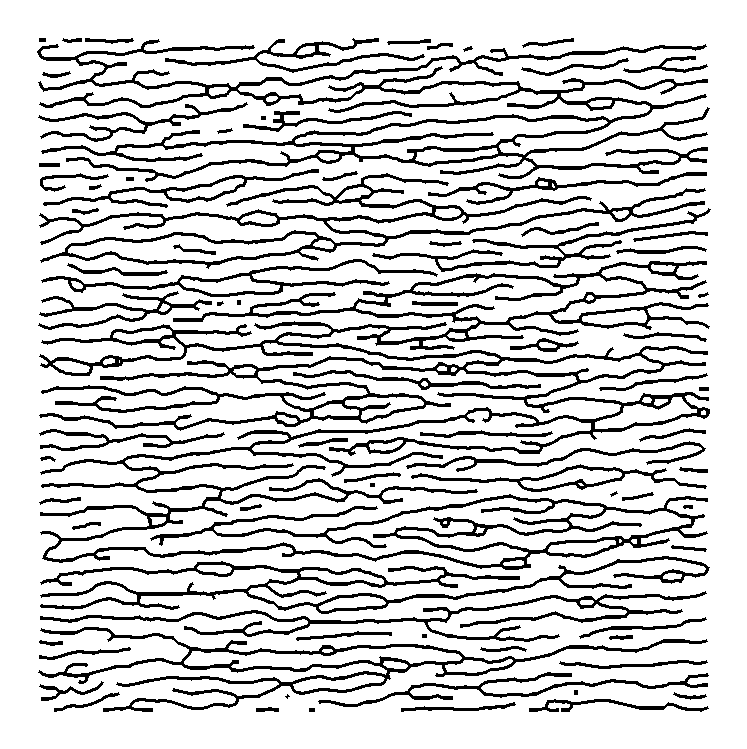

Supplement: Supplemental Information 3 [file peerj-07-7813-s003.zip › Supplemental-3/A-07-1.bmp]

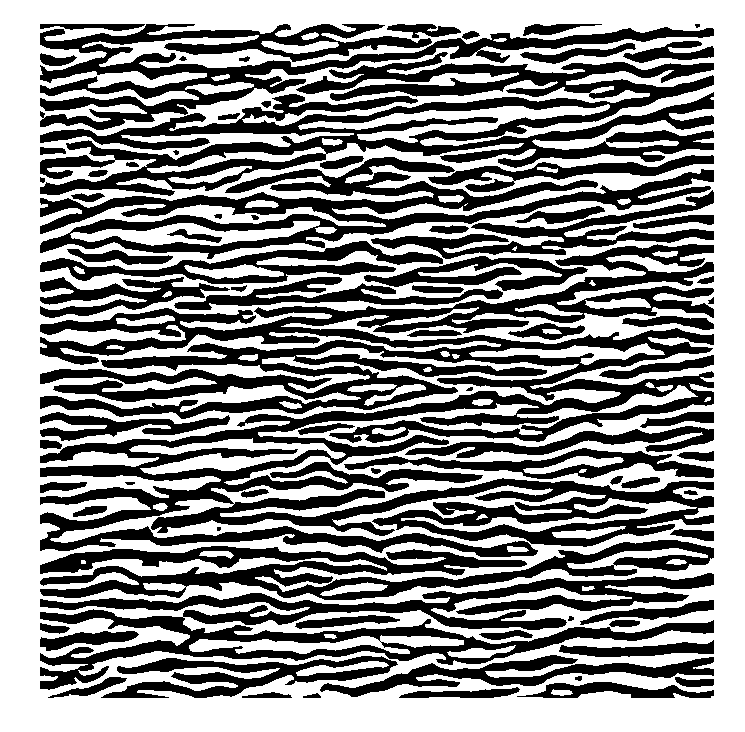

Supplement: Supplemental Information 3 [file peerj-07-7813-s003.zip › Supplemental-3/A-07.bmp]

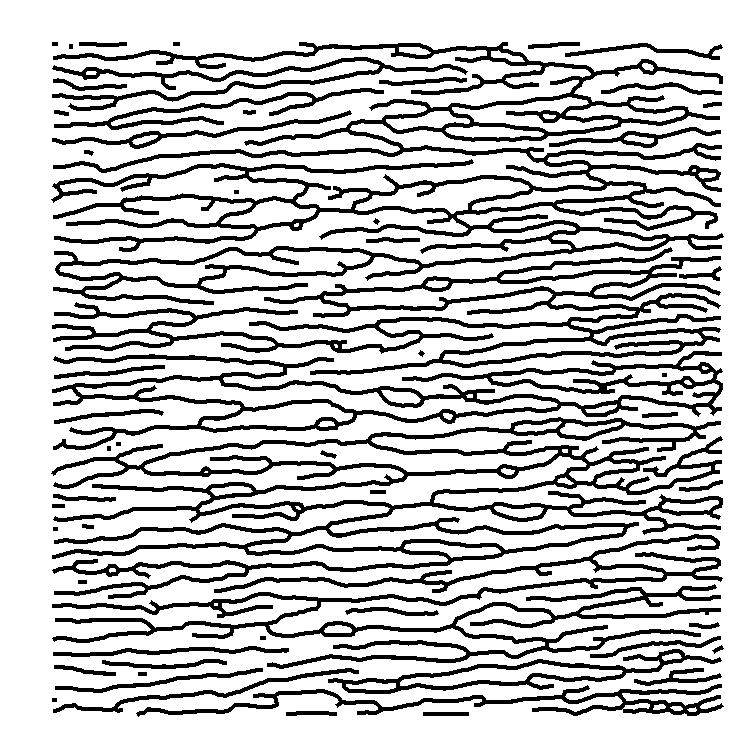

Supplement: Supplemental Information 3 [file peerj-07-7813-s003.zip › Supplemental-3/B-07-1.bmp]

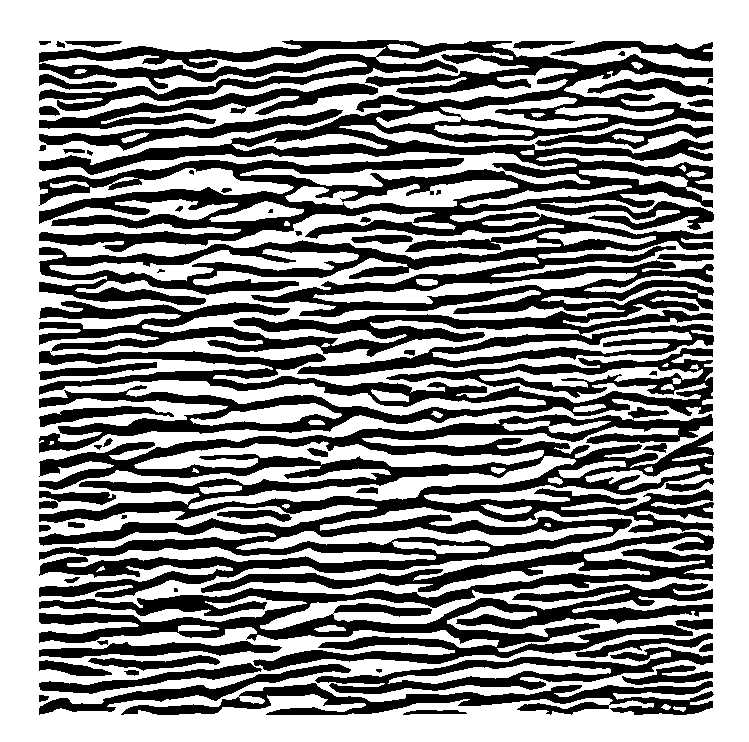

Supplement: Supplemental Information 3 [file peerj-07-7813-s003.zip › Supplemental-3/B-07.bmp]

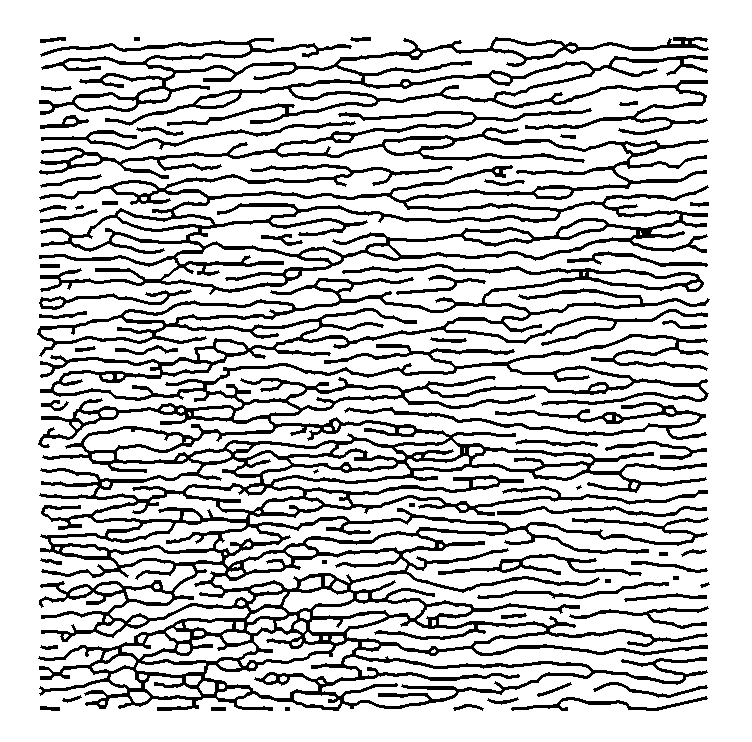

Supplement: Supplemental Information 3 [file peerj-07-7813-s003.zip › Supplemental-3/B-08-1.bmp]

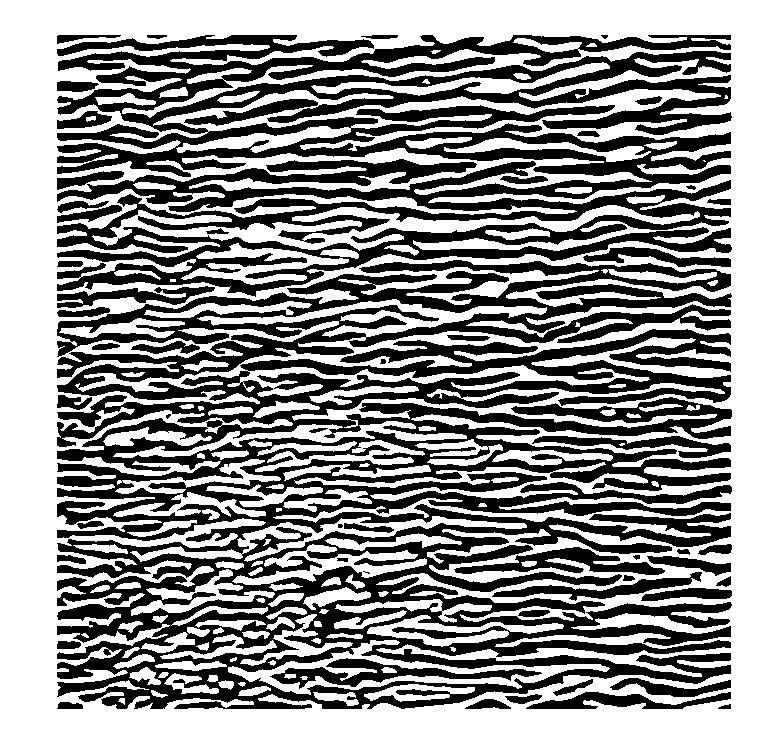

Supplement: Supplemental Information 3 [file peerj-07-7813-s003.zip › Supplemental-3/B-08.bmp]

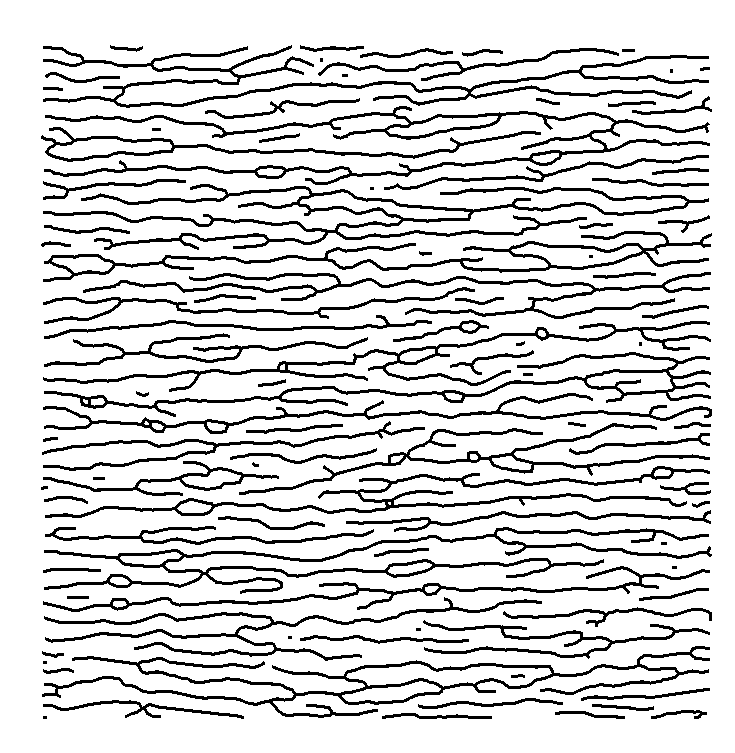

Supplement: Supplemental Information 3 [file peerj-07-7813-s003.zip › Supplemental-3/C-06-1.bmp]

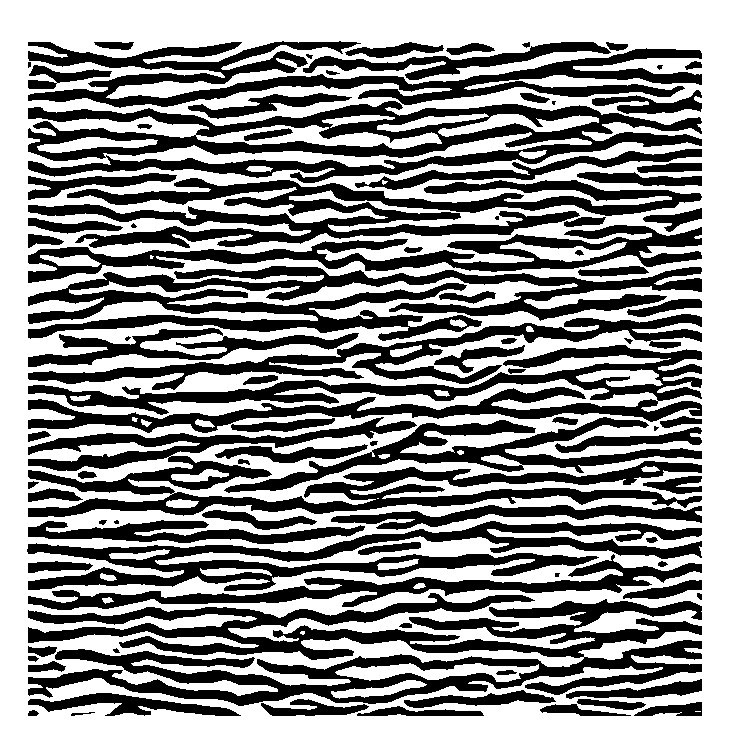

Supplement: Supplemental Information 3 [file peerj-07-7813-s003.zip › Supplemental-3/C-06.bmp]

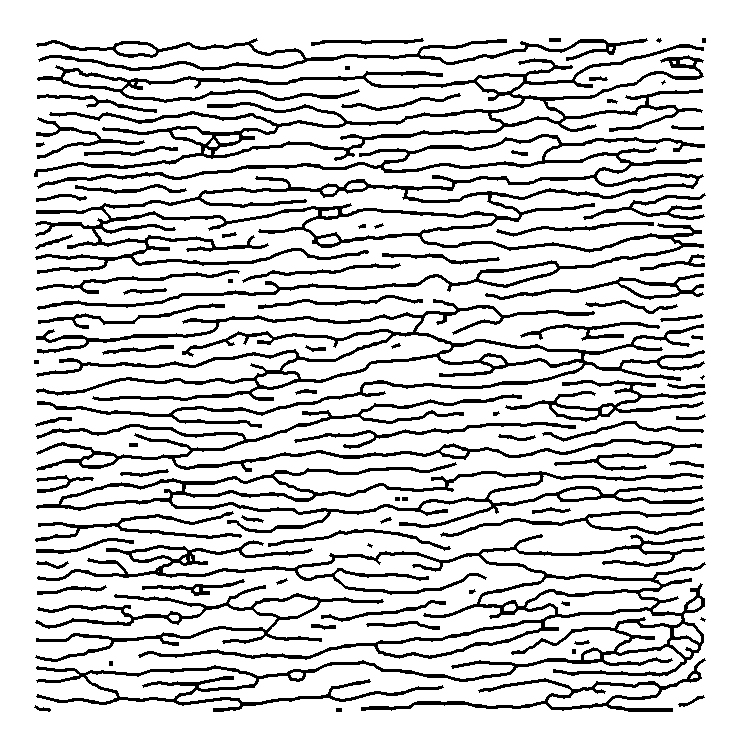

Supplement: Supplemental Information 3 [file peerj-07-7813-s003.zip › Supplemental-3/C-07-1.bmp]

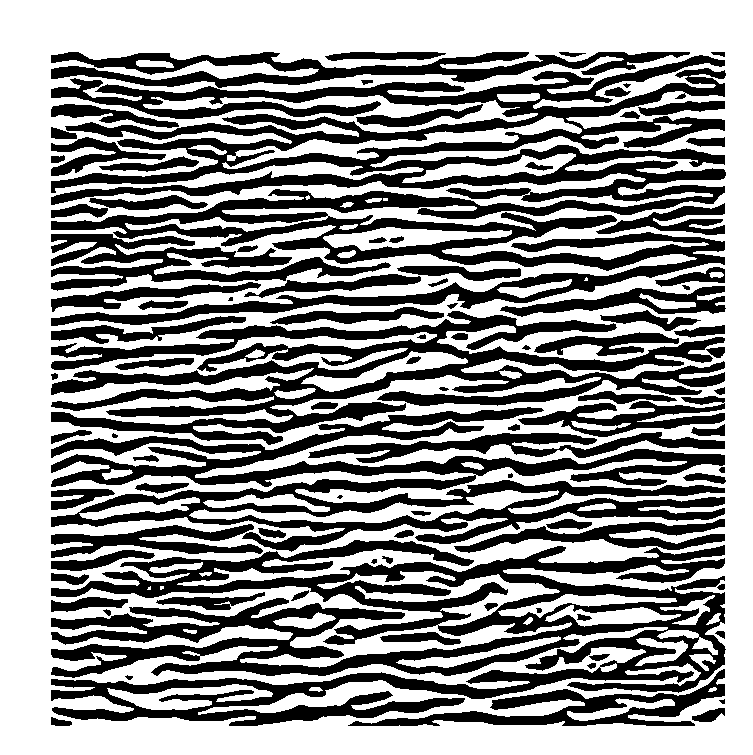

Supplement: Supplemental Information 3 [file peerj-07-7813-s003.zip › Supplemental-3/C-07.bmp]

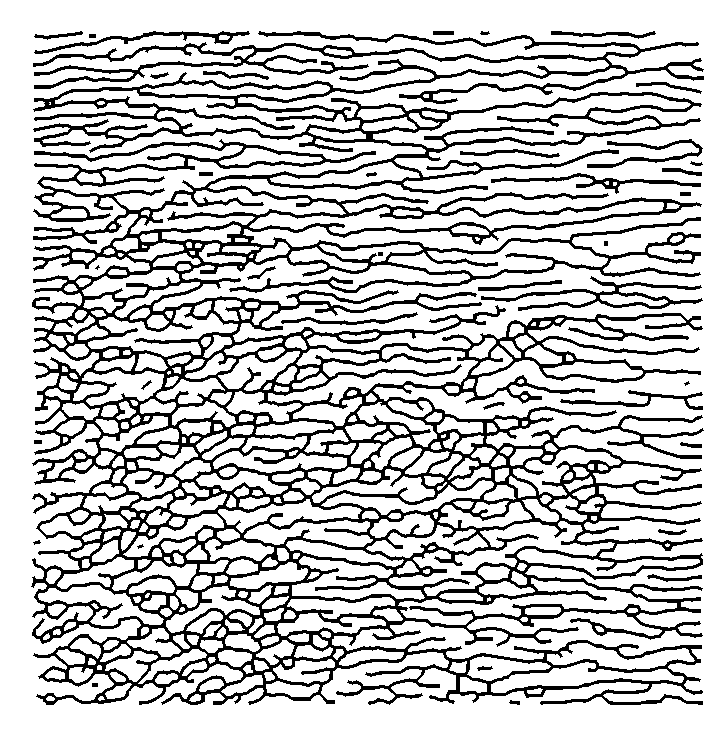

Supplement: Supplemental Information 3 [file peerj-07-7813-s003.zip › Supplemental-3/C-08-1.bmp]

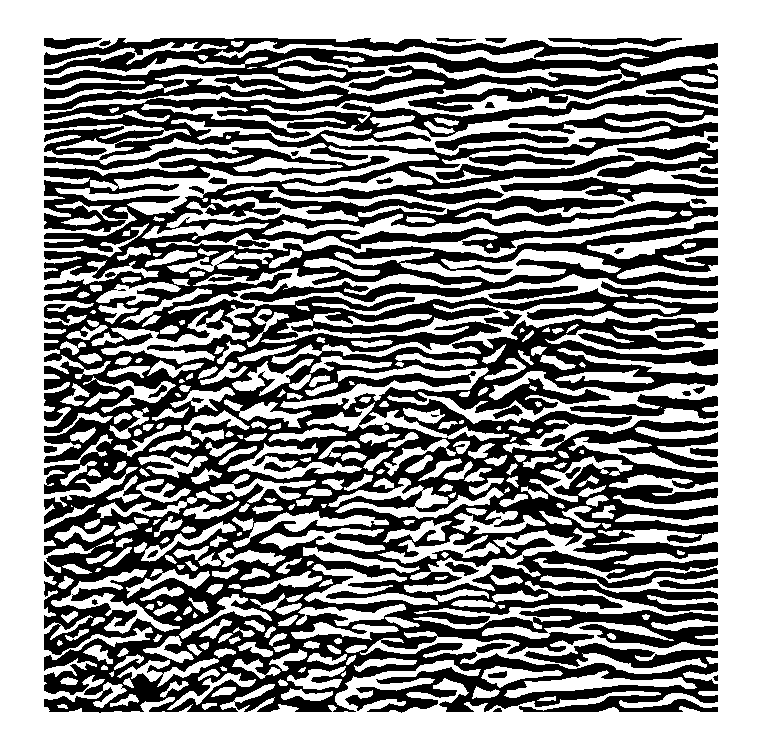

Supplement: Supplemental Information 3 [file peerj-07-7813-s003.zip › Supplemental-3/C-08.bmp]

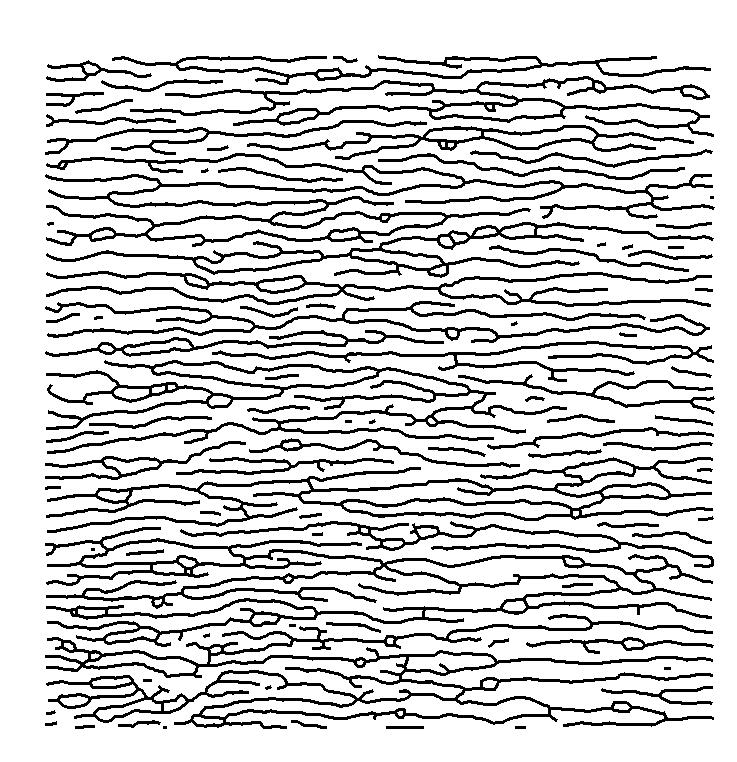

Supplement: Supplemental Information 3 [file peerj-07-7813-s003.zip › Supplemental-3/C-09-1.bmp]

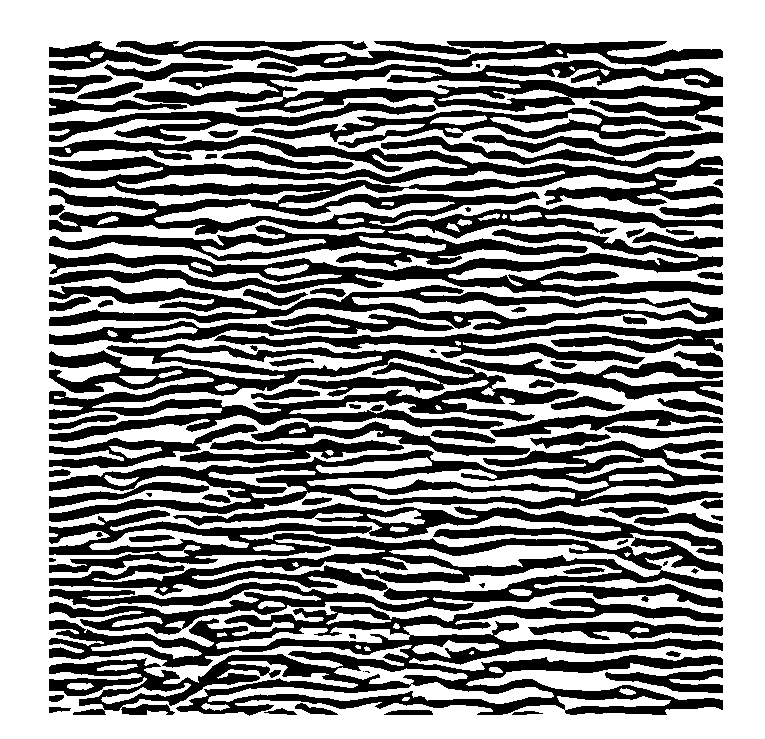

Supplement: Supplemental Information 3 [file peerj-07-7813-s003.zip › Supplemental-3/C-09.bmp]

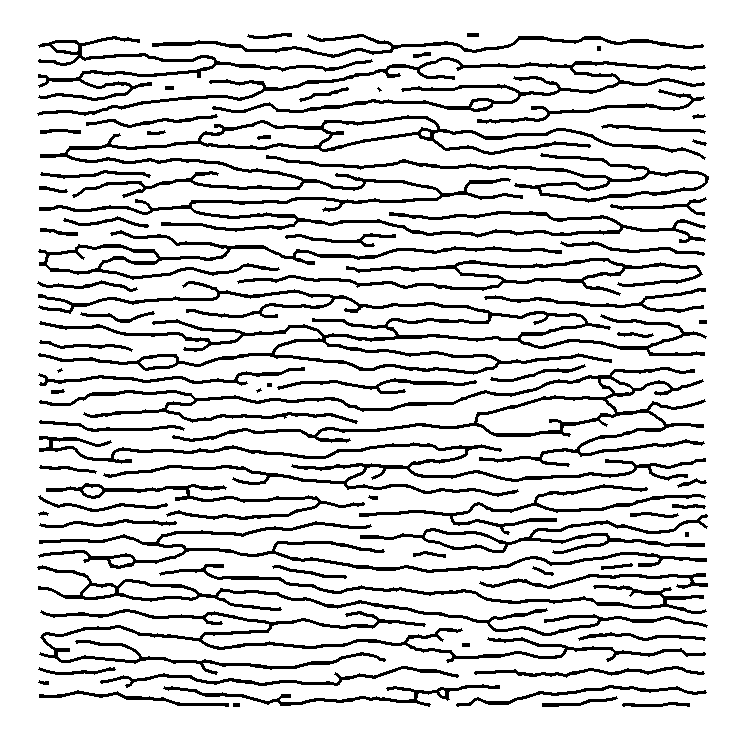

Supplement: Supplemental Information 3 [file peerj-07-7813-s003.zip › Supplemental-3/C-10-1.bmp]

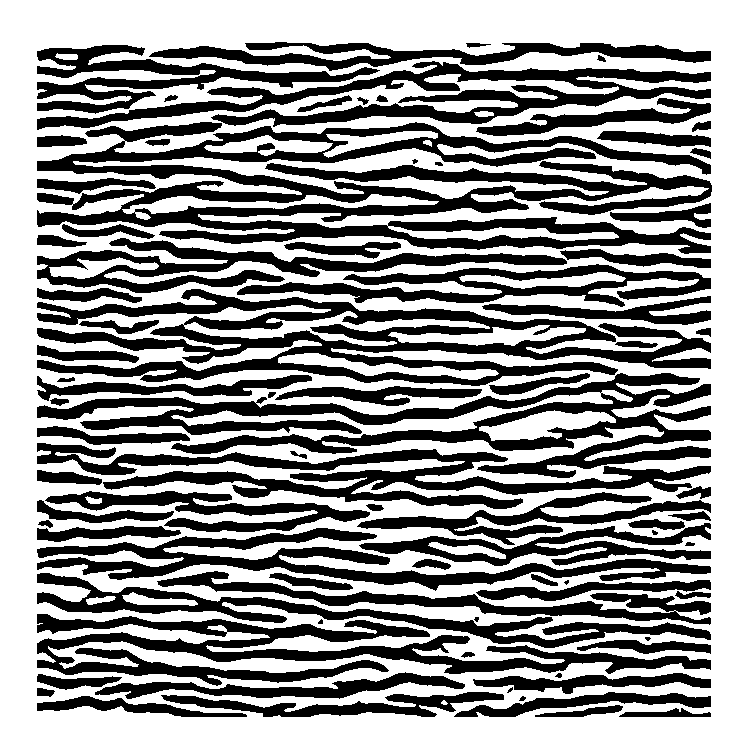

Supplement: Supplemental Information 3 [file peerj-07-7813-s003.zip › Supplemental-3/C-10.bmp]

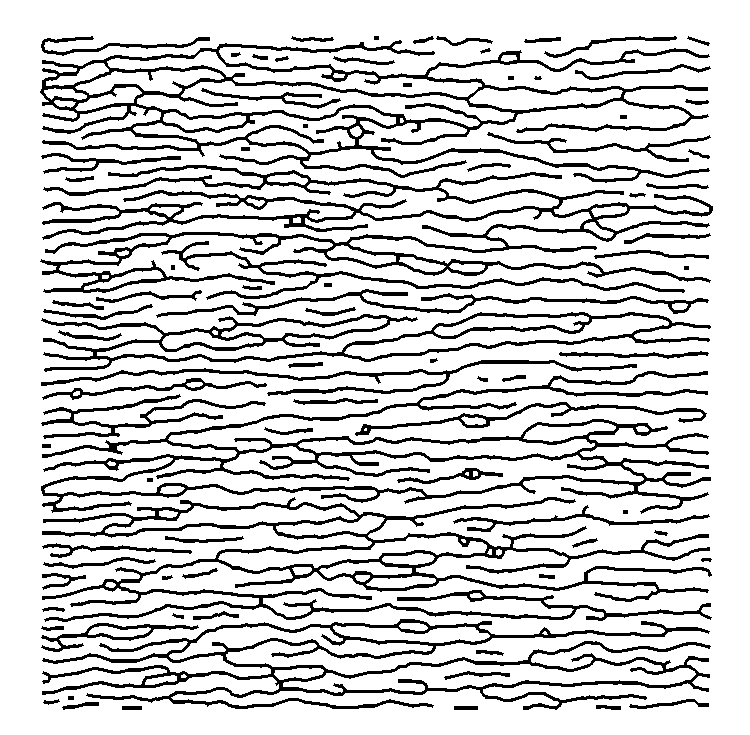

Supplement: Supplemental Information 3 [file peerj-07-7813-s003.zip › Supplemental-3/D-05-1.bmp]

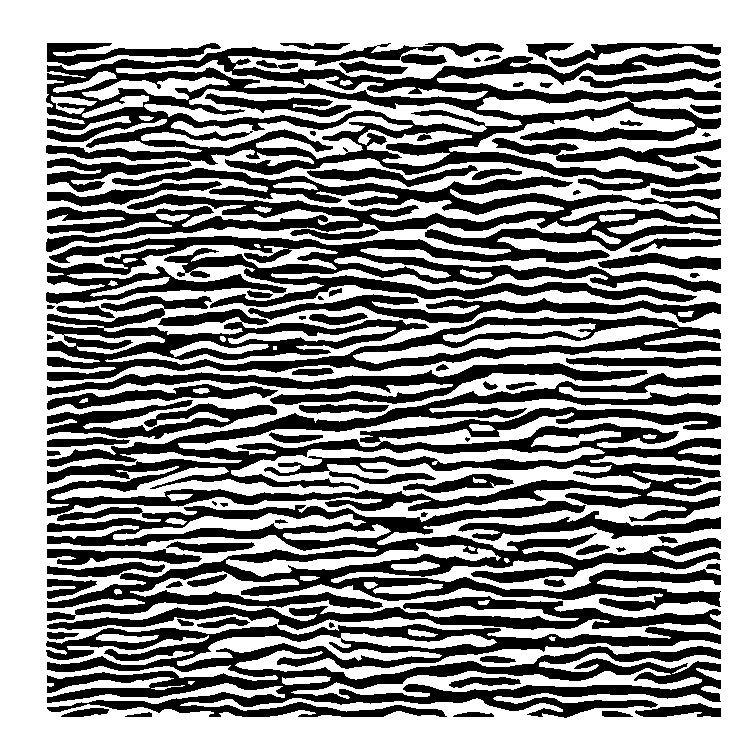

Supplement: Supplemental Information 3 [file peerj-07-7813-s003.zip › Supplemental-3/D-05.bmp]

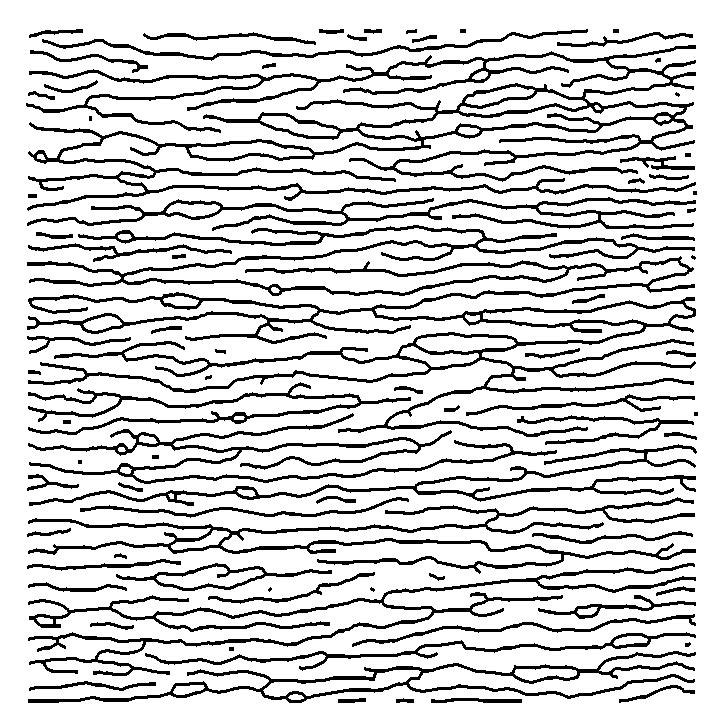

Supplement: Supplemental Information 3 [file peerj-07-7813-s003.zip › Supplemental-3/D-06-1.bmp]

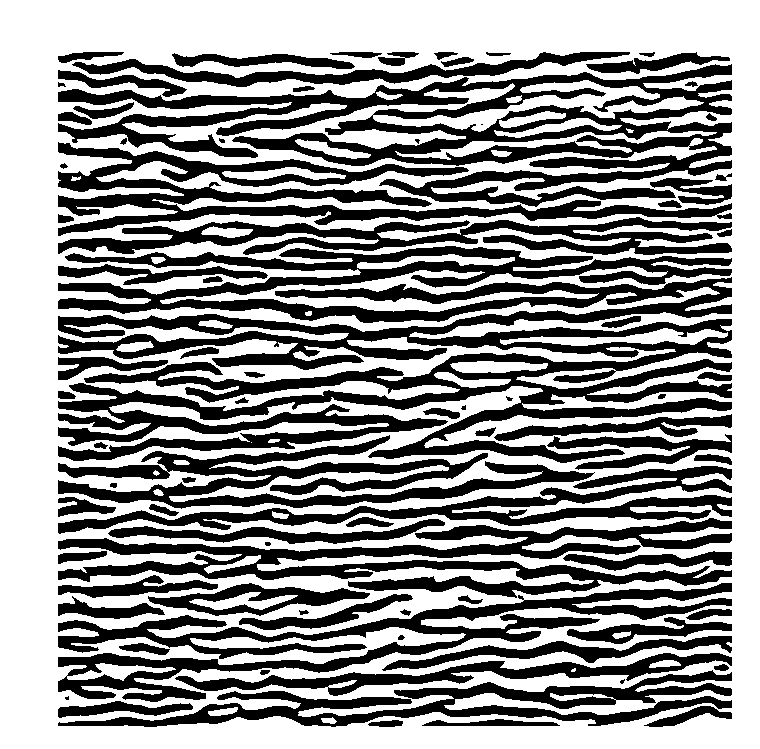

Supplement: Supplemental Information 3 [file peerj-07-7813-s003.zip › Supplemental-3/D-06.bmp]

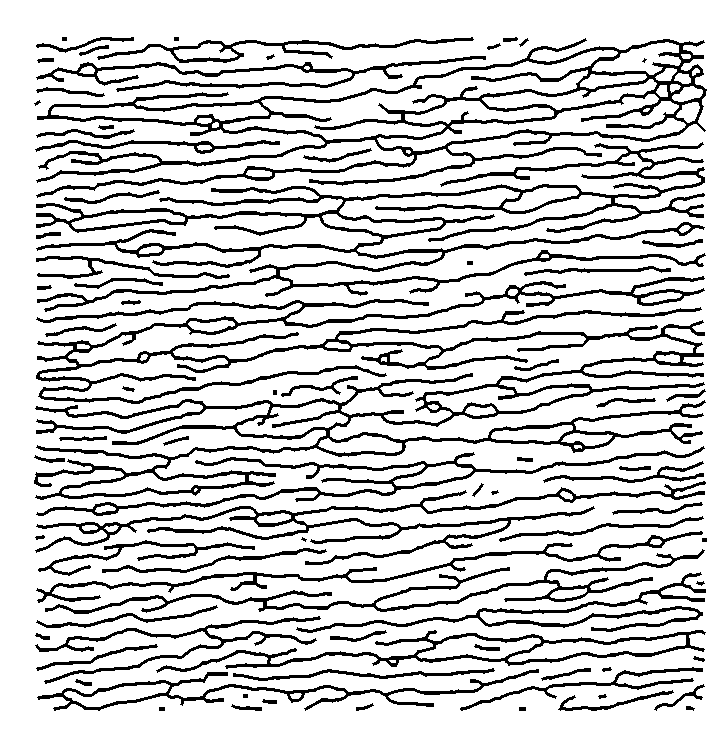

Supplement: Supplemental Information 3 [file peerj-07-7813-s003.zip › Supplemental-3/D-07-1.bmp]

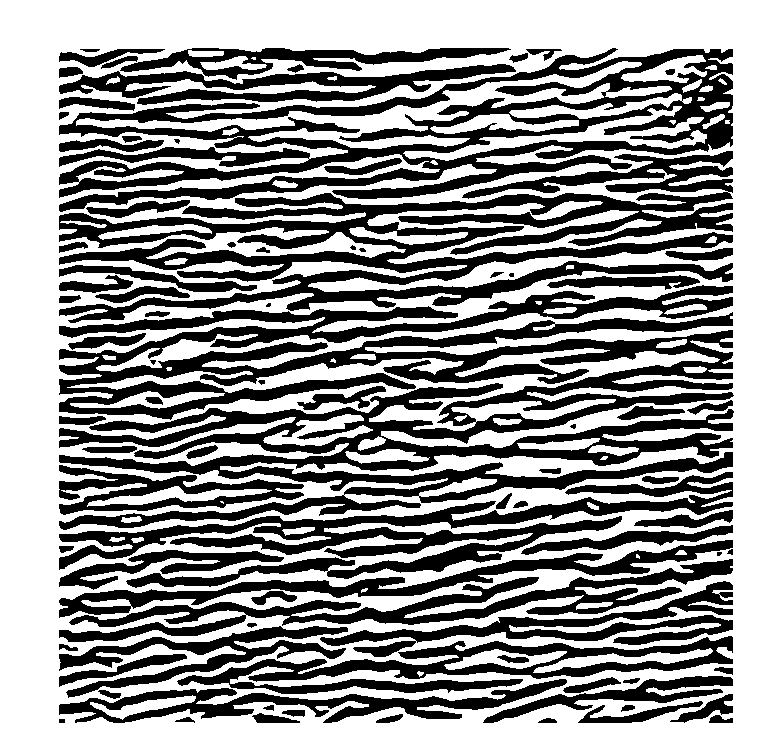

Supplement: Supplemental Information 3 [file peerj-07-7813-s003.zip › Supplemental-3/D-07.bmp]

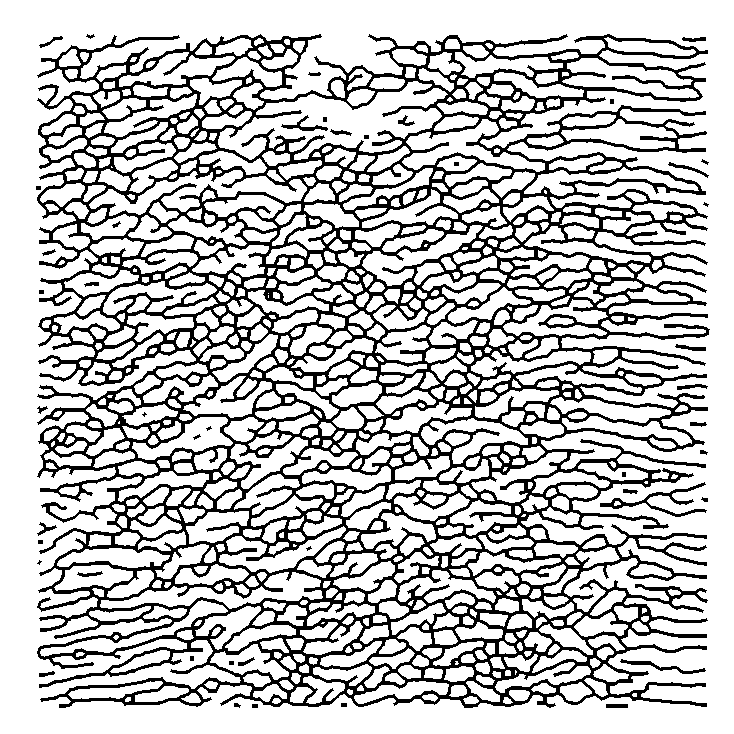

Supplement: Supplemental Information 3 [file peerj-07-7813-s003.zip › Supplemental-3/D-08-1.bmp]

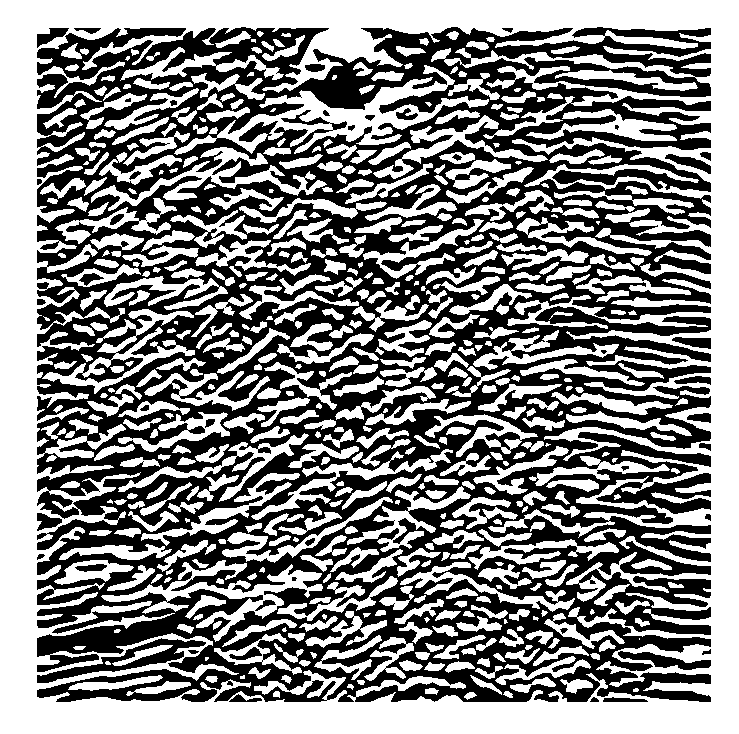

Supplement: Supplemental Information 3 [file peerj-07-7813-s003.zip › Supplemental-3/D-08.bmp]

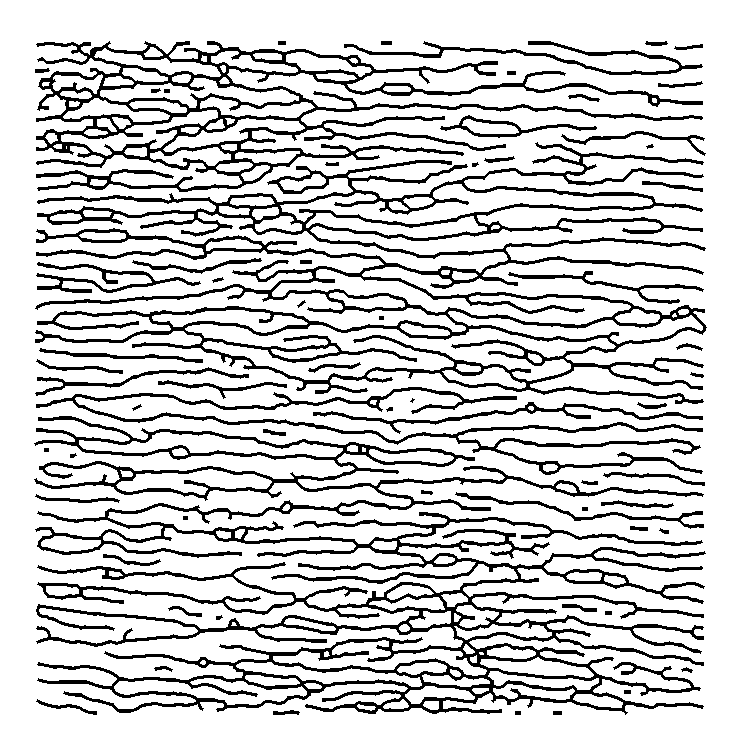

Supplement: Supplemental Information 3 [file peerj-07-7813-s003.zip › Supplemental-3/D-09-1.bmp]

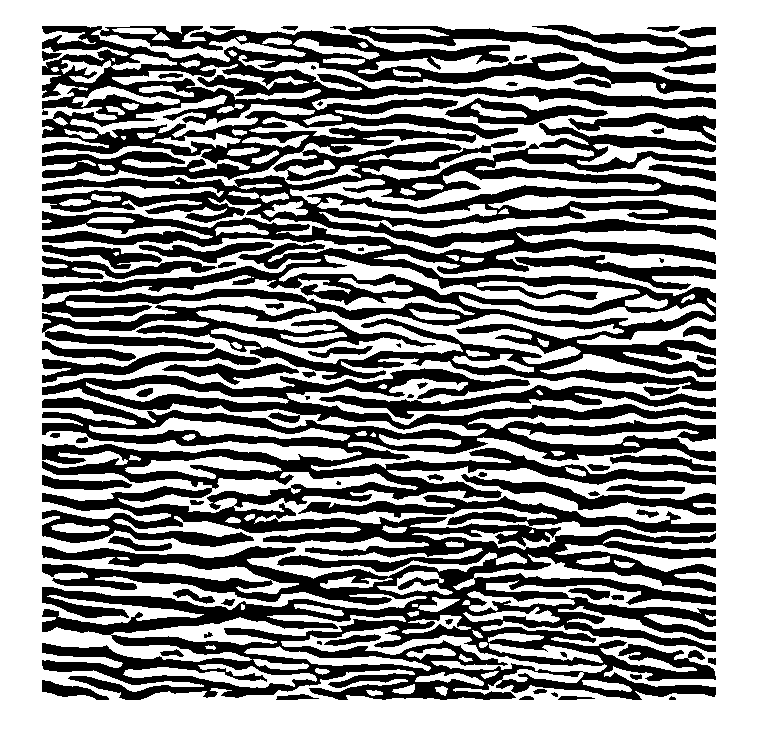

Supplement: Supplemental Information 3 [file peerj-07-7813-s003.zip › Supplemental-3/D-09.bmp]

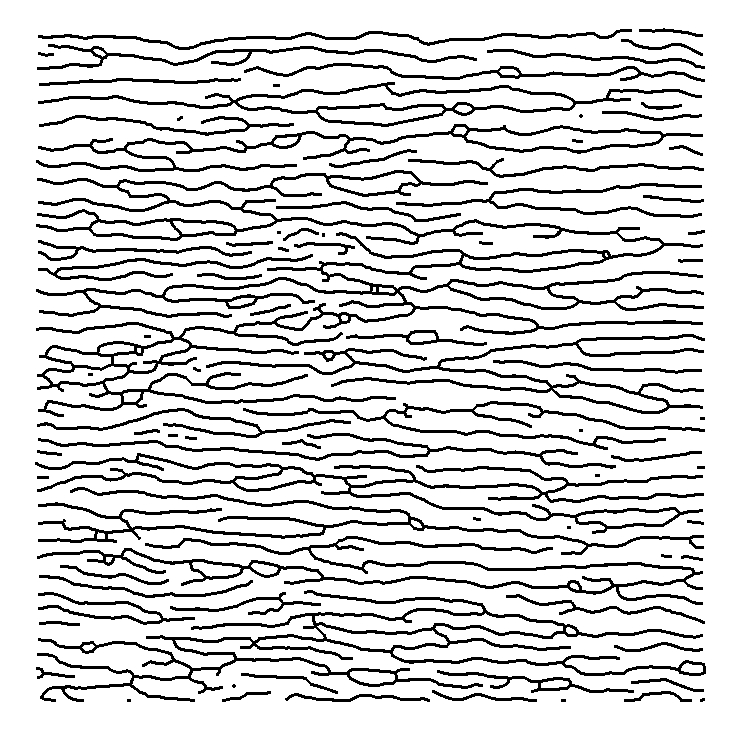

Supplement: Supplemental Information 3 [file peerj-07-7813-s003.zip › Supplemental-3/D-10-1.bmp]

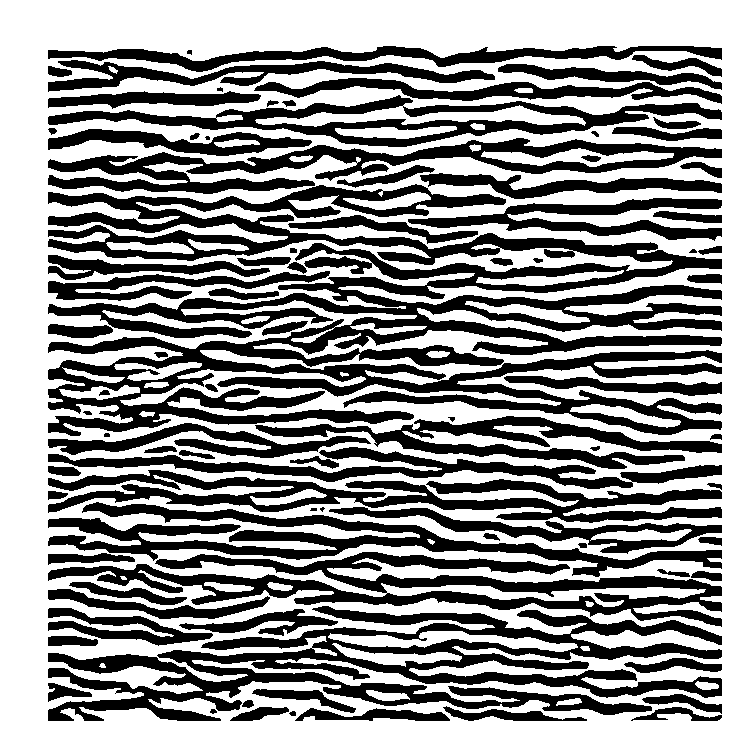

Supplement: Supplemental Information 3 [file peerj-07-7813-s003.zip › Supplemental-3/D-10.bmp]

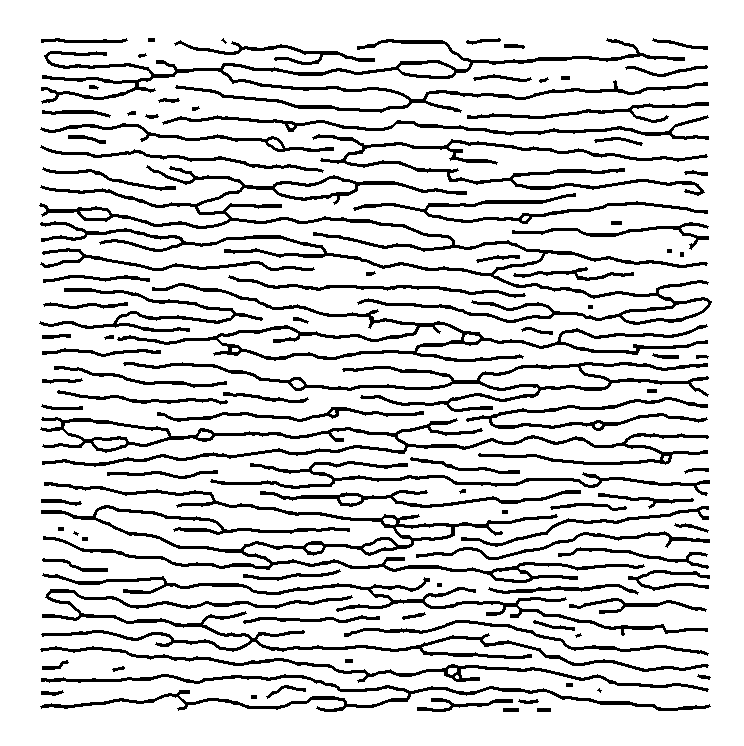

Supplement: Supplemental Information 3 [file peerj-07-7813-s003.zip › Supplemental-3/D-11-1.bmp]

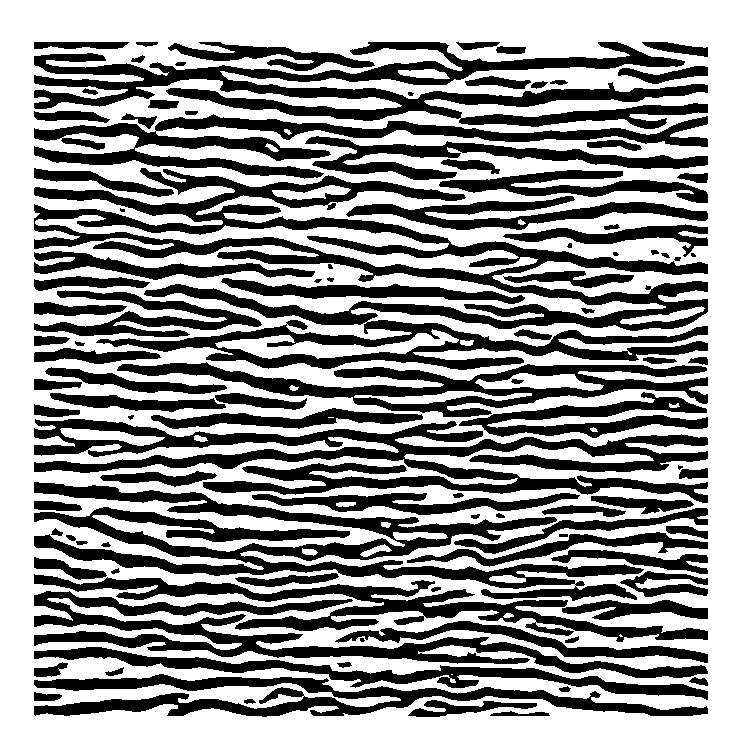

Supplement: Supplemental Information 3 [file peerj-07-7813-s003.zip › Supplemental-3/D-11.bmp]

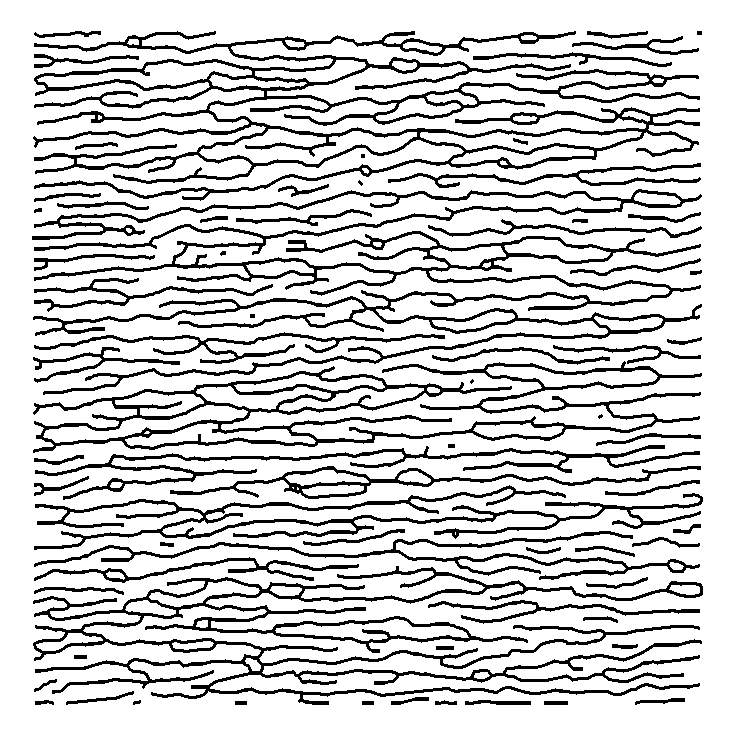

Supplement: Supplemental Information 3 [file peerj-07-7813-s003.zip › Supplemental-3/E-05-1.bmp]

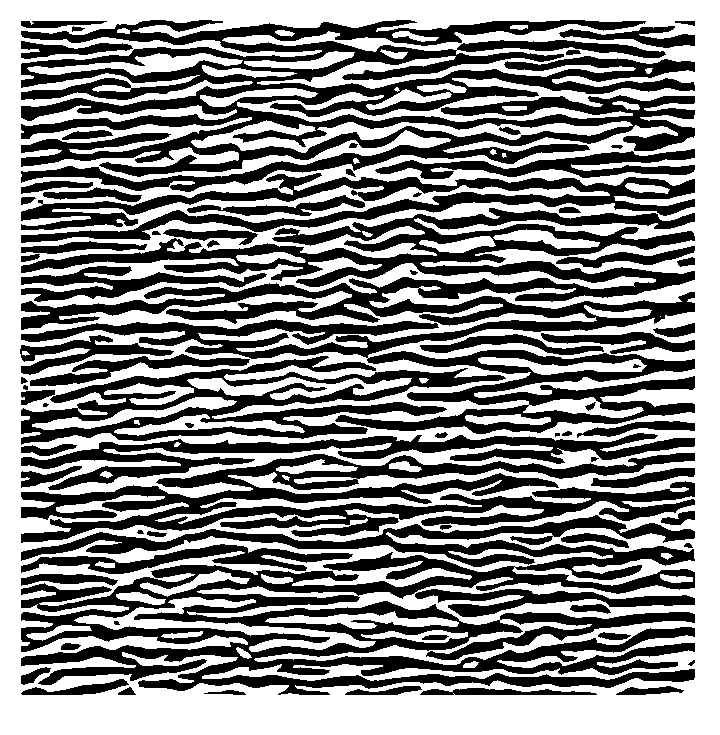

Supplement: Supplemental Information 3 [file peerj-07-7813-s003.zip › Supplemental-3/E-05.bmp]

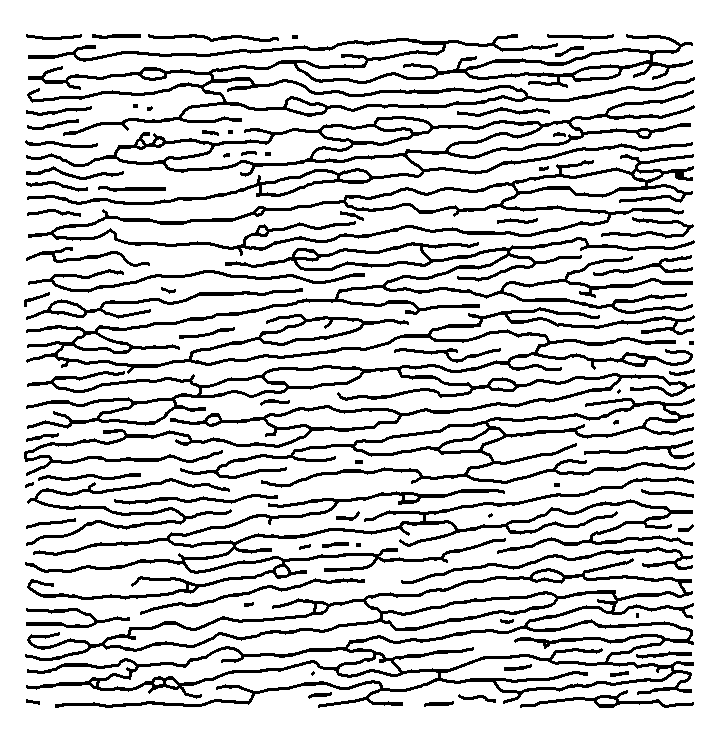

Supplement: Supplemental Information 3 [file peerj-07-7813-s003.zip › Supplemental-3/E-06-1.bmp]

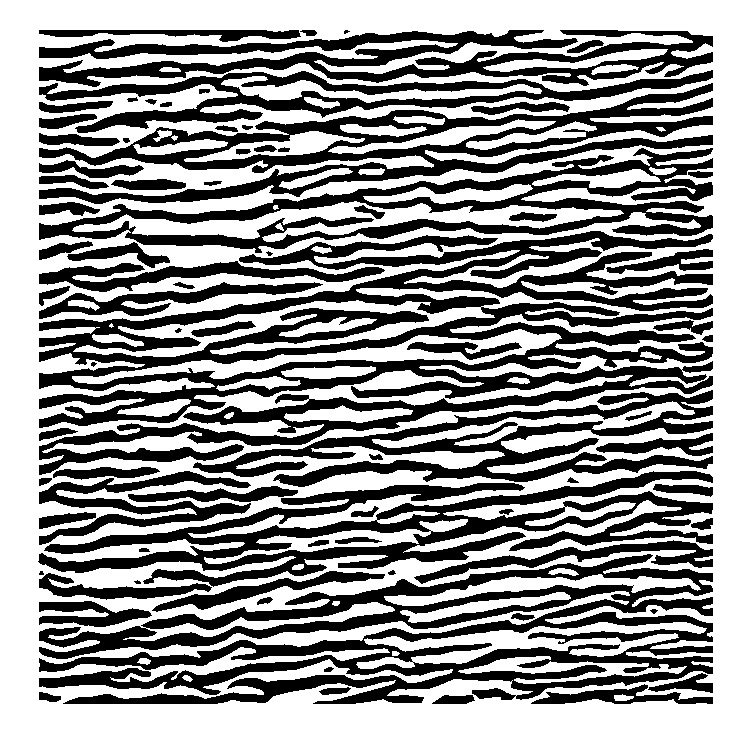

Supplement: Supplemental Information 3 [file peerj-07-7813-s003.zip › Supplemental-3/E-06.bmp]

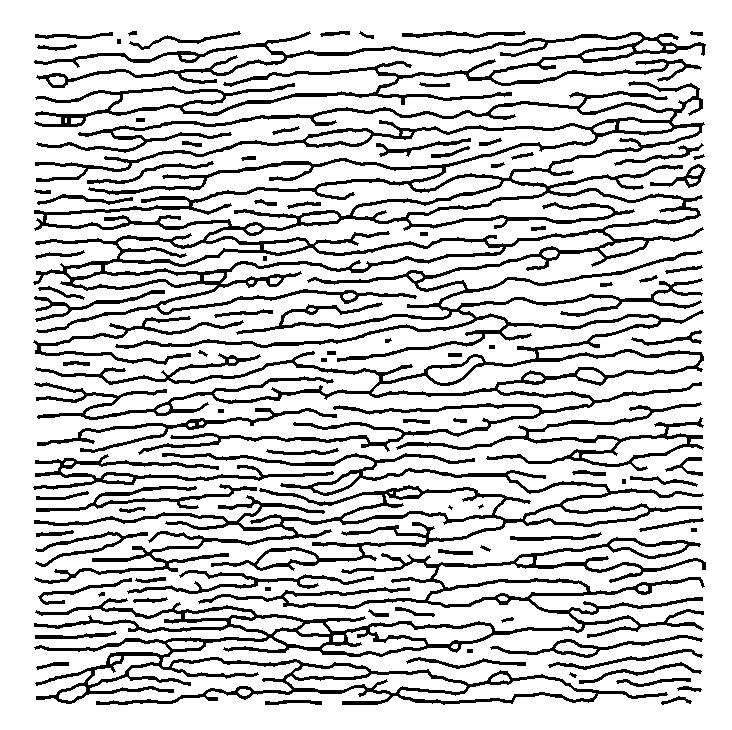

Supplement: Supplemental Information 3 [file peerj-07-7813-s003.zip › Supplemental-3/E-07-1.bmp]

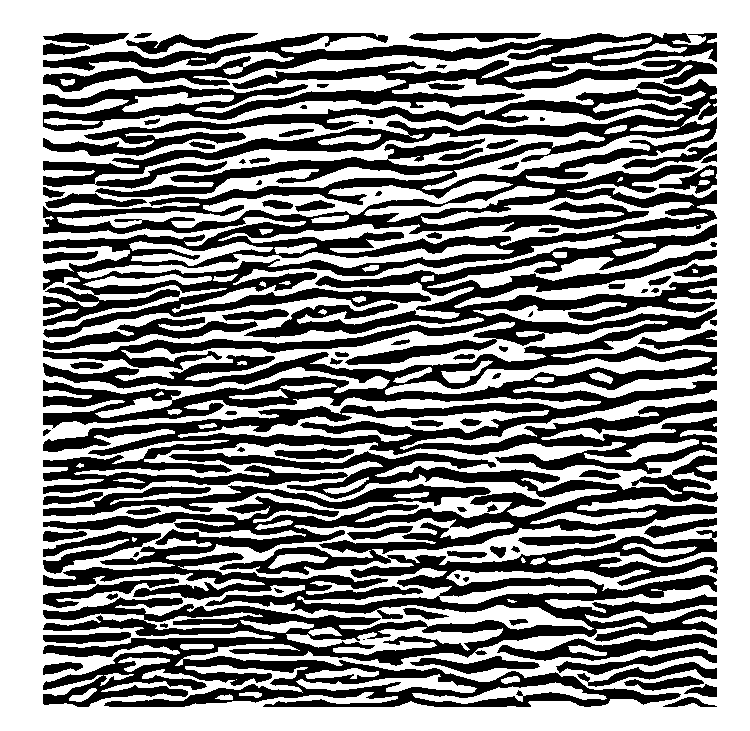

Supplement: Supplemental Information 3 [file peerj-07-7813-s003.zip › Supplemental-3/E-07.bmp]

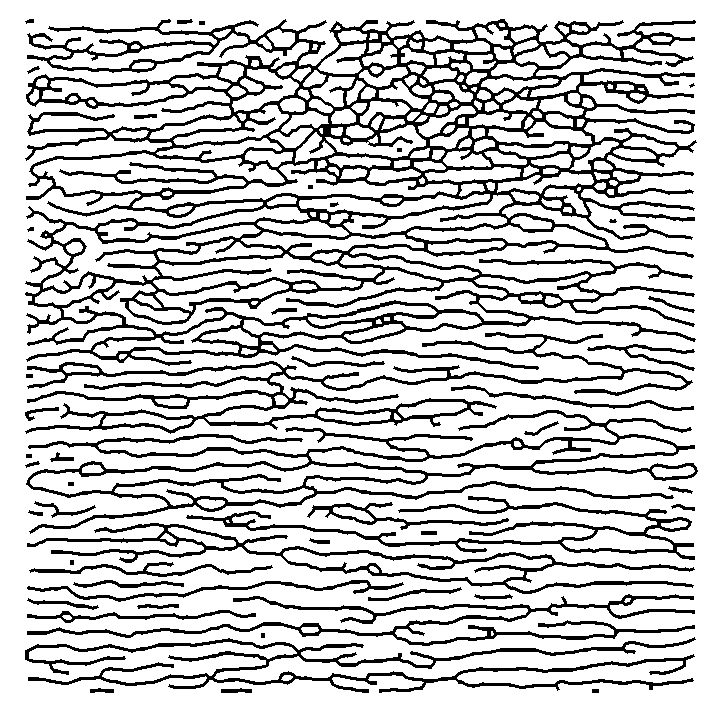

Supplement: Supplemental Information 3 [file peerj-07-7813-s003.zip › Supplemental-3/E-08-1.bmp]

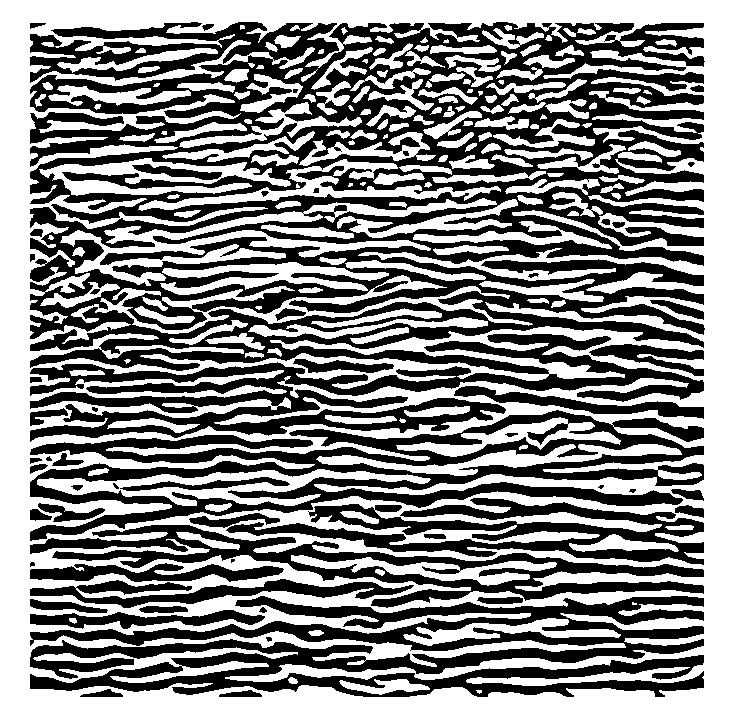

Supplement: Supplemental Information 3 [file peerj-07-7813-s003.zip › Supplemental-3/E-08.bmp]

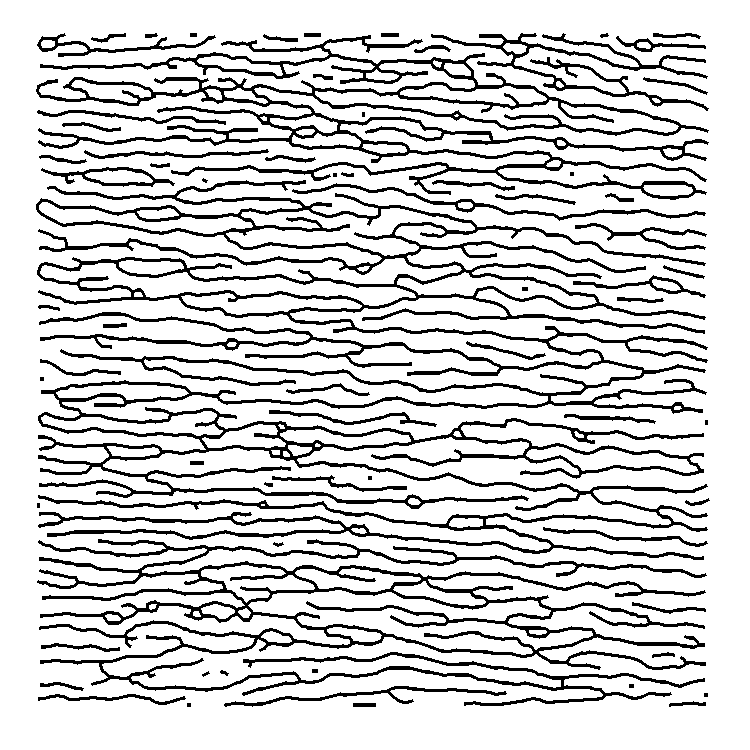

Supplement: Supplemental Information 3 [file peerj-07-7813-s003.zip › Supplemental-3/E-09-1.bmp]

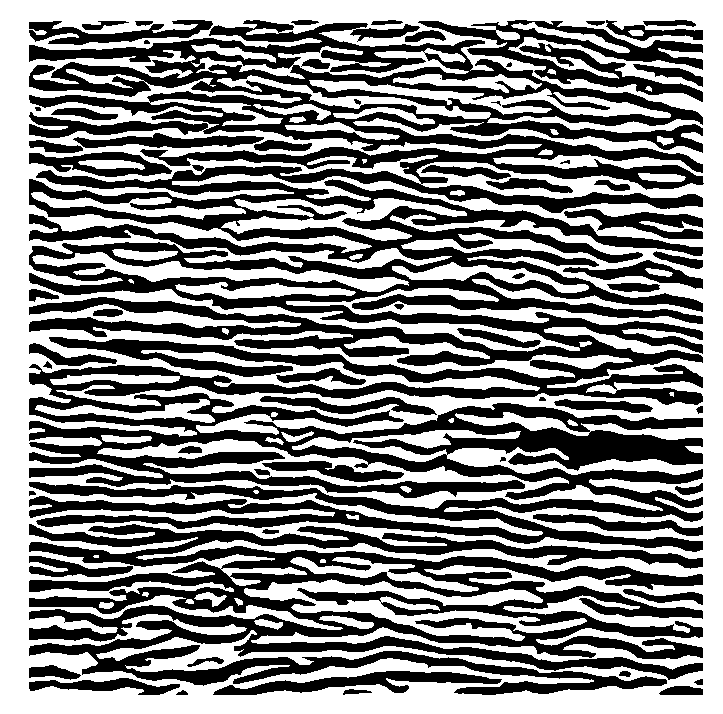

Supplement: Supplemental Information 3 [file peerj-07-7813-s003.zip › Supplemental-3/E-09.bmp]

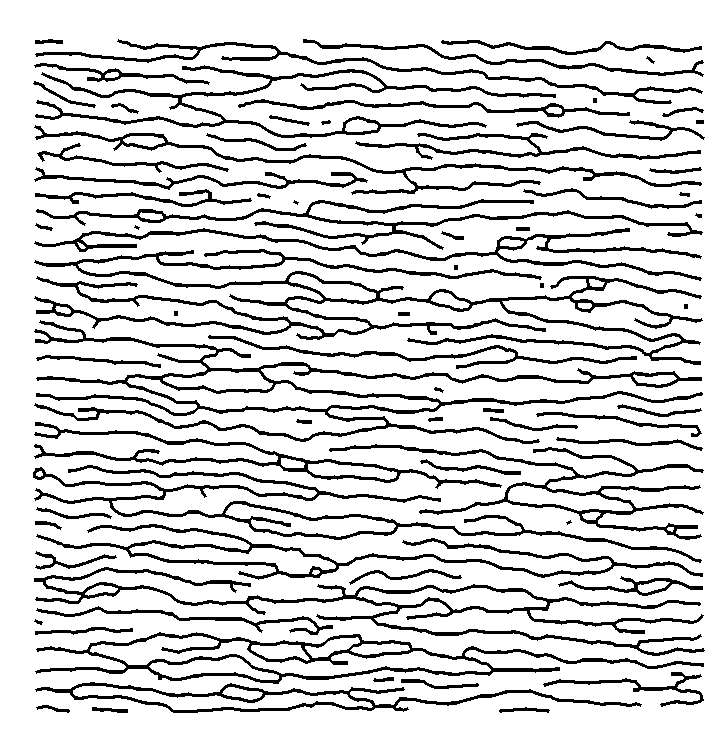

Supplement: Supplemental Information 3 [file peerj-07-7813-s003.zip › Supplemental-3/E-10-1.bmp]

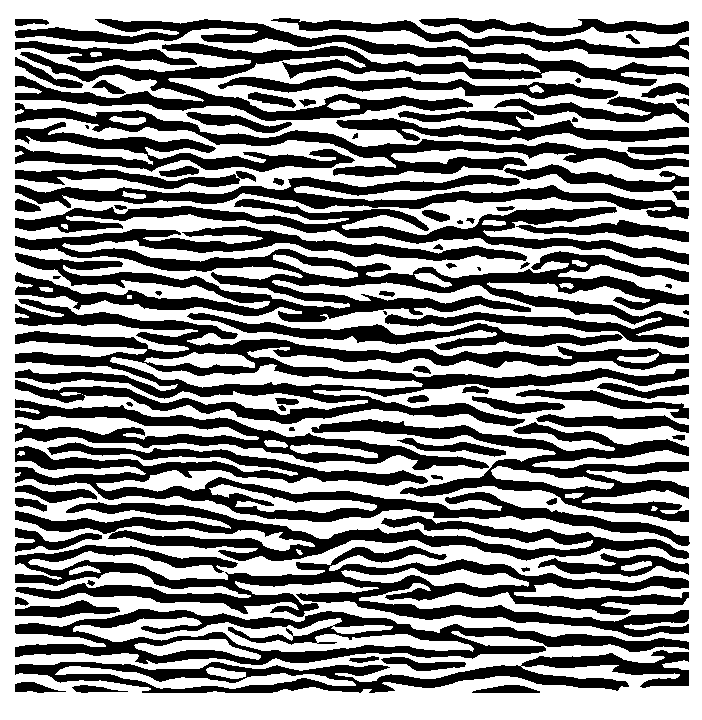

Supplement: Supplemental Information 3 [file peerj-07-7813-s003.zip › Supplemental-3/E-10.bmp]

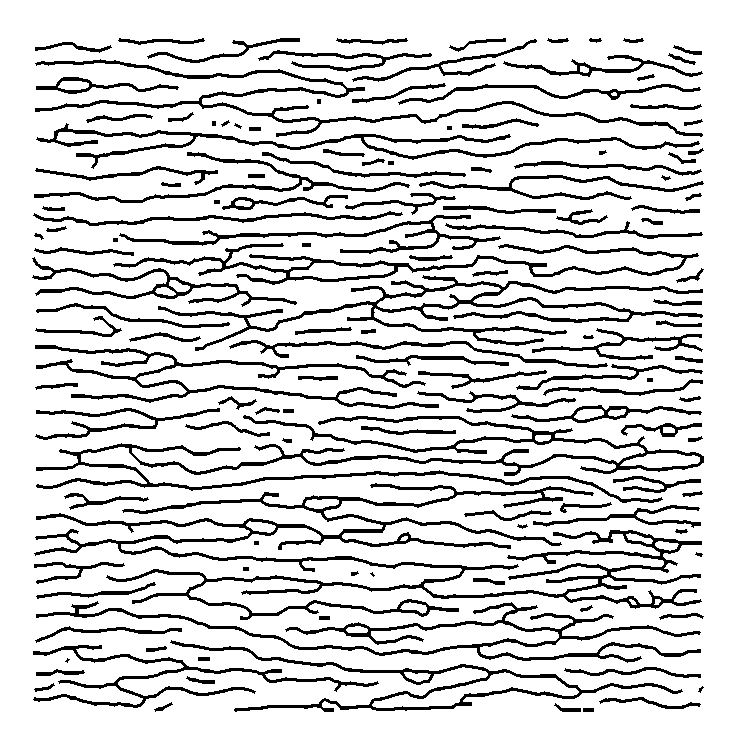

Supplement: Supplemental Information 3 [file peerj-07-7813-s003.zip › Supplemental-3/E-11-1.bmp]

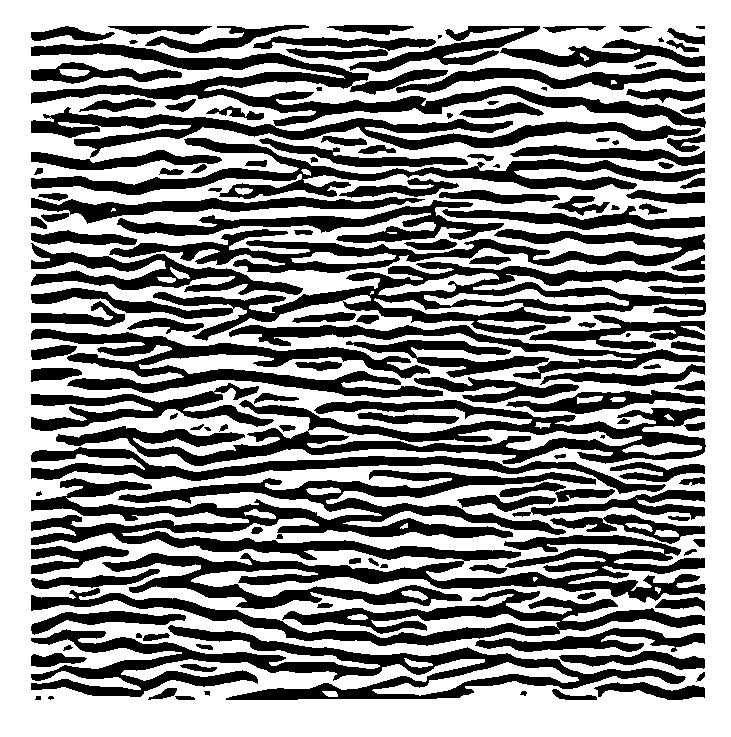

Supplement: Supplemental Information 3 [file peerj-07-7813-s003.zip › Supplemental-3/E-11.bmp]

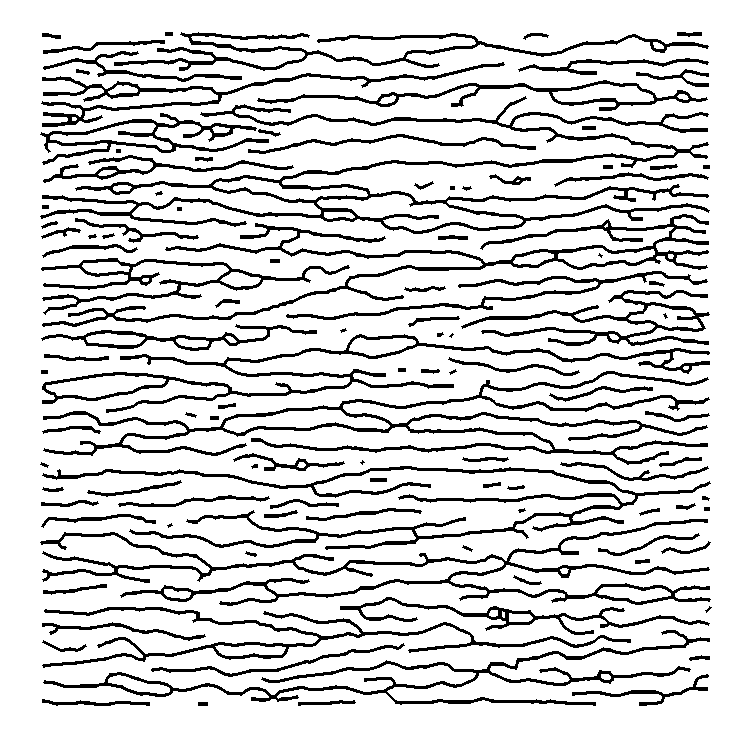

Supplement: Supplemental Information 3 [file peerj-07-7813-s003.zip › Supplemental-3/E-12-1.bmp]

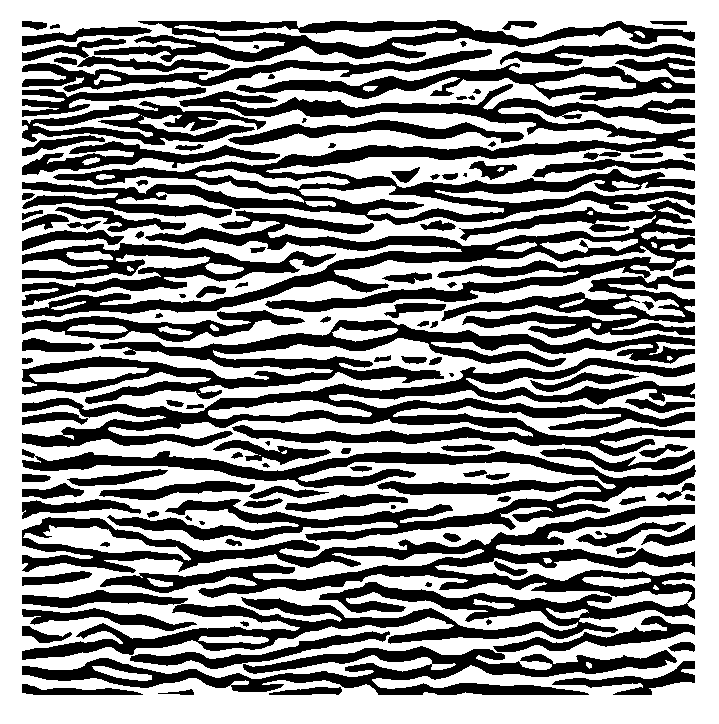

Supplement: Supplemental Information 3 [file peerj-07-7813-s003.zip › Supplemental-3/E-12.bmp]

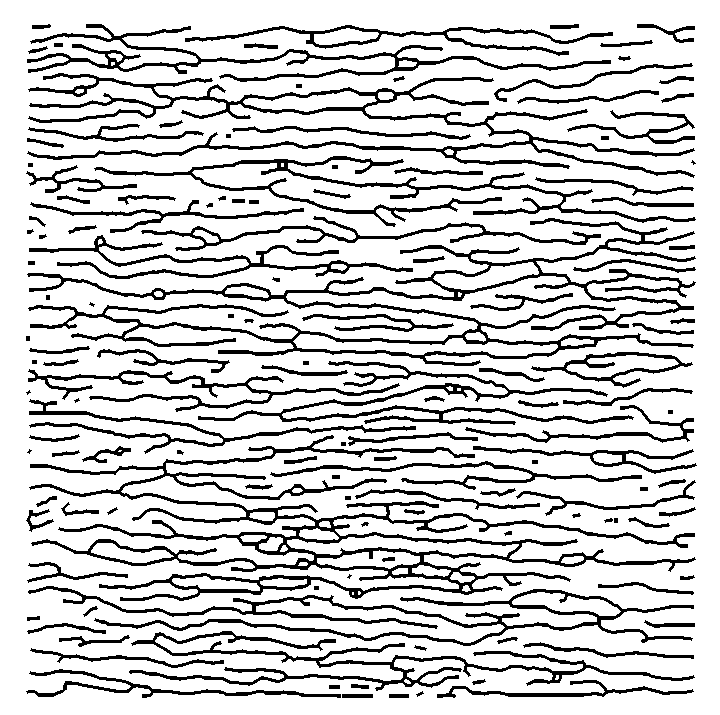

Supplement: Supplemental Information 3 [file peerj-07-7813-s003.zip › Supplemental-3/E-13-1.bmp]

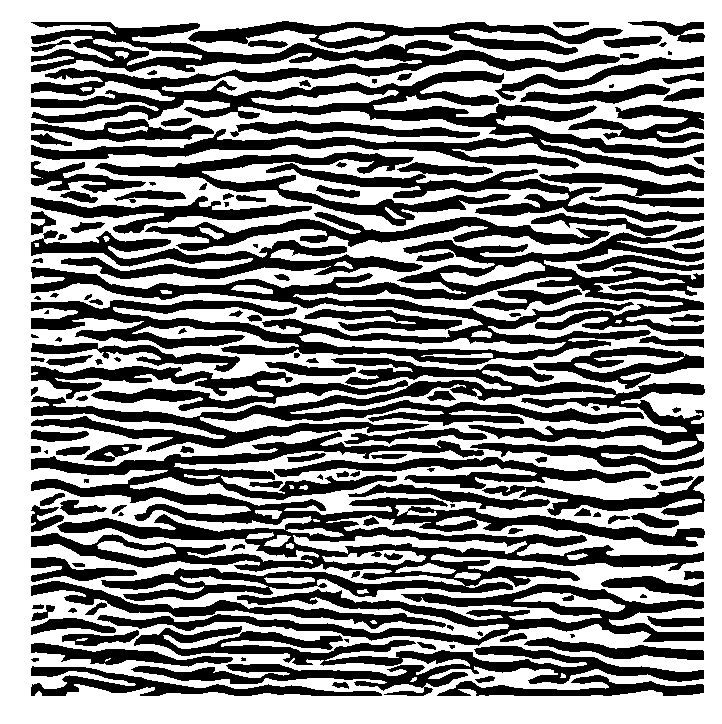

Supplement: Supplemental Information 3 [file peerj-07-7813-s003.zip › Supplemental-3/E-13.bmp]

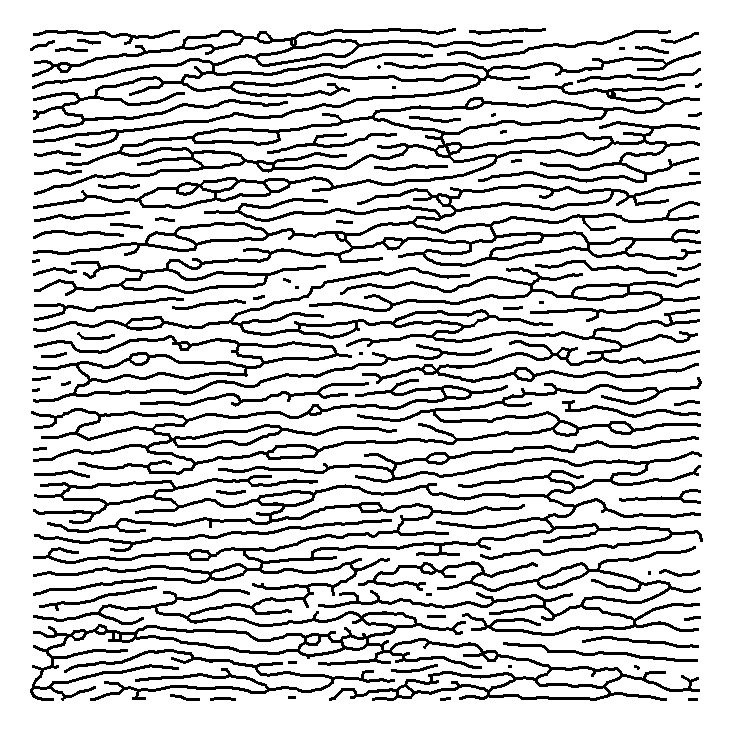

Supplement: Supplemental Information 3 [file peerj-07-7813-s003.zip › Supplemental-3/F-04-1.bmp]

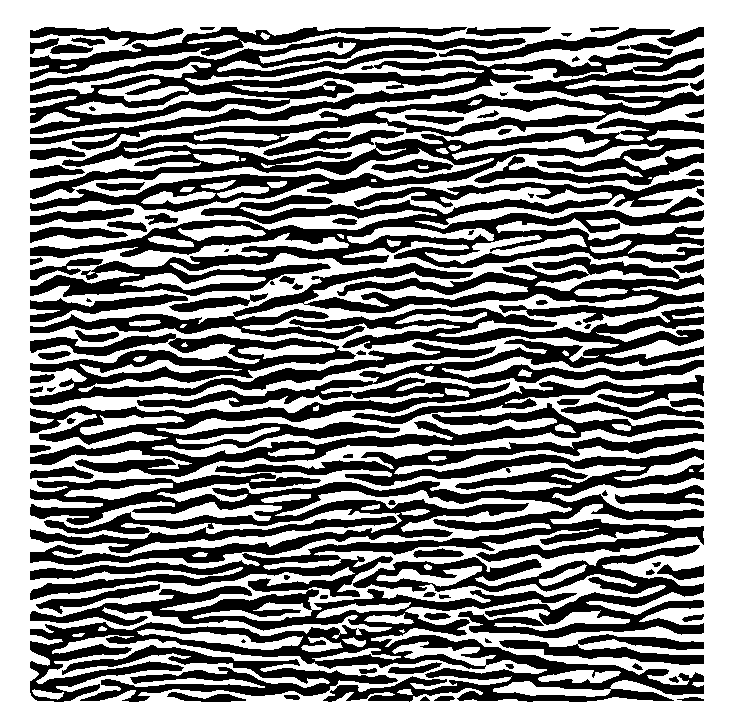

Supplement: Supplemental Information 3 [file peerj-07-7813-s003.zip › Supplemental-3/F-04.bmp]

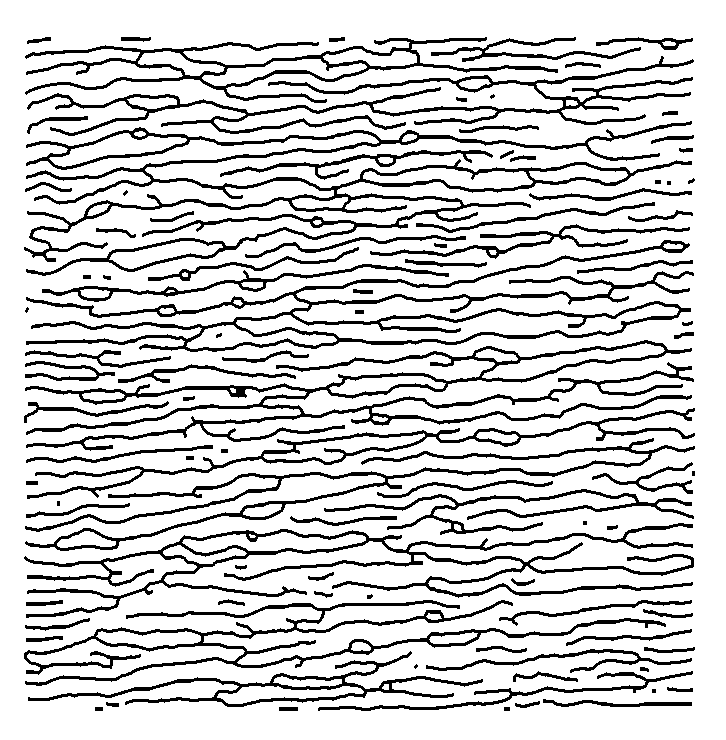

Supplement: Supplemental Information 3 [file peerj-07-7813-s003.zip › Supplemental-3/F-05-1.bmp]

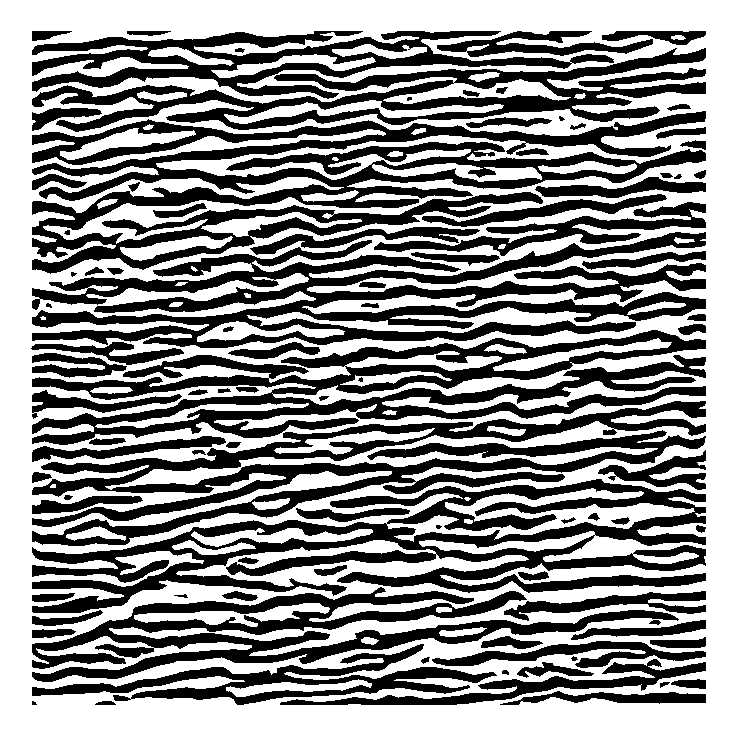

Supplement: Supplemental Information 3 [file peerj-07-7813-s003.zip › Supplemental-3/F-05.bmp]

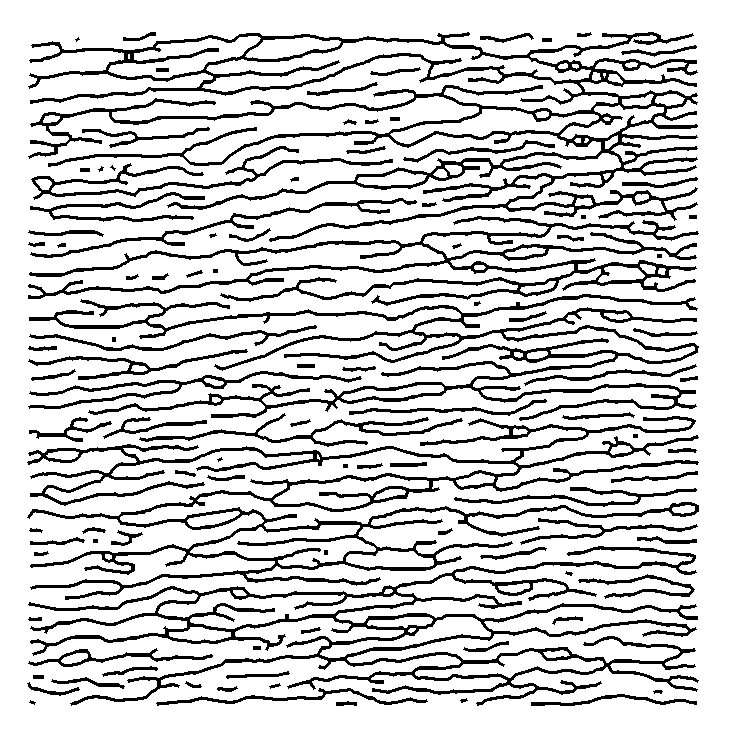

Supplement: Supplemental Information 3 [file peerj-07-7813-s003.zip › Supplemental-3/F-06-1.bmp]

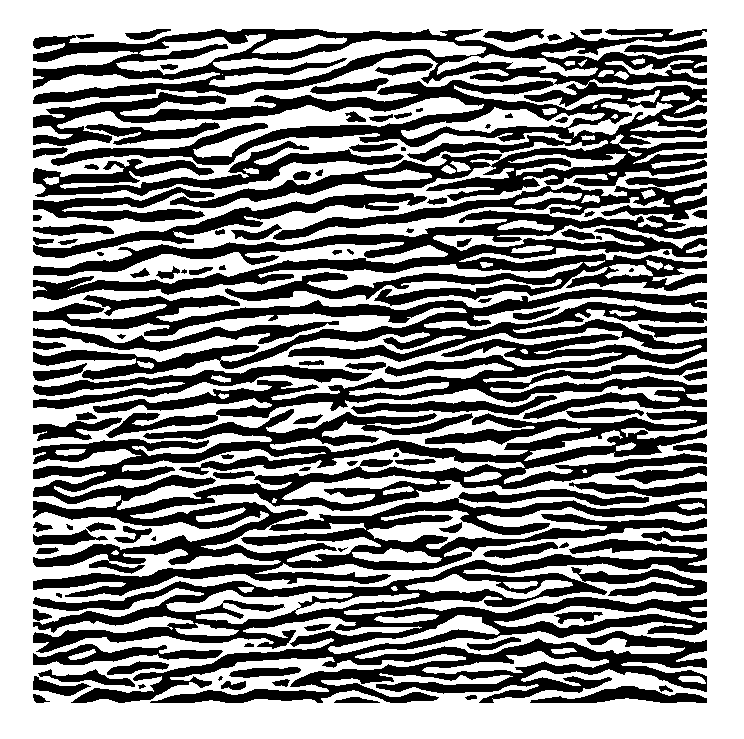

Supplement: Supplemental Information 3 [file peerj-07-7813-s003.zip › Supplemental-3/F-06.bmp]

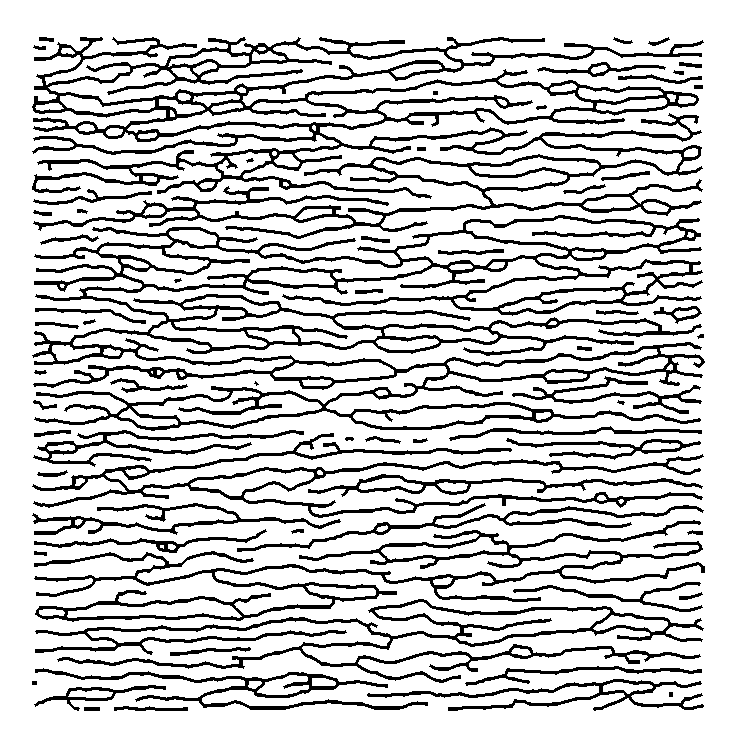

Supplement: Supplemental Information 3 [file peerj-07-7813-s003.zip › Supplemental-3/F-07-1.bmp]

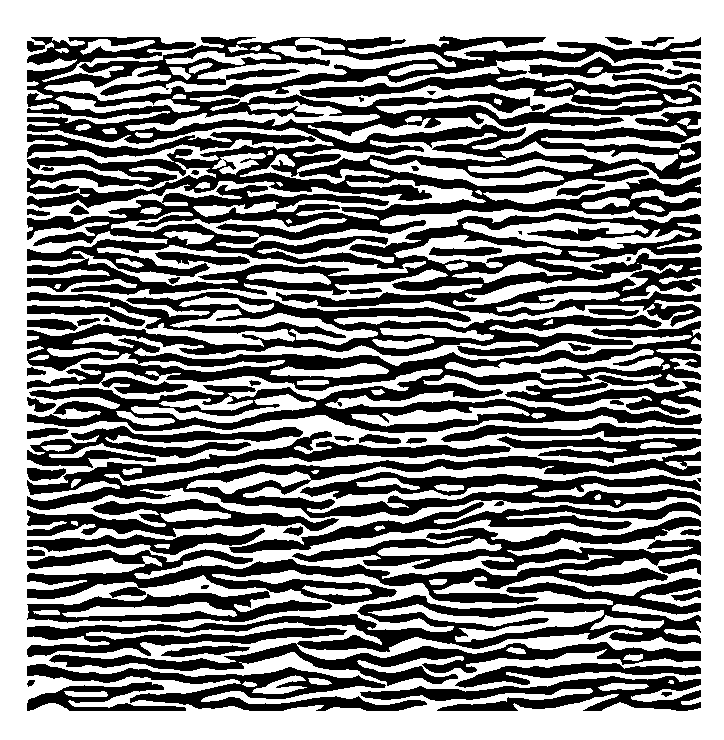

Supplement: Supplemental Information 3 [file peerj-07-7813-s003.zip › Supplemental-3/F-07.bmp]

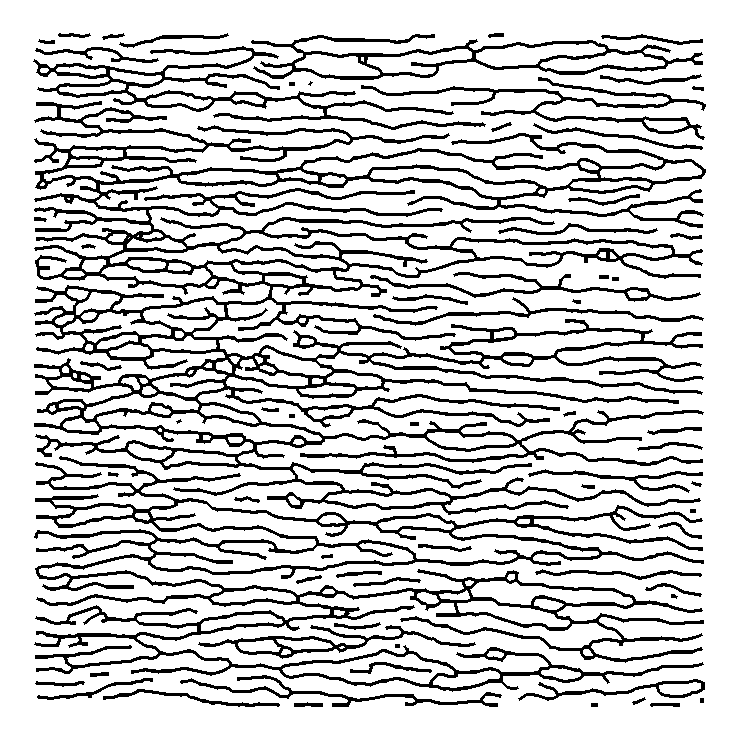

Supplement: Supplemental Information 3 [file peerj-07-7813-s003.zip › Supplemental-3/F-08-1.bmp]

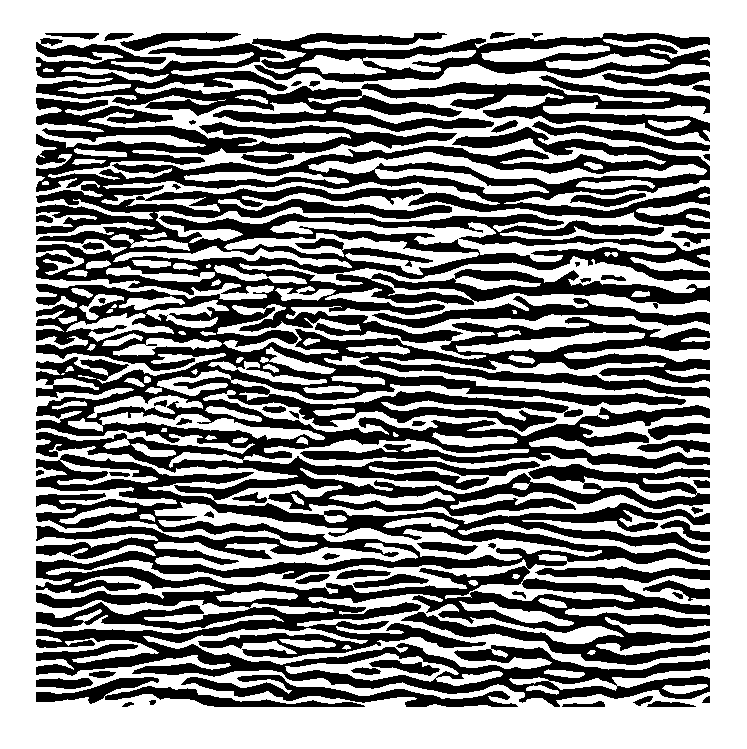

Supplement: Supplemental Information 3 [file peerj-07-7813-s003.zip › Supplemental-3/F-08.bmp]

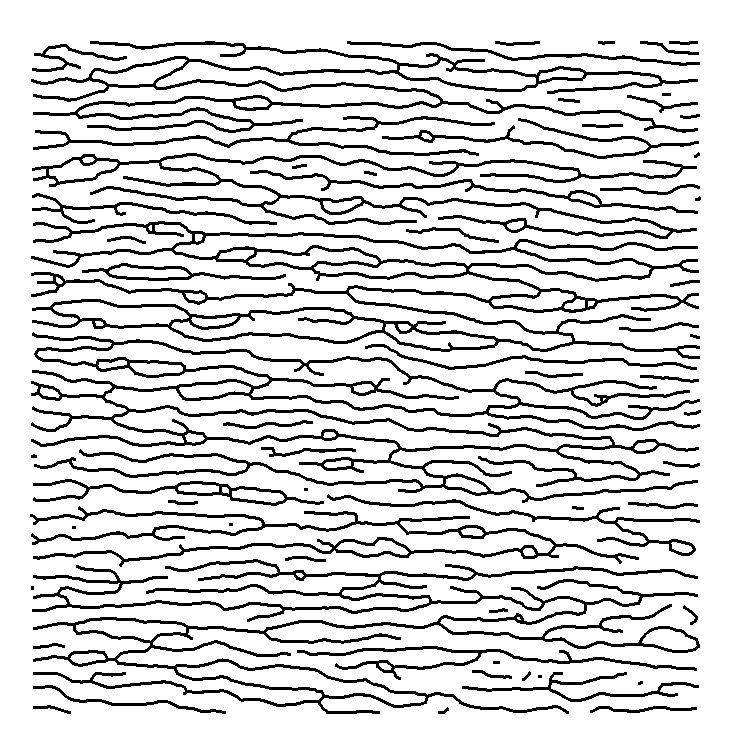

Supplement: Supplemental Information 3 [file peerj-07-7813-s003.zip › Supplemental-3/F-09-1.bmp]

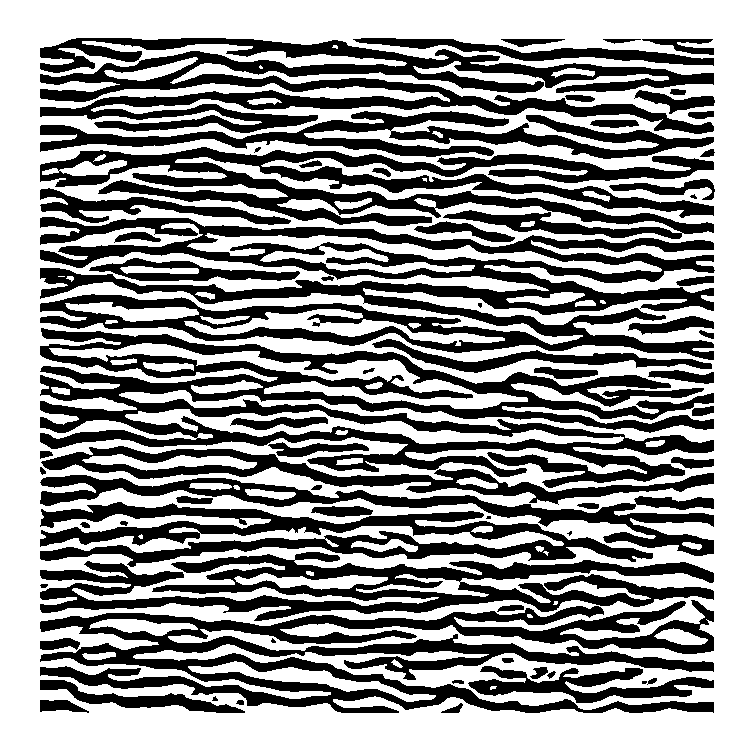

Supplement: Supplemental Information 3 [file peerj-07-7813-s003.zip › Supplemental-3/F-09.bmp]

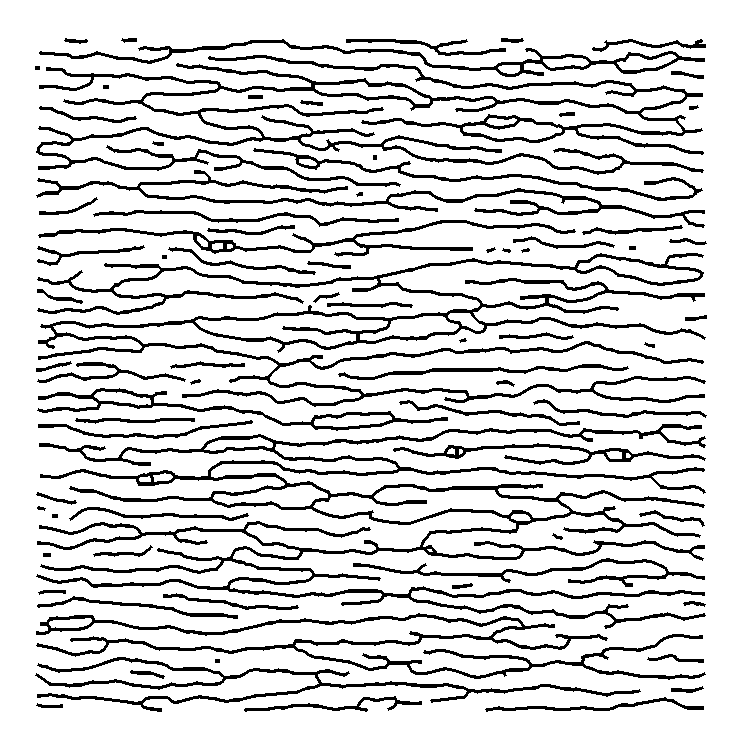

Supplement: Supplemental Information 3 [file peerj-07-7813-s003.zip › Supplemental-3/F-10-1.bmp]

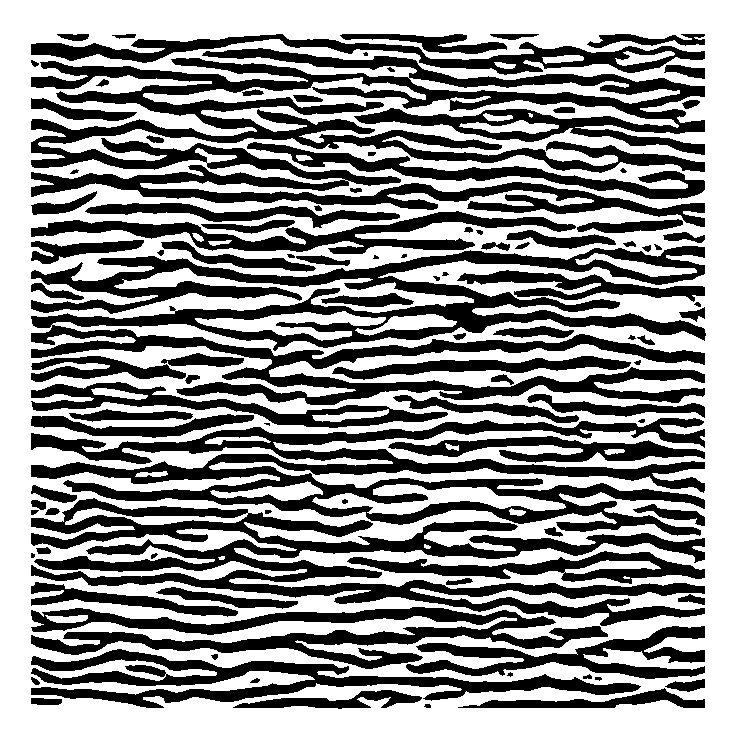

Supplement: Supplemental Information 3 [file peerj-07-7813-s003.zip › Supplemental-3/F-10.bmp]

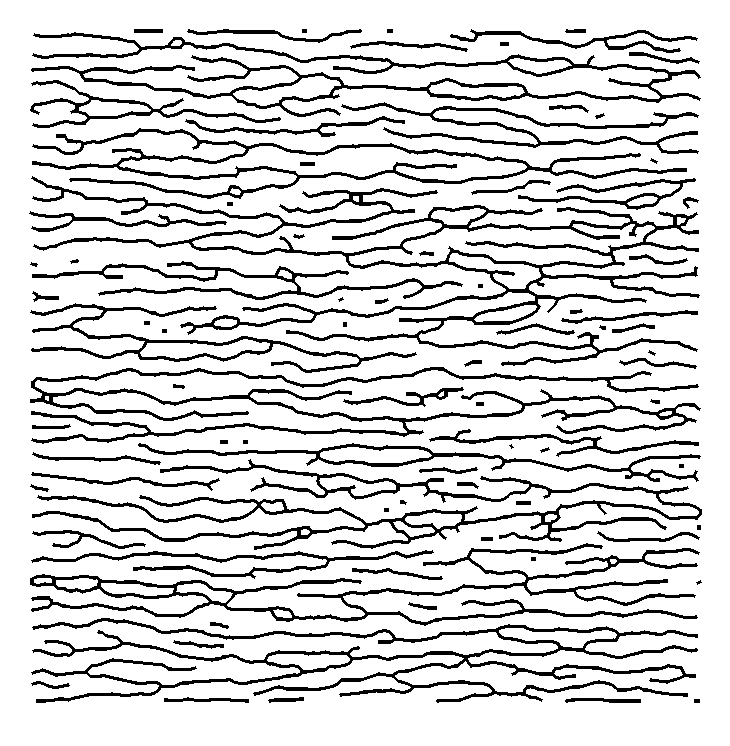

Supplement: Supplemental Information 3 [file peerj-07-7813-s003.zip › Supplemental-3/F-11-1.bmp]

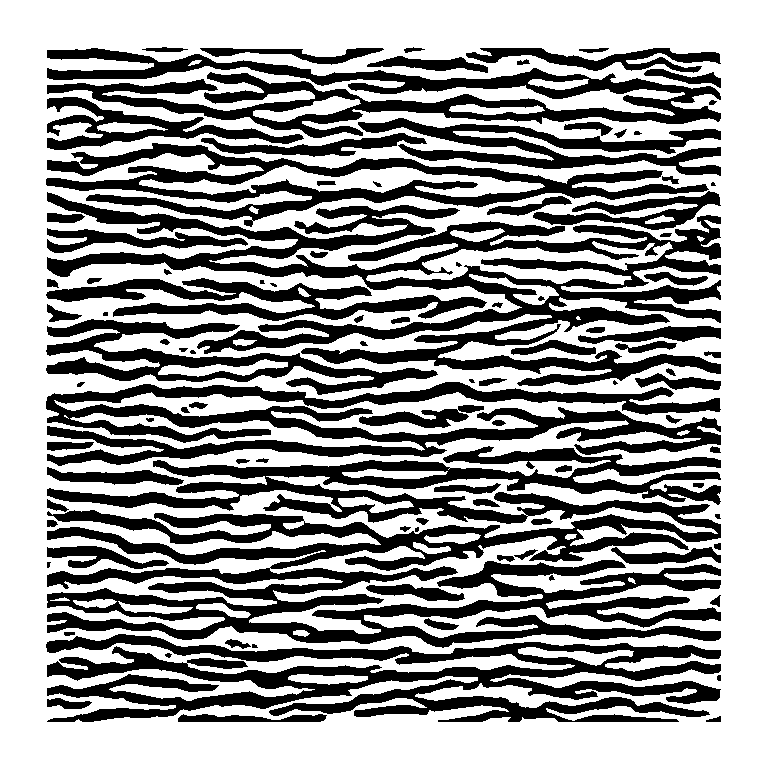

Supplement: Supplemental Information 3 [file peerj-07-7813-s003.zip › Supplemental-3/F-11.bmp]

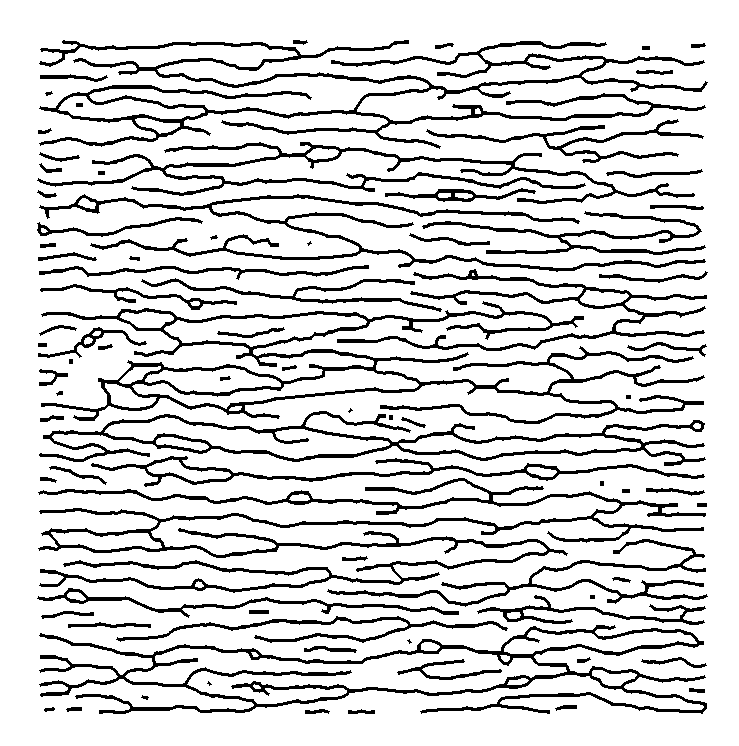

Supplement: Supplemental Information 3 [file peerj-07-7813-s003.zip › Supplemental-3/F-12-1.bmp]

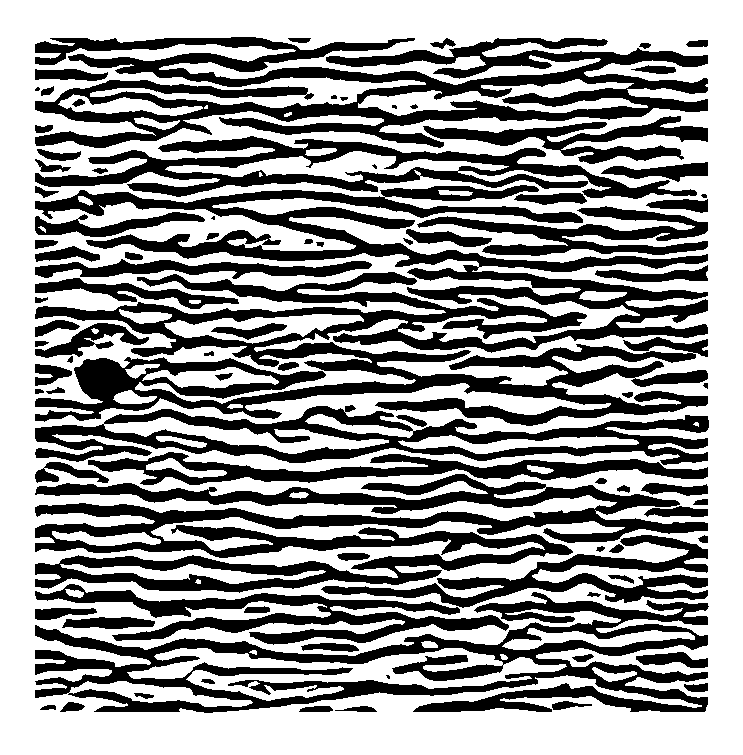

Supplement: Supplemental Information 3 [file peerj-07-7813-s003.zip › Supplemental-3/F-12.bmp]

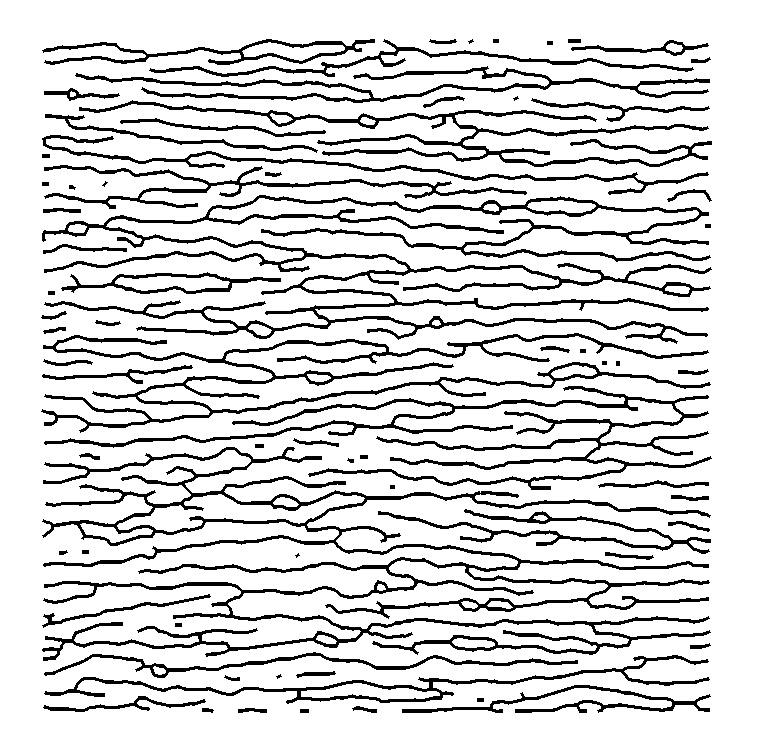

Supplement: Supplemental Information 3 [file peerj-07-7813-s003.zip › Supplemental-3/F-13-1.bmp]

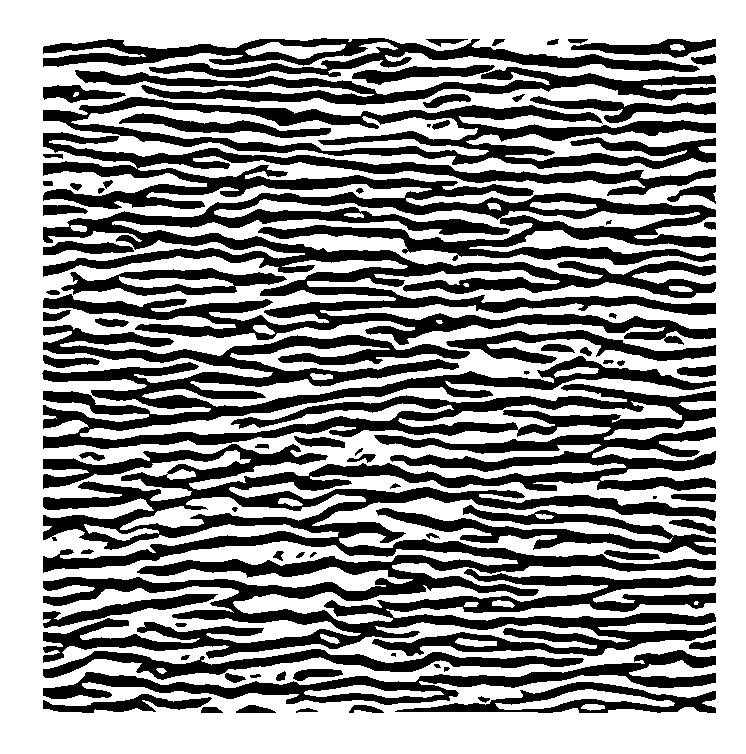

Supplement: Supplemental Information 3 [file peerj-07-7813-s003.zip › Supplemental-3/F-13.bmp]

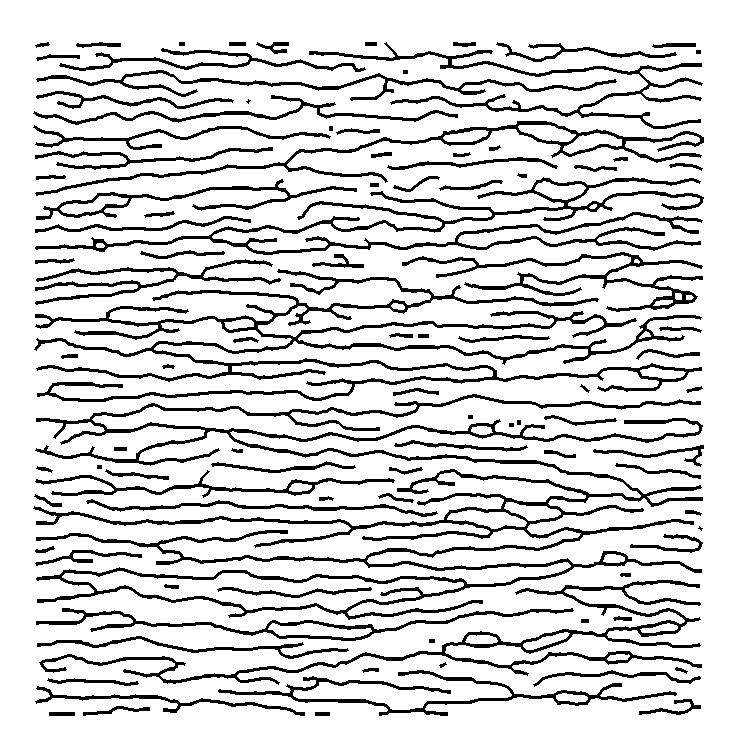

Supplement: Supplemental Information 3 [file peerj-07-7813-s003.zip › Supplemental-3/F-14-1.bmp]

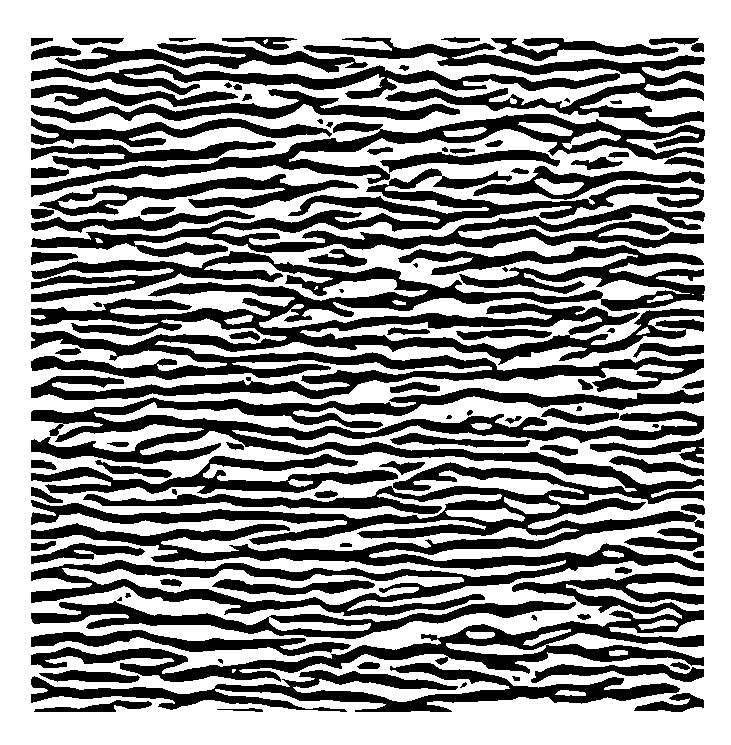

Supplement: Supplemental Information 3 [file peerj-07-7813-s003.zip › Supplemental-3/F-14.bmp]

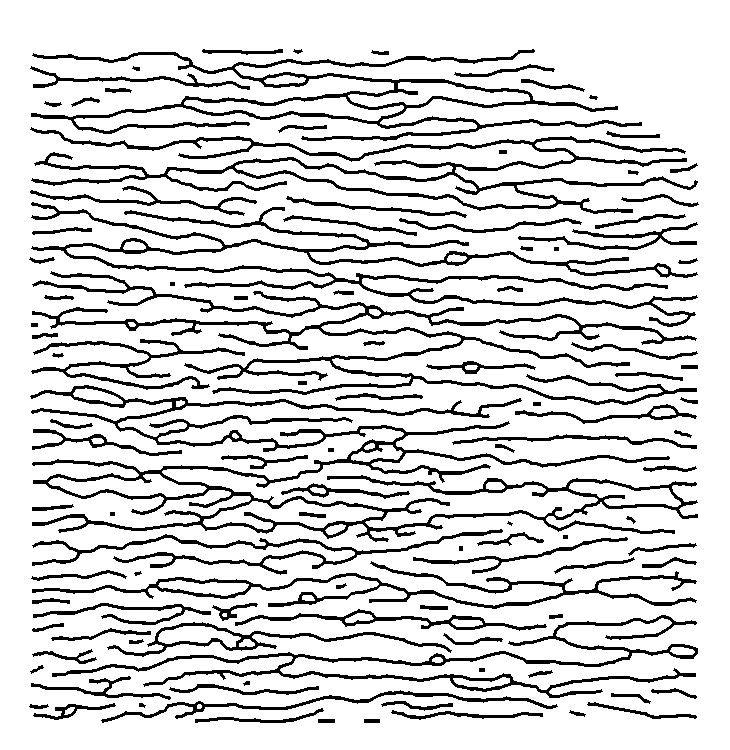

Supplement: Supplemental Information 3 [file peerj-07-7813-s003.zip › Supplemental-3/F-15-1.bmp]

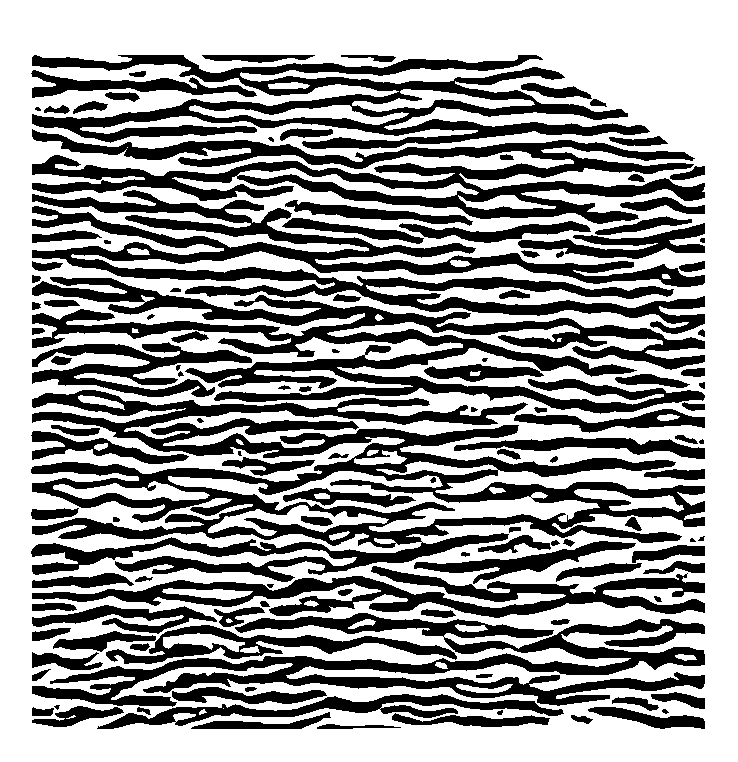

Supplement: Supplemental Information 3 [file peerj-07-7813-s003.zip › Supplemental-3/F-15.bmp]

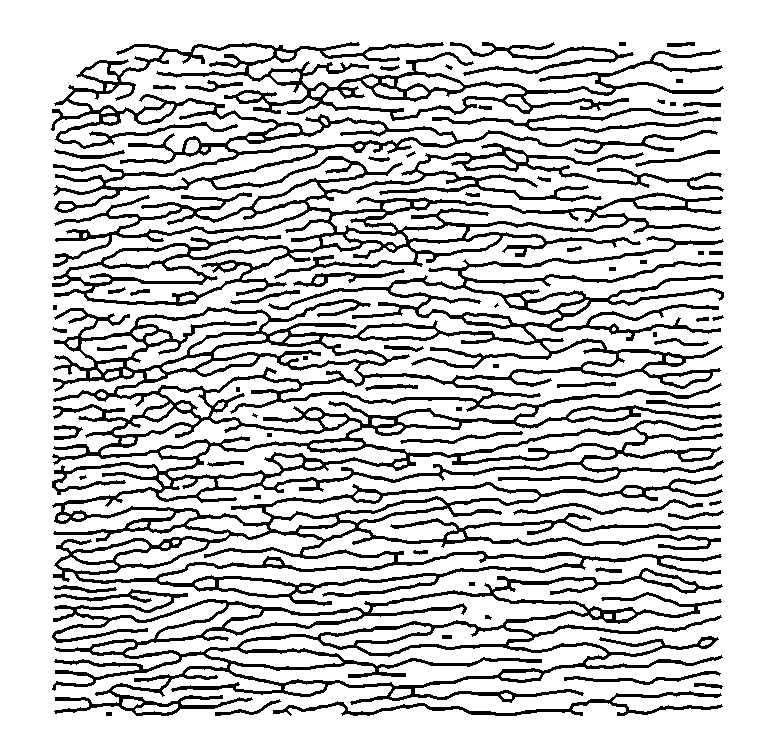

Supplement: Supplemental Information 3 [file peerj-07-7813-s003.zip › Supplemental-3/G-03-1.bmp]
