# Supplementary figures and images for: Layered patterns in nature, medicine, and materials: quantifying anisotropic structures and cyclicity (part 3 of 4)
Source: PeerJ. 2019 Oct 14;7:e7813. doi: 10.7717/peerj.7813 (PMC6797002; doi:10.7717/peerj.7813)

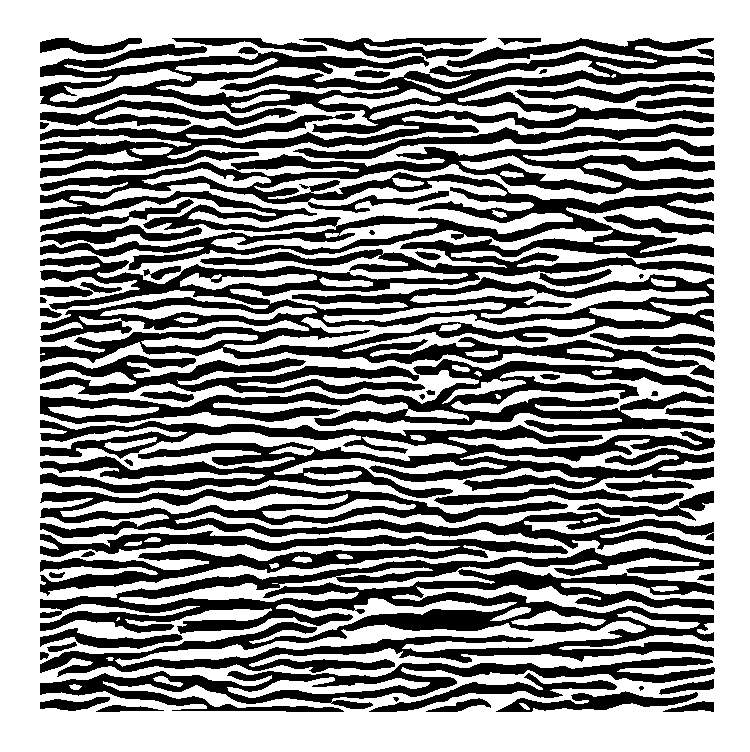

Supplement: Supplemental Information 3 [file peerj-07-7813-s003.zip › Supplemental-3/J-06.bmp]

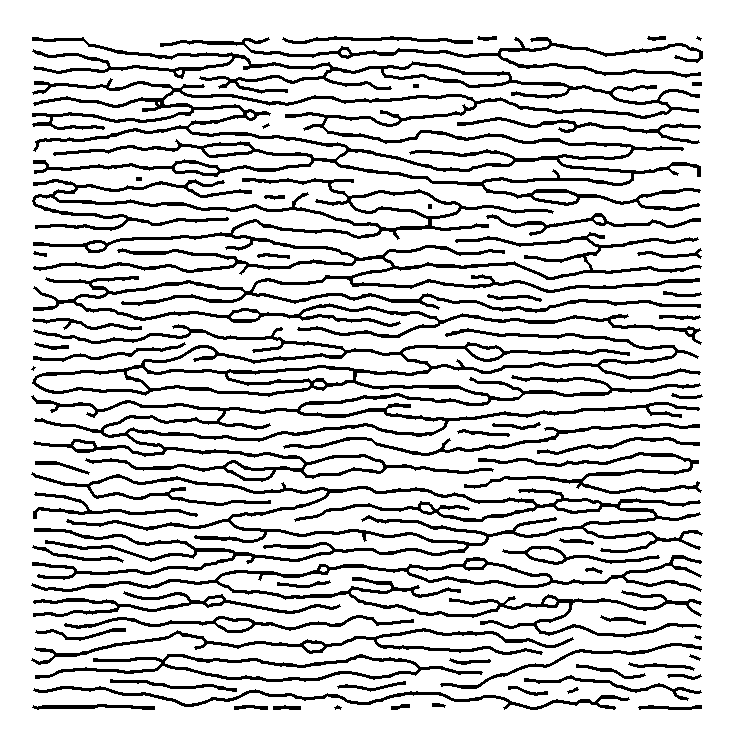

Supplement: Supplemental Information 3 [file peerj-07-7813-s003.zip › Supplemental-3/J-07-1.bmp]

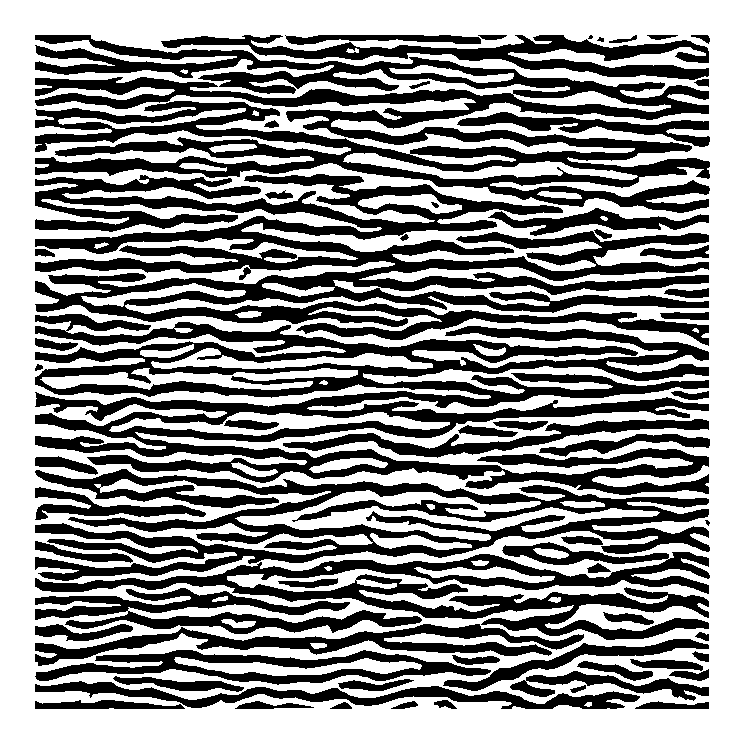

Supplement: Supplemental Information 3 [file peerj-07-7813-s003.zip › Supplemental-3/J-07.bmp]

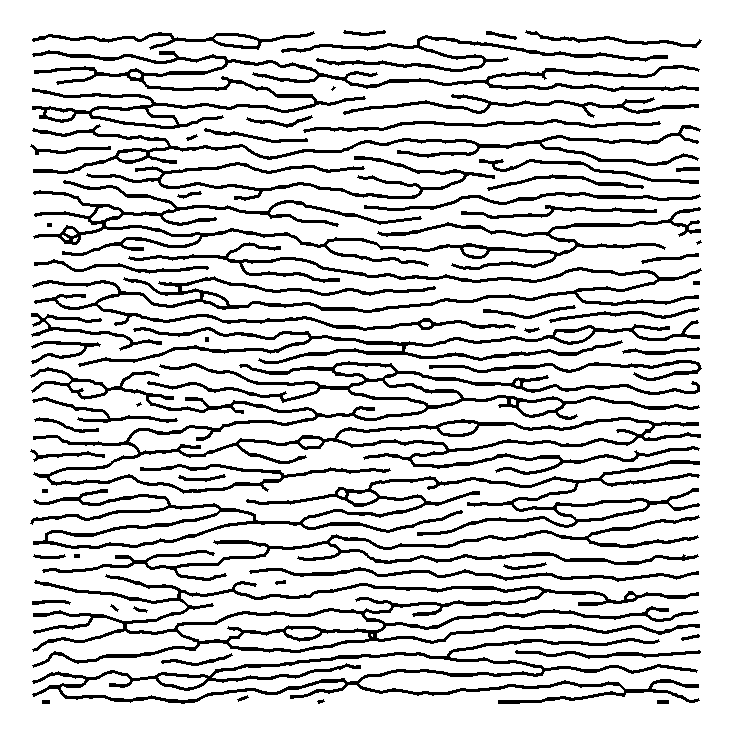

Supplement: Supplemental Information 3 [file peerj-07-7813-s003.zip › Supplemental-3/J-08-1.bmp]

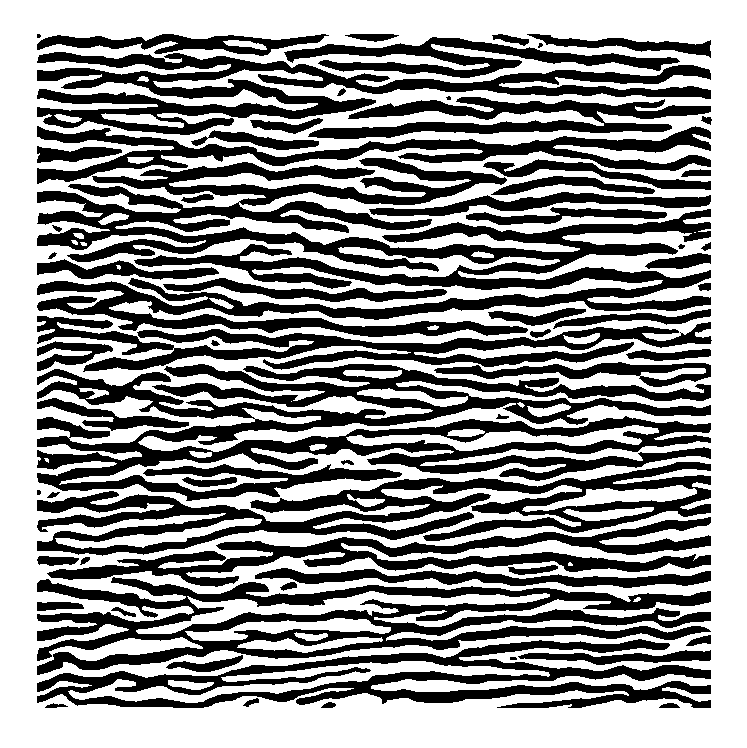

Supplement: Supplemental Information 3 [file peerj-07-7813-s003.zip › Supplemental-3/J-08.bmp]

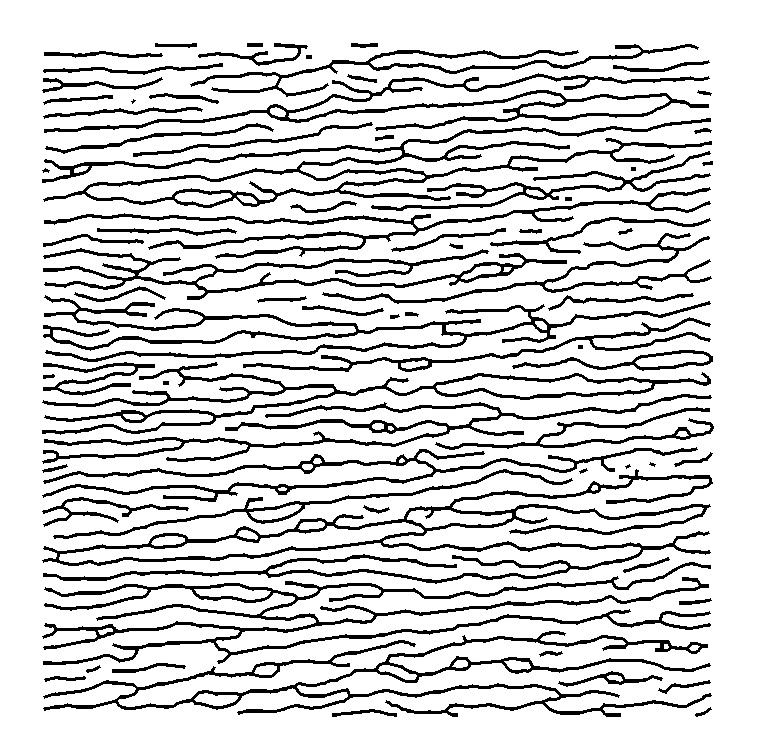

Supplement: Supplemental Information 3 [file peerj-07-7813-s003.zip › Supplemental-3/J-09-1.bmp]

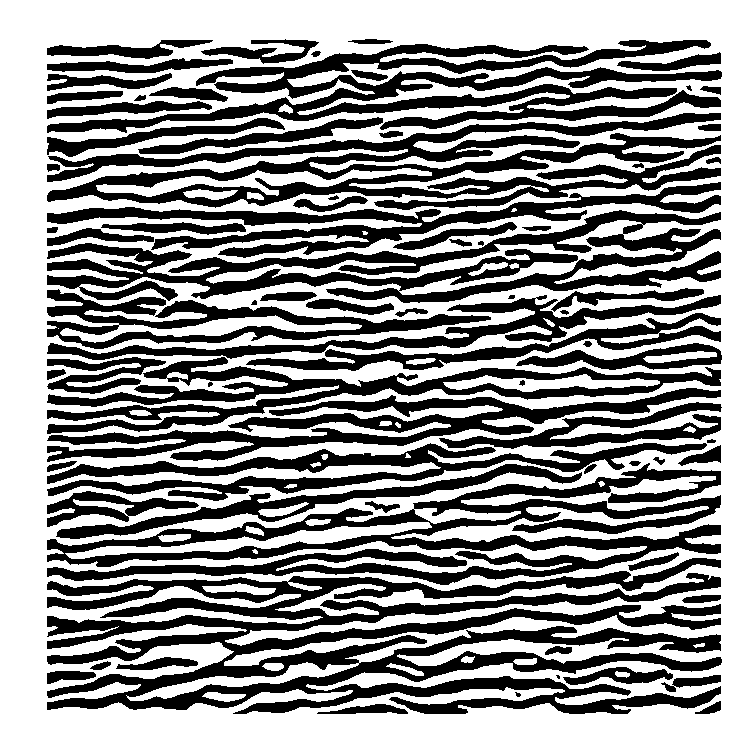

Supplement: Supplemental Information 3 [file peerj-07-7813-s003.zip › Supplemental-3/J-09.bmp]

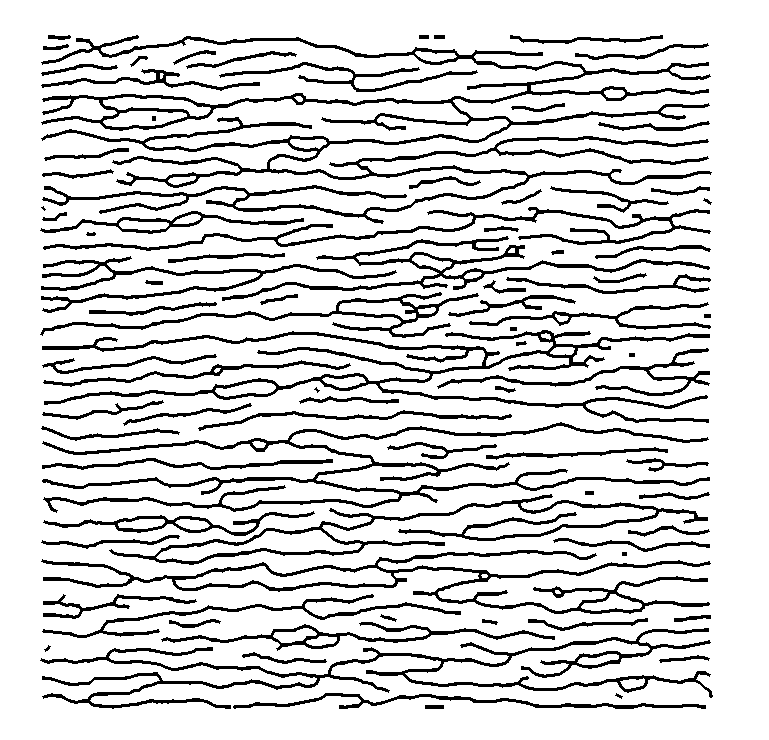

Supplement: Supplemental Information 3 [file peerj-07-7813-s003.zip › Supplemental-3/J-10-1.bmp]

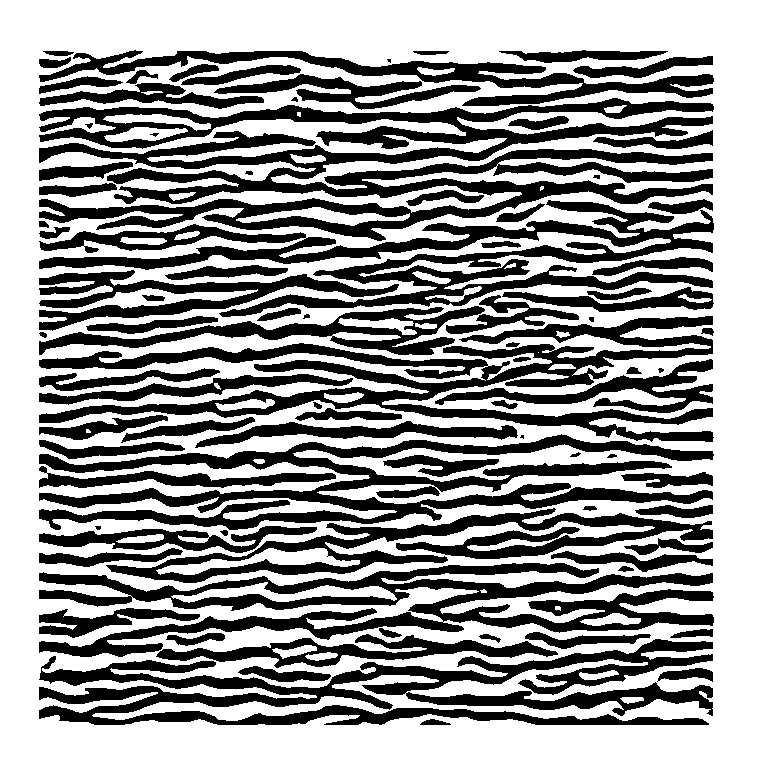

Supplement: Supplemental Information 3 [file peerj-07-7813-s003.zip › Supplemental-3/J-10.bmp]

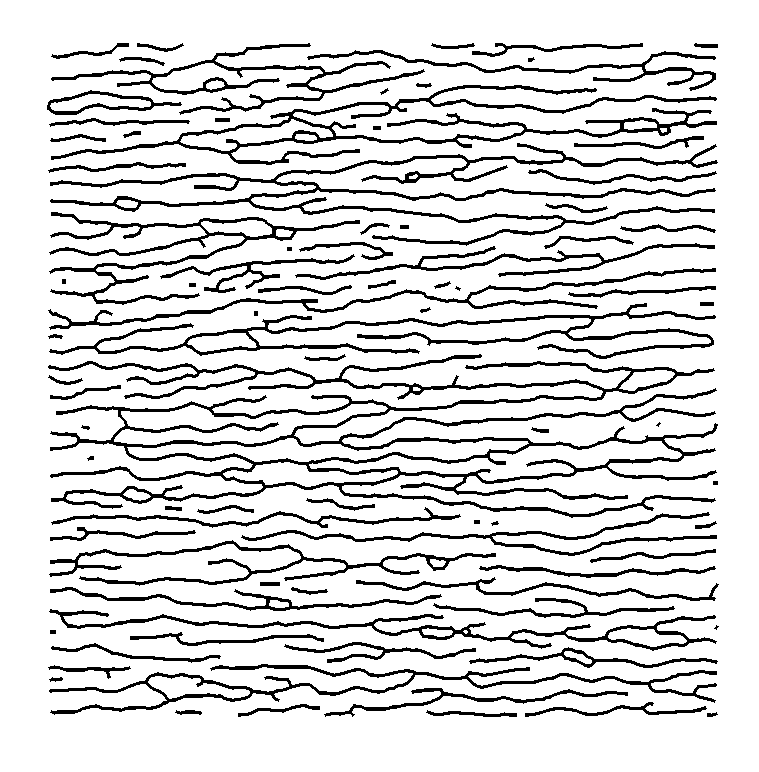

Supplement: Supplemental Information 3 [file peerj-07-7813-s003.zip › Supplemental-3/J-11-1.bmp]

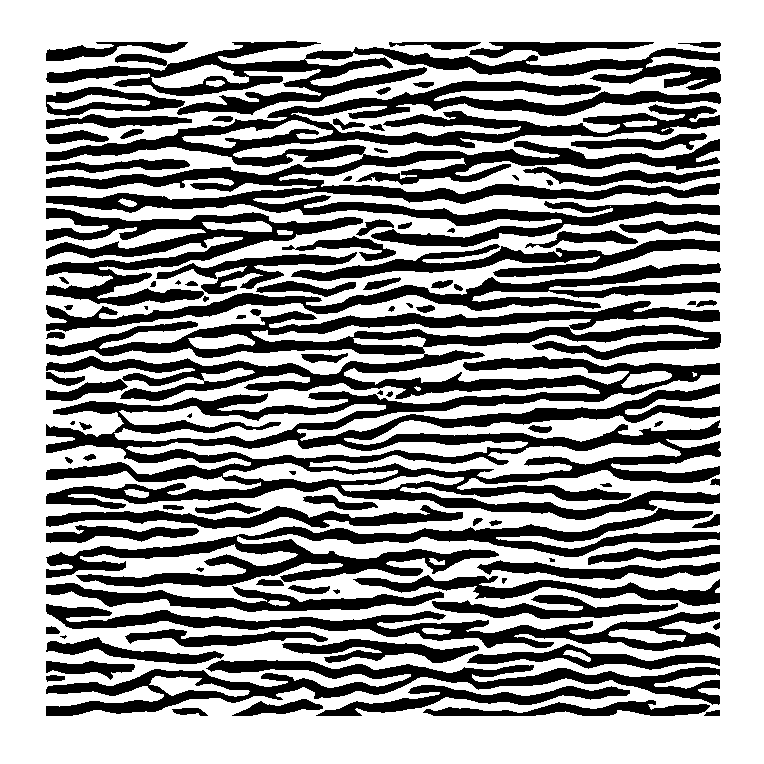

Supplement: Supplemental Information 3 [file peerj-07-7813-s003.zip › Supplemental-3/J-11.bmp]

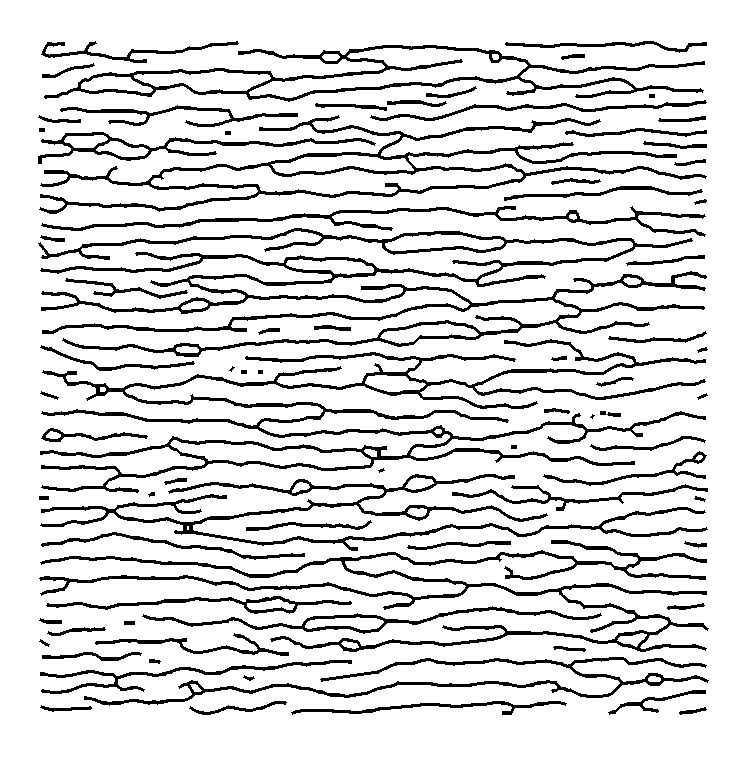

Supplement: Supplemental Information 3 [file peerj-07-7813-s003.zip › Supplemental-3/J-12-1.bmp]

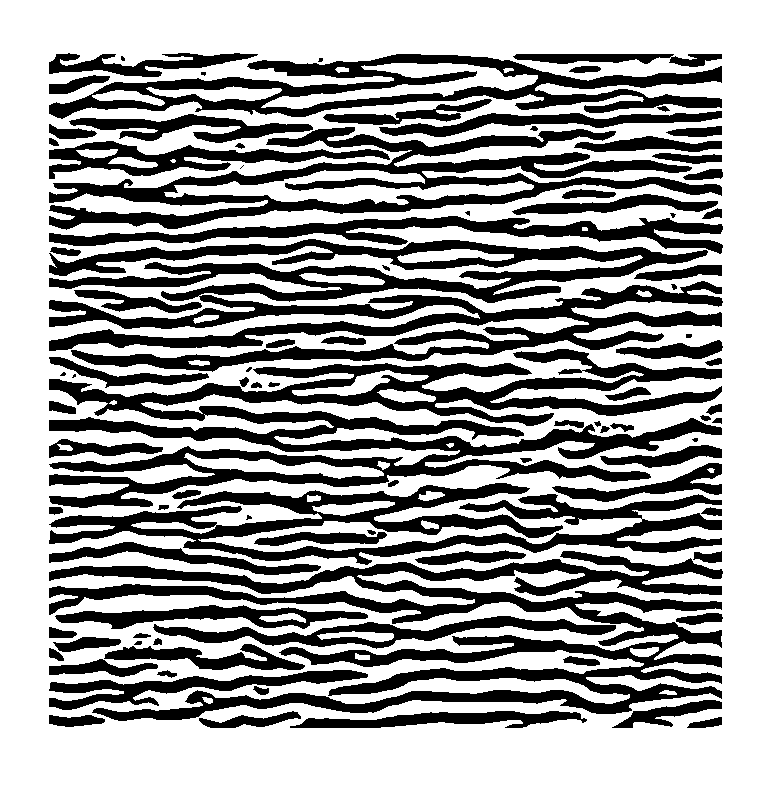

Supplement: Supplemental Information 3 [file peerj-07-7813-s003.zip › Supplemental-3/J-12.bmp]

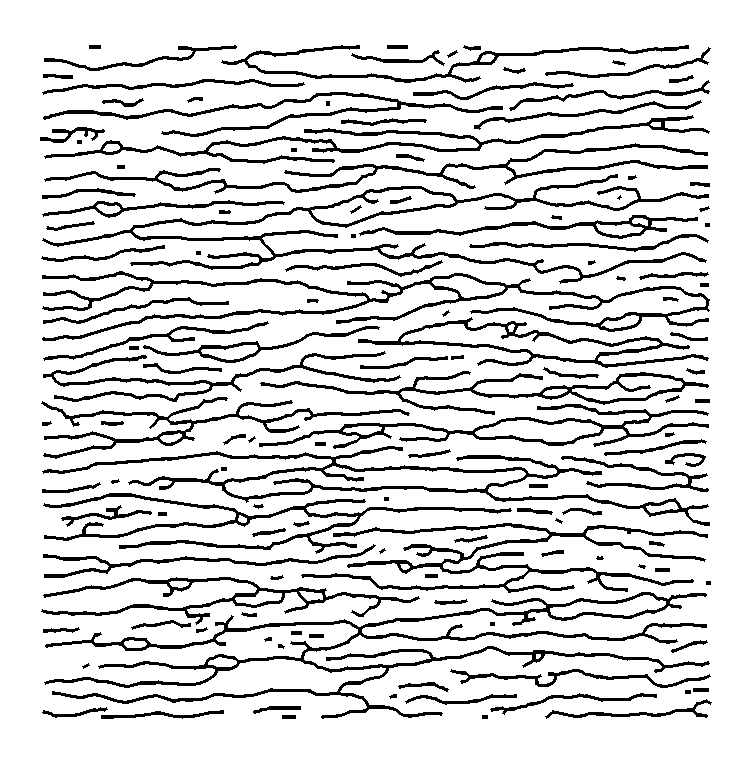

Supplement: Supplemental Information 3 [file peerj-07-7813-s003.zip › Supplemental-3/J-13-1.bmp]

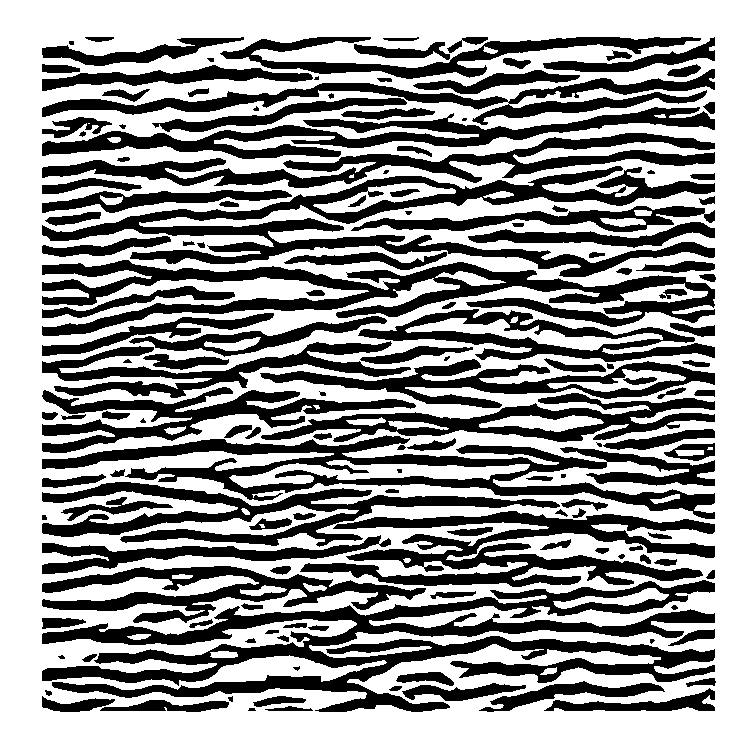

Supplement: Supplemental Information 3 [file peerj-07-7813-s003.zip › Supplemental-3/J-13.bmp]

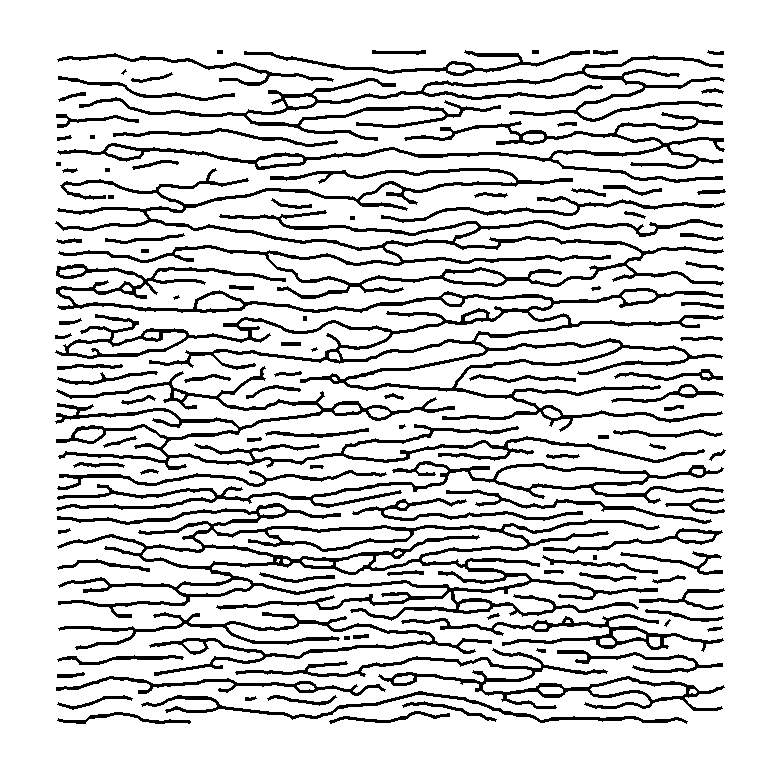

Supplement: Supplemental Information 3 [file peerj-07-7813-s003.zip › Supplemental-3/J-14-1.bmp]

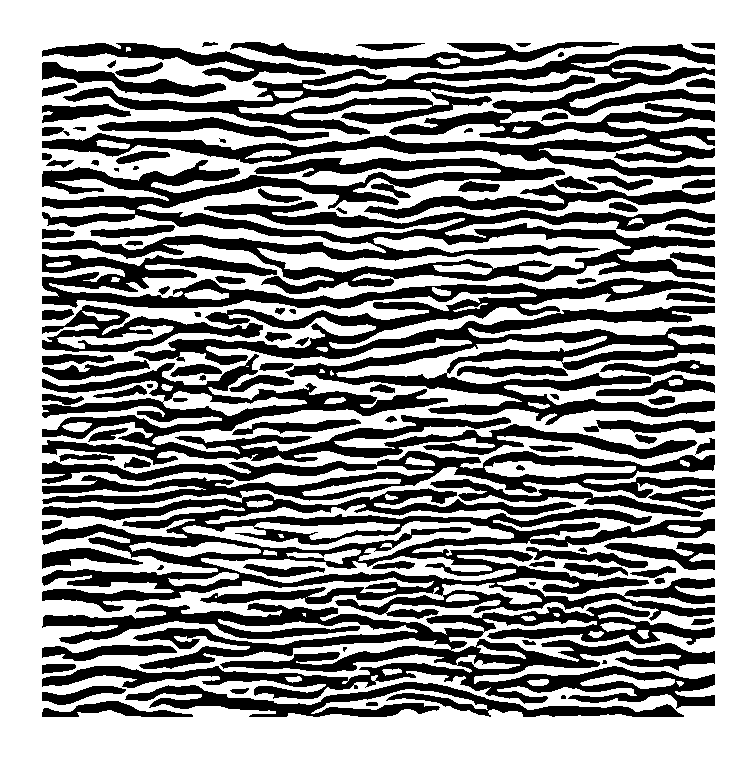

Supplement: Supplemental Information 3 [file peerj-07-7813-s003.zip › Supplemental-3/J-14.bmp]

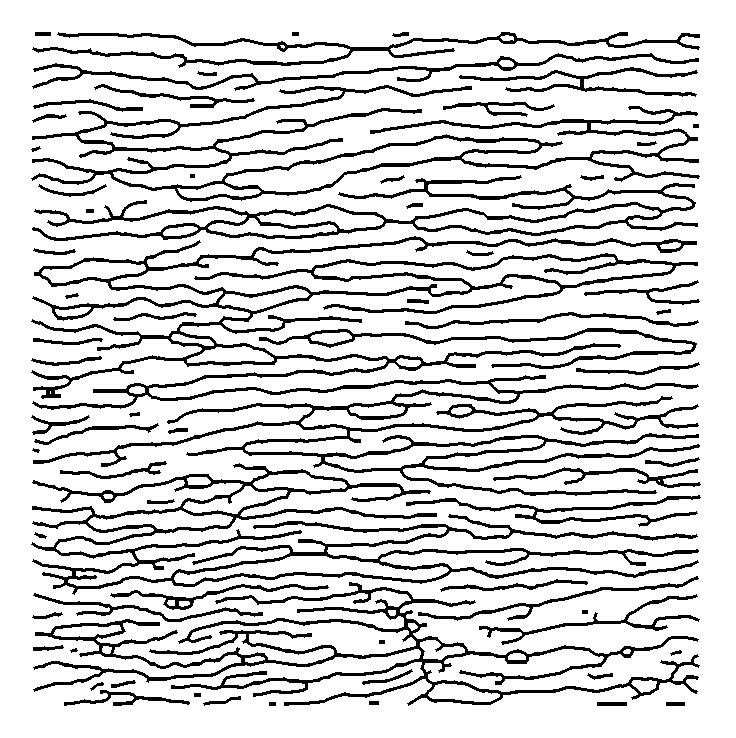

Supplement: Supplemental Information 3 [file peerj-07-7813-s003.zip › Supplemental-3/J-15-1.bmp]

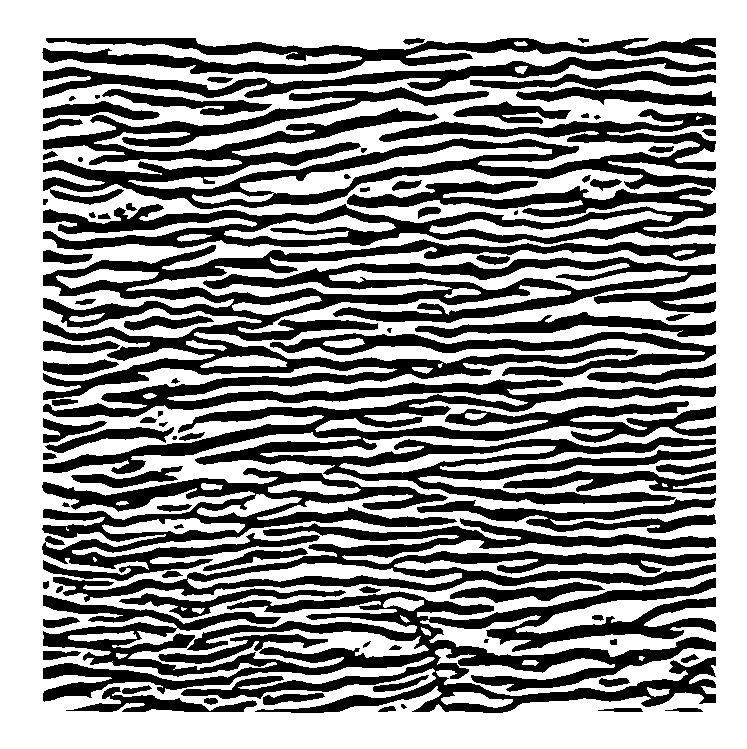

Supplement: Supplemental Information 3 [file peerj-07-7813-s003.zip › Supplemental-3/J-15.bmp]

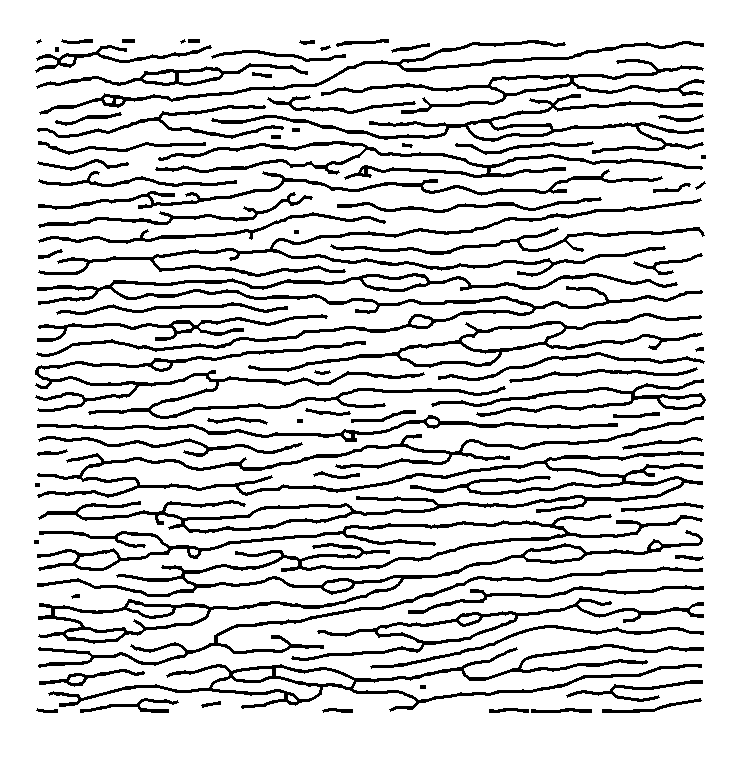

Supplement: Supplemental Information 3 [file peerj-07-7813-s003.zip › Supplemental-3/J-16-1.bmp]

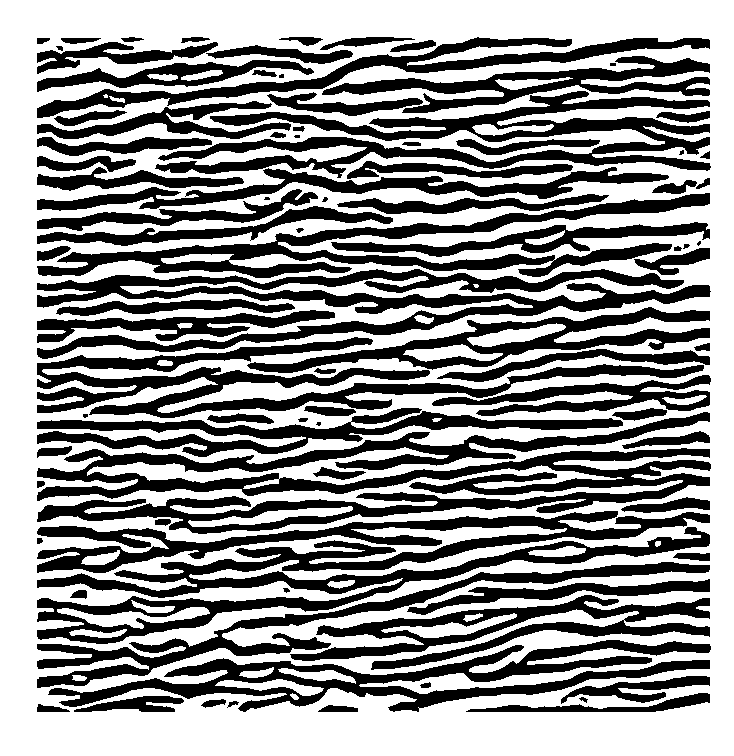

Supplement: Supplemental Information 3 [file peerj-07-7813-s003.zip › Supplemental-3/J-16.bmp]

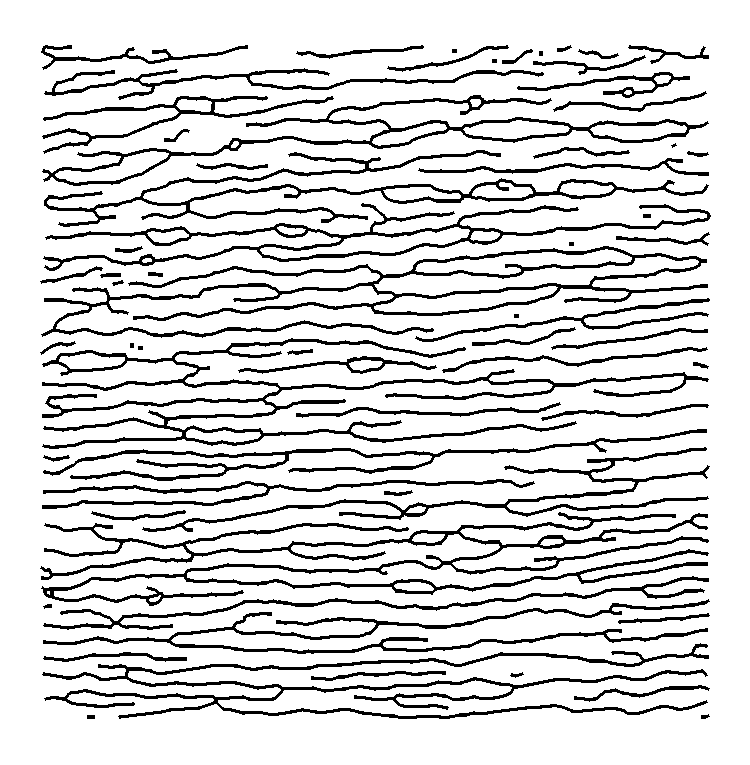

Supplement: Supplemental Information 3 [file peerj-07-7813-s003.zip › Supplemental-3/J-17-1.bmp]

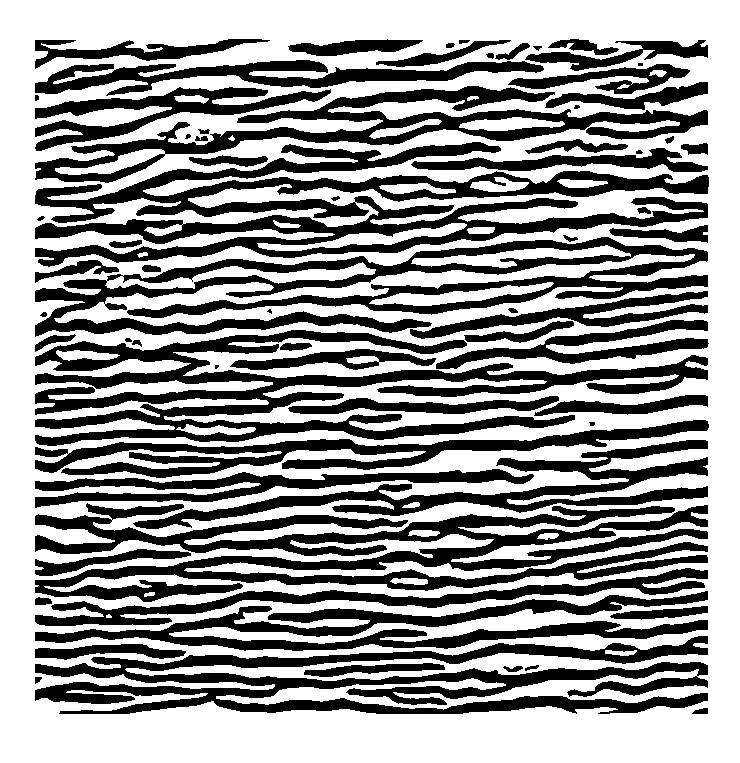

Supplement: Supplemental Information 3 [file peerj-07-7813-s003.zip › Supplemental-3/J-17.bmp]

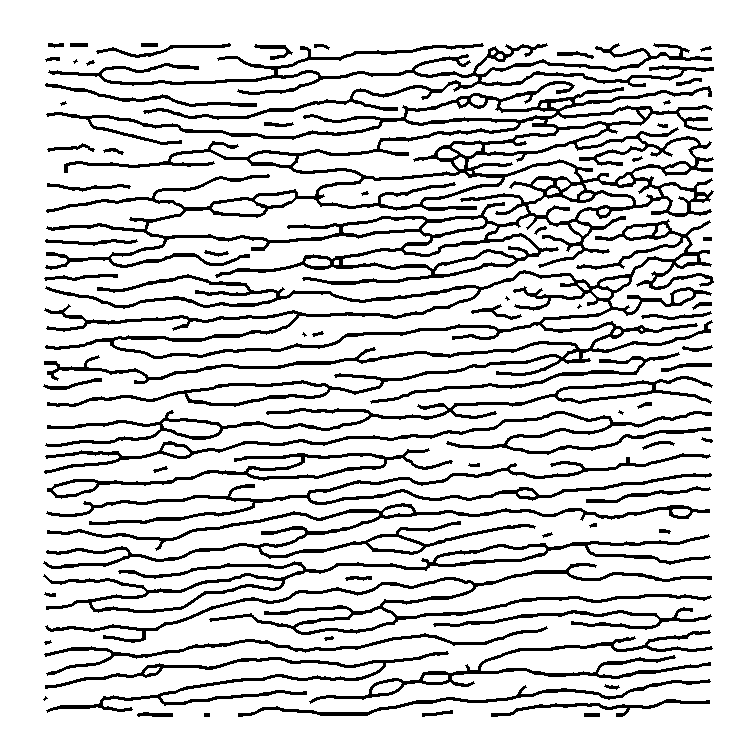

Supplement: Supplemental Information 3 [file peerj-07-7813-s003.zip › Supplemental-3/J-18-1.bmp]

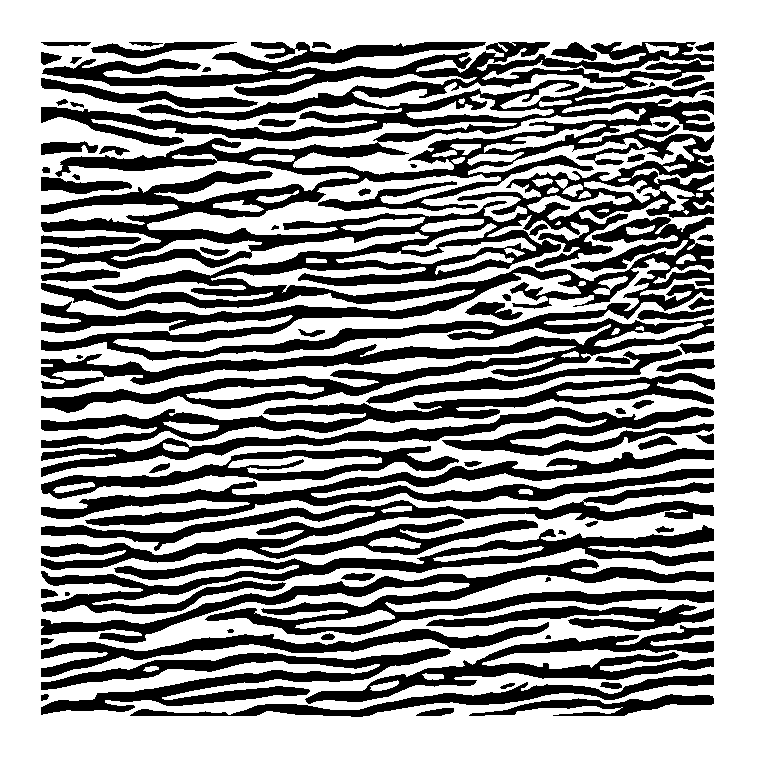

Supplement: Supplemental Information 3 [file peerj-07-7813-s003.zip › Supplemental-3/J-18.bmp]

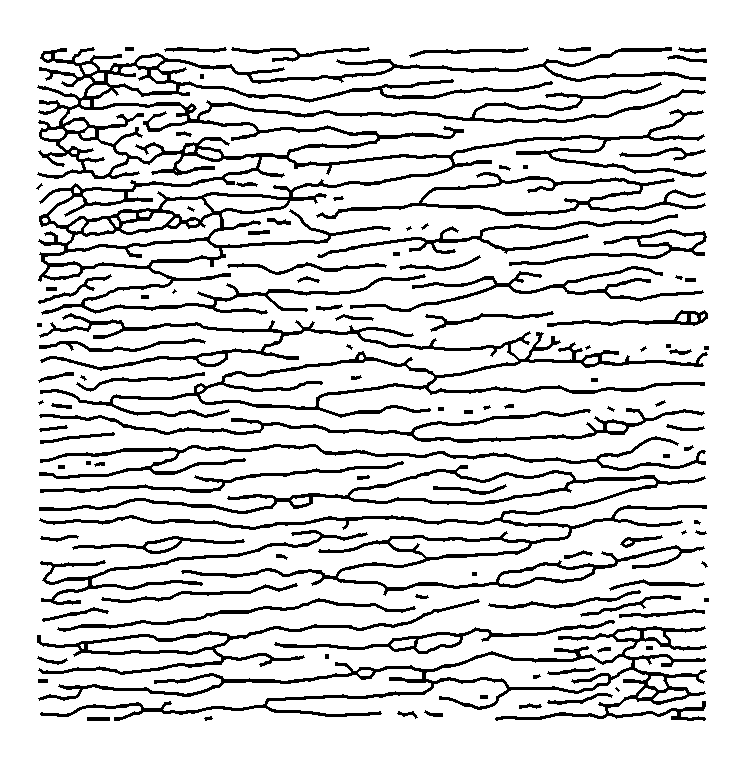

Supplement: Supplemental Information 3 [file peerj-07-7813-s003.zip › Supplemental-3/J-19-1.bmp]

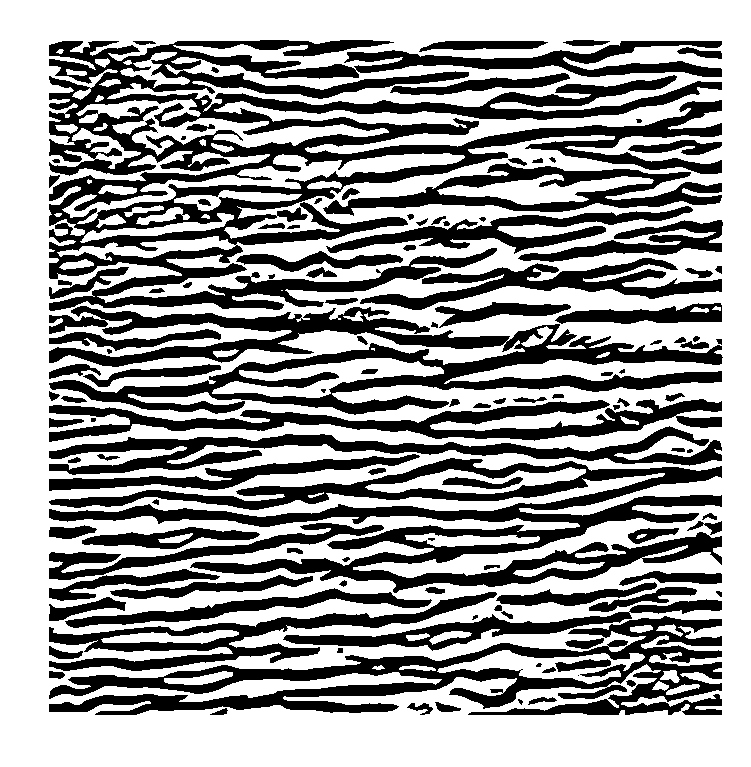

Supplement: Supplemental Information 3 [file peerj-07-7813-s003.zip › Supplemental-3/J-19.bmp]

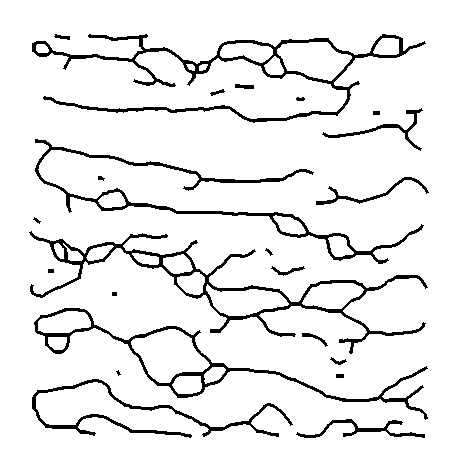

Supplement: Supplemental Information 4 [file peerj-07-7813-s004.zip › Supplemental-4/A-04-1.bmp]

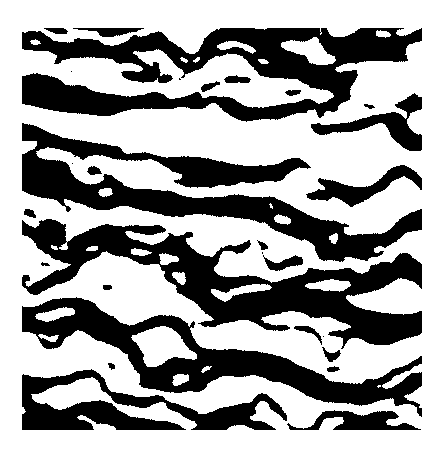

Supplement: Supplemental Information 4 [file peerj-07-7813-s004.zip › Supplemental-4/A-04.bmp]

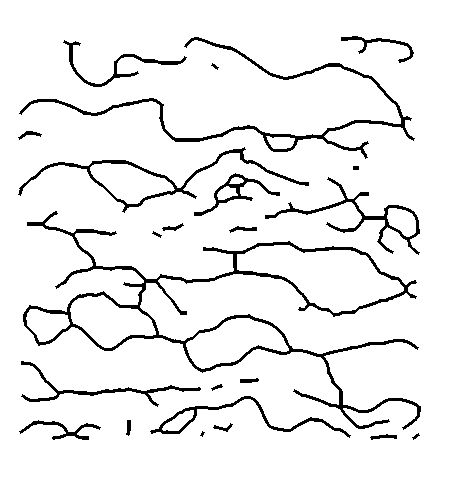

Supplement: Supplemental Information 4 [file peerj-07-7813-s004.zip › Supplemental-4/A-05-1.bmp]

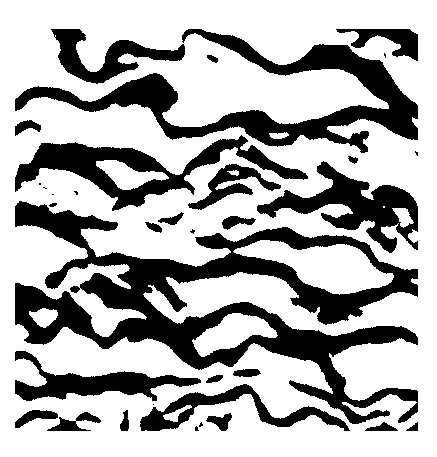

Supplement: Supplemental Information 4 [file peerj-07-7813-s004.zip › Supplemental-4/A-05.bmp]

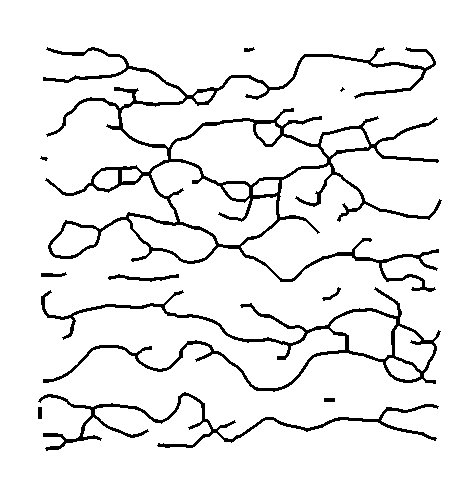

Supplement: Supplemental Information 4 [file peerj-07-7813-s004.zip › Supplemental-4/A-06-1.bmp]

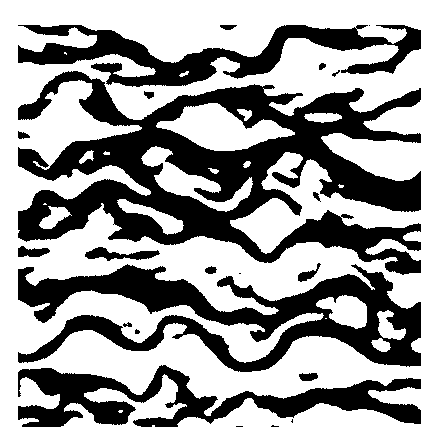

Supplement: Supplemental Information 4 [file peerj-07-7813-s004.zip › Supplemental-4/A-06.bmp]

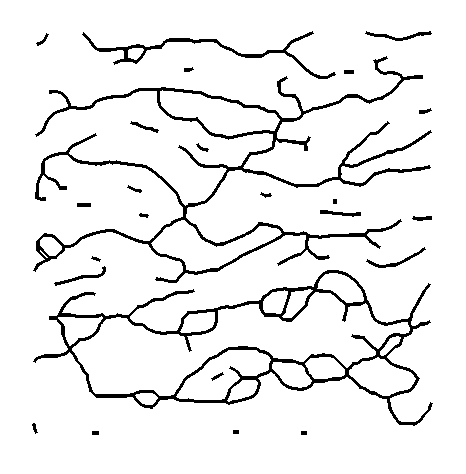

Supplement: Supplemental Information 4 [file peerj-07-7813-s004.zip › Supplemental-4/A-07-1.bmp]

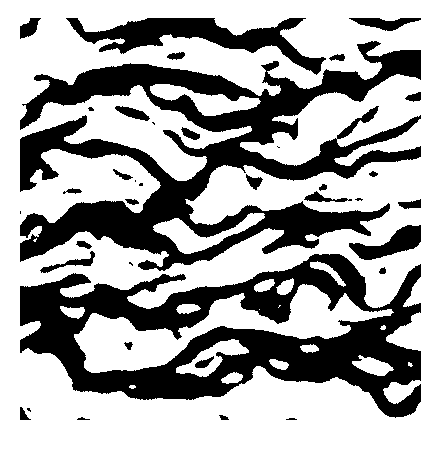

Supplement: Supplemental Information 4 [file peerj-07-7813-s004.zip › Supplemental-4/A-07.bmp]

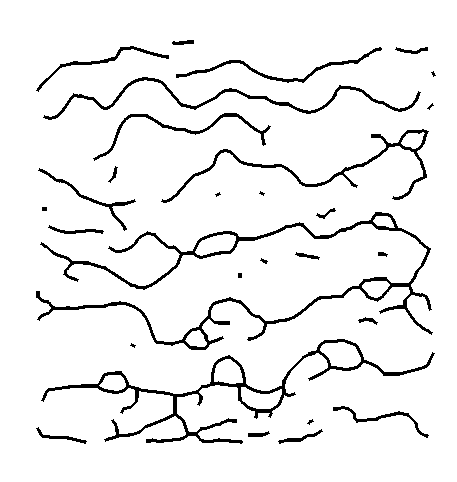

Supplement: Supplemental Information 4 [file peerj-07-7813-s004.zip › Supplemental-4/A-08-1.bmp]

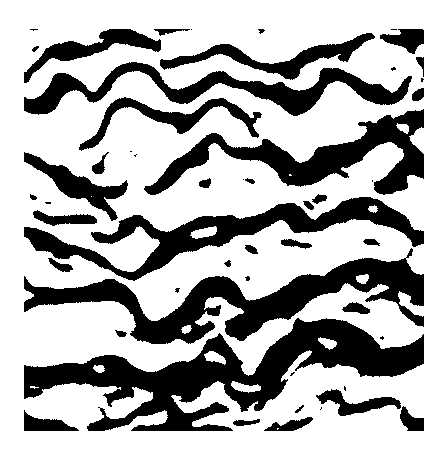

Supplement: Supplemental Information 4 [file peerj-07-7813-s004.zip › Supplemental-4/A-08.bmp]

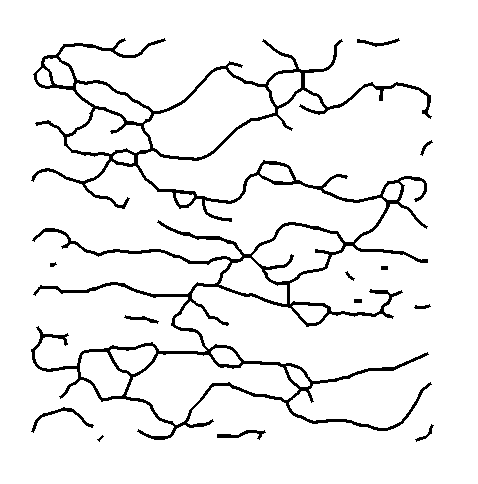

Supplement: Supplemental Information 4 [file peerj-07-7813-s004.zip › Supplemental-4/A-09-1.bmp]

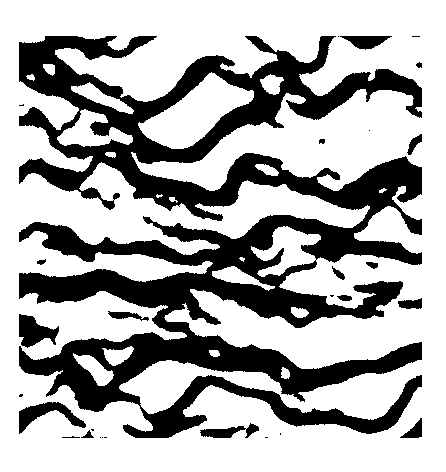

Supplement: Supplemental Information 4 [file peerj-07-7813-s004.zip › Supplemental-4/A-09.bmp]

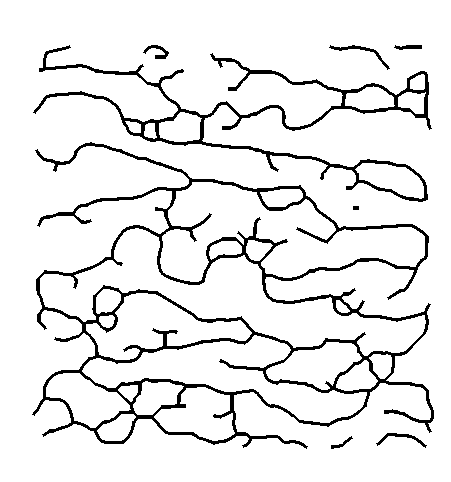

Supplement: Supplemental Information 4 [file peerj-07-7813-s004.zip › Supplemental-4/B-03-1.bmp]

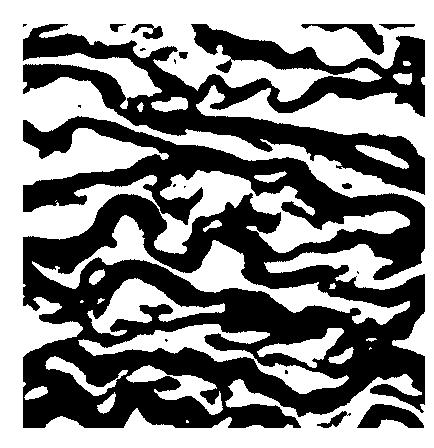

Supplement: Supplemental Information 4 [file peerj-07-7813-s004.zip › Supplemental-4/B-03.bmp]

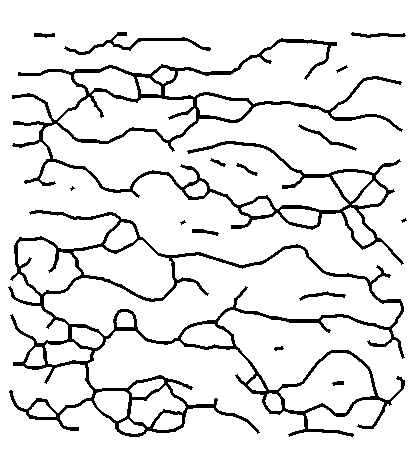

Supplement: Supplemental Information 4 [file peerj-07-7813-s004.zip › Supplemental-4/B-04-1.bmp]

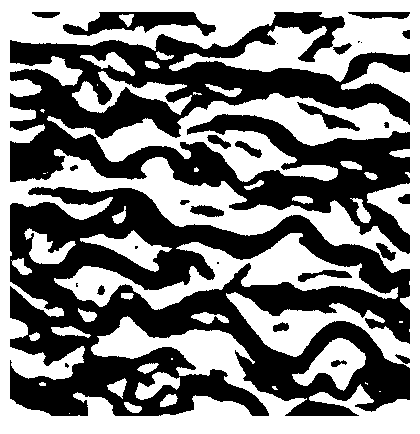

Supplement: Supplemental Information 4 [file peerj-07-7813-s004.zip › Supplemental-4/B-04.bmp]

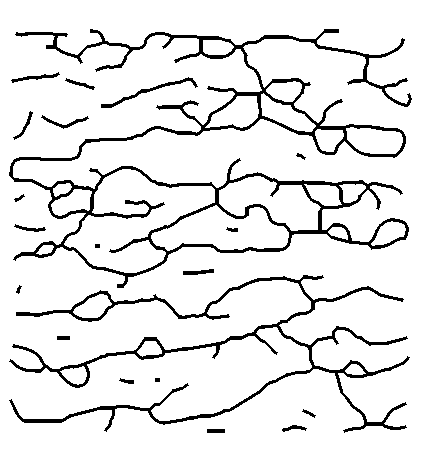

Supplement: Supplemental Information 4 [file peerj-07-7813-s004.zip › Supplemental-4/B-05-1.bmp]

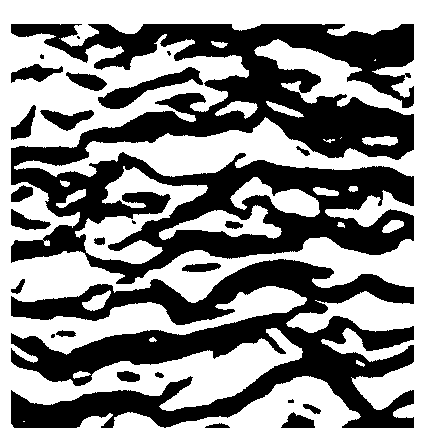

Supplement: Supplemental Information 4 [file peerj-07-7813-s004.zip › Supplemental-4/B-05.bmp]

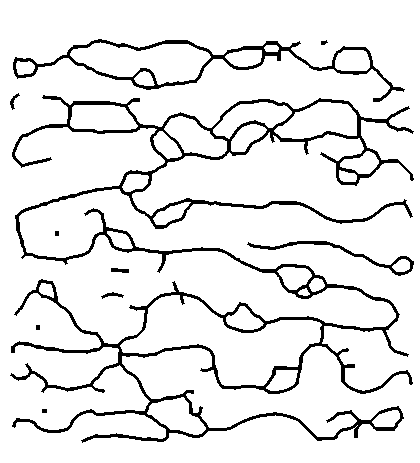

Supplement: Supplemental Information 4 [file peerj-07-7813-s004.zip › Supplemental-4/B-06-1.bmp]

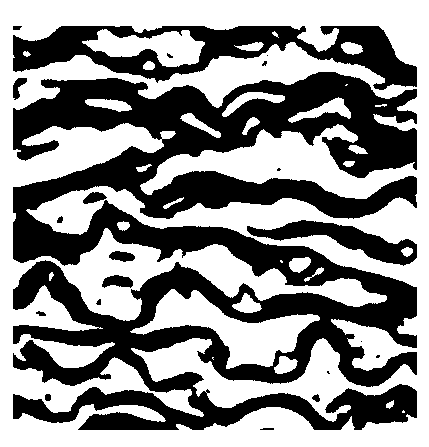

Supplement: Supplemental Information 4 [file peerj-07-7813-s004.zip › Supplemental-4/B-06.bmp]

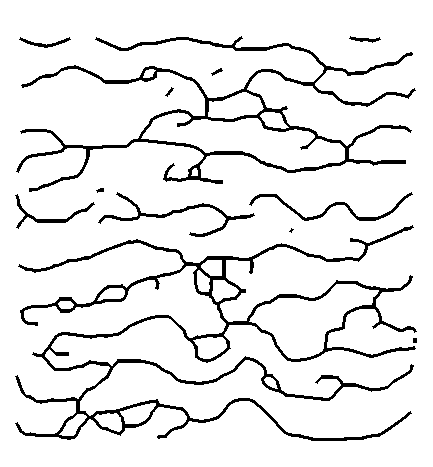

Supplement: Supplemental Information 4 [file peerj-07-7813-s004.zip › Supplemental-4/B-07-1.bmp]

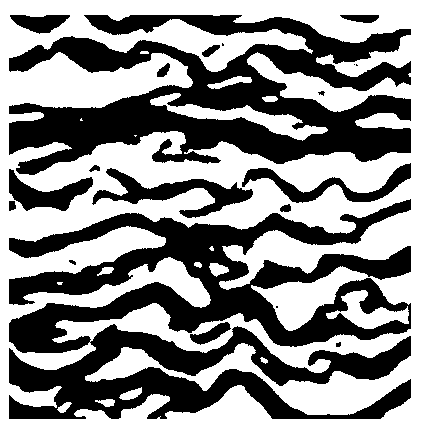

Supplement: Supplemental Information 4 [file peerj-07-7813-s004.zip › Supplemental-4/B-07.bmp]

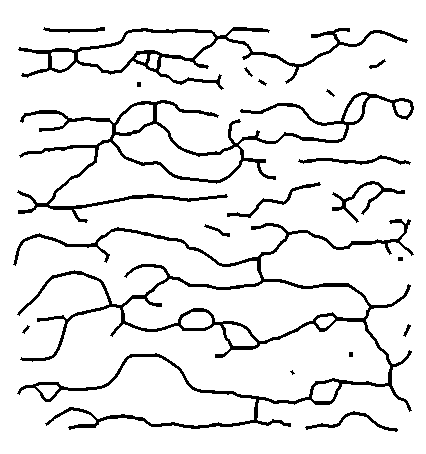

Supplement: Supplemental Information 4 [file peerj-07-7813-s004.zip › Supplemental-4/B-08-1.bmp]

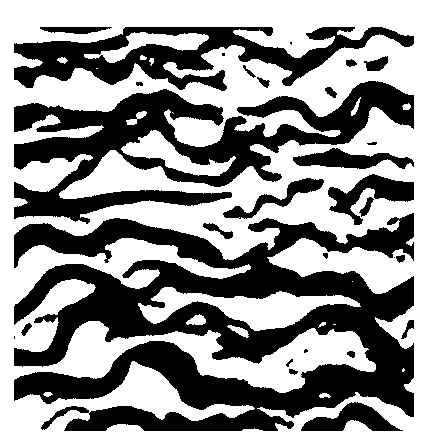

Supplement: Supplemental Information 4 [file peerj-07-7813-s004.zip › Supplemental-4/B-08.bmp]

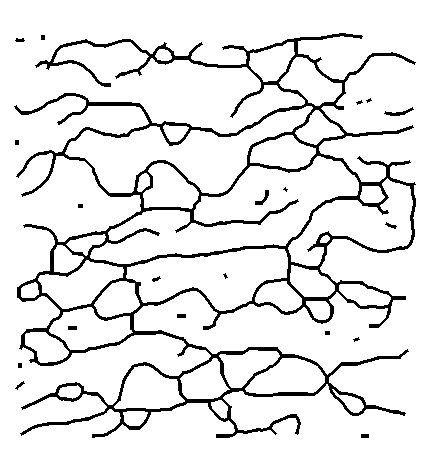

Supplement: Supplemental Information 4 [file peerj-07-7813-s004.zip › Supplemental-4/B-09-1.bmp]

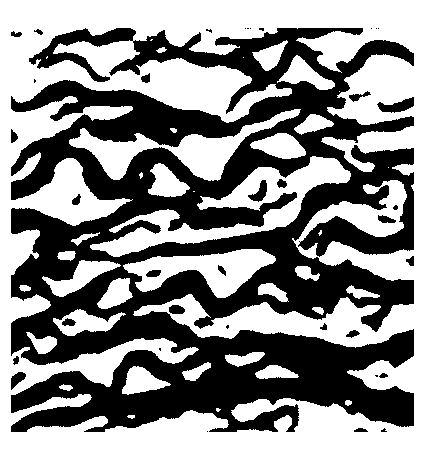

Supplement: Supplemental Information 4 [file peerj-07-7813-s004.zip › Supplemental-4/B-09.bmp]

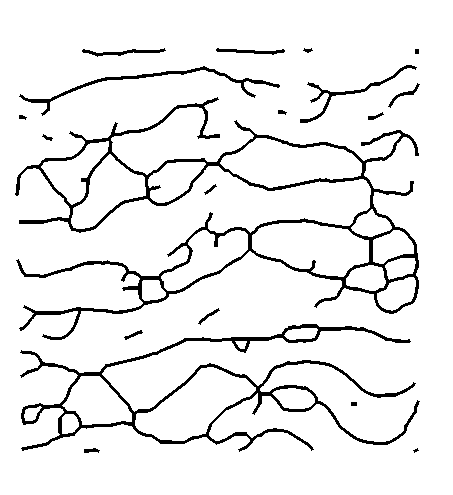

Supplement: Supplemental Information 4 [file peerj-07-7813-s004.zip › Supplemental-4/B-10-1.bmp]

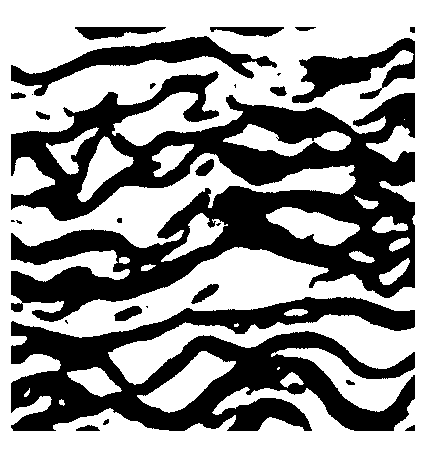

Supplement: Supplemental Information 4 [file peerj-07-7813-s004.zip › Supplemental-4/B-10.bmp]

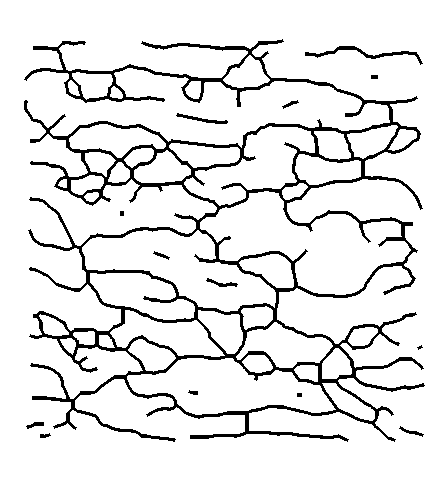

Supplement: Supplemental Information 4 [file peerj-07-7813-s004.zip › Supplemental-4/C-02-1.bmp]

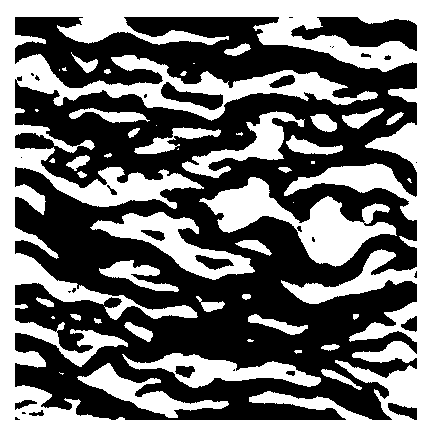

Supplement: Supplemental Information 4 [file peerj-07-7813-s004.zip › Supplemental-4/C-02.bmp]

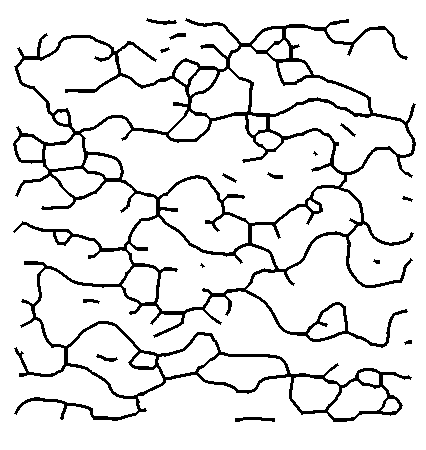

Supplement: Supplemental Information 4 [file peerj-07-7813-s004.zip › Supplemental-4/C-03-1.bmp]

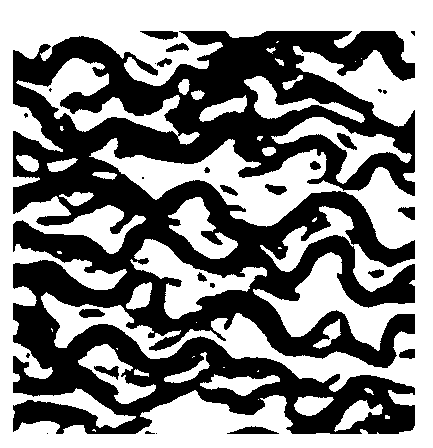

Supplement: Supplemental Information 4 [file peerj-07-7813-s004.zip › Supplemental-4/C-03.bmp]

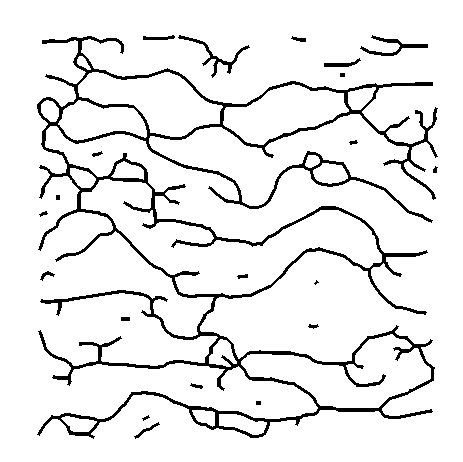

Supplement: Supplemental Information 4 [file peerj-07-7813-s004.zip › Supplemental-4/C-04-1.bmp]

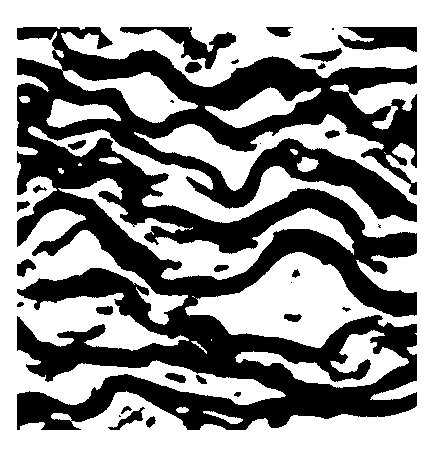

Supplement: Supplemental Information 4 [file peerj-07-7813-s004.zip › Supplemental-4/C-04.bmp]

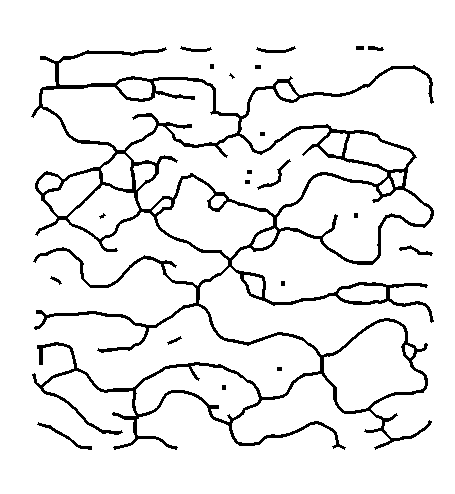

Supplement: Supplemental Information 4 [file peerj-07-7813-s004.zip › Supplemental-4/C-05-1.bmp]

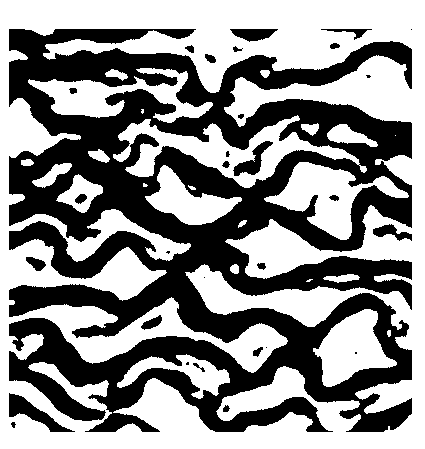

Supplement: Supplemental Information 4 [file peerj-07-7813-s004.zip › Supplemental-4/C-05.bmp]

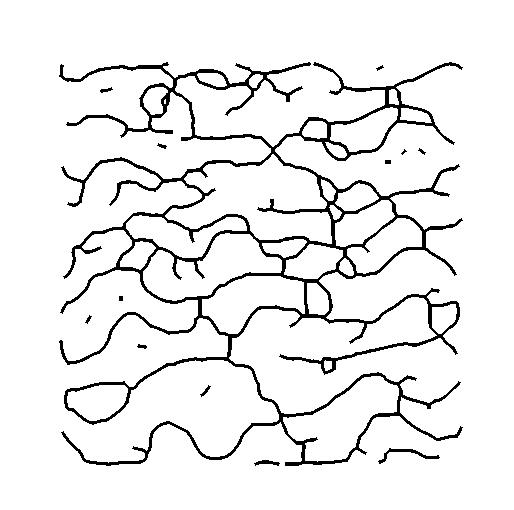

Supplement: Supplemental Information 4 [file peerj-07-7813-s004.zip › Supplemental-4/C-06-1.bmp]

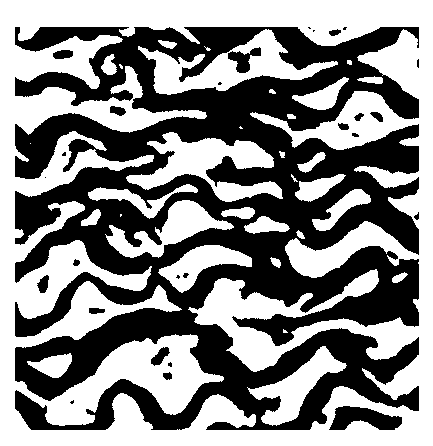

Supplement: Supplemental Information 4 [file peerj-07-7813-s004.zip › Supplemental-4/C-06.bmp]

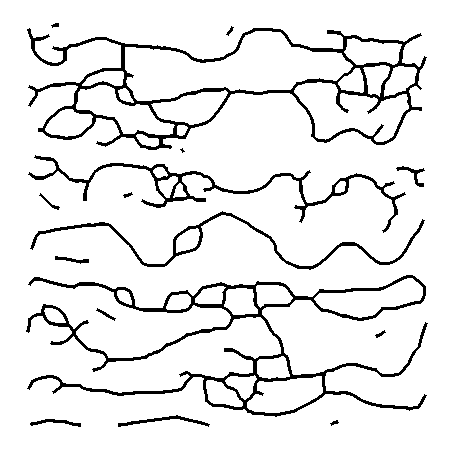

Supplement: Supplemental Information 4 [file peerj-07-7813-s004.zip › Supplemental-4/C-07-1.bmp]

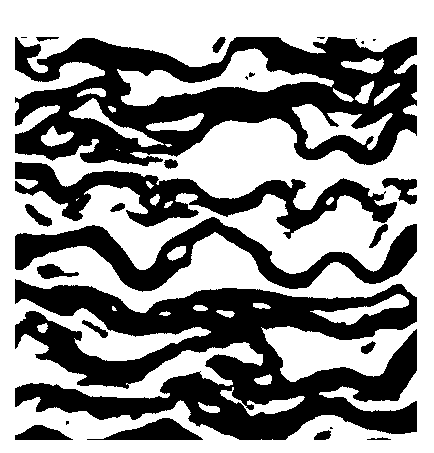

Supplement: Supplemental Information 4 [file peerj-07-7813-s004.zip › Supplemental-4/C-07.bmp]

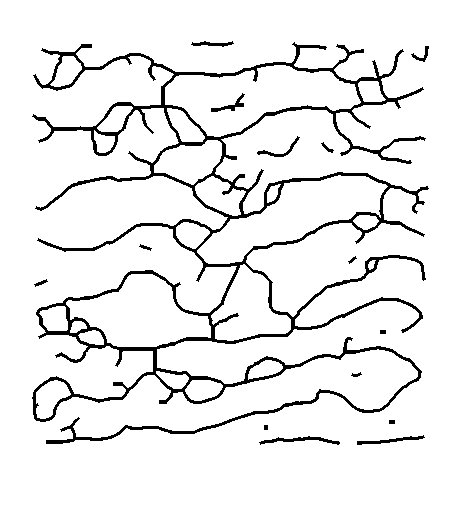

Supplement: Supplemental Information 4 [file peerj-07-7813-s004.zip › Supplemental-4/C-08-1.bmp]

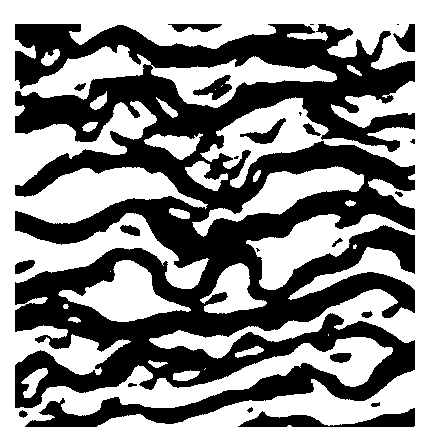

Supplement: Supplemental Information 4 [file peerj-07-7813-s004.zip › Supplemental-4/C-08.bmp]

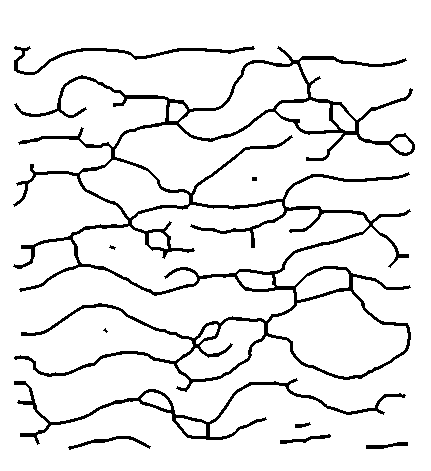

Supplement: Supplemental Information 4 [file peerj-07-7813-s004.zip › Supplemental-4/C-09-1.bmp]

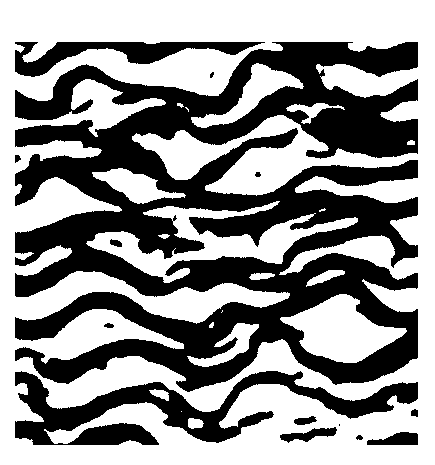

Supplement: Supplemental Information 4 [file peerj-07-7813-s004.zip › Supplemental-4/C-09.bmp]

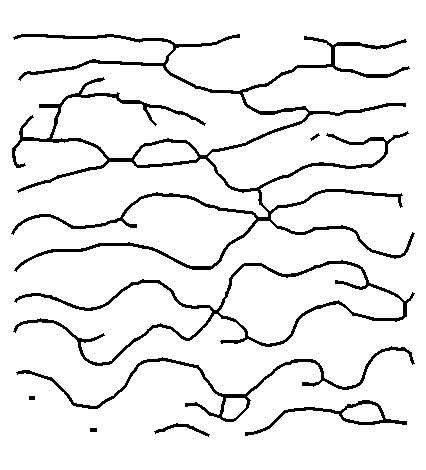

Supplement: Supplemental Information 4 [file peerj-07-7813-s004.zip › Supplemental-4/C-10-1.bmp]

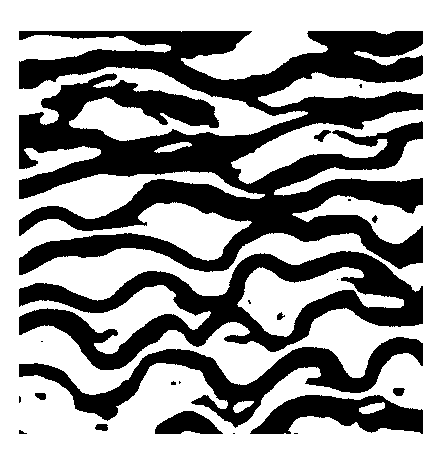

Supplement: Supplemental Information 4 [file peerj-07-7813-s004.zip › Supplemental-4/C-10.bmp]

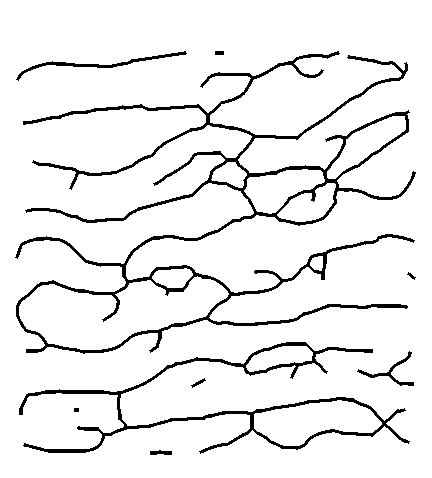

Supplement: Supplemental Information 4 [file peerj-07-7813-s004.zip › Supplemental-4/C-11-1.bmp]

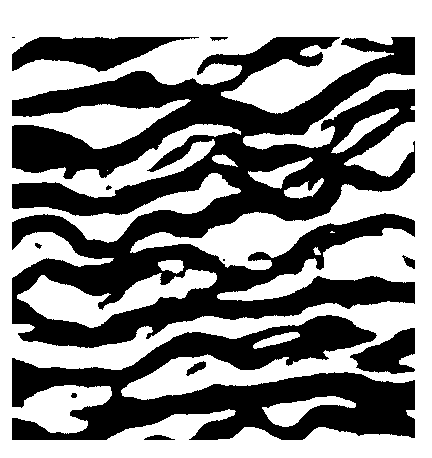

Supplement: Supplemental Information 4 [file peerj-07-7813-s004.zip › Supplemental-4/C-11.bmp]

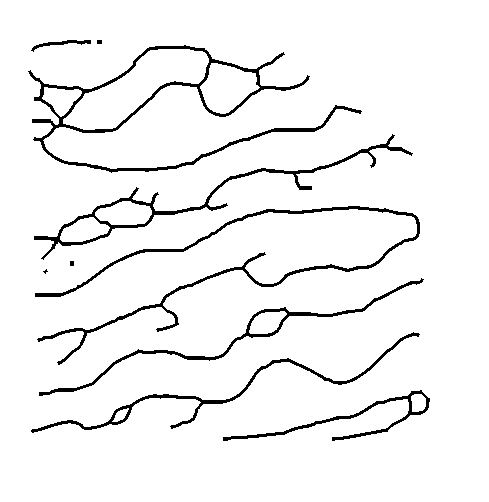

Supplement: Supplemental Information 4 [file peerj-07-7813-s004.zip › Supplemental-4/C-12-1.bmp]

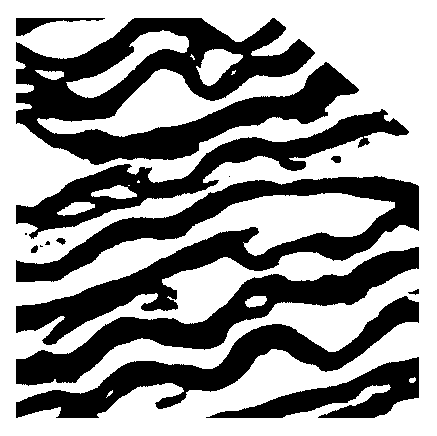

Supplement: Supplemental Information 4 [file peerj-07-7813-s004.zip › Supplemental-4/C-12.bmp]

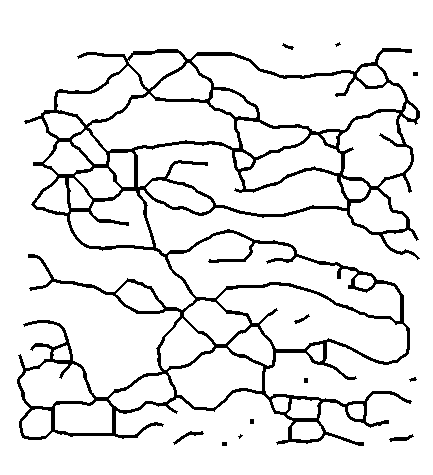

Supplement: Supplemental Information 4 [file peerj-07-7813-s004.zip › Supplemental-4/D-01-1.bmp]

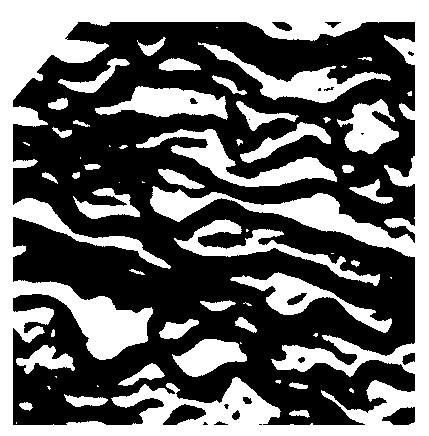

Supplement: Supplemental Information 4 [file peerj-07-7813-s004.zip › Supplemental-4/D-01.bmp]

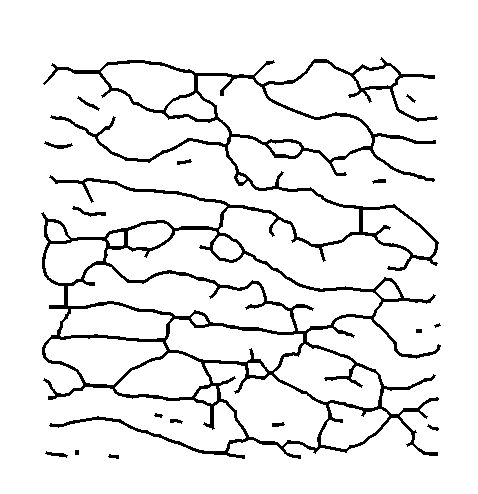

Supplement: Supplemental Information 4 [file peerj-07-7813-s004.zip › Supplemental-4/D-02-1.bmp]

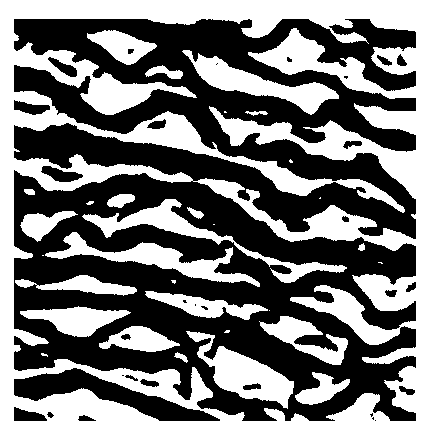

Supplement: Supplemental Information 4 [file peerj-07-7813-s004.zip › Supplemental-4/D-02.bmp]

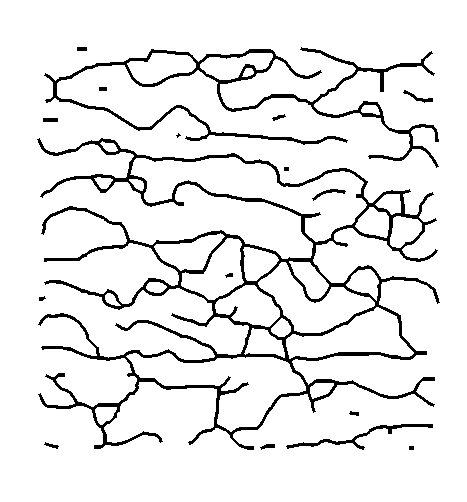

Supplement: Supplemental Information 4 [file peerj-07-7813-s004.zip › Supplemental-4/D-03-1.bmp]

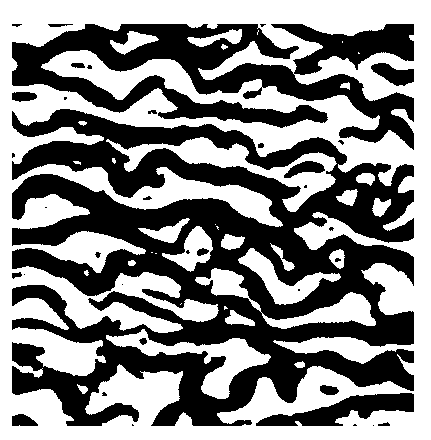

Supplement: Supplemental Information 4 [file peerj-07-7813-s004.zip › Supplemental-4/D-03.bmp]

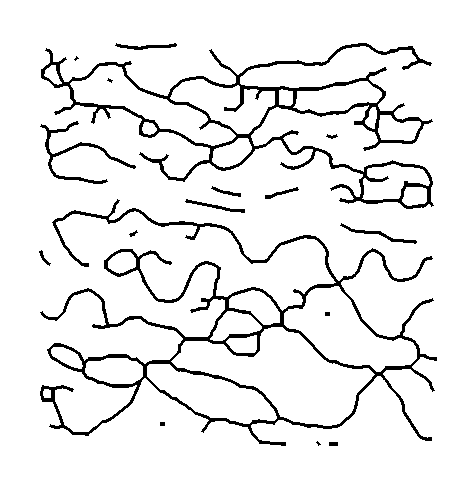

Supplement: Supplemental Information 4 [file peerj-07-7813-s004.zip › Supplemental-4/D-04-1.bmp]

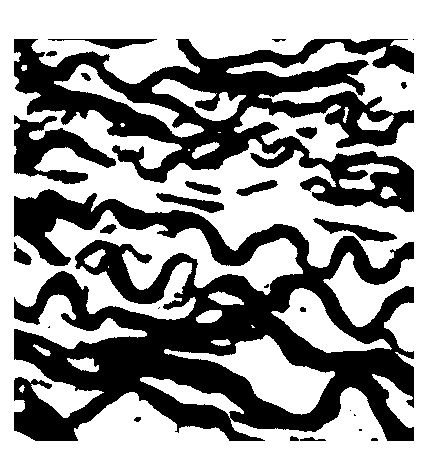

Supplement: Supplemental Information 4 [file peerj-07-7813-s004.zip › Supplemental-4/D-04.bmp]

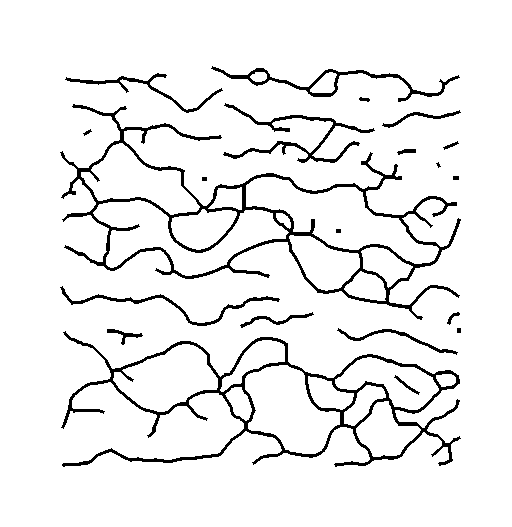

Supplement: Supplemental Information 4 [file peerj-07-7813-s004.zip › Supplemental-4/D-05-1.bmp]

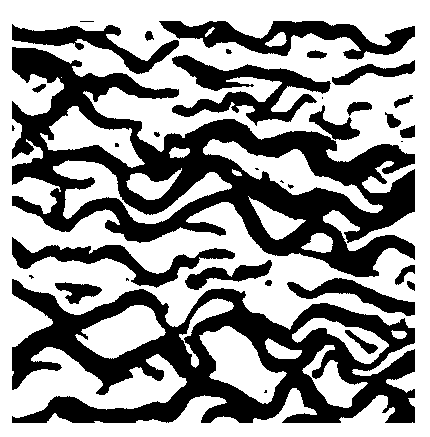

Supplement: Supplemental Information 4 [file peerj-07-7813-s004.zip › Supplemental-4/D-05.bmp]

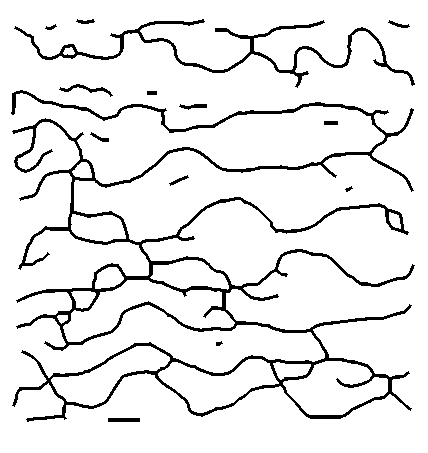

Supplement: Supplemental Information 4 [file peerj-07-7813-s004.zip › Supplemental-4/D-06-1.bmp]

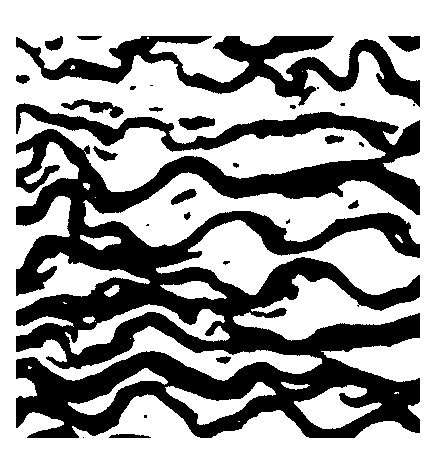

Supplement: Supplemental Information 4 [file peerj-07-7813-s004.zip › Supplemental-4/D-06.bmp]

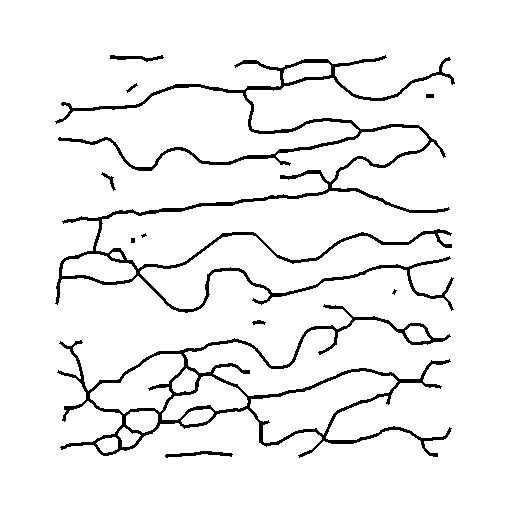

Supplement: Supplemental Information 4 [file peerj-07-7813-s004.zip › Supplemental-4/D-07-1.bmp]

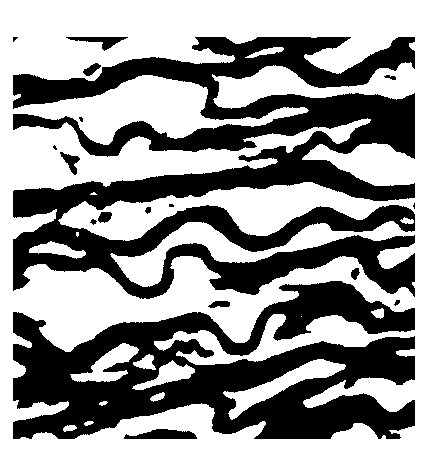

Supplement: Supplemental Information 4 [file peerj-07-7813-s004.zip › Supplemental-4/D-07.bmp]

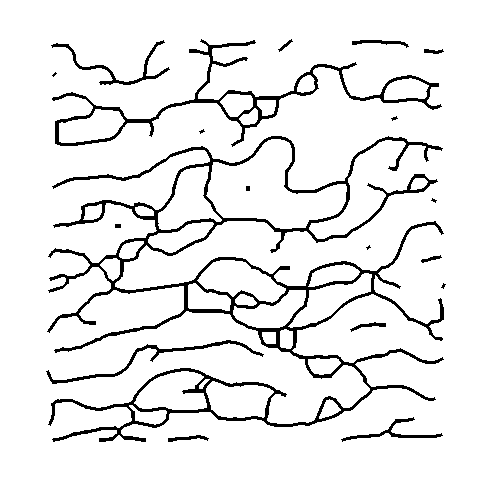

Supplement: Supplemental Information 4 [file peerj-07-7813-s004.zip › Supplemental-4/D-08-1.bmp]

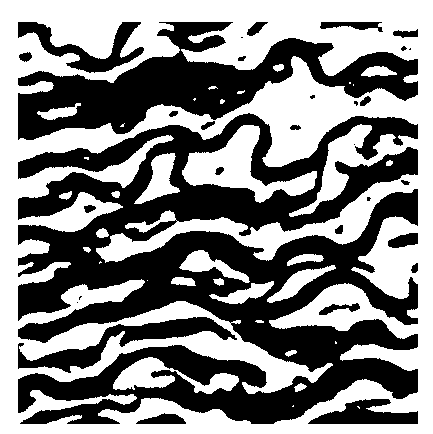

Supplement: Supplemental Information 4 [file peerj-07-7813-s004.zip › Supplemental-4/D-08.bmp]

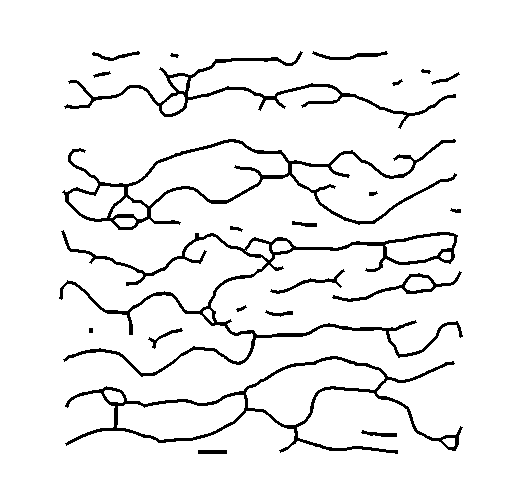

Supplement: Supplemental Information 4 [file peerj-07-7813-s004.zip › Supplemental-4/D-09-1.bmp]

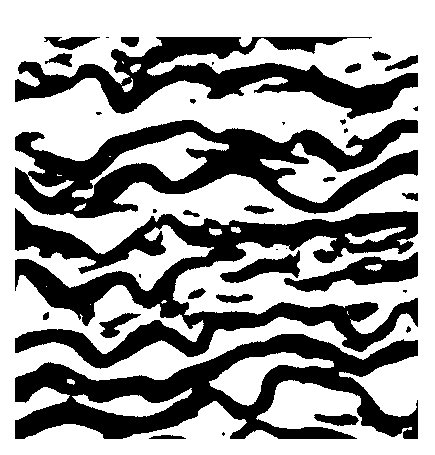

Supplement: Supplemental Information 4 [file peerj-07-7813-s004.zip › Supplemental-4/D-09.bmp]

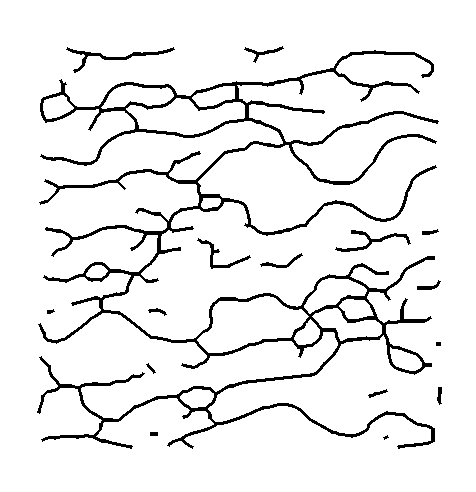

Supplement: Supplemental Information 4 [file peerj-07-7813-s004.zip › Supplemental-4/D-10-1.bmp]

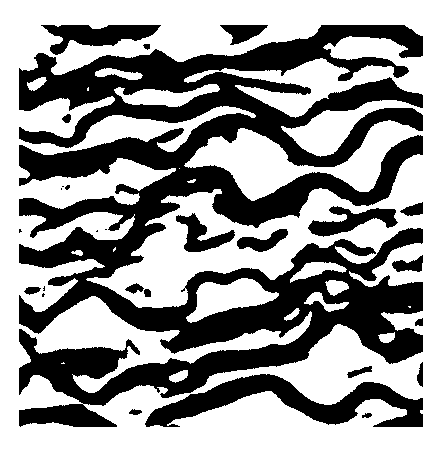

Supplement: Supplemental Information 4 [file peerj-07-7813-s004.zip › Supplemental-4/D-10.bmp]

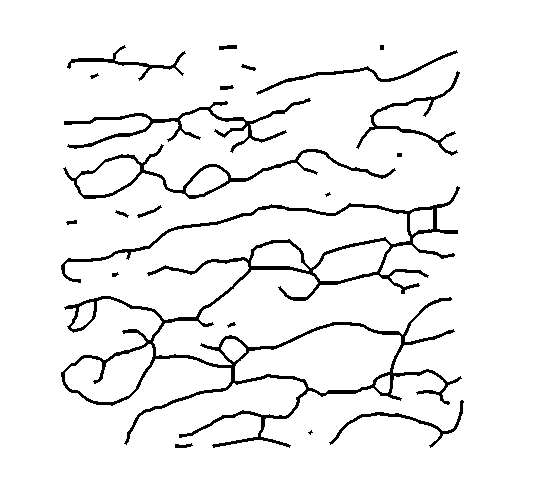

Supplement: Supplemental Information 4 [file peerj-07-7813-s004.zip › Supplemental-4/D-11-1.bmp]

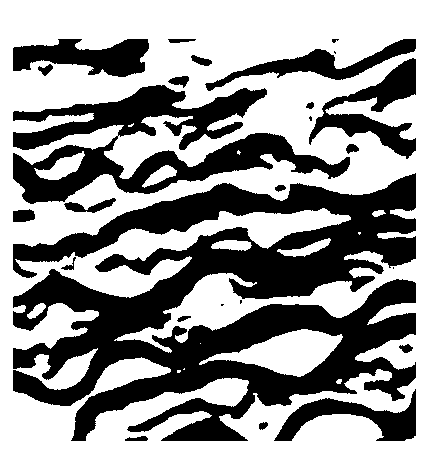

Supplement: Supplemental Information 4 [file peerj-07-7813-s004.zip › Supplemental-4/D-11.bmp]

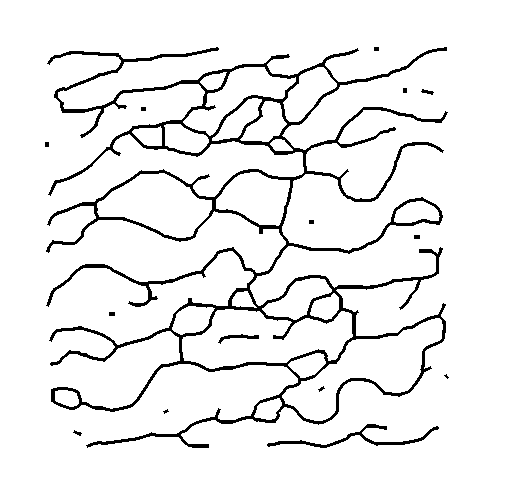

Supplement: Supplemental Information 4 [file peerj-07-7813-s004.zip › Supplemental-4/D-12-1.bmp]
