# Supplementary figures and images for: Layered patterns in nature, medicine, and materials: quantifying anisotropic structures and cyclicity (part 2 of 4)
Source: PeerJ. 2019 Oct 14;7:e7813. doi: 10.7717/peerj.7813 (PMC6797002; doi:10.7717/peerj.7813)

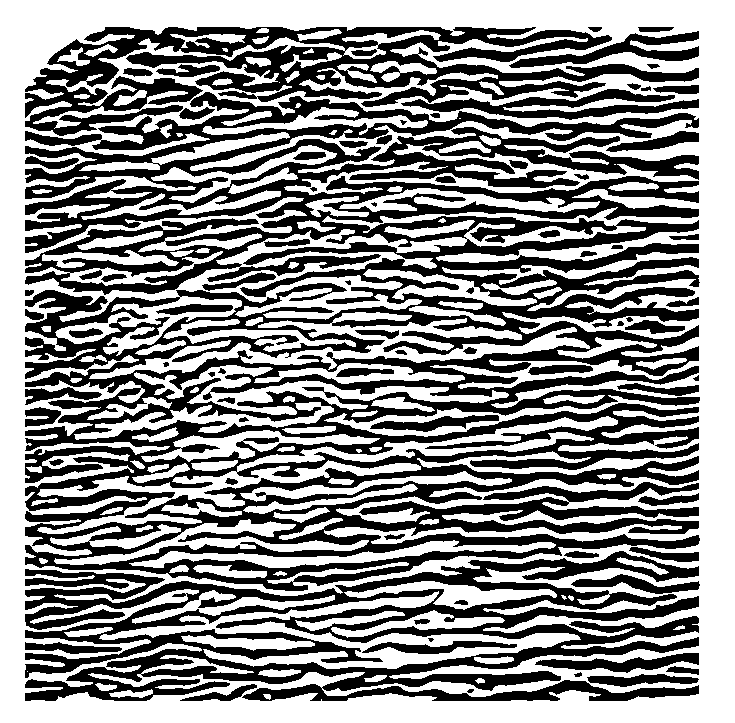

Supplement: Supplemental Information 3 [file peerj-07-7813-s003.zip › Supplemental-3/G-03.bmp]

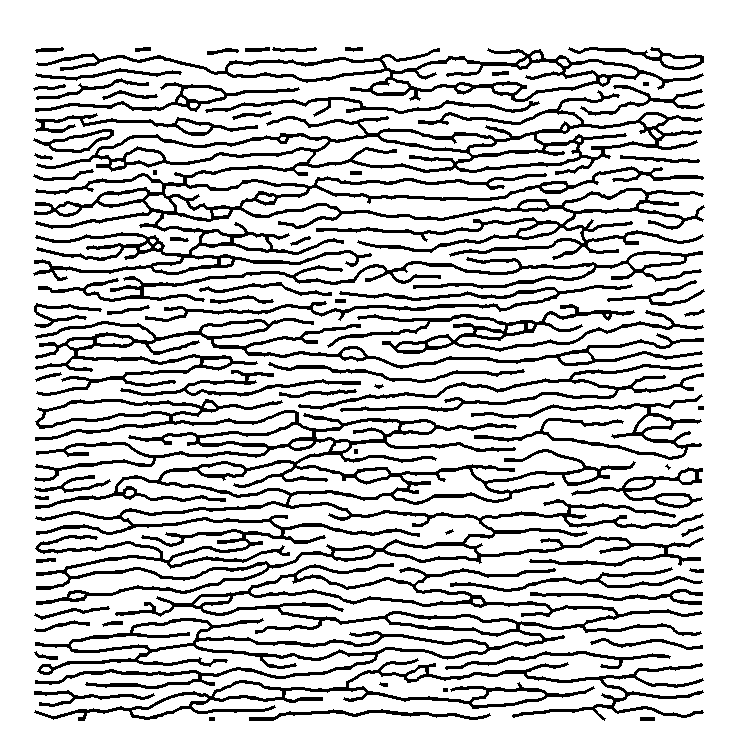

Supplement: Supplemental Information 3 [file peerj-07-7813-s003.zip › Supplemental-3/G-04-1.bmp]

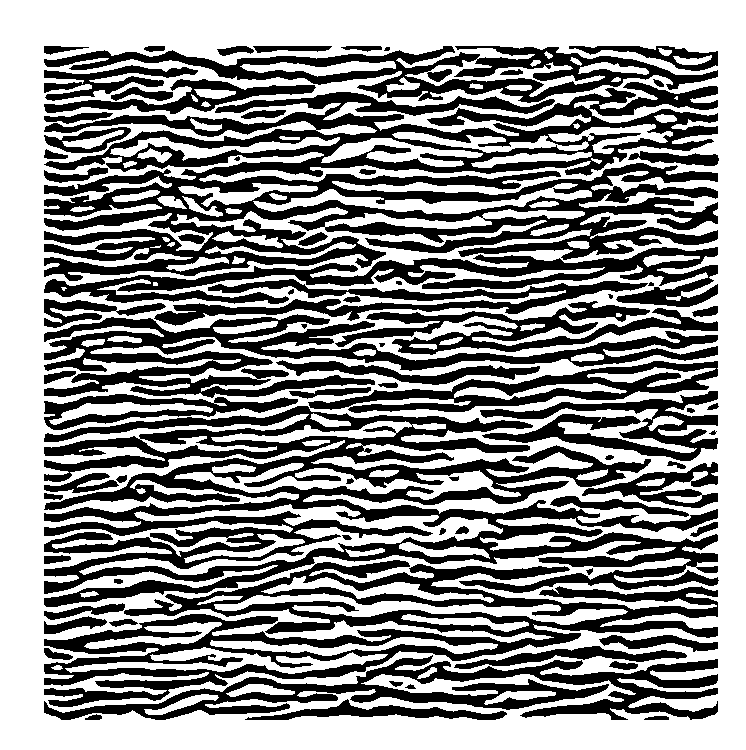

Supplement: Supplemental Information 3 [file peerj-07-7813-s003.zip › Supplemental-3/G-04.bmp]

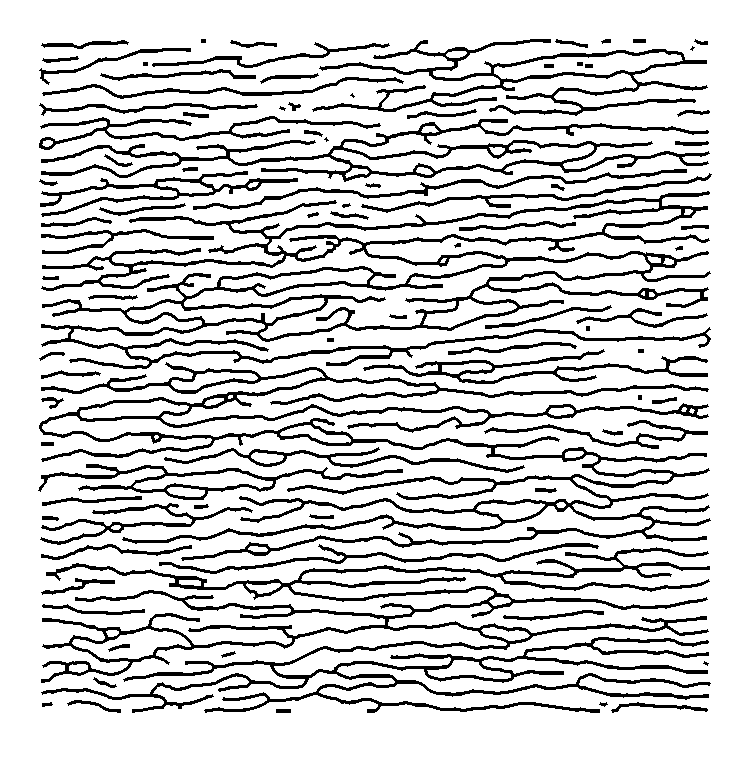

Supplement: Supplemental Information 3 [file peerj-07-7813-s003.zip › Supplemental-3/G-05-1.bmp]

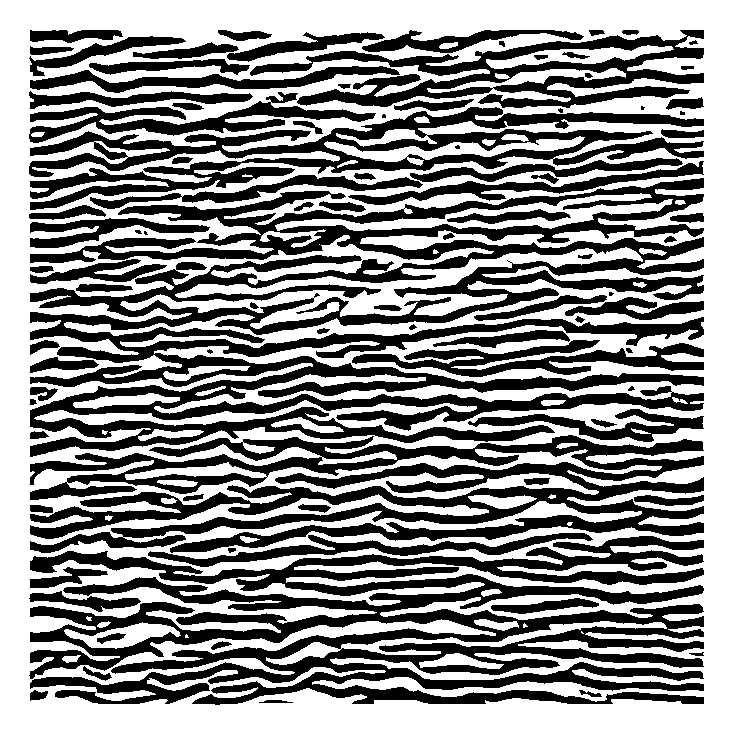

Supplement: Supplemental Information 3 [file peerj-07-7813-s003.zip › Supplemental-3/G-05.bmp]

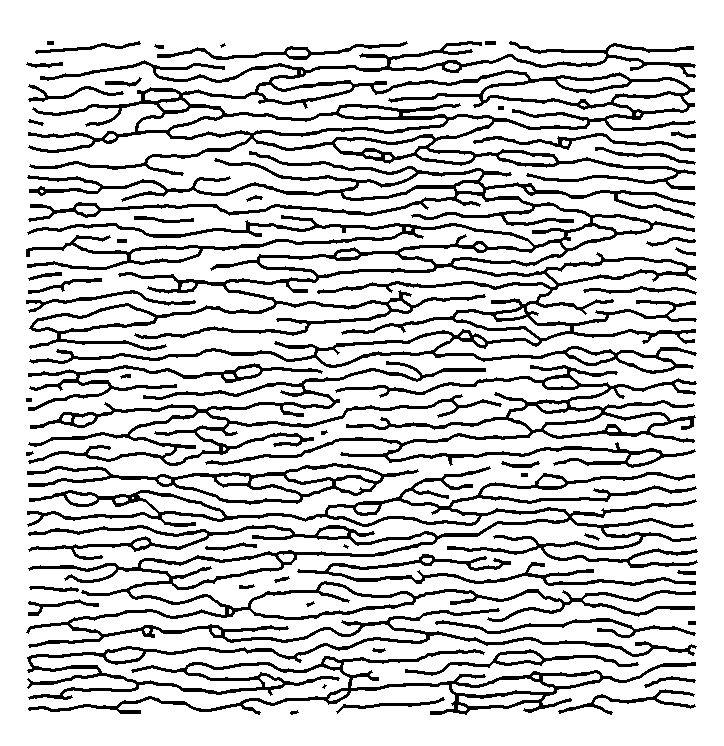

Supplement: Supplemental Information 3 [file peerj-07-7813-s003.zip › Supplemental-3/G-06-1.bmp]

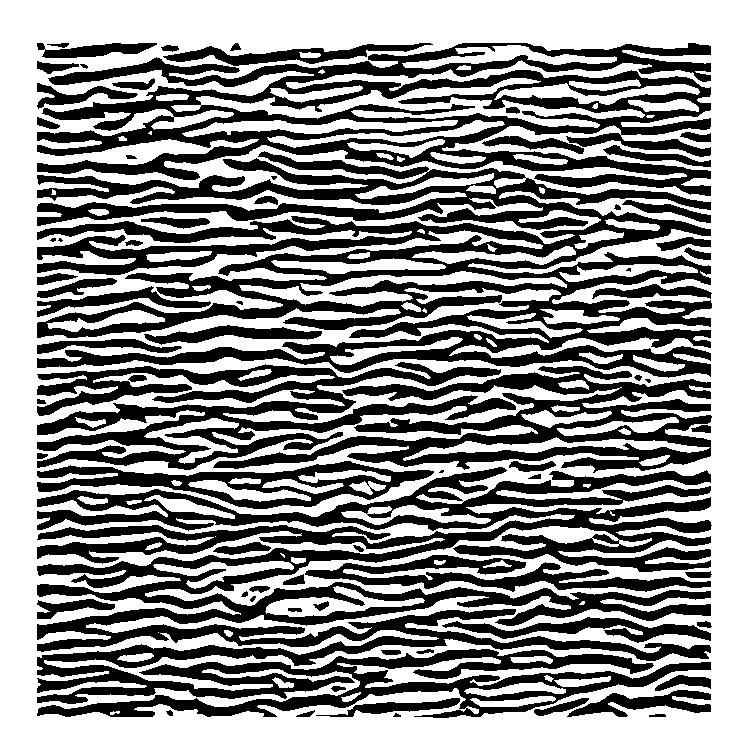

Supplement: Supplemental Information 3 [file peerj-07-7813-s003.zip › Supplemental-3/G-06.bmp]

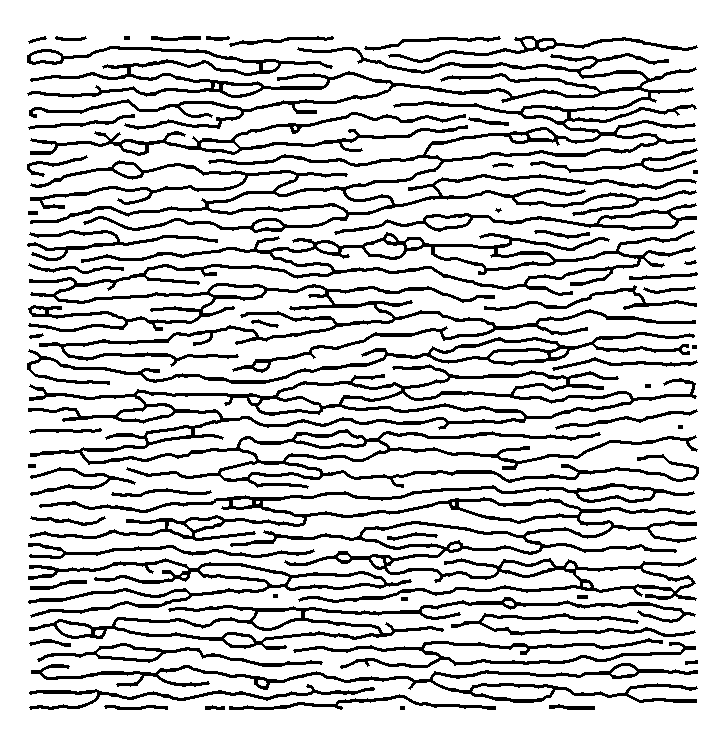

Supplement: Supplemental Information 3 [file peerj-07-7813-s003.zip › Supplemental-3/G-07-1.bmp]

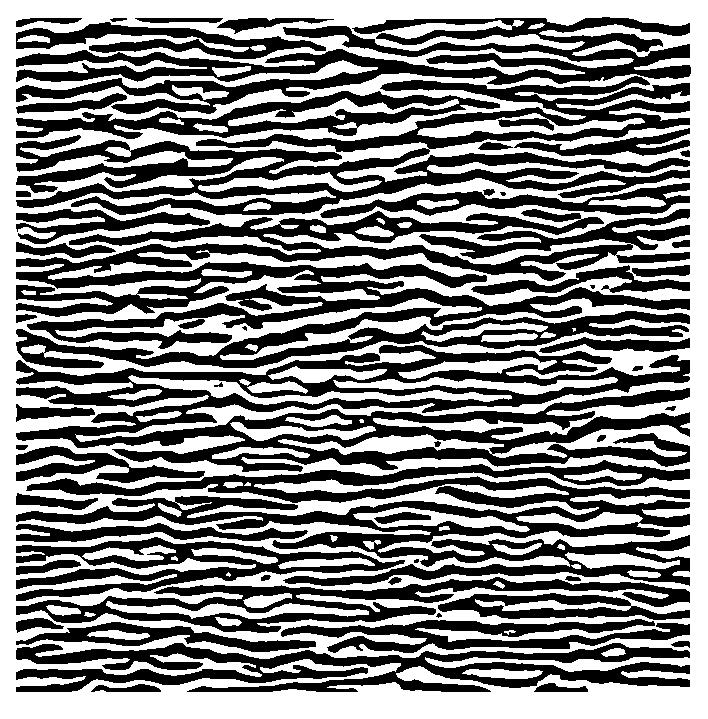

Supplement: Supplemental Information 3 [file peerj-07-7813-s003.zip › Supplemental-3/G-07.bmp]

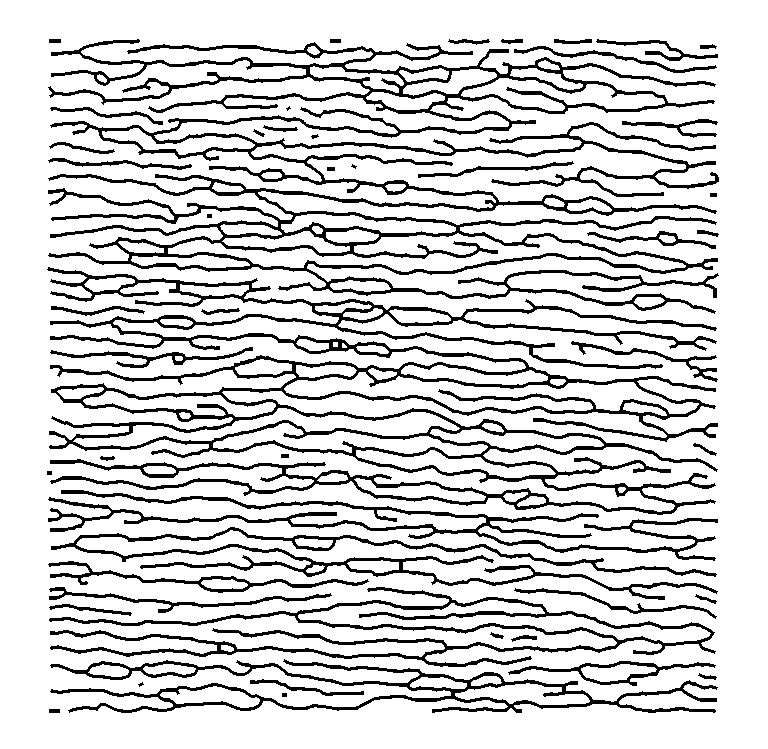

Supplement: Supplemental Information 3 [file peerj-07-7813-s003.zip › Supplemental-3/G-08-1.bmp]

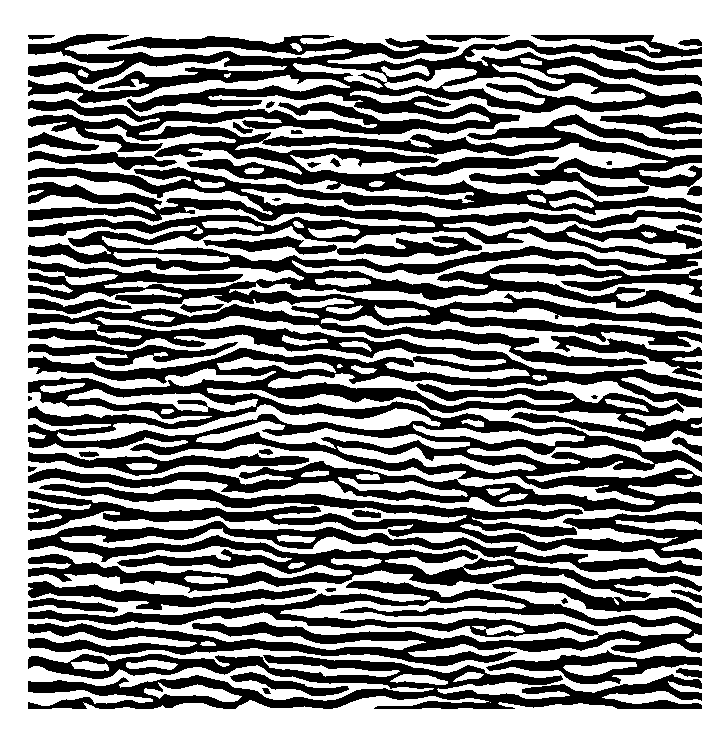

Supplement: Supplemental Information 3 [file peerj-07-7813-s003.zip › Supplemental-3/G-08.bmp]

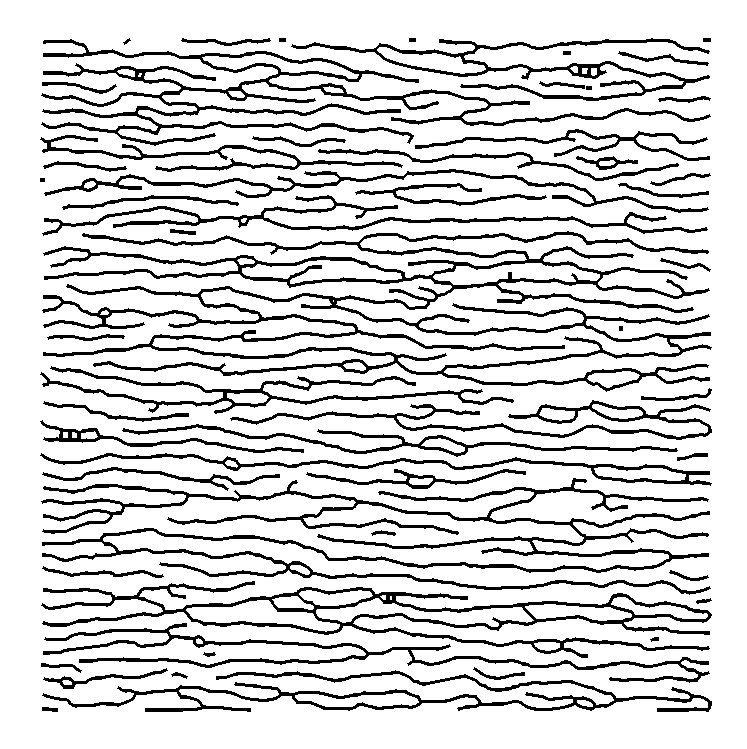

Supplement: Supplemental Information 3 [file peerj-07-7813-s003.zip › Supplemental-3/G-09-1.bmp]

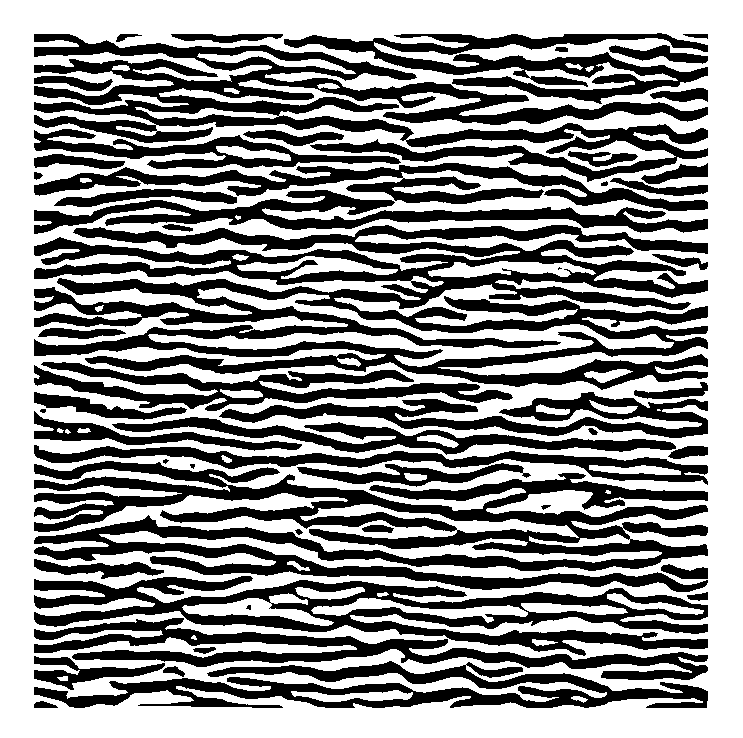

Supplement: Supplemental Information 3 [file peerj-07-7813-s003.zip › Supplemental-3/G-09.bmp]

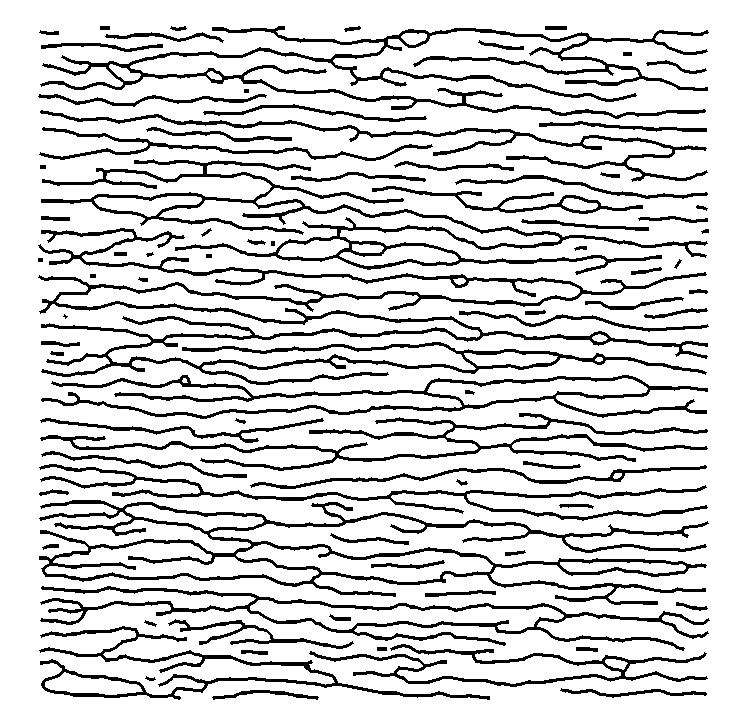

Supplement: Supplemental Information 3 [file peerj-07-7813-s003.zip › Supplemental-3/G-10-1.bmp]

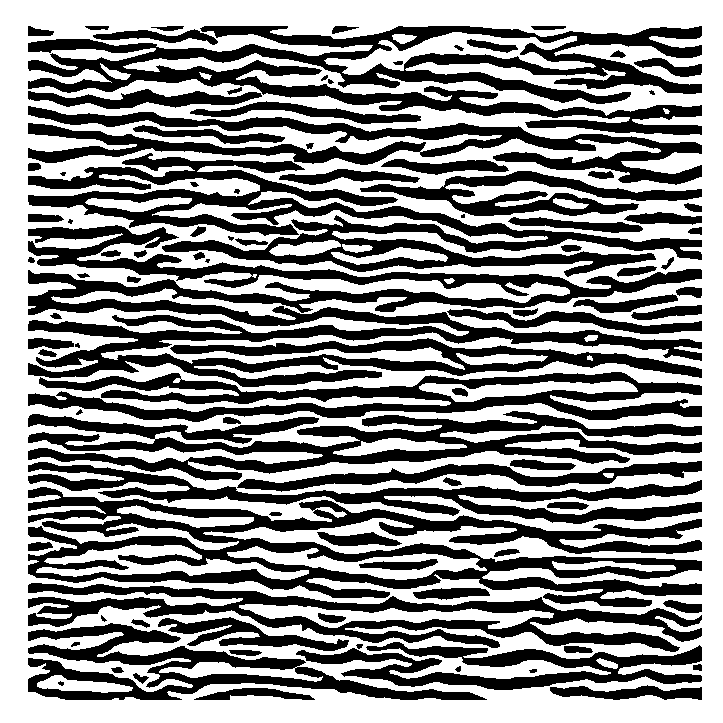

Supplement: Supplemental Information 3 [file peerj-07-7813-s003.zip › Supplemental-3/G-10.bmp]

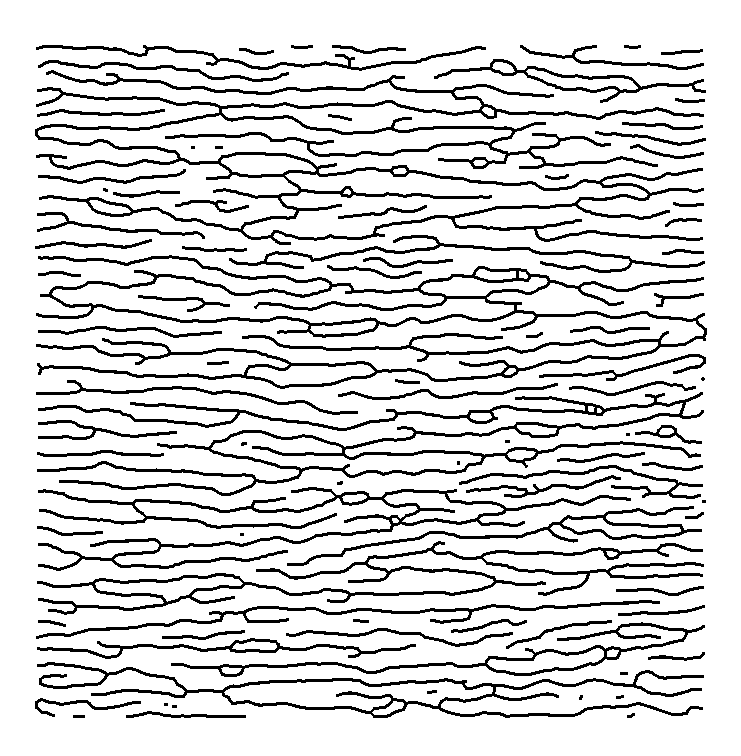

Supplement: Supplemental Information 3 [file peerj-07-7813-s003.zip › Supplemental-3/G-11-1.bmp]

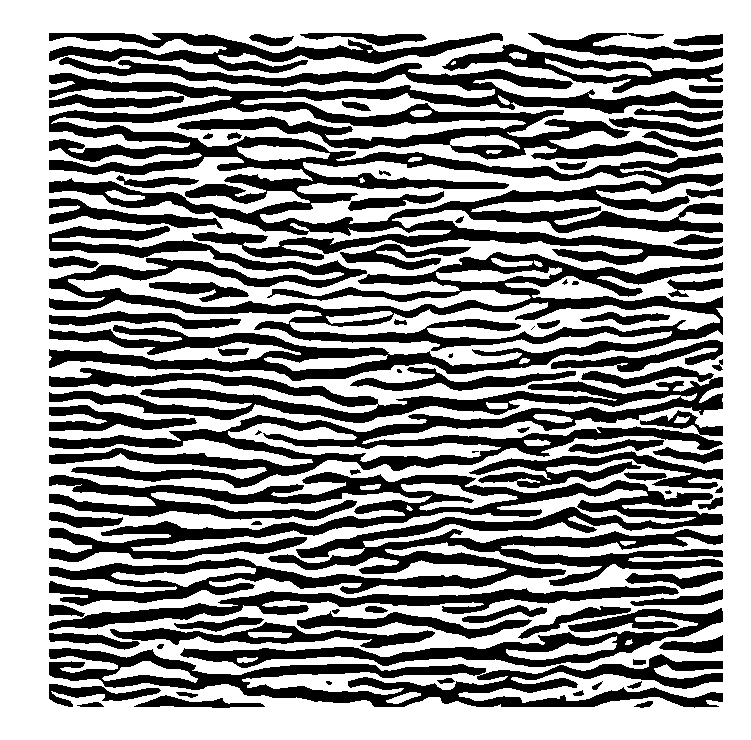

Supplement: Supplemental Information 3 [file peerj-07-7813-s003.zip › Supplemental-3/G-11.bmp]

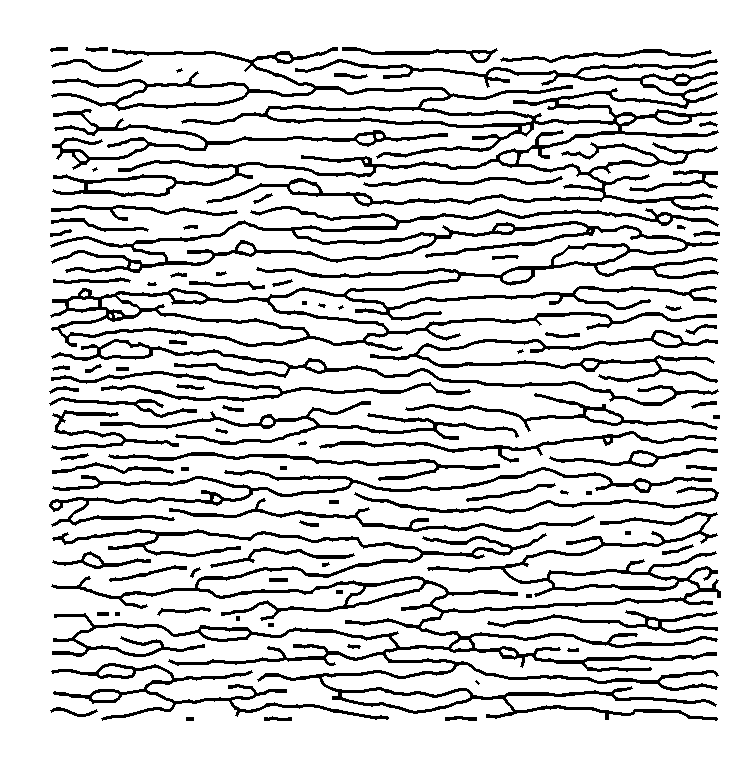

Supplement: Supplemental Information 3 [file peerj-07-7813-s003.zip › Supplemental-3/G-12-1.bmp]

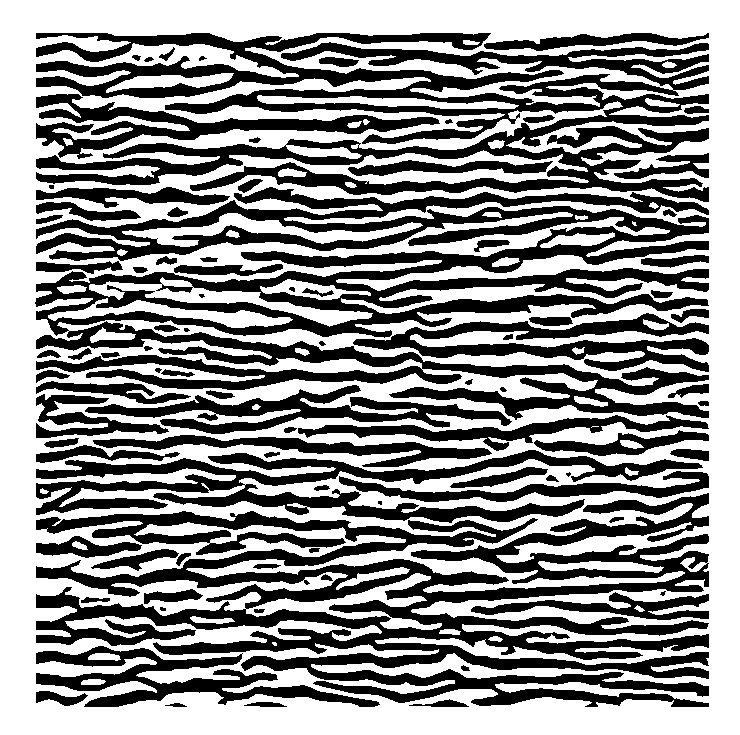

Supplement: Supplemental Information 3 [file peerj-07-7813-s003.zip › Supplemental-3/G-12.bmp]

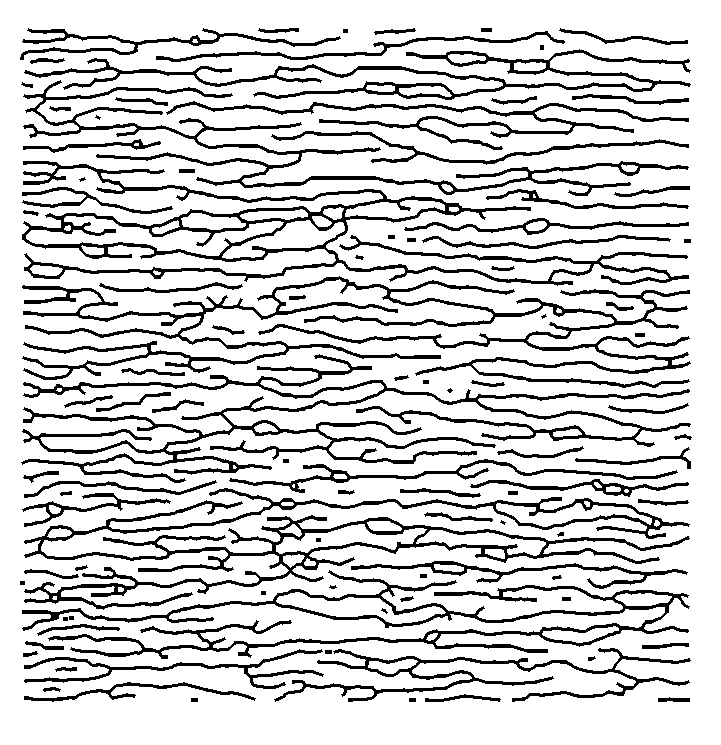

Supplement: Supplemental Information 3 [file peerj-07-7813-s003.zip › Supplemental-3/G-13-1.bmp]

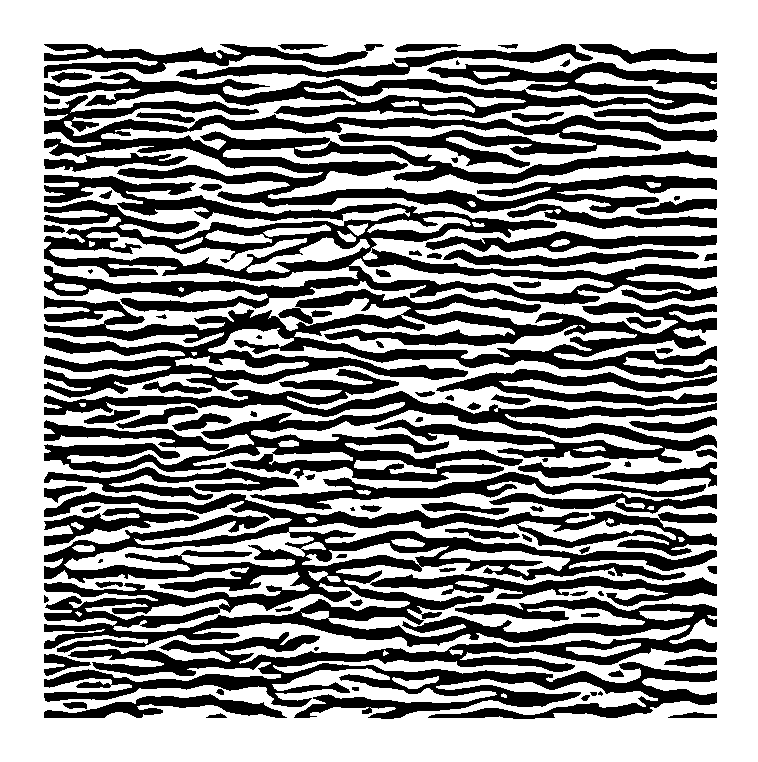

Supplement: Supplemental Information 3 [file peerj-07-7813-s003.zip › Supplemental-3/G-13.bmp]

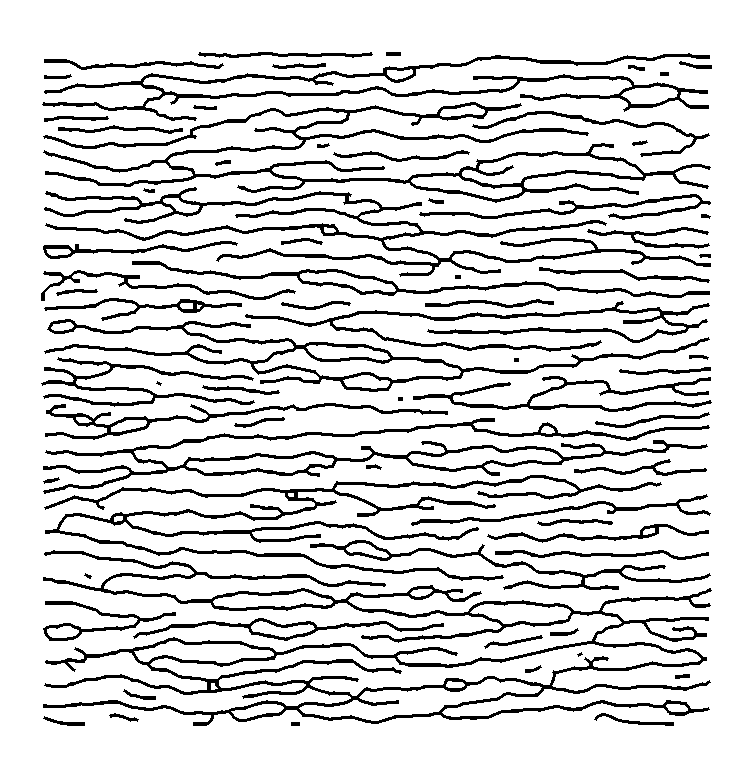

Supplement: Supplemental Information 3 [file peerj-07-7813-s003.zip › Supplemental-3/G-14-1.bmp]

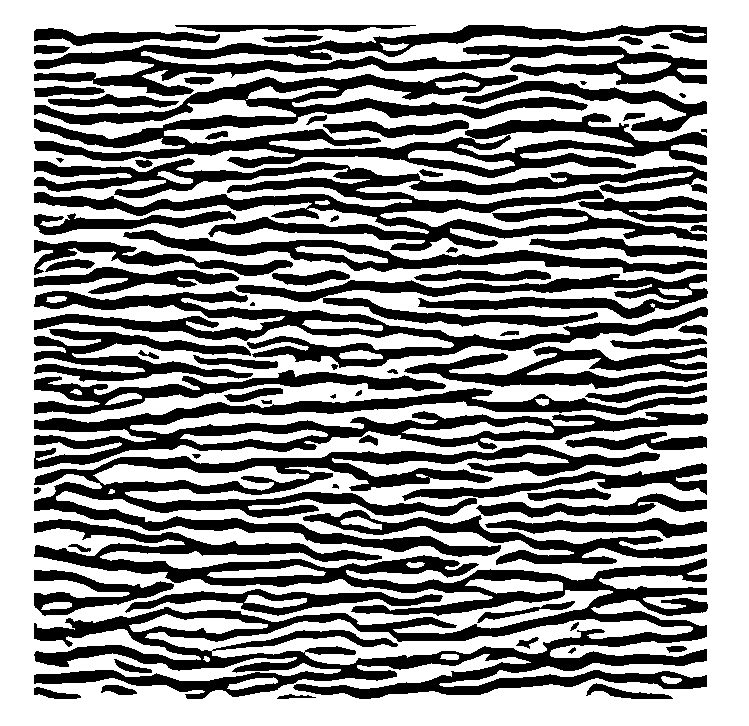

Supplement: Supplemental Information 3 [file peerj-07-7813-s003.zip › Supplemental-3/G-14.bmp]

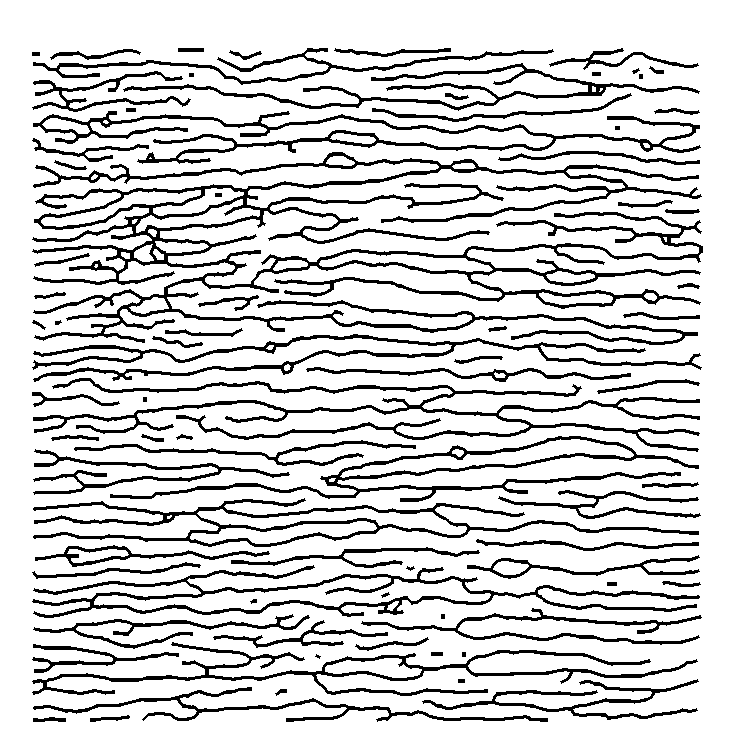

Supplement: Supplemental Information 3 [file peerj-07-7813-s003.zip › Supplemental-3/G-15-1.bmp]

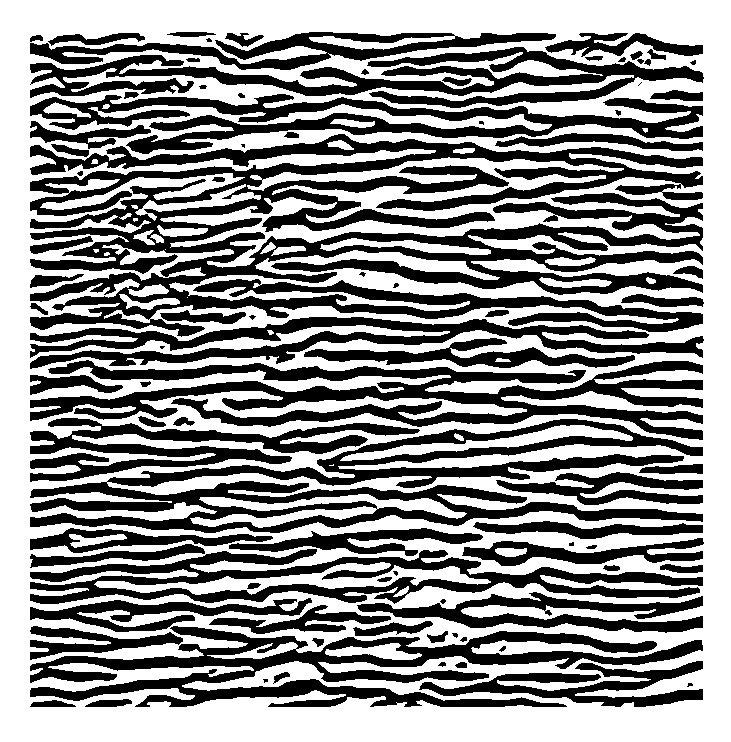

Supplement: Supplemental Information 3 [file peerj-07-7813-s003.zip › Supplemental-3/G-15.bmp]

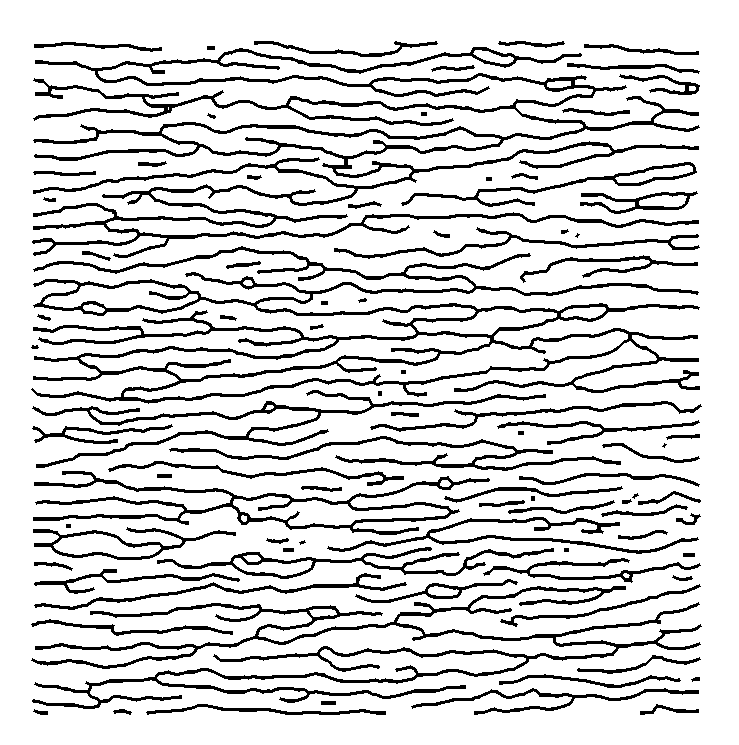

Supplement: Supplemental Information 3 [file peerj-07-7813-s003.zip › Supplemental-3/G-16-1.bmp]

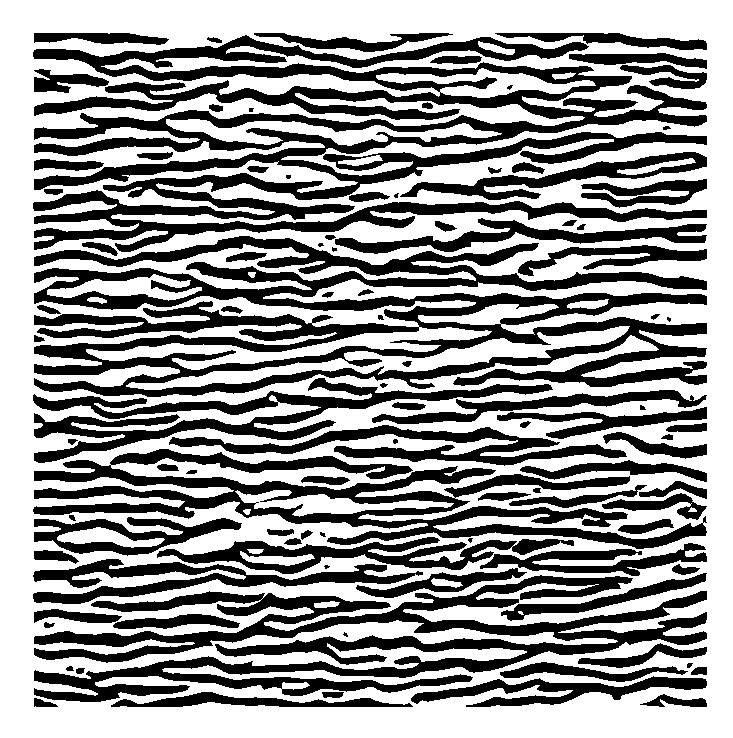

Supplement: Supplemental Information 3 [file peerj-07-7813-s003.zip › Supplemental-3/G-16.bmp]

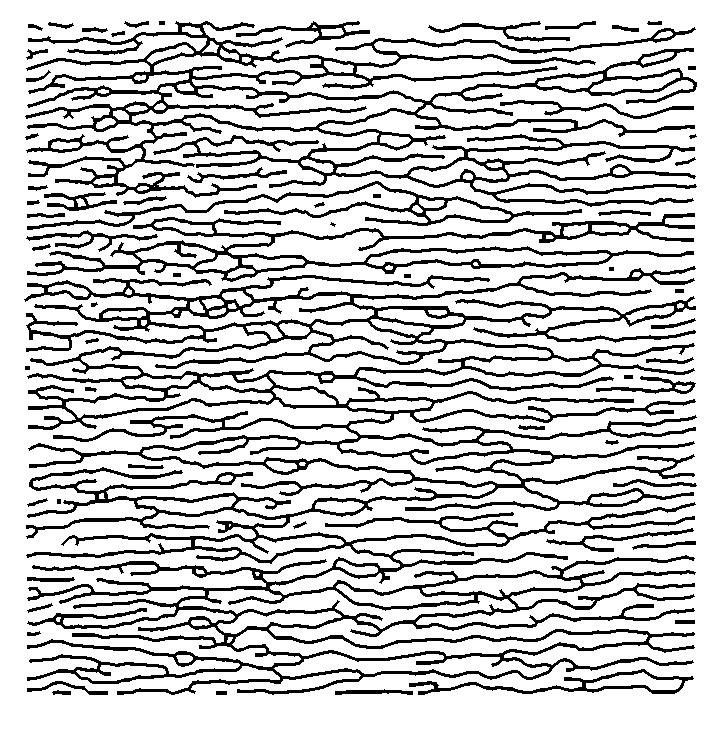

Supplement: Supplemental Information 3 [file peerj-07-7813-s003.zip › Supplemental-3/H-03-1.bmp]

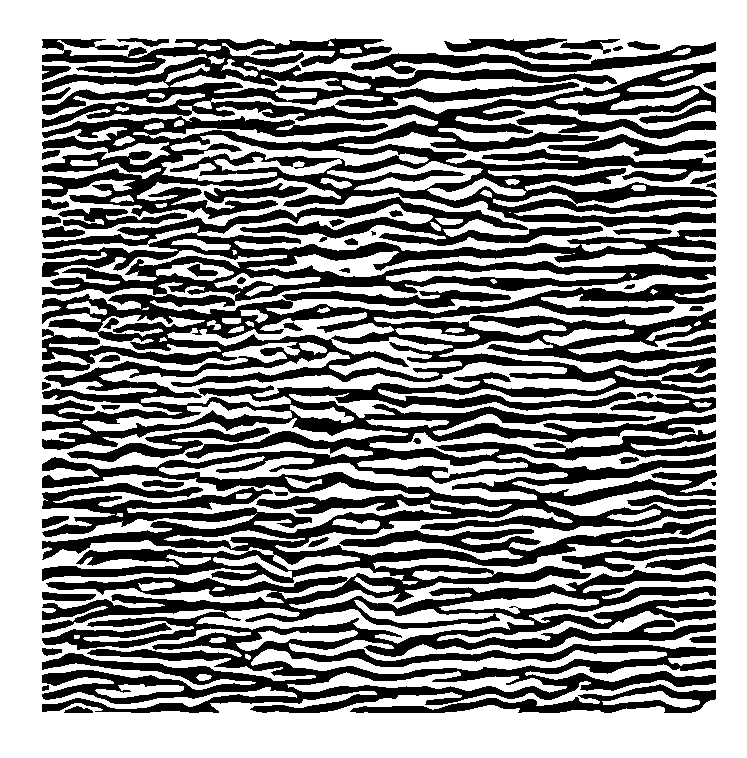

Supplement: Supplemental Information 3 [file peerj-07-7813-s003.zip › Supplemental-3/H-03.bmp]

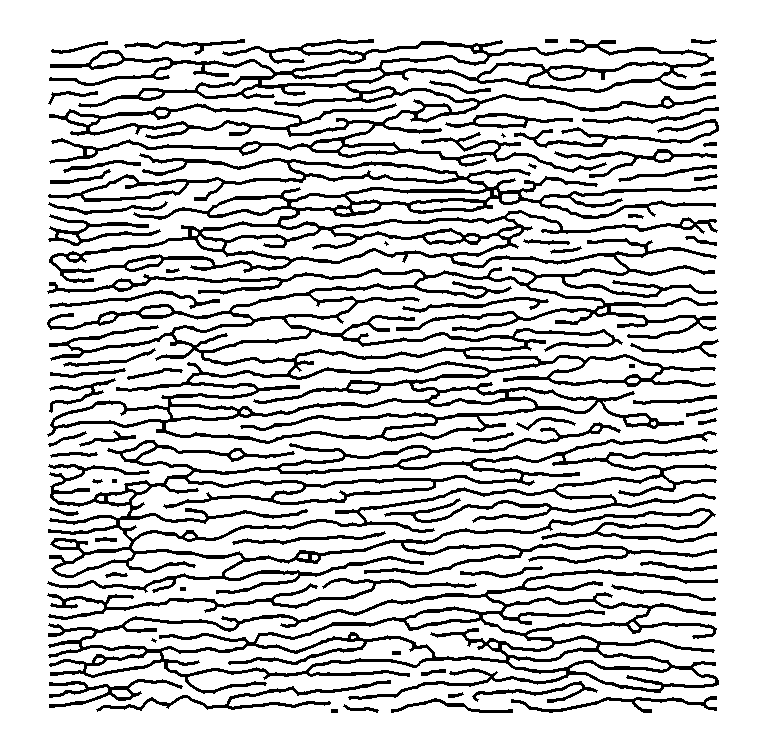

Supplement: Supplemental Information 3 [file peerj-07-7813-s003.zip › Supplemental-3/H-04-1.bmp]

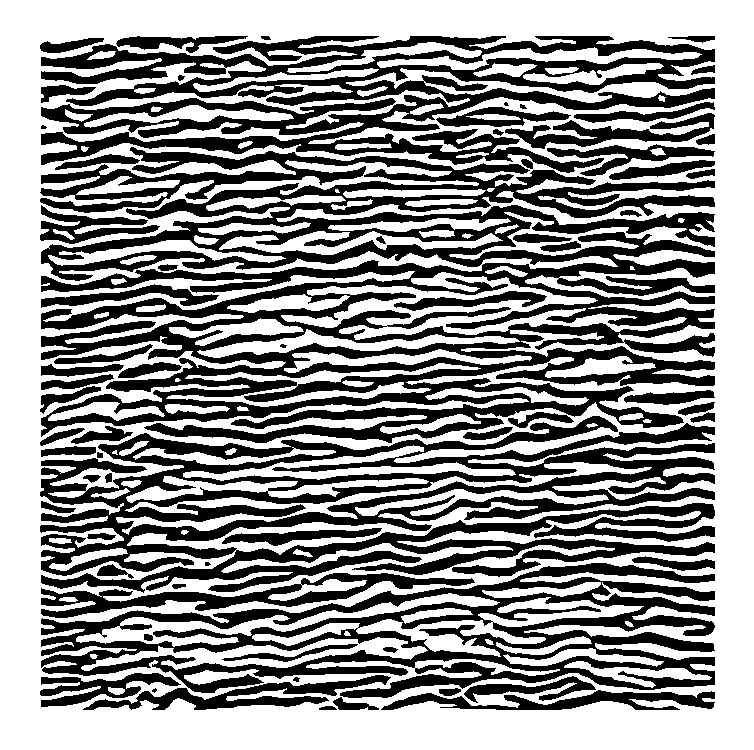

Supplement: Supplemental Information 3 [file peerj-07-7813-s003.zip › Supplemental-3/H-04.bmp]

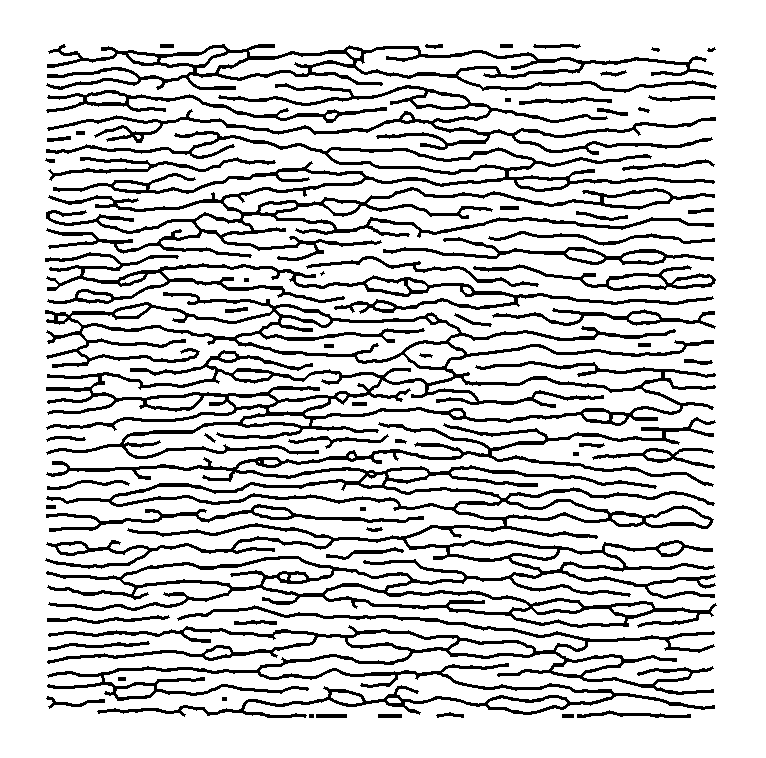

Supplement: Supplemental Information 3 [file peerj-07-7813-s003.zip › Supplemental-3/H-05-1.bmp]

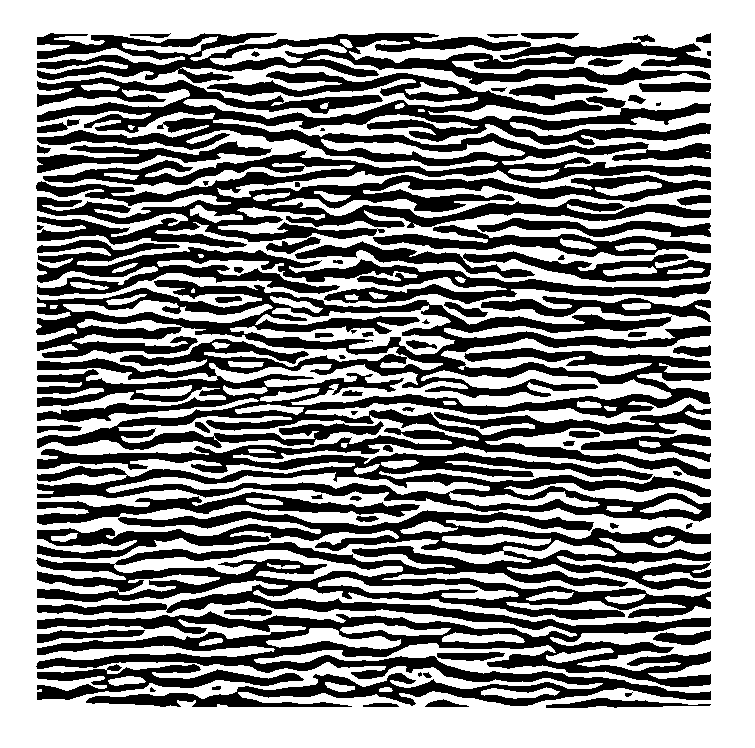

Supplement: Supplemental Information 3 [file peerj-07-7813-s003.zip › Supplemental-3/H-05.bmp]

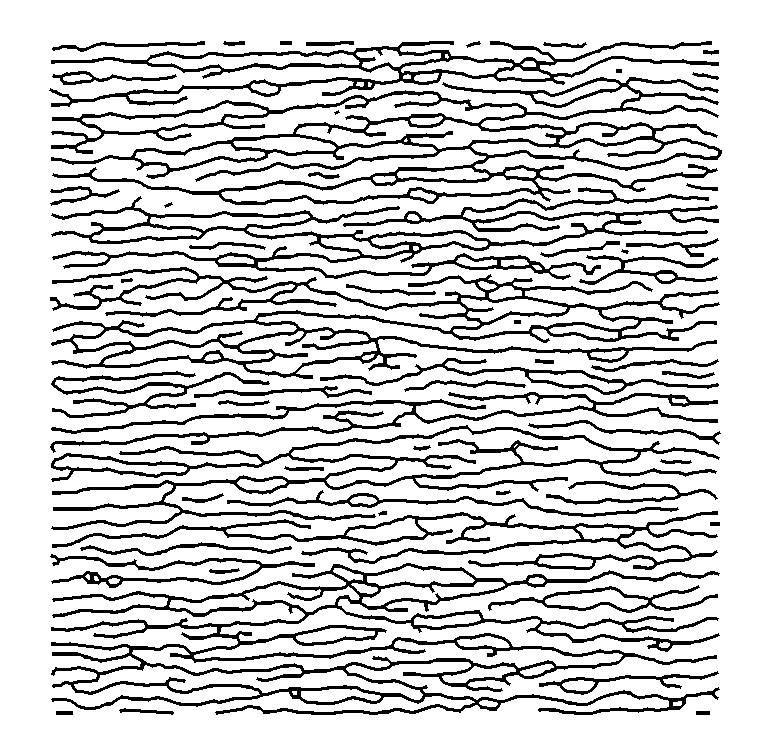

Supplement: Supplemental Information 3 [file peerj-07-7813-s003.zip › Supplemental-3/H-06-1.bmp]

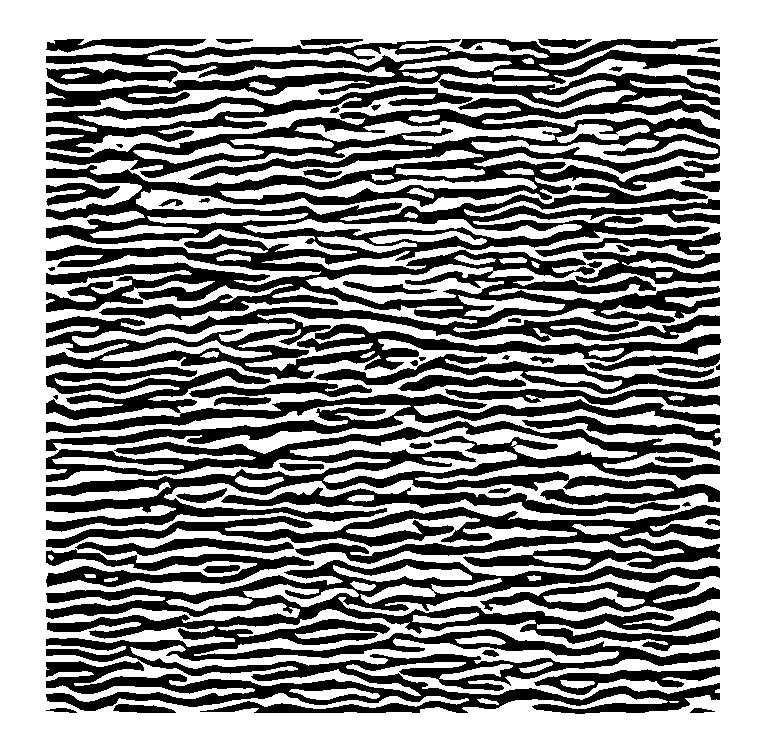

Supplement: Supplemental Information 3 [file peerj-07-7813-s003.zip › Supplemental-3/H-06.bmp]

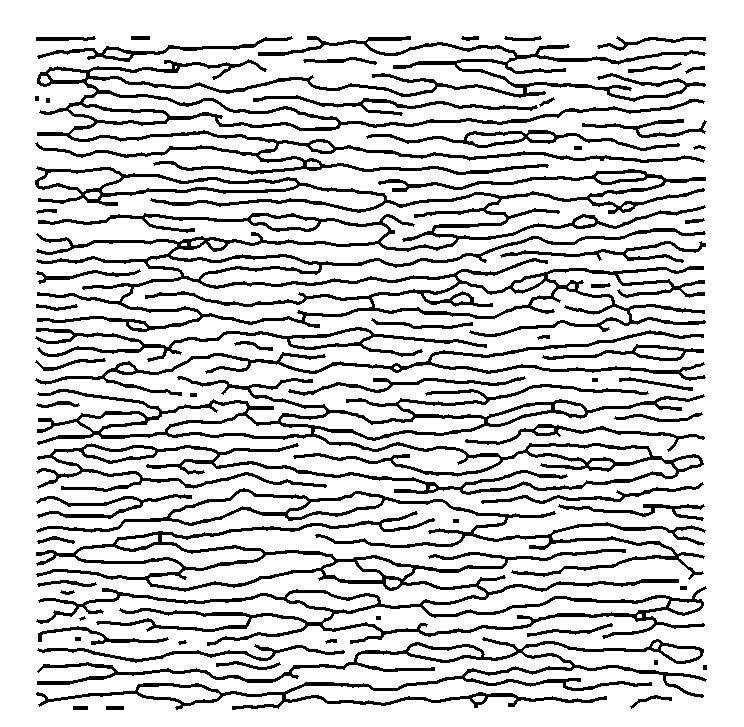

Supplement: Supplemental Information 3 [file peerj-07-7813-s003.zip › Supplemental-3/H-07-1.bmp]

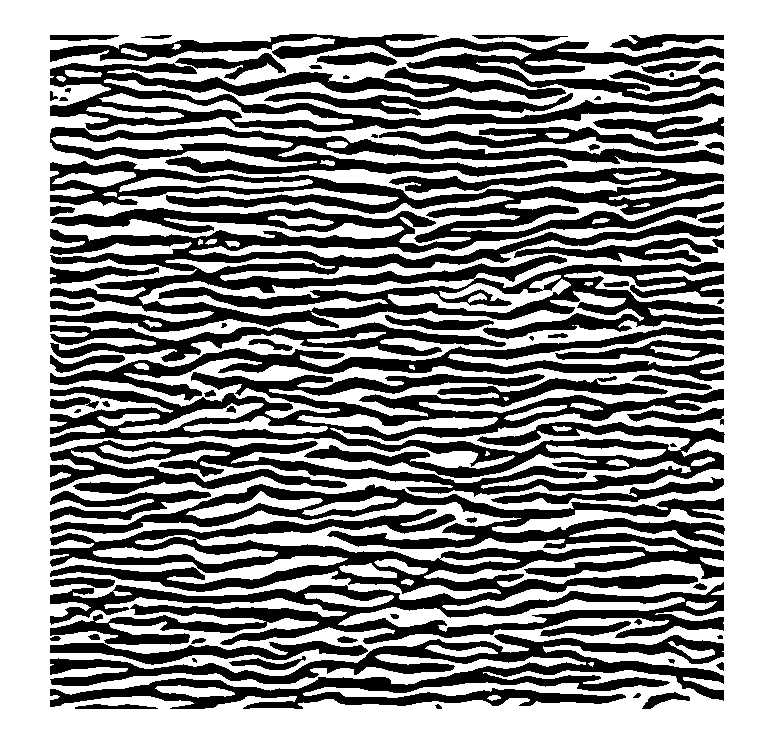

Supplement: Supplemental Information 3 [file peerj-07-7813-s003.zip › Supplemental-3/H-07.bmp]

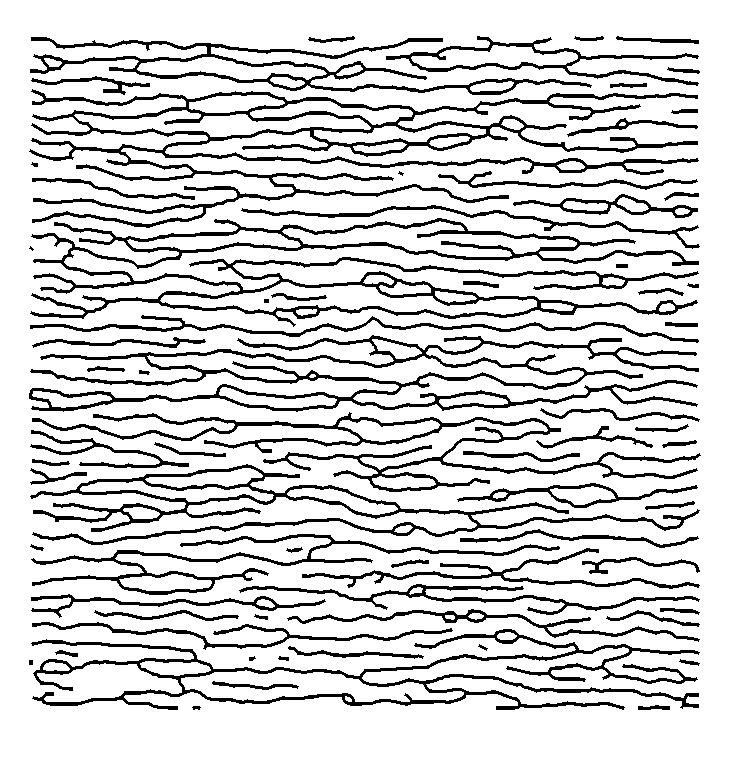

Supplement: Supplemental Information 3 [file peerj-07-7813-s003.zip › Supplemental-3/H-08-1.bmp]

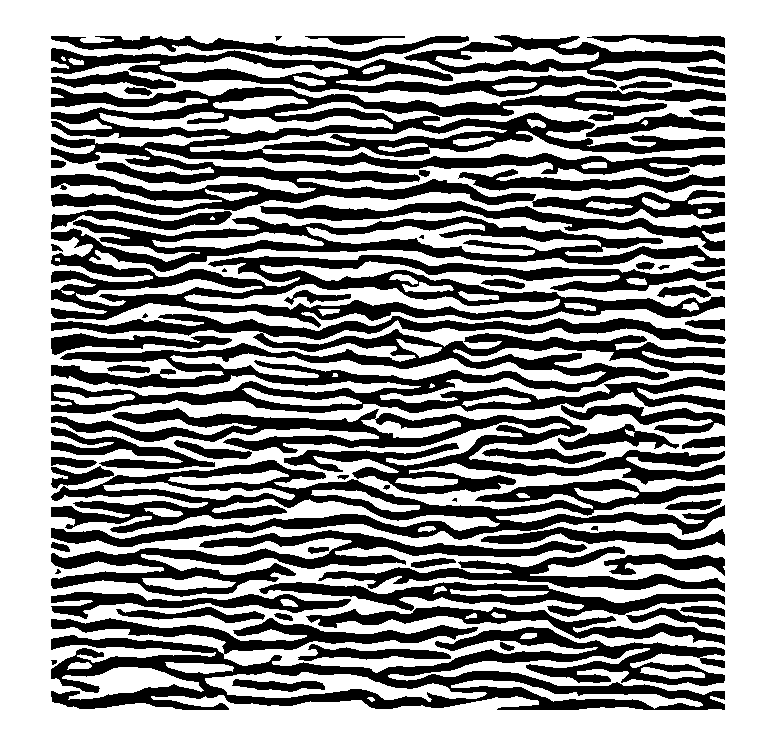

Supplement: Supplemental Information 3 [file peerj-07-7813-s003.zip › Supplemental-3/H-08.bmp]

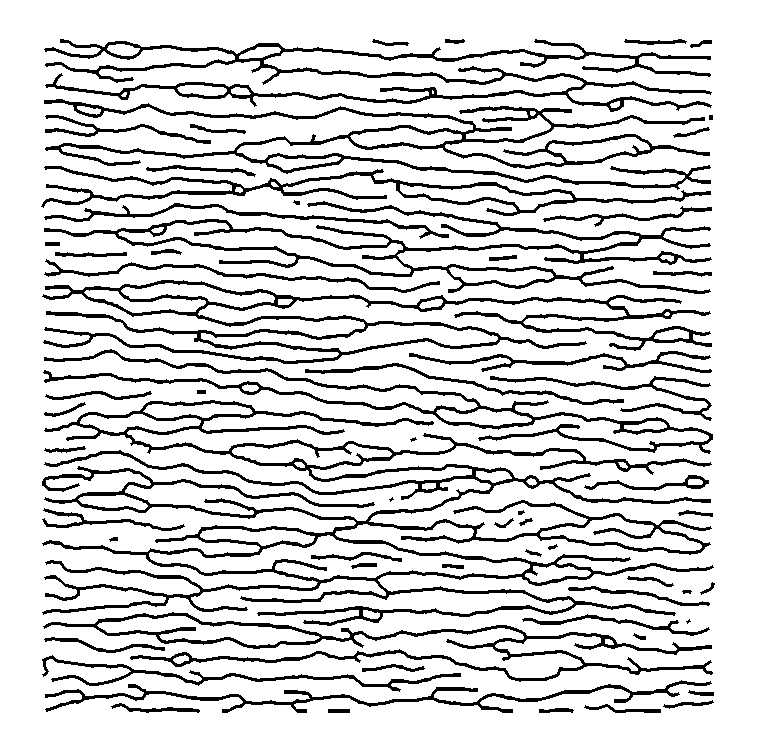

Supplement: Supplemental Information 3 [file peerj-07-7813-s003.zip › Supplemental-3/H-09-1.bmp]

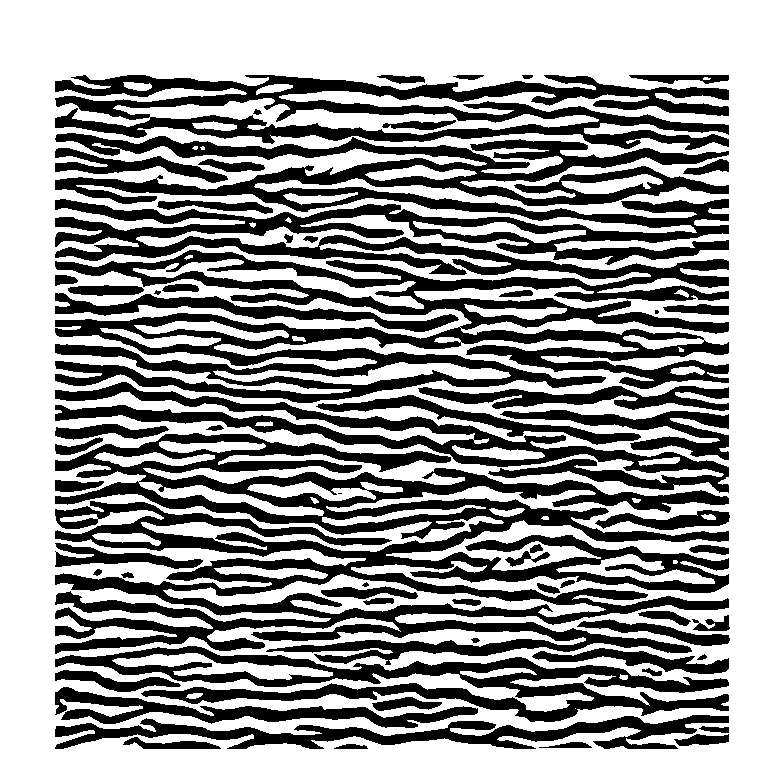

Supplement: Supplemental Information 3 [file peerj-07-7813-s003.zip › Supplemental-3/H-09.bmp]

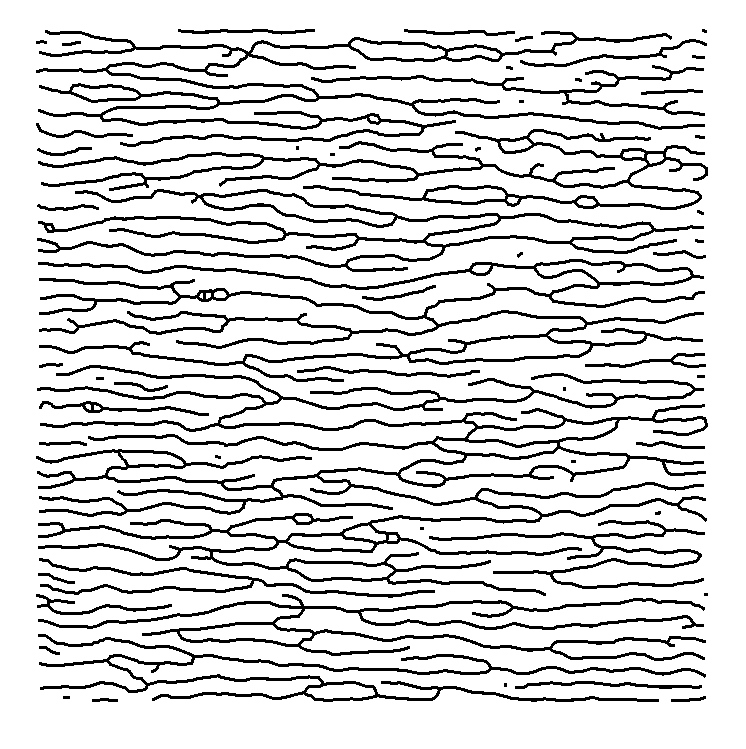

Supplement: Supplemental Information 3 [file peerj-07-7813-s003.zip › Supplemental-3/H-10-1.bmp]

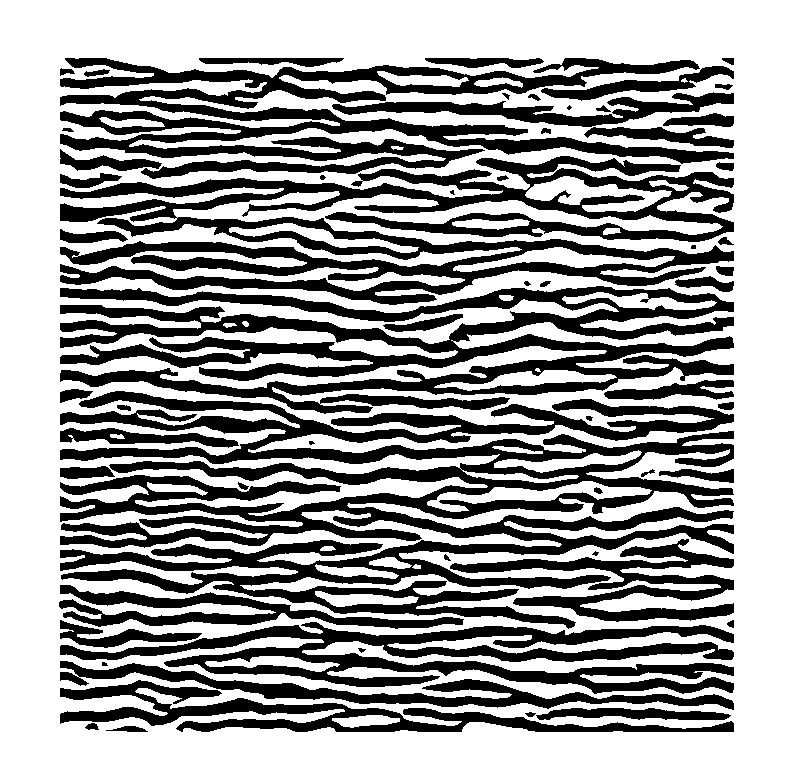

Supplement: Supplemental Information 3 [file peerj-07-7813-s003.zip › Supplemental-3/H-10.bmp]

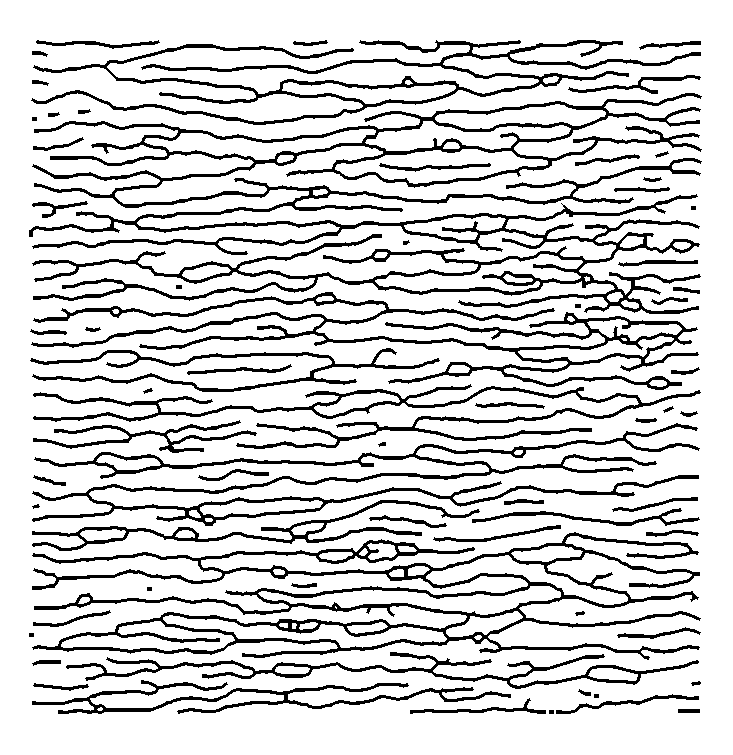

Supplement: Supplemental Information 3 [file peerj-07-7813-s003.zip › Supplemental-3/H-11-1.bmp]

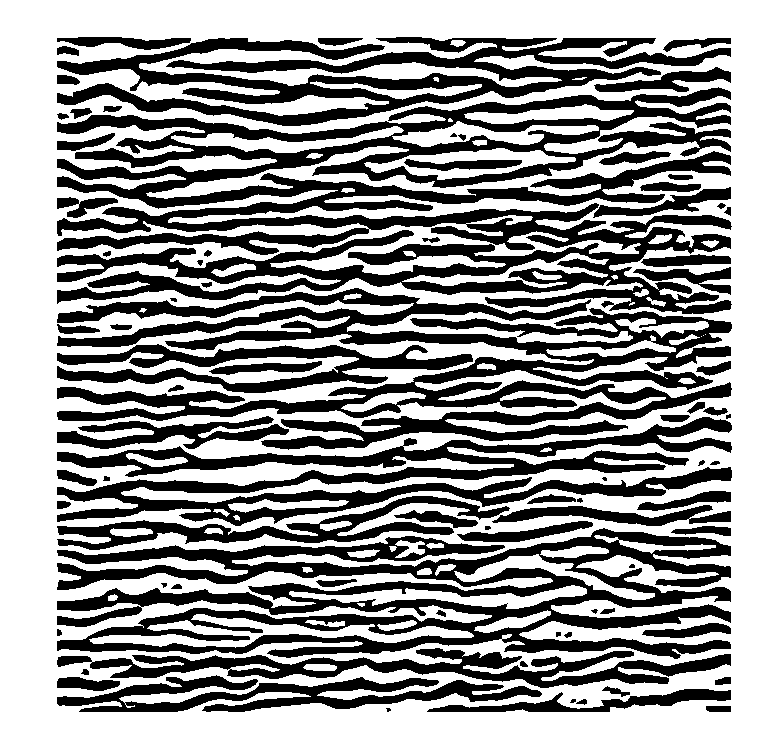

Supplement: Supplemental Information 3 [file peerj-07-7813-s003.zip › Supplemental-3/H-11.bmp]

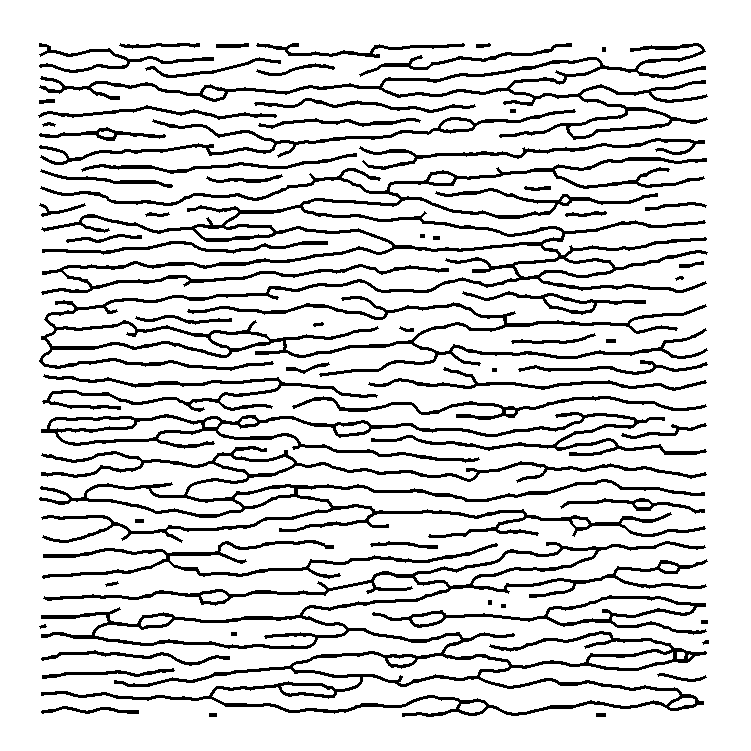

Supplement: Supplemental Information 3 [file peerj-07-7813-s003.zip › Supplemental-3/H-12-1.bmp]

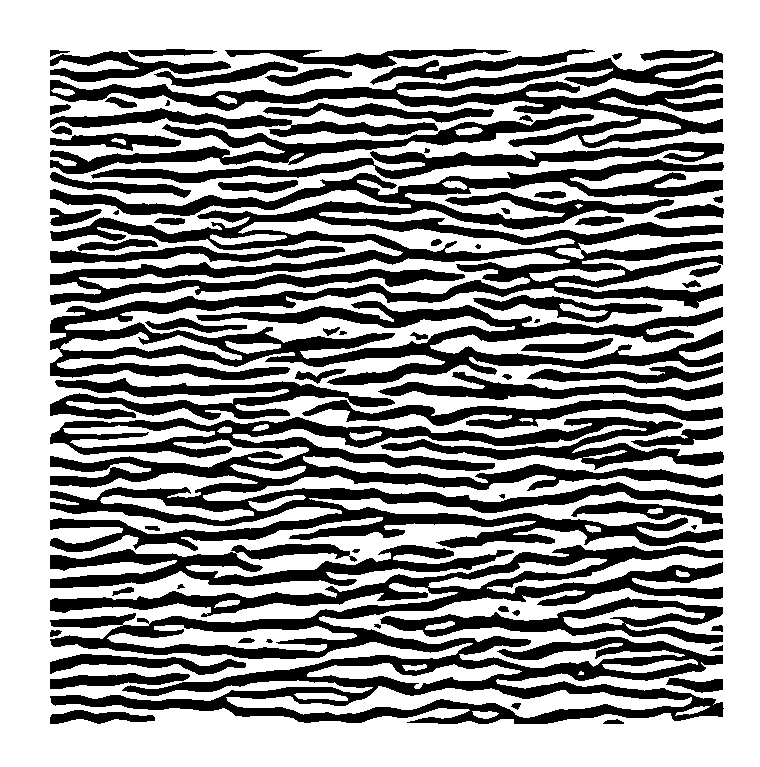

Supplement: Supplemental Information 3 [file peerj-07-7813-s003.zip › Supplemental-3/H-12.bmp]

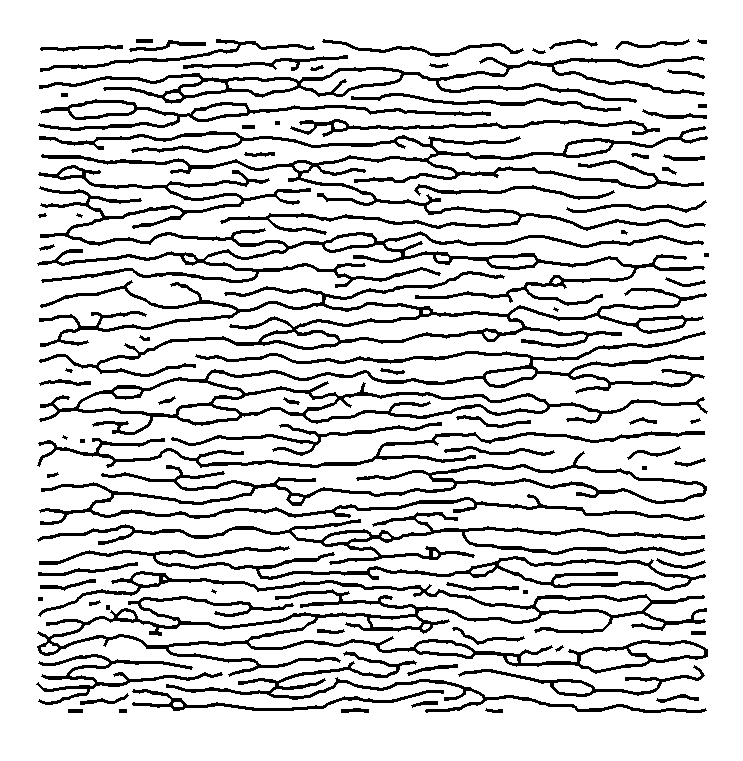

Supplement: Supplemental Information 3 [file peerj-07-7813-s003.zip › Supplemental-3/H-13-1.bmp]

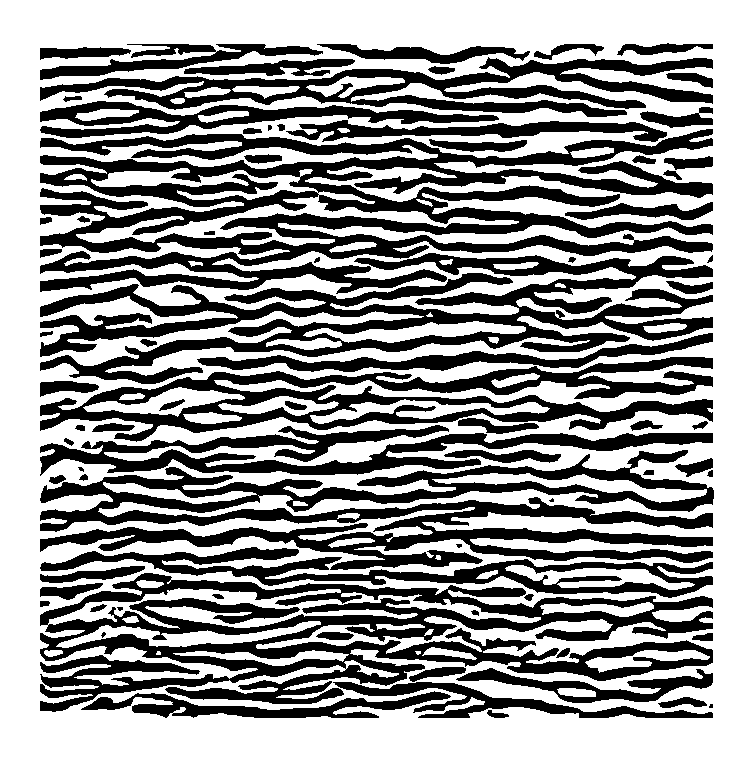

Supplement: Supplemental Information 3 [file peerj-07-7813-s003.zip › Supplemental-3/H-13.bmp]

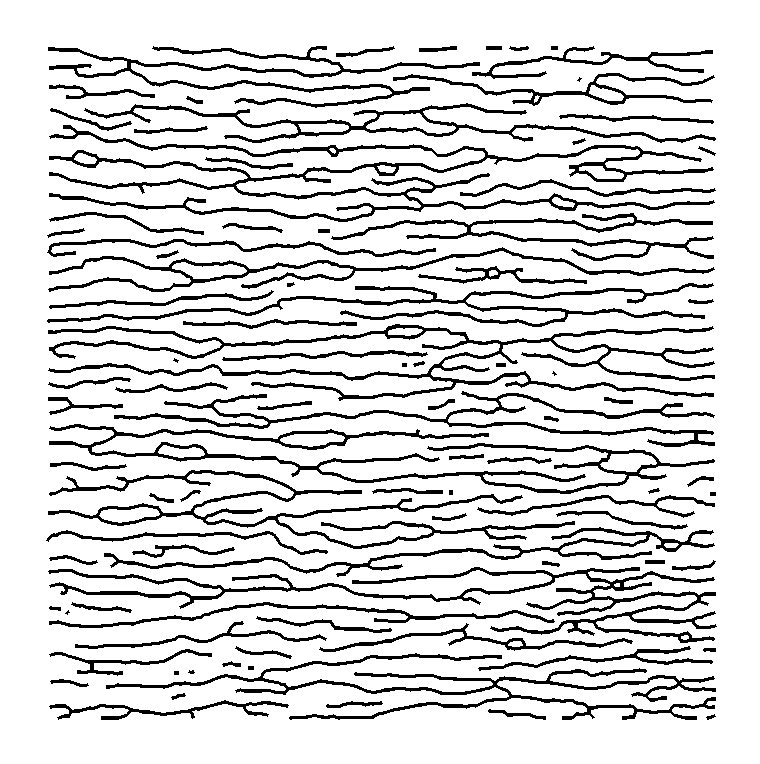

Supplement: Supplemental Information 3 [file peerj-07-7813-s003.zip › Supplemental-3/H-14-1.bmp]

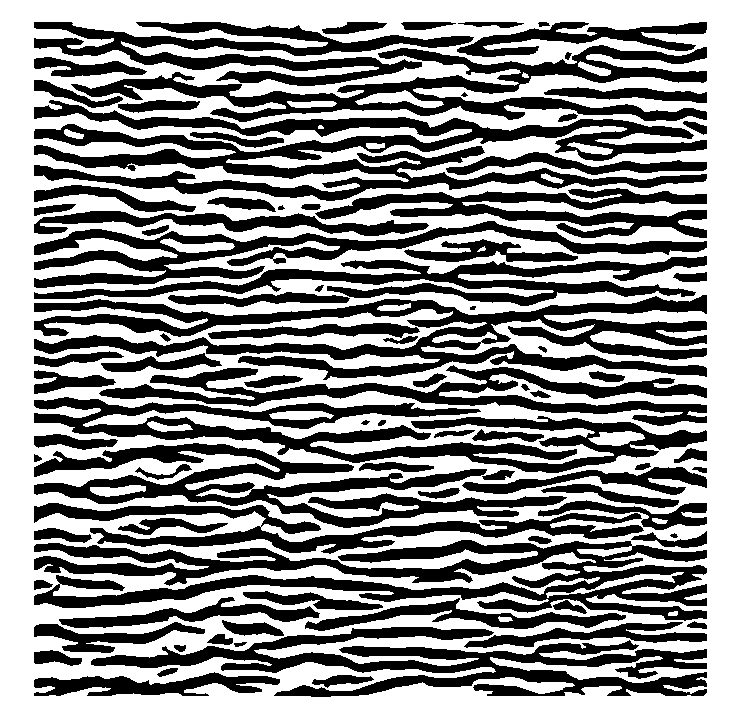

Supplement: Supplemental Information 3 [file peerj-07-7813-s003.zip › Supplemental-3/H-14.bmp]

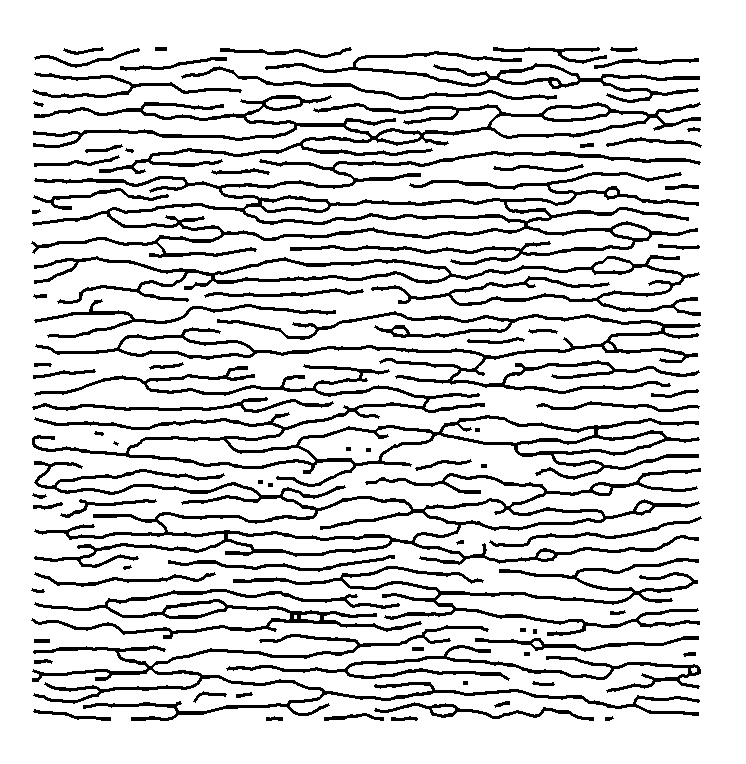

Supplement: Supplemental Information 3 [file peerj-07-7813-s003.zip › Supplemental-3/H-15-1.bmp]

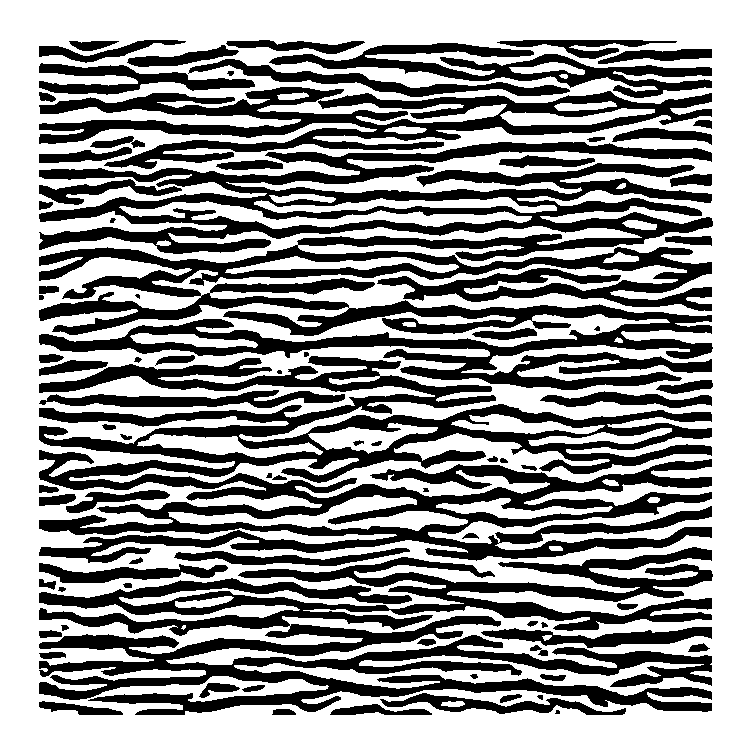

Supplement: Supplemental Information 3 [file peerj-07-7813-s003.zip › Supplemental-3/H-15.bmp]

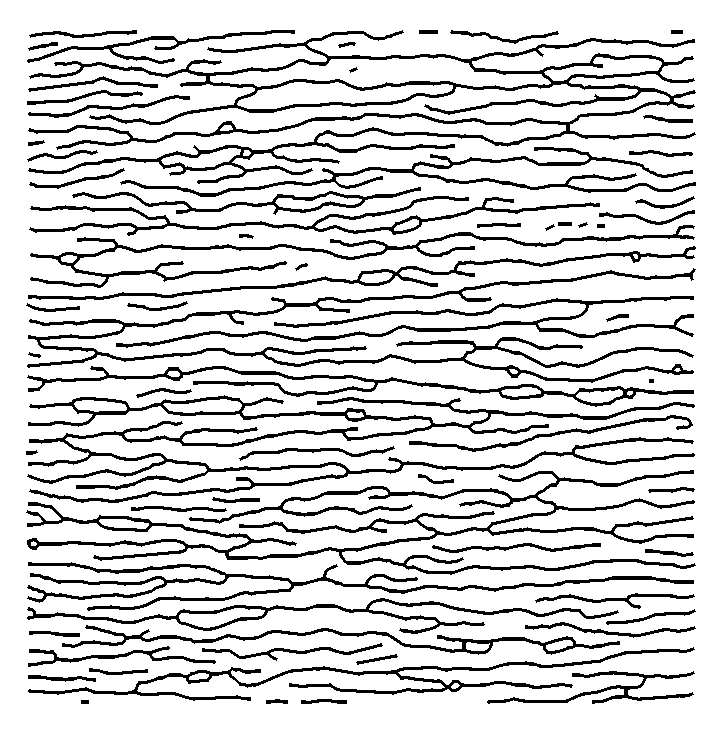

Supplement: Supplemental Information 3 [file peerj-07-7813-s003.zip › Supplemental-3/H-16-1.bmp]

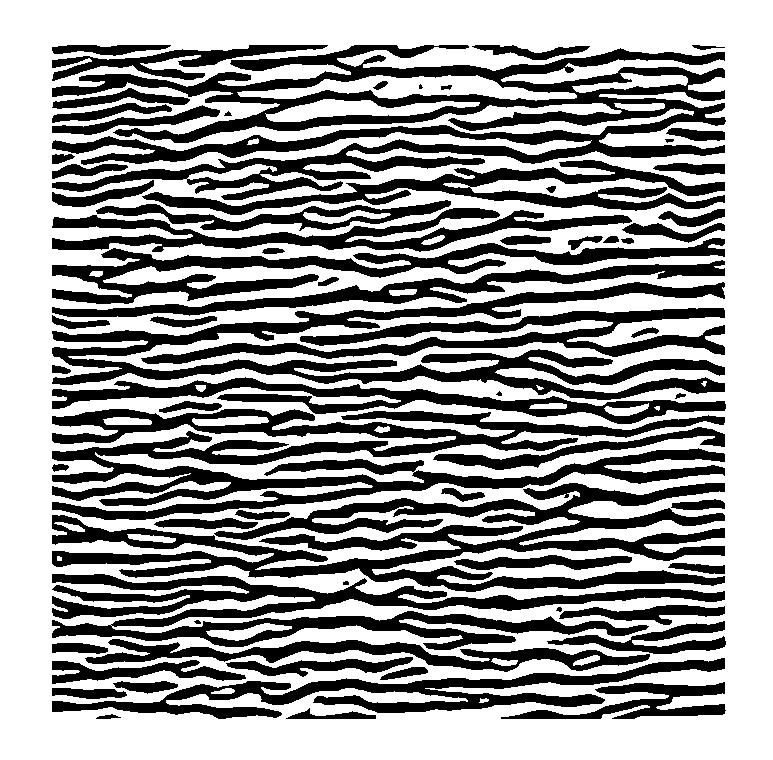

Supplement: Supplemental Information 3 [file peerj-07-7813-s003.zip › Supplemental-3/H-16.bmp]

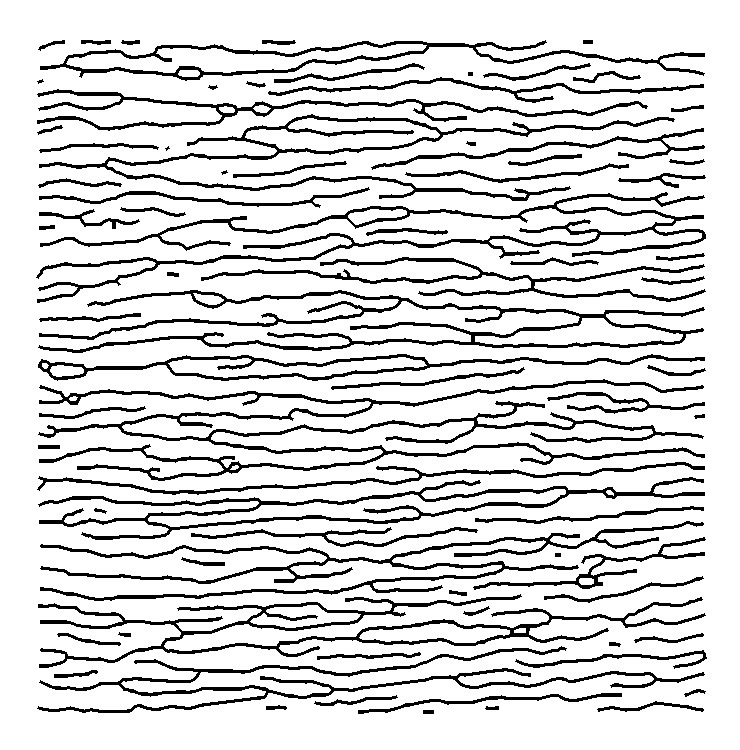

Supplement: Supplemental Information 3 [file peerj-07-7813-s003.zip › Supplemental-3/H-17-1.bmp]

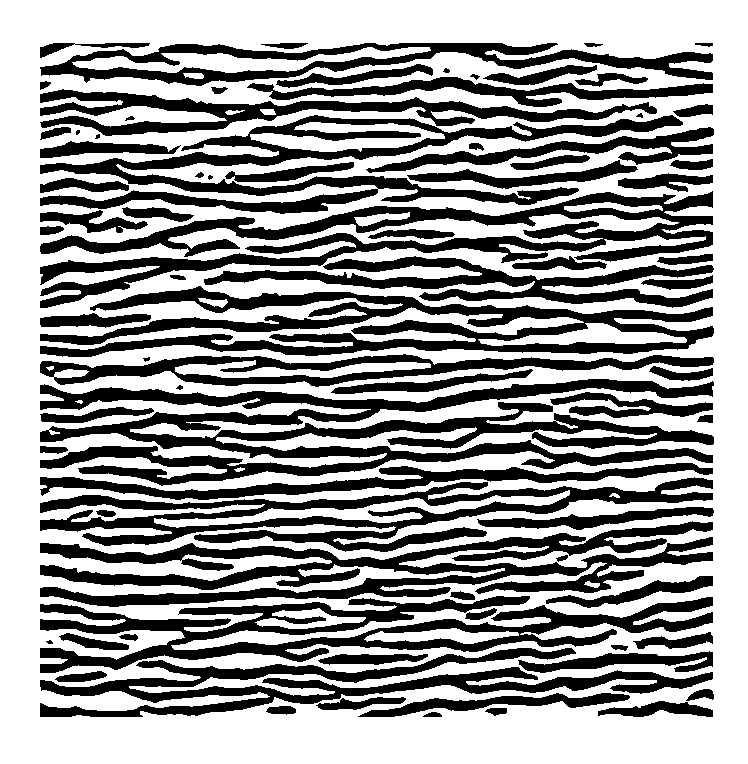

Supplement: Supplemental Information 3 [file peerj-07-7813-s003.zip › Supplemental-3/H-17.bmp]

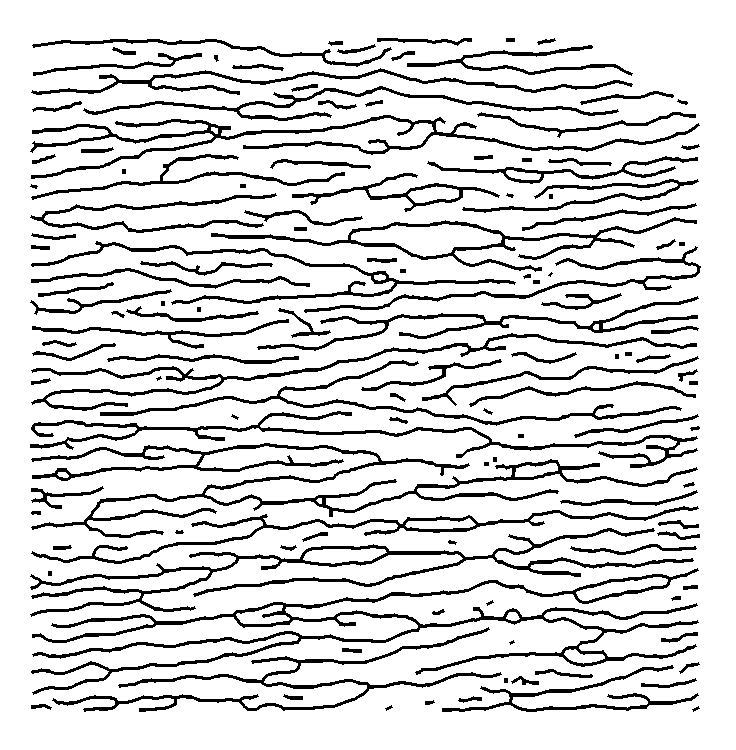

Supplement: Supplemental Information 3 [file peerj-07-7813-s003.zip › Supplemental-3/H-18-1.bmp]

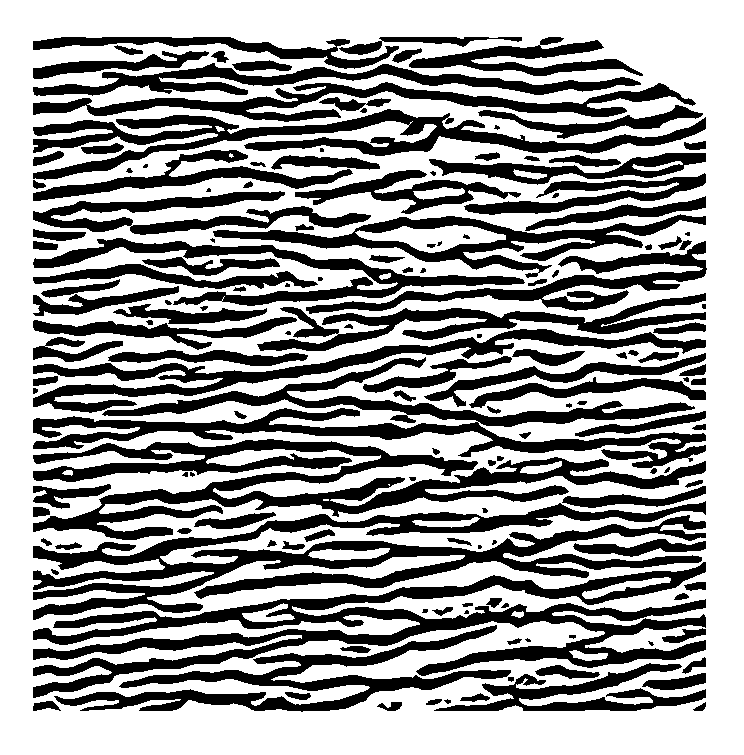

Supplement: Supplemental Information 3 [file peerj-07-7813-s003.zip › Supplemental-3/H-18.bmp]

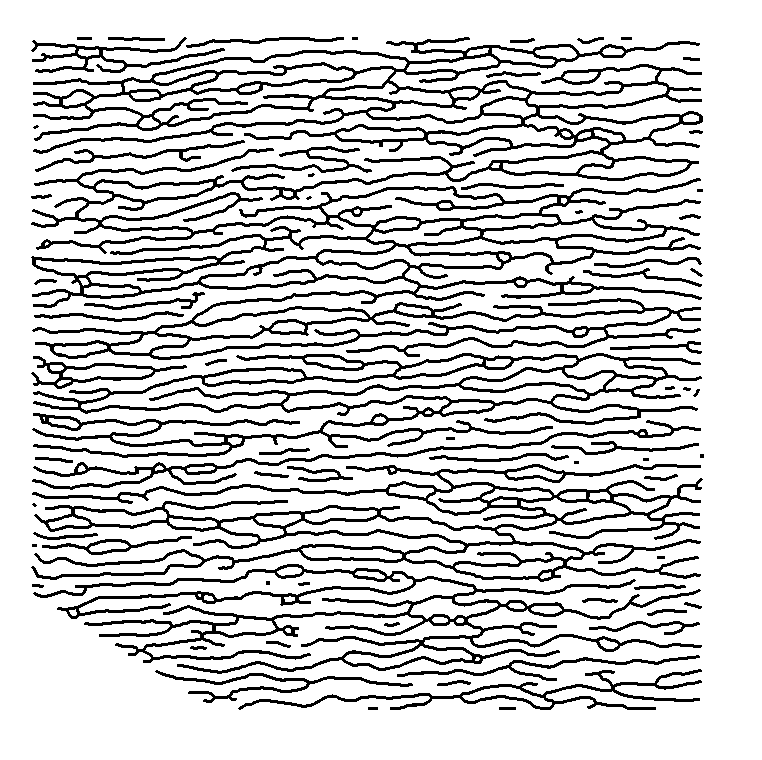

Supplement: Supplemental Information 3 [file peerj-07-7813-s003.zip › Supplemental-3/I-02-1.bmp]

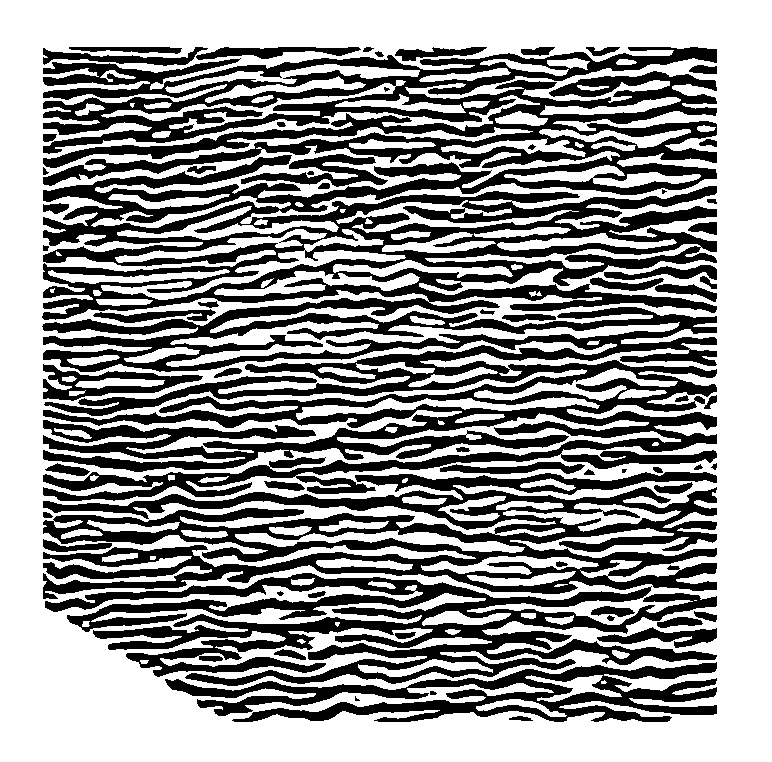

Supplement: Supplemental Information 3 [file peerj-07-7813-s003.zip › Supplemental-3/I-02.bmp]

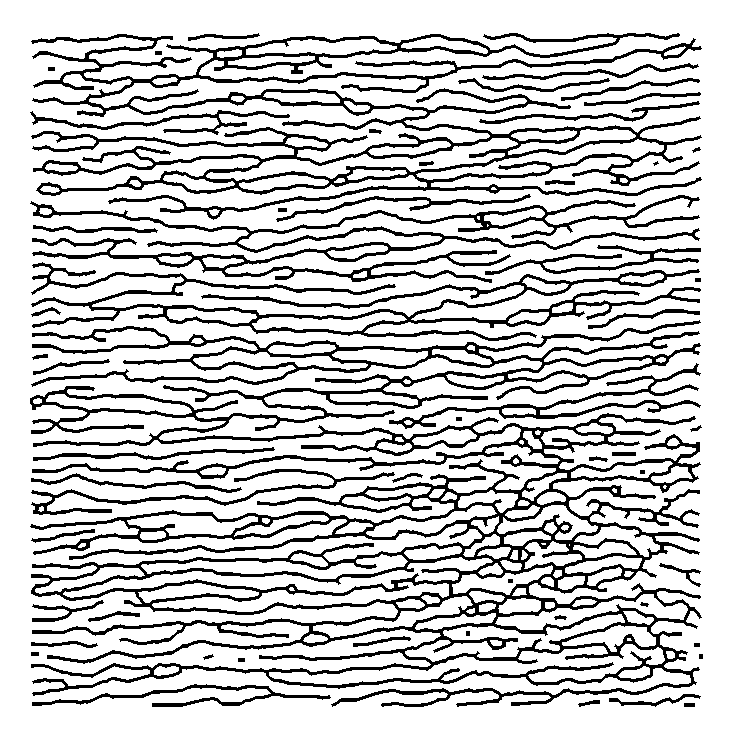

Supplement: Supplemental Information 3 [file peerj-07-7813-s003.zip › Supplemental-3/I-03-1.bmp]

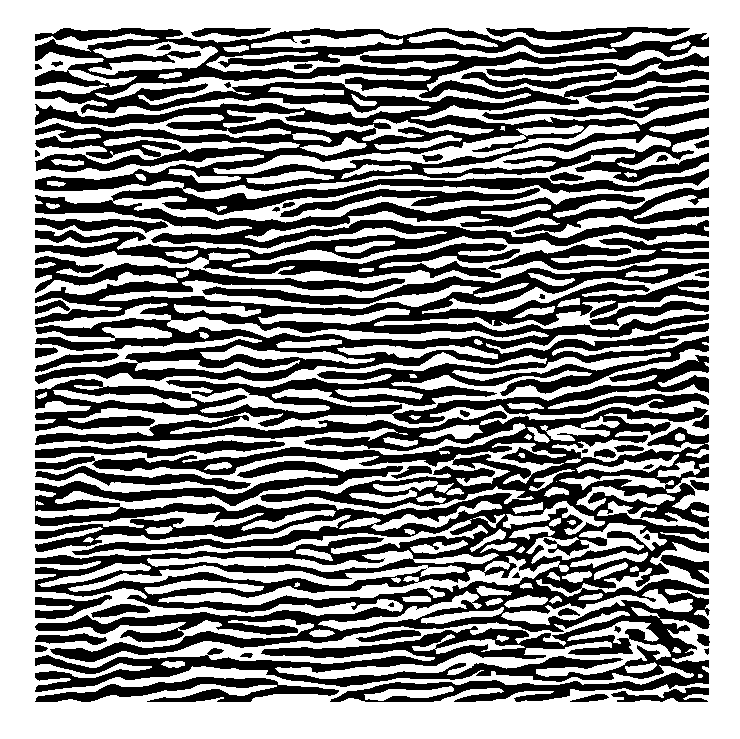

Supplement: Supplemental Information 3 [file peerj-07-7813-s003.zip › Supplemental-3/I-03.bmp]

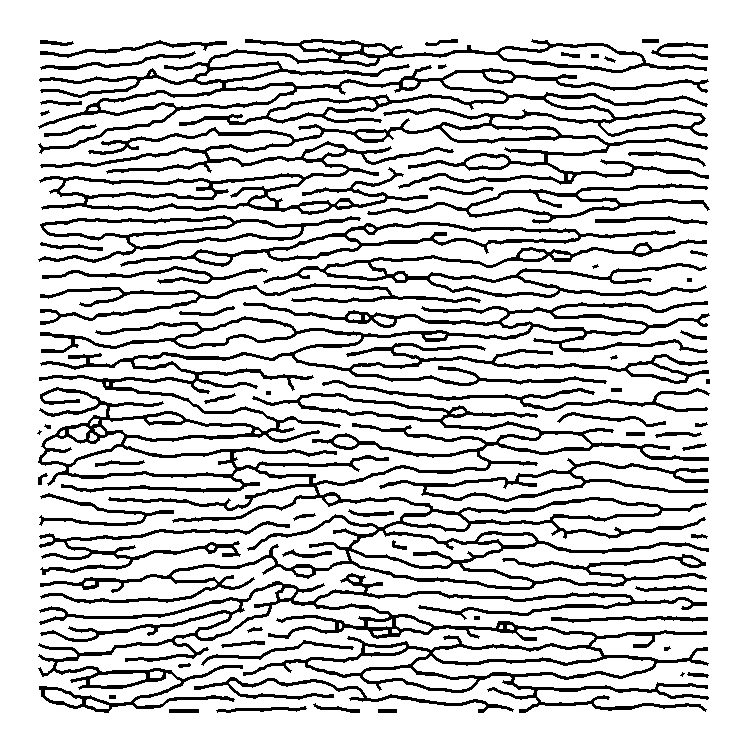

Supplement: Supplemental Information 3 [file peerj-07-7813-s003.zip › Supplemental-3/I-04-1.bmp]

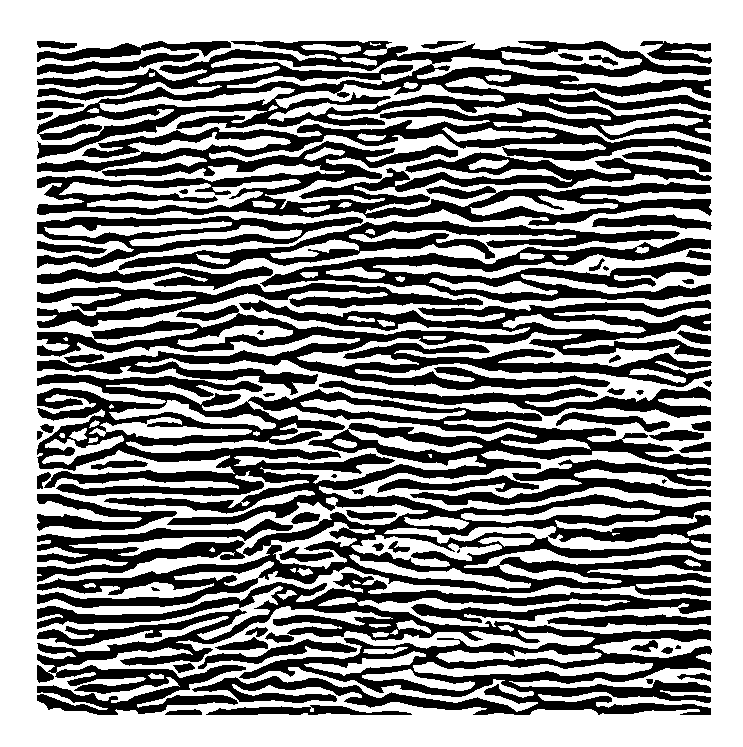

Supplement: Supplemental Information 3 [file peerj-07-7813-s003.zip › Supplemental-3/I-04.bmp]

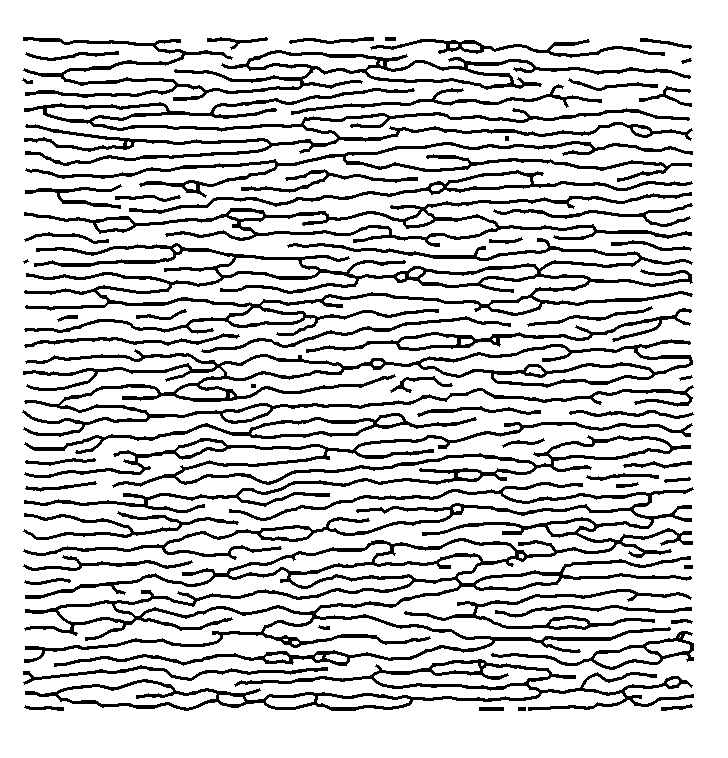

Supplement: Supplemental Information 3 [file peerj-07-7813-s003.zip › Supplemental-3/I-05-1.bmp]

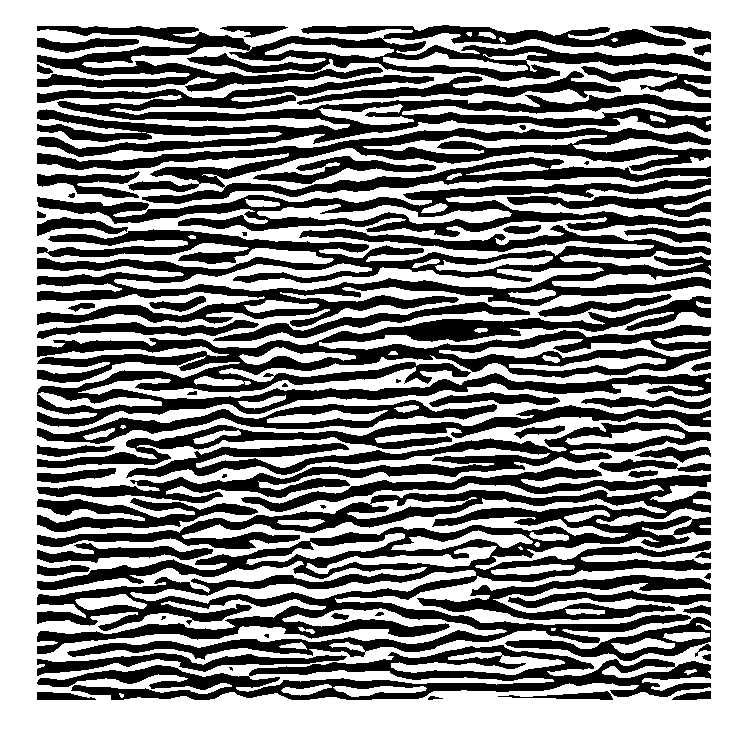

Supplement: Supplemental Information 3 [file peerj-07-7813-s003.zip › Supplemental-3/I-05.bmp]

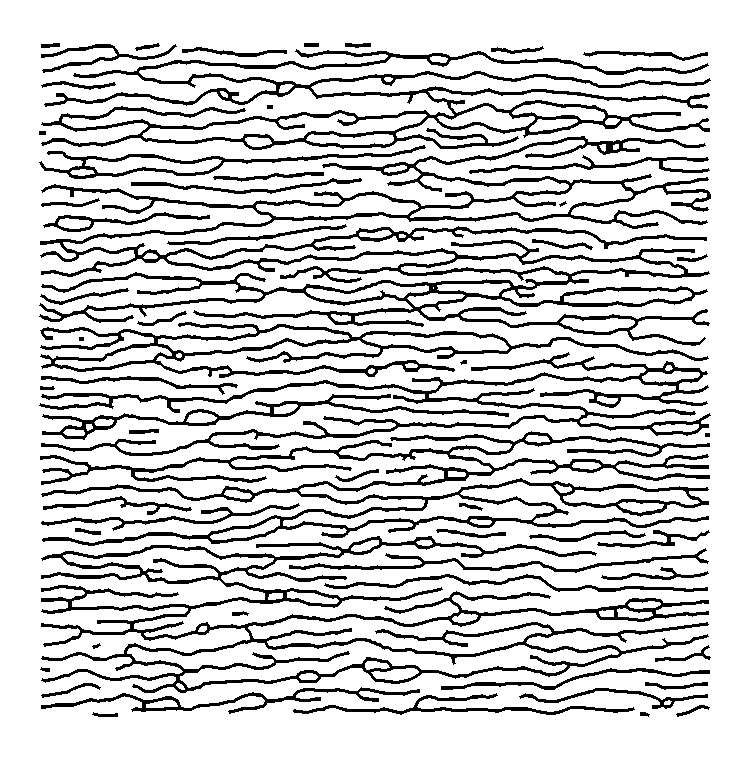

Supplement: Supplemental Information 3 [file peerj-07-7813-s003.zip › Supplemental-3/I-06-1.bmp]

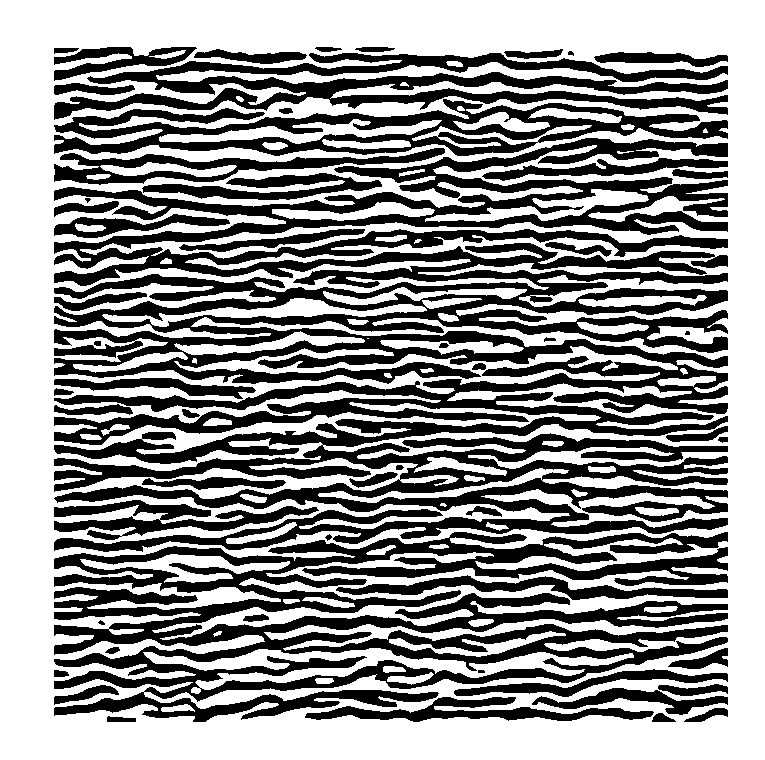

Supplement: Supplemental Information 3 [file peerj-07-7813-s003.zip › Supplemental-3/I-06.bmp]

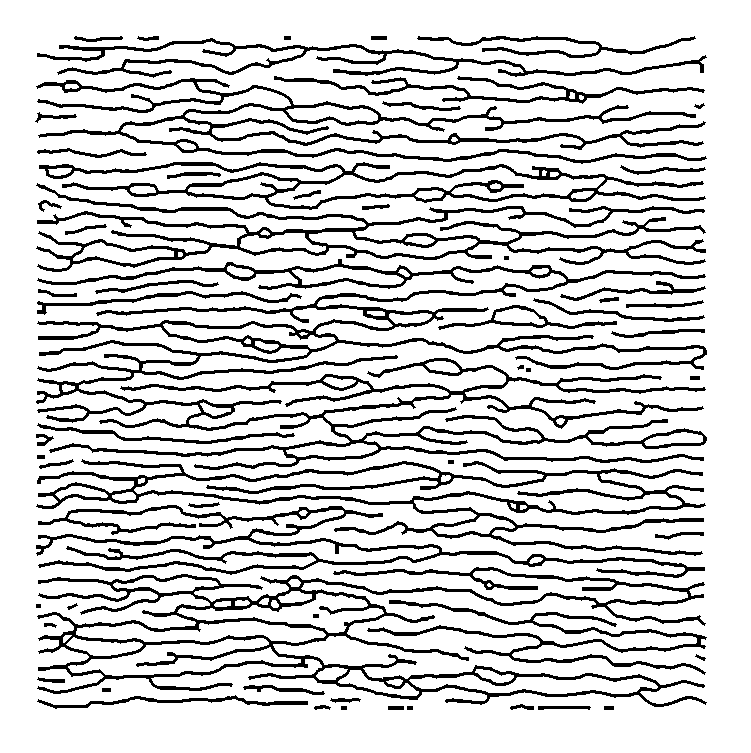

Supplement: Supplemental Information 3 [file peerj-07-7813-s003.zip › Supplemental-3/I-07-1.bmp]

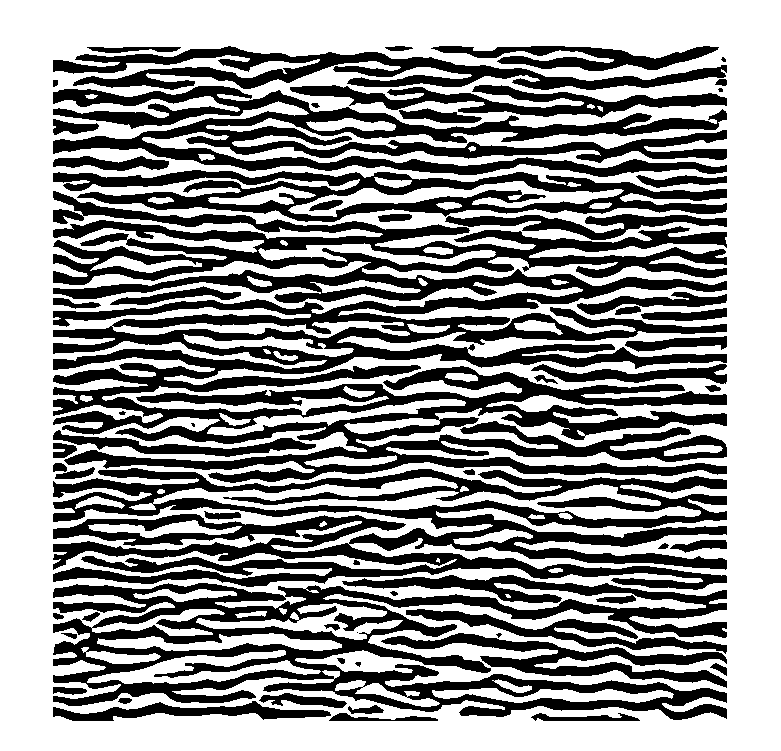

Supplement: Supplemental Information 3 [file peerj-07-7813-s003.zip › Supplemental-3/I-07.bmp]

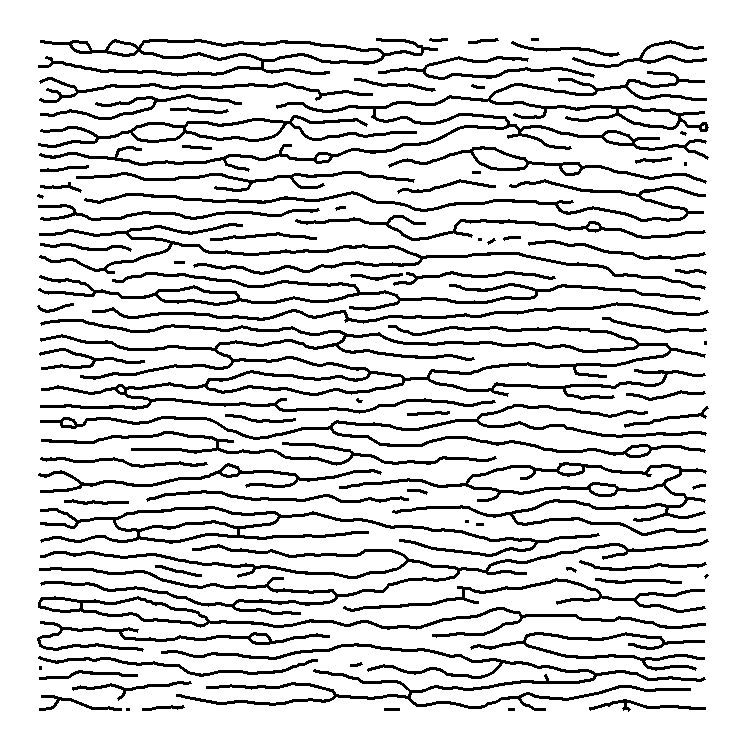

Supplement: Supplemental Information 3 [file peerj-07-7813-s003.zip › Supplemental-3/I-08-1.bmp]

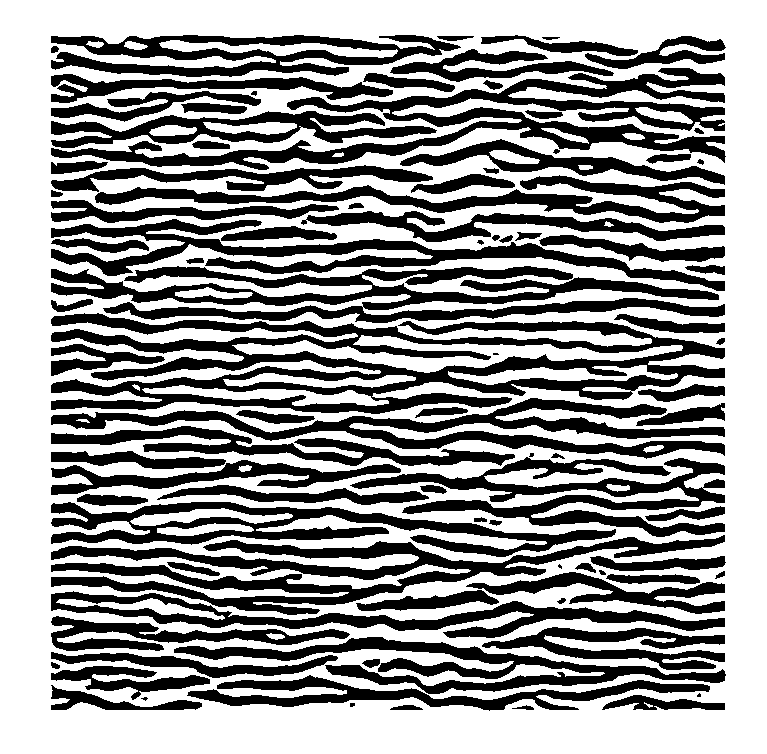

Supplement: Supplemental Information 3 [file peerj-07-7813-s003.zip › Supplemental-3/I-08.bmp]

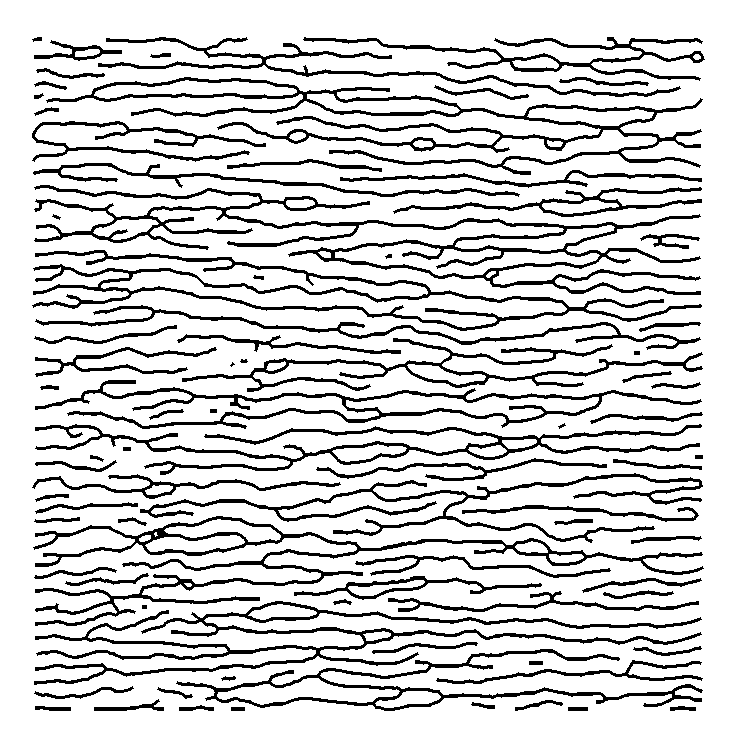

Supplement: Supplemental Information 3 [file peerj-07-7813-s003.zip › Supplemental-3/I-09-1.bmp]

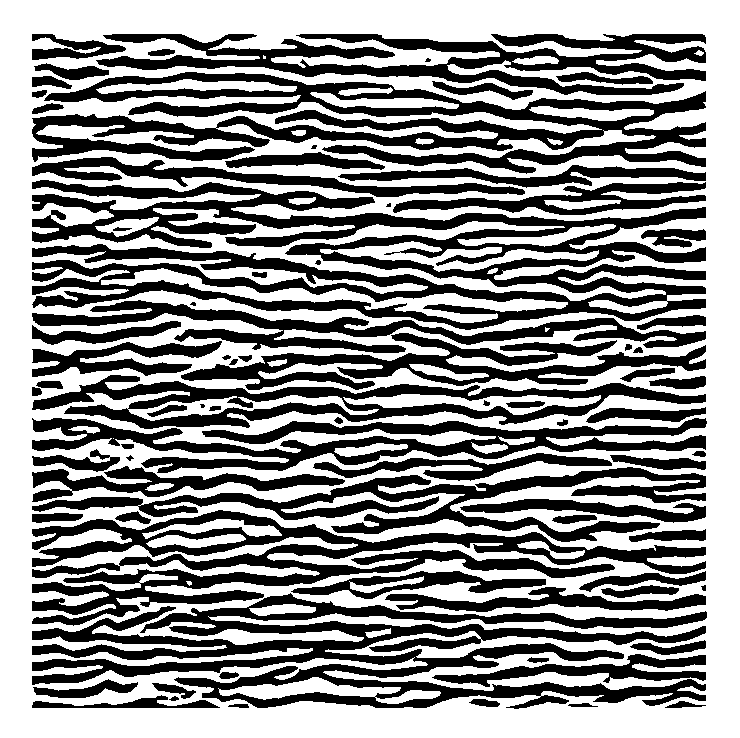

Supplement: Supplemental Information 3 [file peerj-07-7813-s003.zip › Supplemental-3/I-09.bmp]

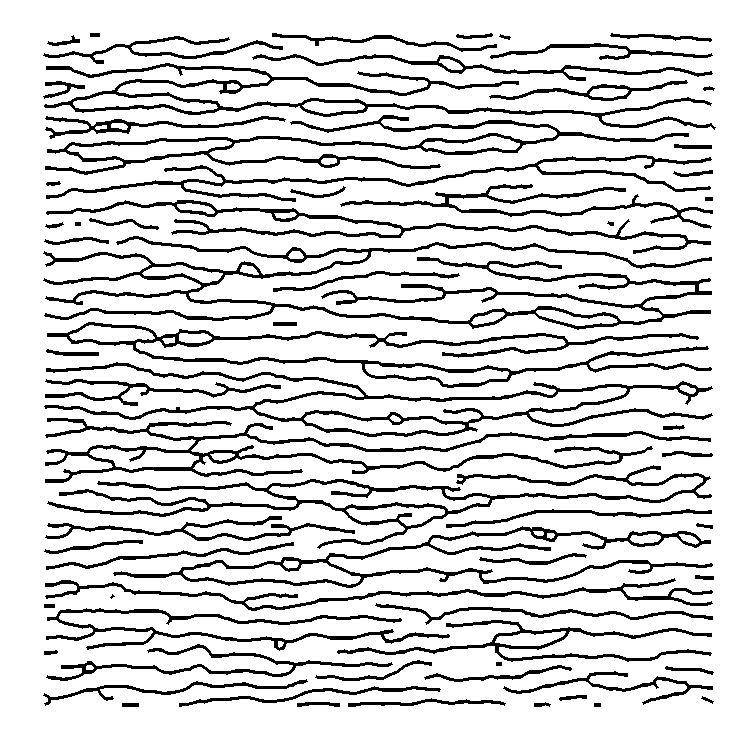

Supplement: Supplemental Information 3 [file peerj-07-7813-s003.zip › Supplemental-3/I-10-1.bmp]

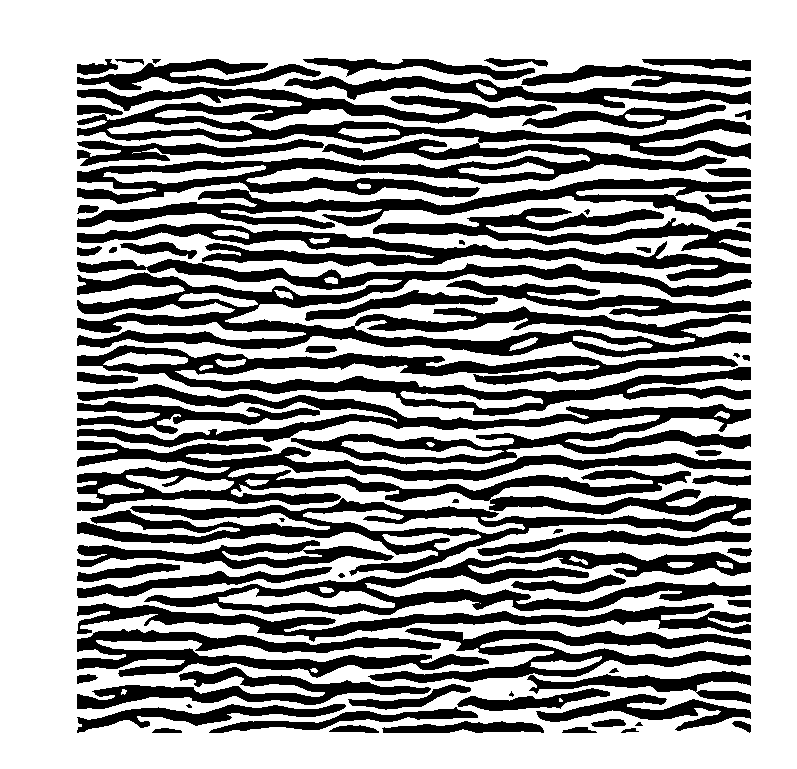

Supplement: Supplemental Information 3 [file peerj-07-7813-s003.zip › Supplemental-3/I-10.bmp]

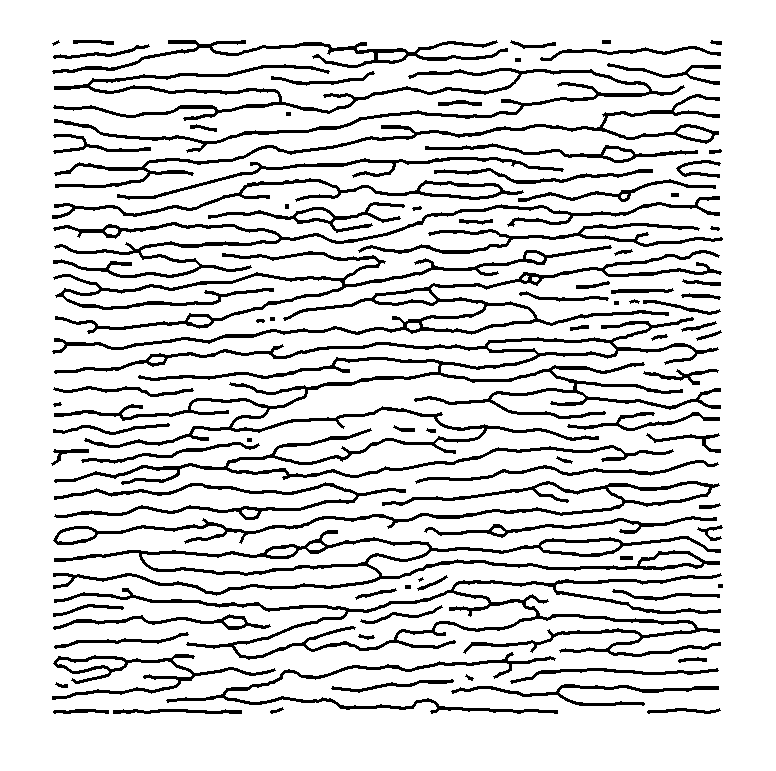

Supplement: Supplemental Information 3 [file peerj-07-7813-s003.zip › Supplemental-3/I-11-1.bmp]

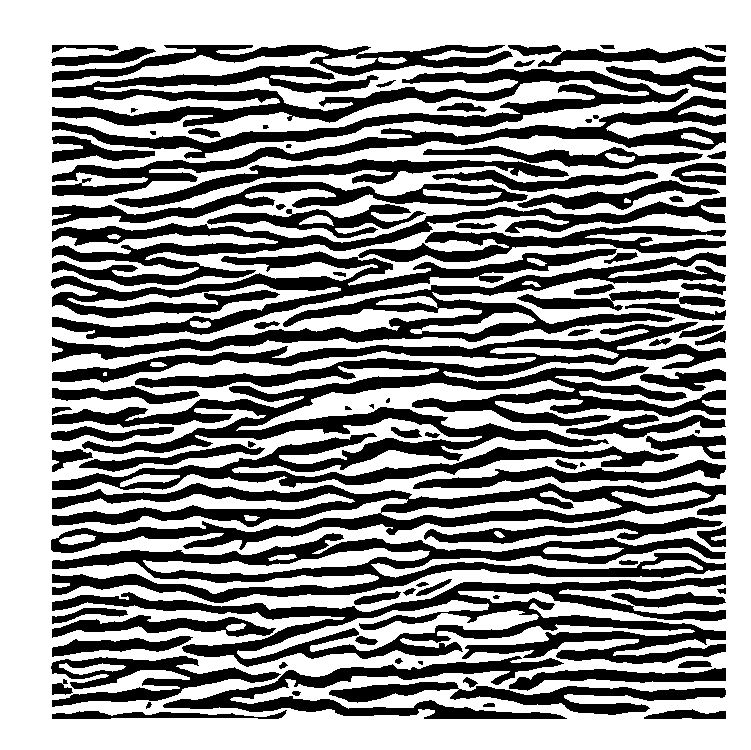

Supplement: Supplemental Information 3 [file peerj-07-7813-s003.zip › Supplemental-3/I-11.bmp]

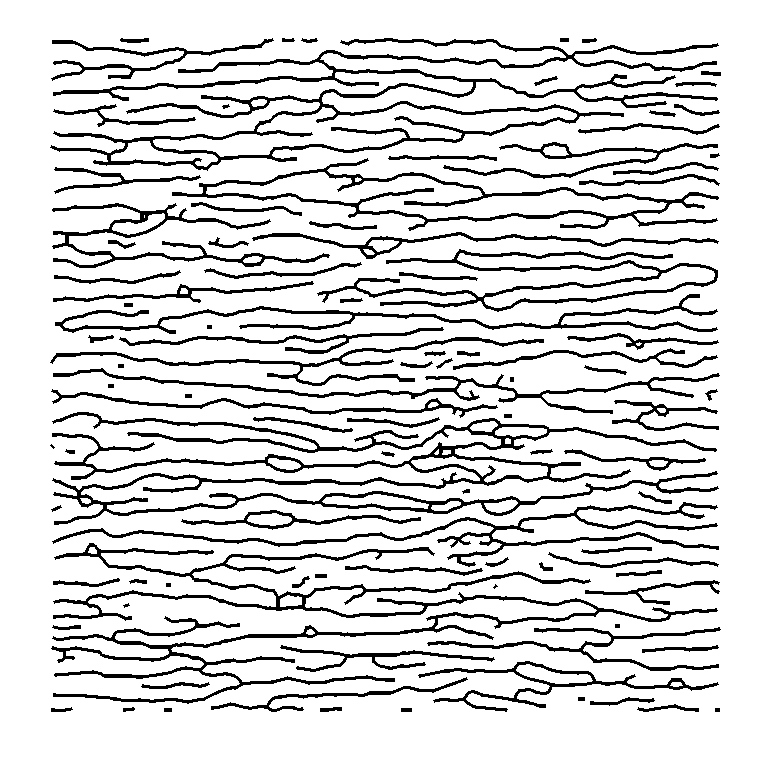

Supplement: Supplemental Information 3 [file peerj-07-7813-s003.zip › Supplemental-3/I-12-1.bmp]

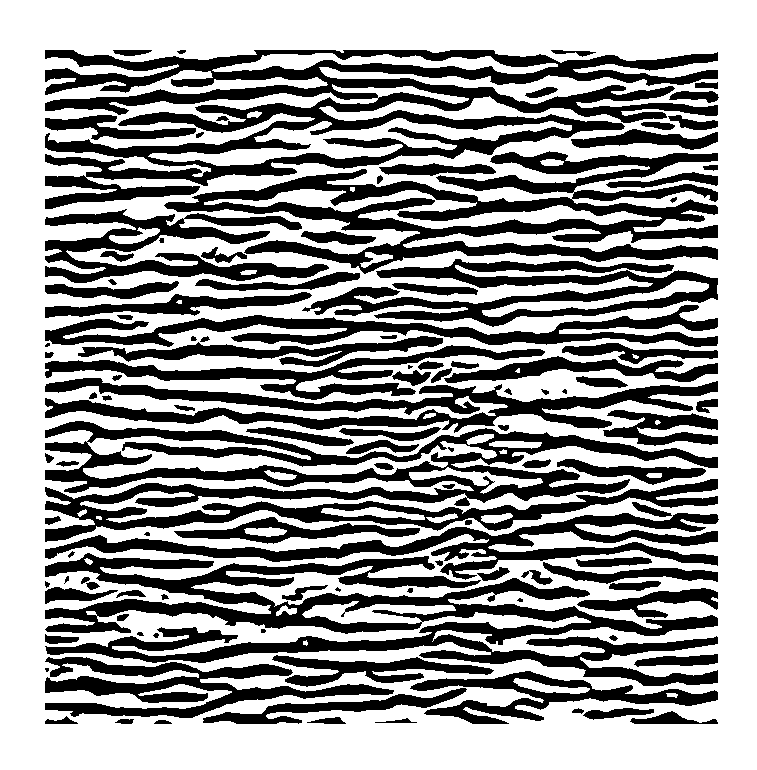

Supplement: Supplemental Information 3 [file peerj-07-7813-s003.zip › Supplemental-3/I-12.bmp]

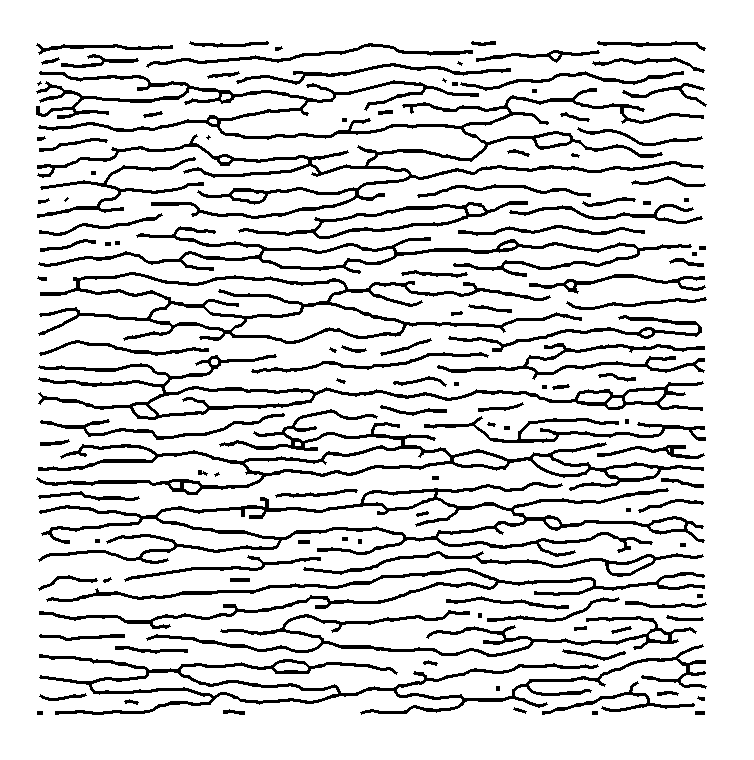

Supplement: Supplemental Information 3 [file peerj-07-7813-s003.zip › Supplemental-3/I-13-1.bmp]

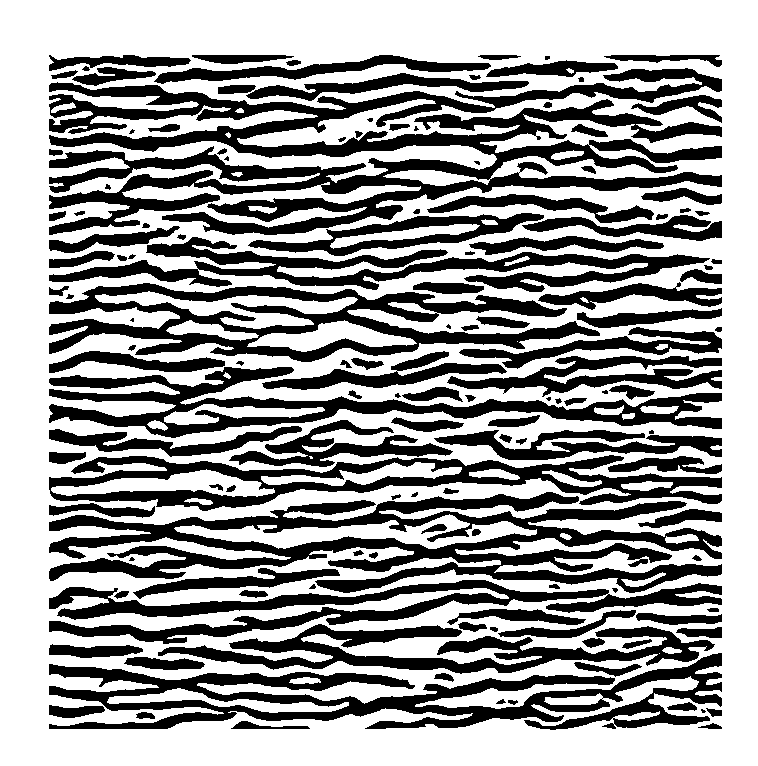

Supplement: Supplemental Information 3 [file peerj-07-7813-s003.zip › Supplemental-3/I-13.bmp]

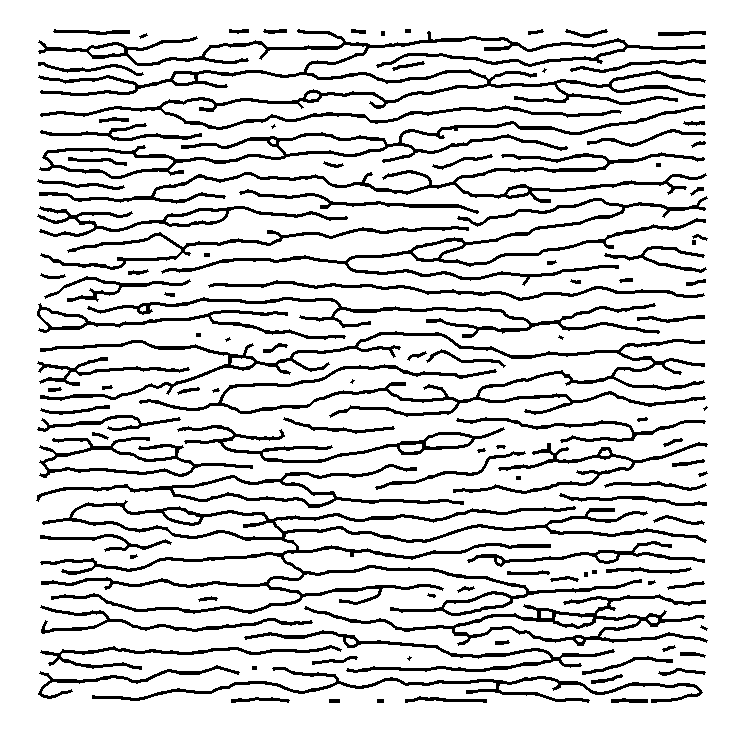

Supplement: Supplemental Information 3 [file peerj-07-7813-s003.zip › Supplemental-3/I-14-1.bmp]

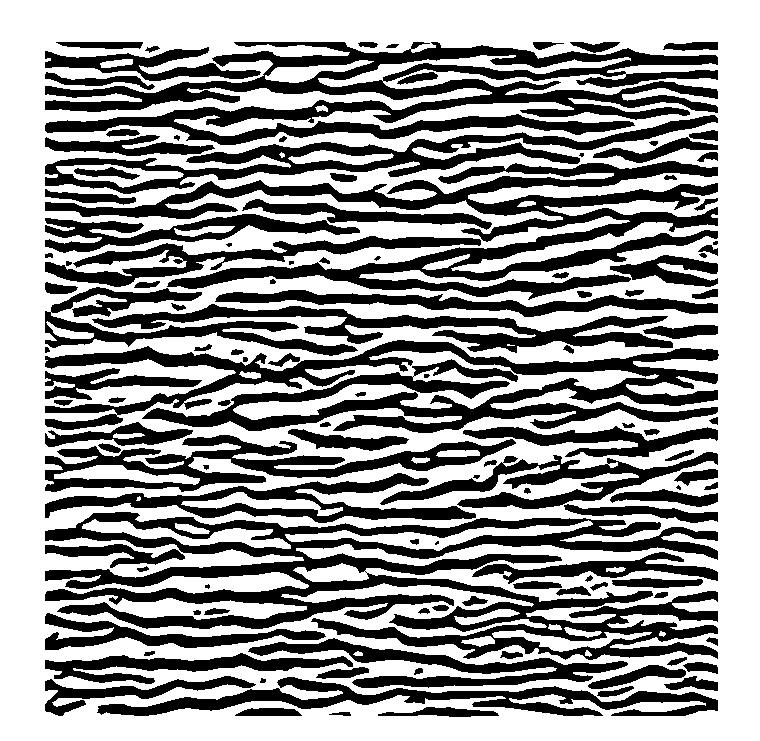

Supplement: Supplemental Information 3 [file peerj-07-7813-s003.zip › Supplemental-3/I-14.bmp]

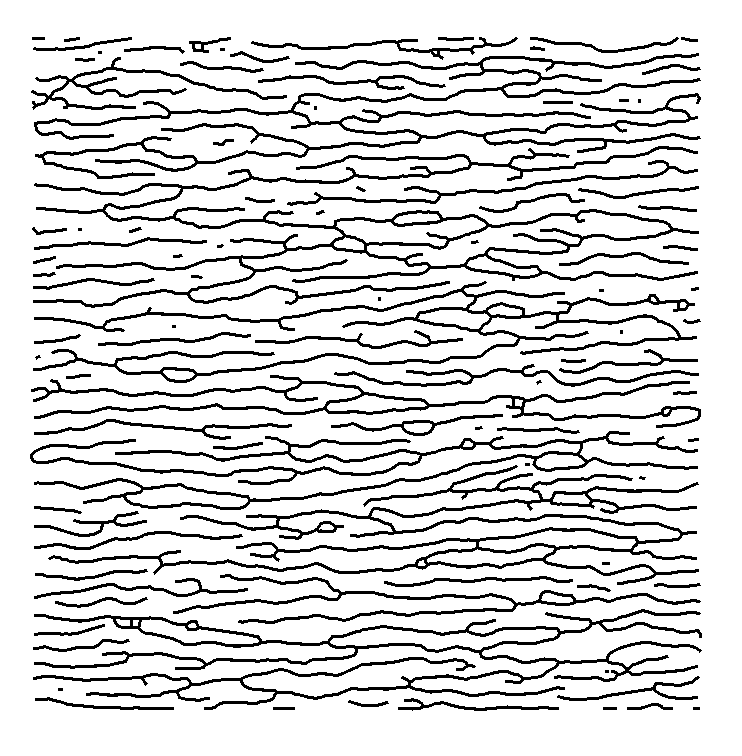

Supplement: Supplemental Information 3 [file peerj-07-7813-s003.zip › Supplemental-3/I-15-1.bmp]

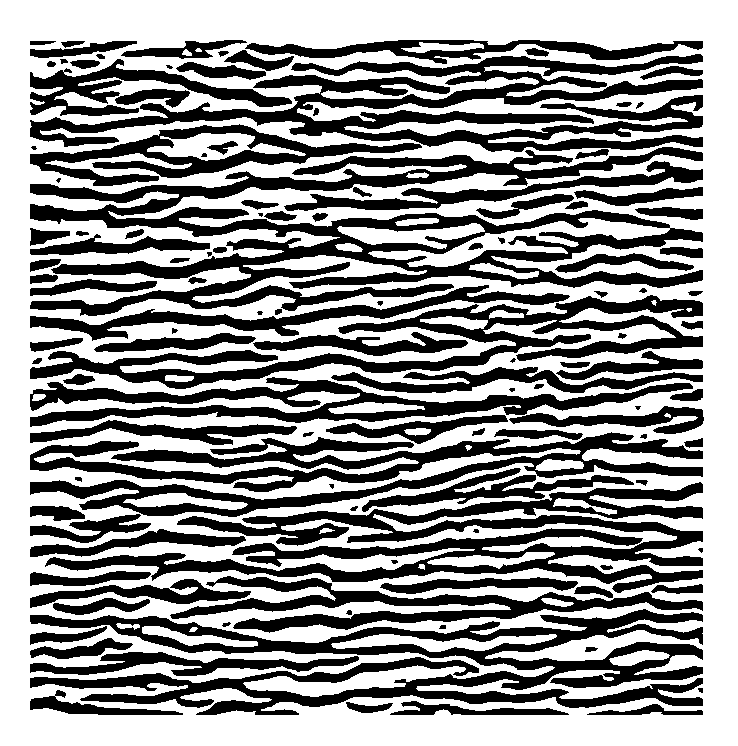

Supplement: Supplemental Information 3 [file peerj-07-7813-s003.zip › Supplemental-3/I-15.bmp]

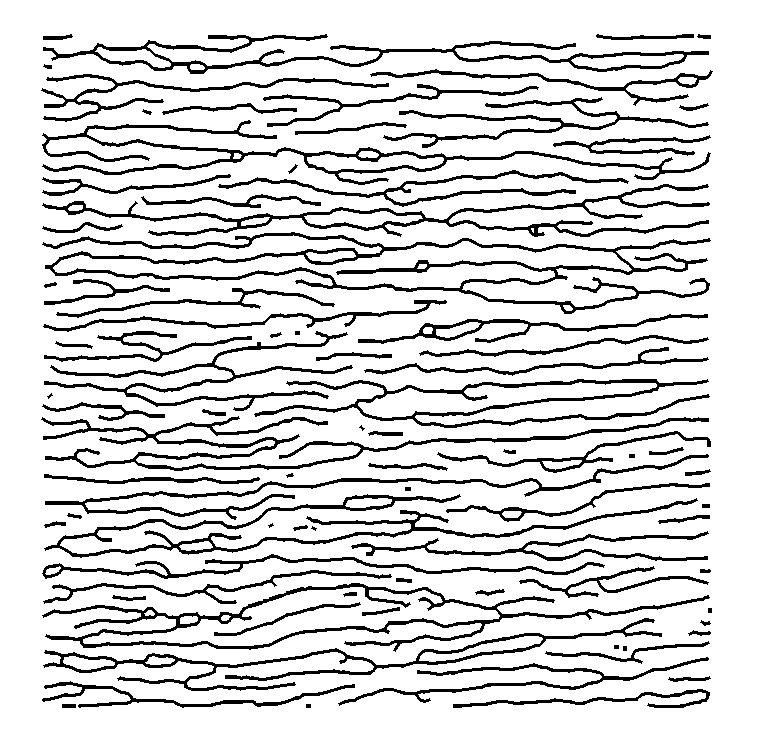

Supplement: Supplemental Information 3 [file peerj-07-7813-s003.zip › Supplemental-3/I-16-1.bmp]

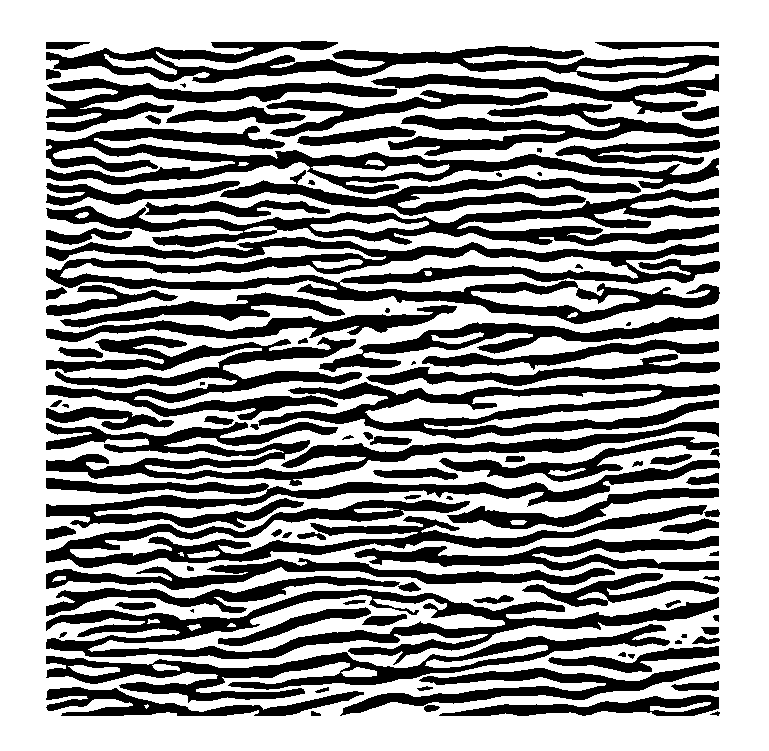

Supplement: Supplemental Information 3 [file peerj-07-7813-s003.zip › Supplemental-3/I-16.bmp]

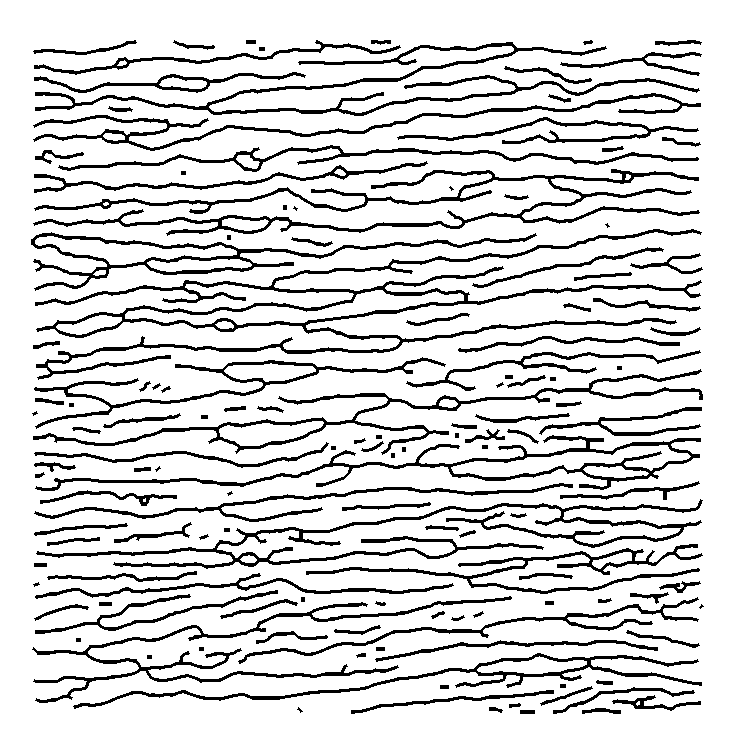

Supplement: Supplemental Information 3 [file peerj-07-7813-s003.zip › Supplemental-3/I-17-1.bmp]

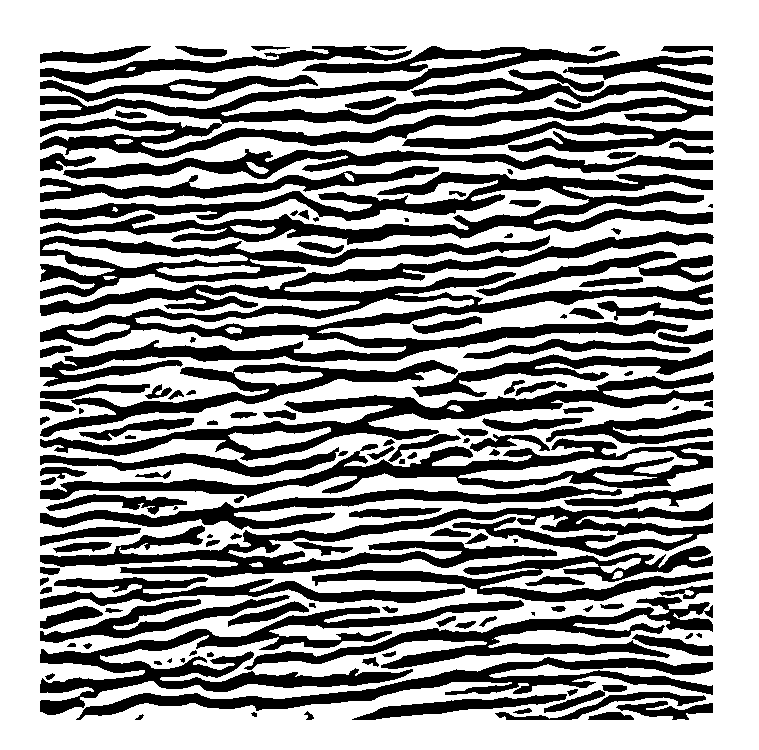

Supplement: Supplemental Information 3 [file peerj-07-7813-s003.zip › Supplemental-3/I-17.bmp]

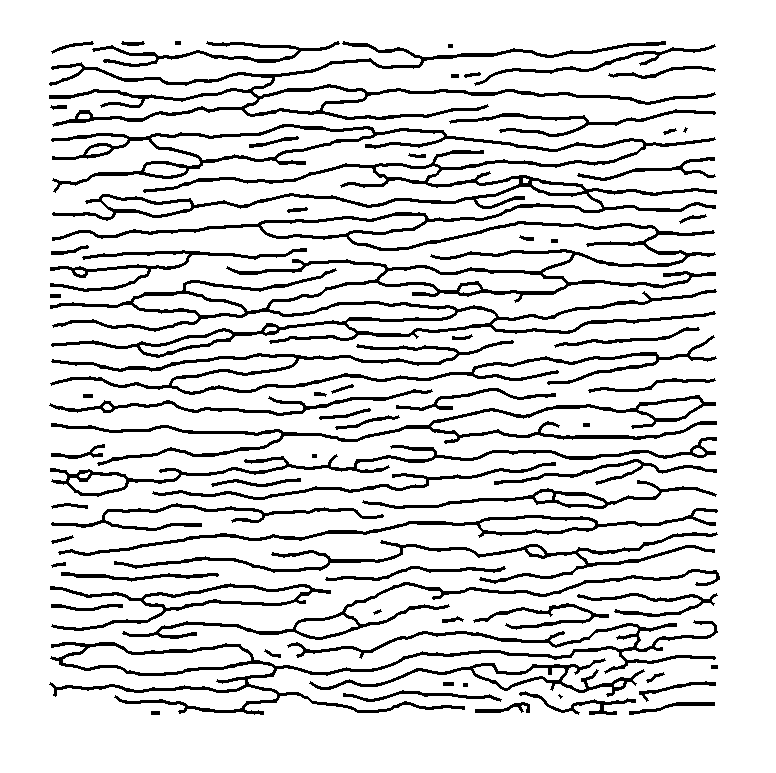

Supplement: Supplemental Information 3 [file peerj-07-7813-s003.zip › Supplemental-3/I-18-1.bmp]

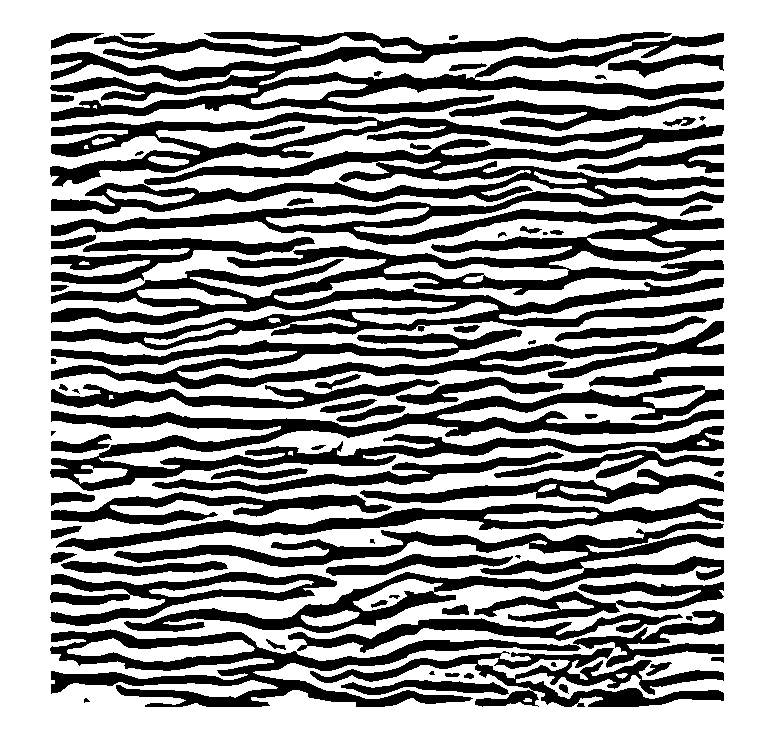

Supplement: Supplemental Information 3 [file peerj-07-7813-s003.zip › Supplemental-3/I-18.bmp]

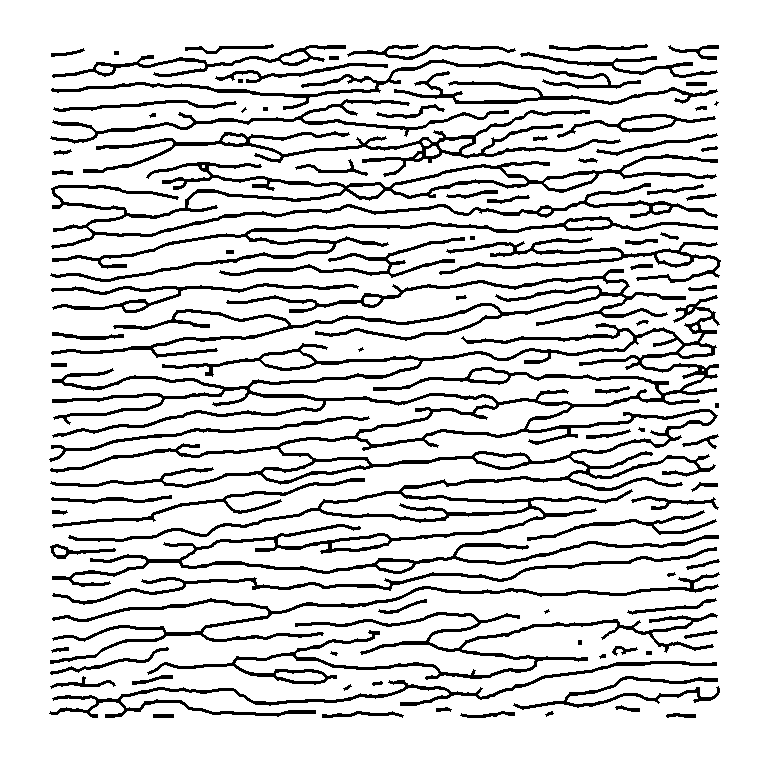

Supplement: Supplemental Information 3 [file peerj-07-7813-s003.zip › Supplemental-3/I-19-1.bmp]

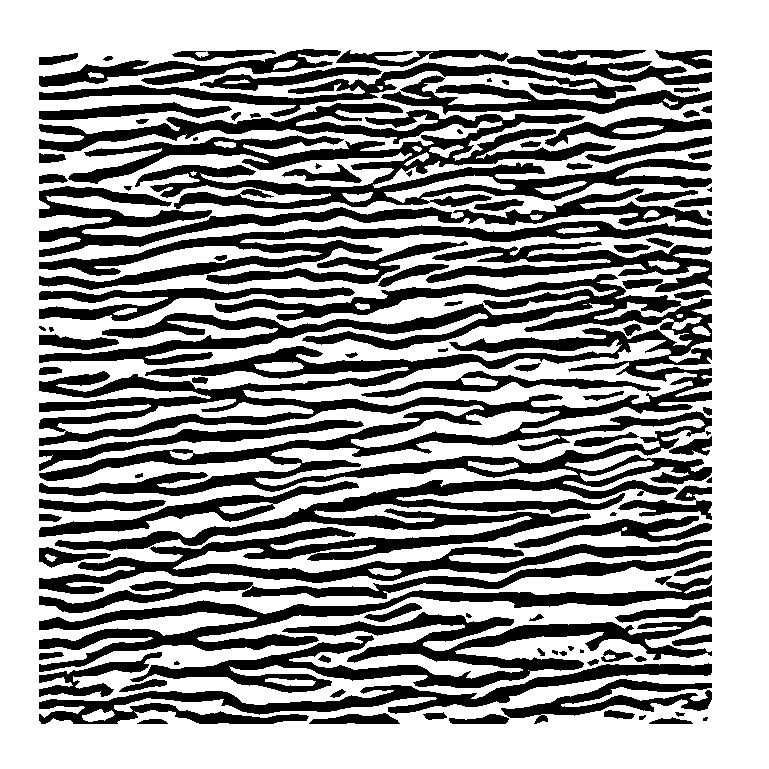

Supplement: Supplemental Information 3 [file peerj-07-7813-s003.zip › Supplemental-3/I-19.bmp]

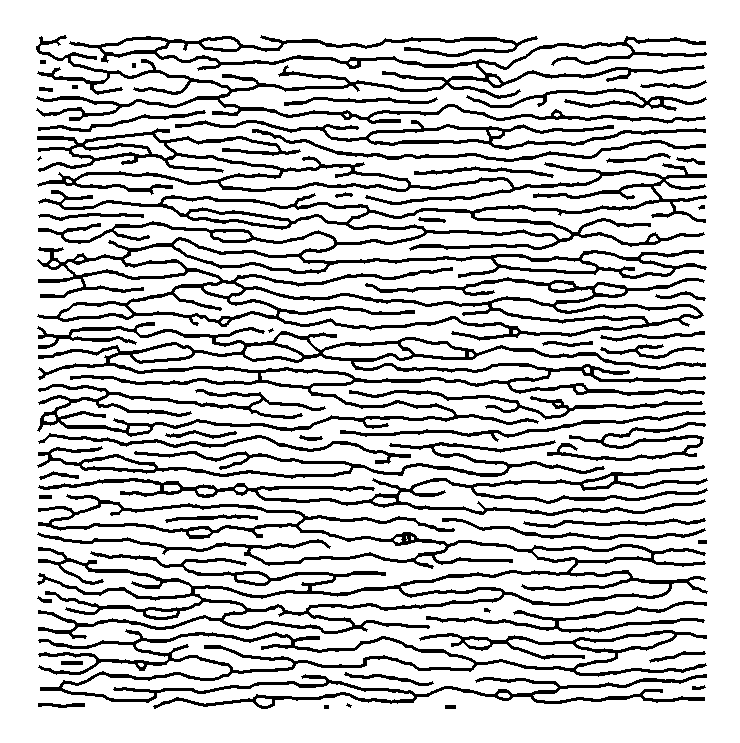

Supplement: Supplemental Information 3 [file peerj-07-7813-s003.zip › Supplemental-3/J-04-1.bmp]

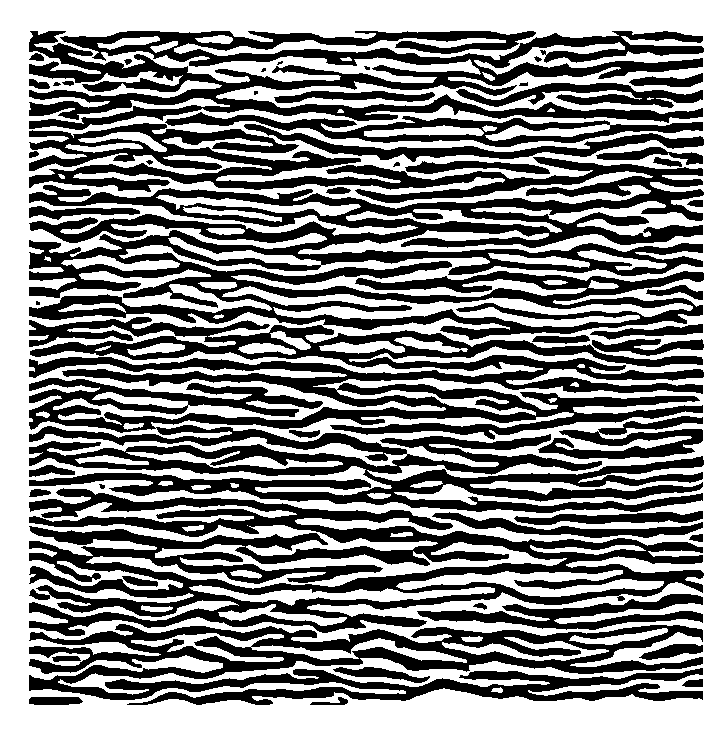

Supplement: Supplemental Information 3 [file peerj-07-7813-s003.zip › Supplemental-3/J-04.bmp]

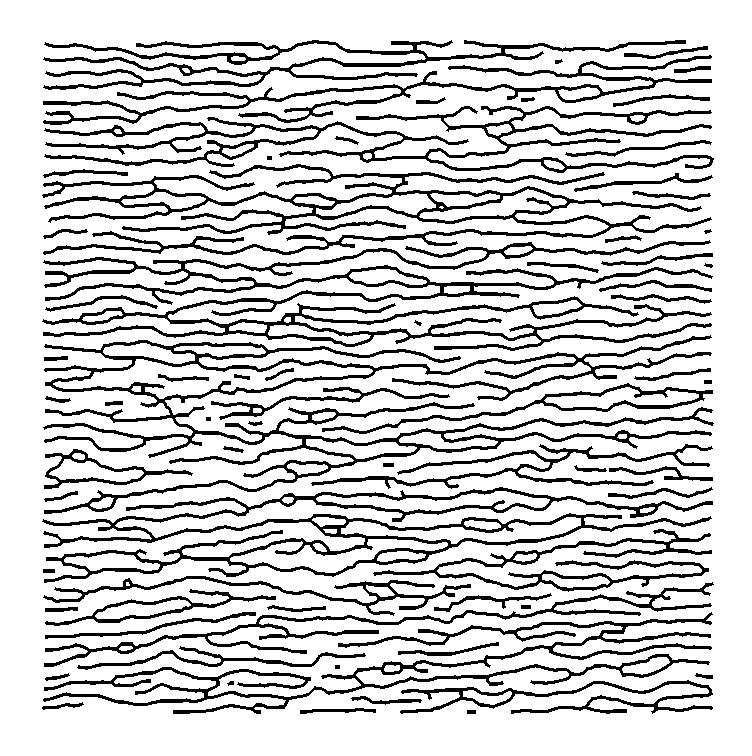

Supplement: Supplemental Information 3 [file peerj-07-7813-s003.zip › Supplemental-3/J-05-1.bmp]

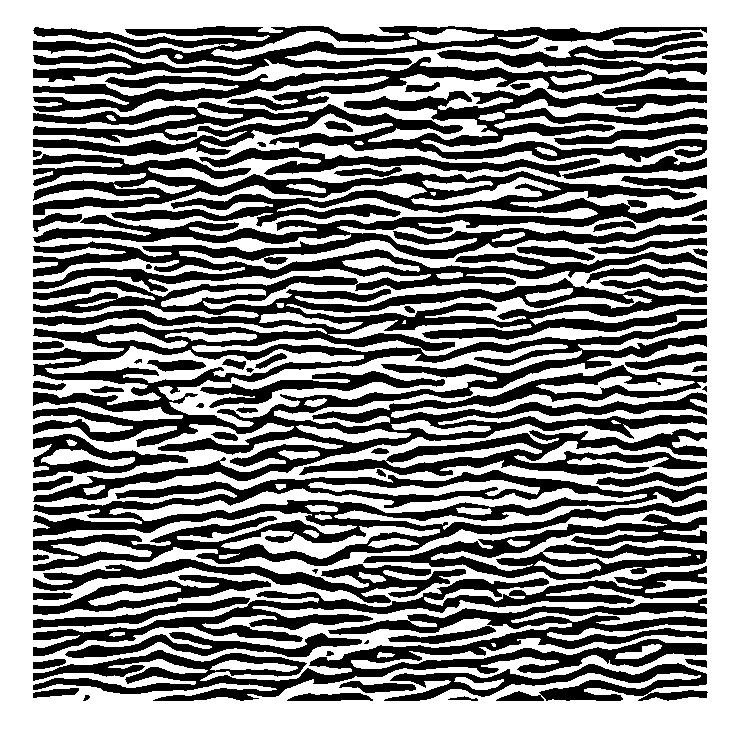

Supplement: Supplemental Information 3 [file peerj-07-7813-s003.zip › Supplemental-3/J-05.bmp]

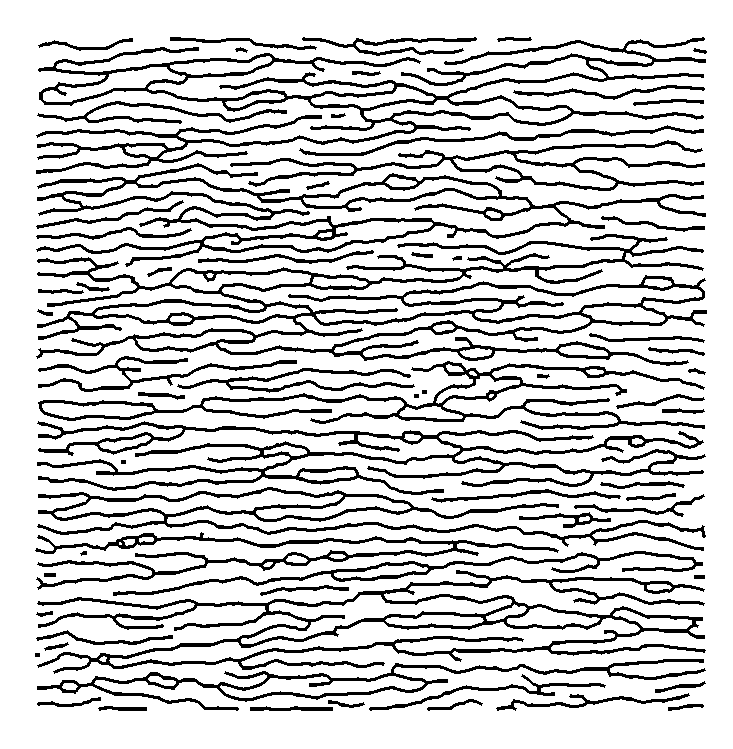

Supplement: Supplemental Information 3 [file peerj-07-7813-s003.zip › Supplemental-3/J-06-1.bmp]
