# Supplementary figures and images for: Layered patterns in nature, medicine, and materials: quantifying anisotropic structures and cyclicity (part 4 of 4)
Source: PeerJ. 2019 Oct 14;7:e7813. doi: 10.7717/peerj.7813 (PMC6797002; doi:10.7717/peerj.7813)

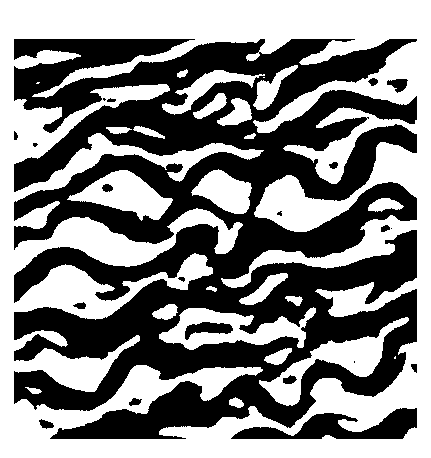

Supplement: Supplemental Information 4 [file peerj-07-7813-s004.zip › Supplemental-4/D-12.bmp]

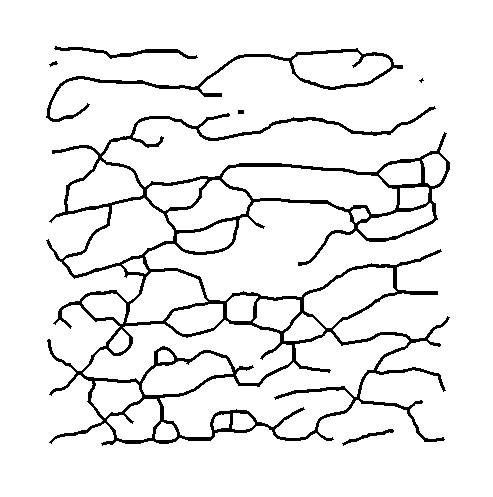

Supplement: Supplemental Information 4 [file peerj-07-7813-s004.zip › Supplemental-4/D-13-1.bmp]

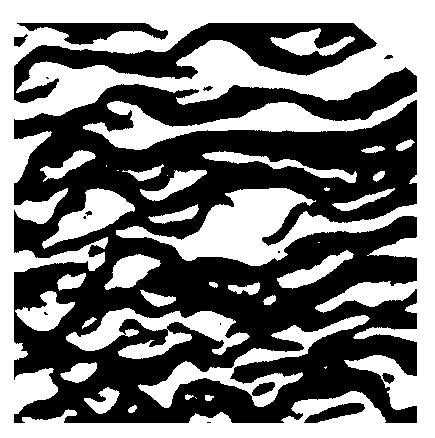

Supplement: Supplemental Information 4 [file peerj-07-7813-s004.zip › Supplemental-4/D-13.bmp]

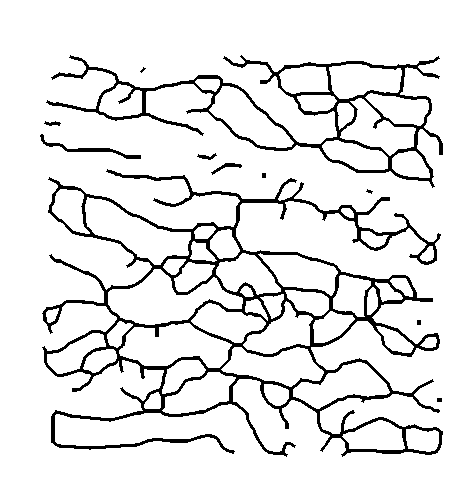

Supplement: Supplemental Information 4 [file peerj-07-7813-s004.zip › Supplemental-4/E-02-1.bmp]

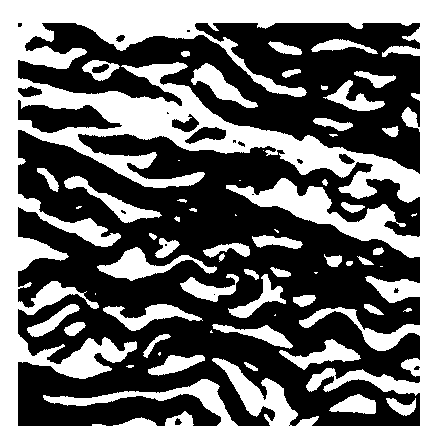

Supplement: Supplemental Information 4 [file peerj-07-7813-s004.zip › Supplemental-4/E-02.bmp]

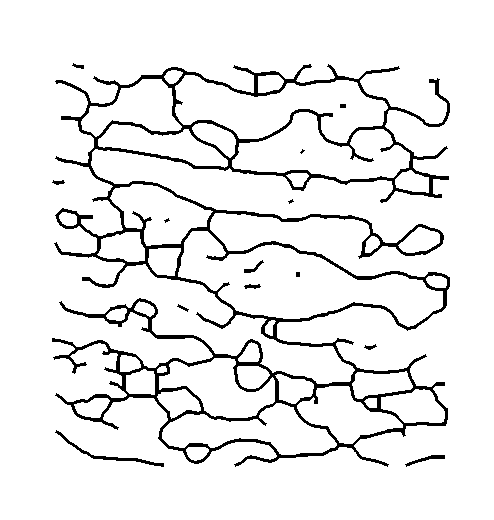

Supplement: Supplemental Information 4 [file peerj-07-7813-s004.zip › Supplemental-4/E-03-1.bmp]

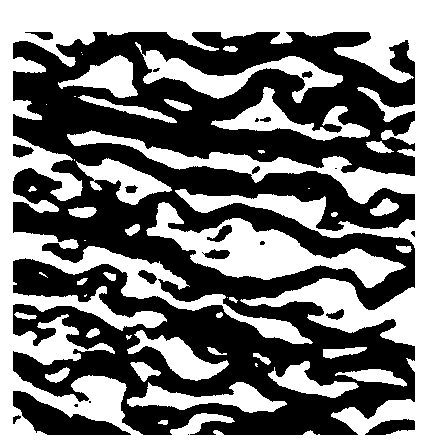

Supplement: Supplemental Information 4 [file peerj-07-7813-s004.zip › Supplemental-4/E-03.bmp]

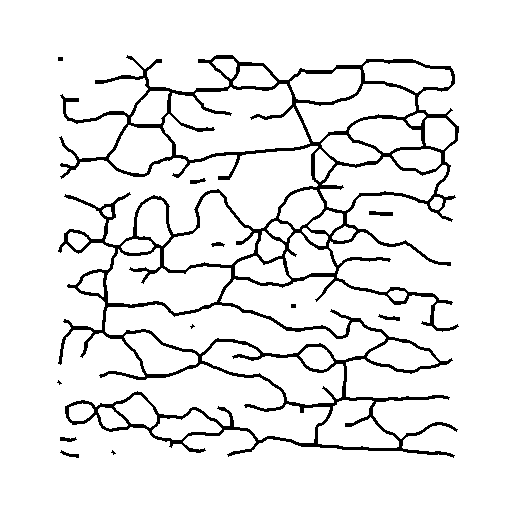

Supplement: Supplemental Information 4 [file peerj-07-7813-s004.zip › Supplemental-4/E-04-1.bmp]

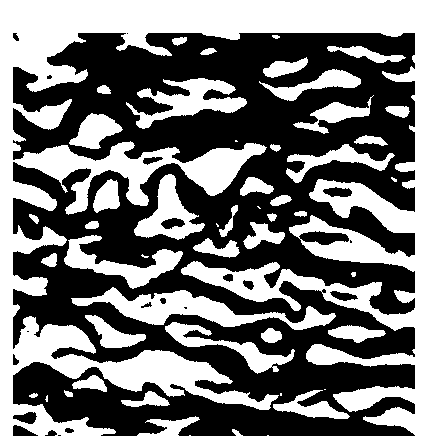

Supplement: Supplemental Information 4 [file peerj-07-7813-s004.zip › Supplemental-4/E-04.bmp]

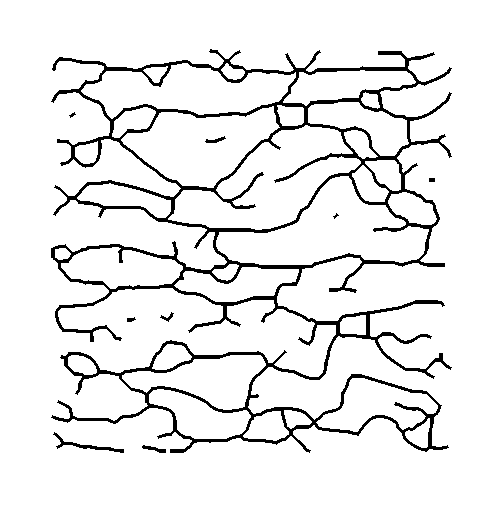

Supplement: Supplemental Information 4 [file peerj-07-7813-s004.zip › Supplemental-4/E-05-1.bmp]

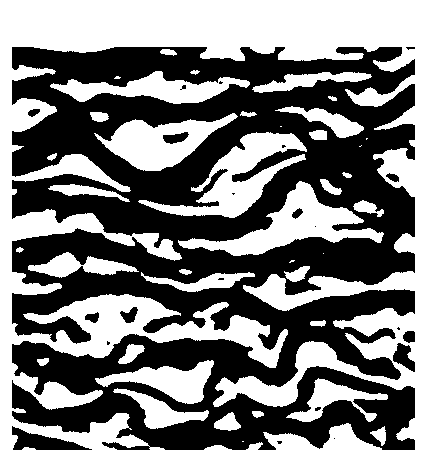

Supplement: Supplemental Information 4 [file peerj-07-7813-s004.zip › Supplemental-4/E-05.bmp]

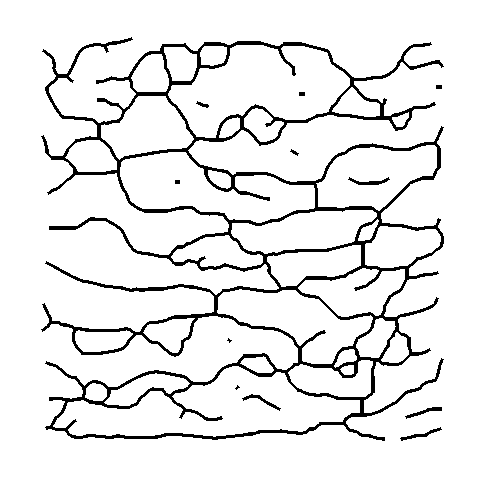

Supplement: Supplemental Information 4 [file peerj-07-7813-s004.zip › Supplemental-4/E-06-1.bmp]

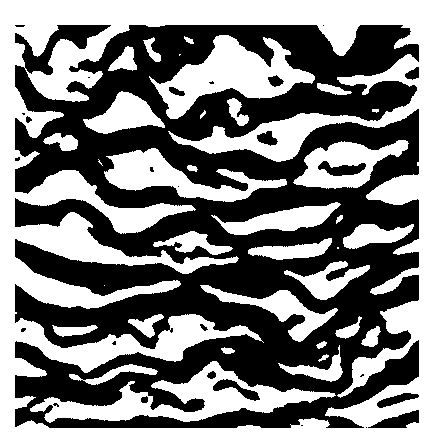

Supplement: Supplemental Information 4 [file peerj-07-7813-s004.zip › Supplemental-4/E-06.bmp]

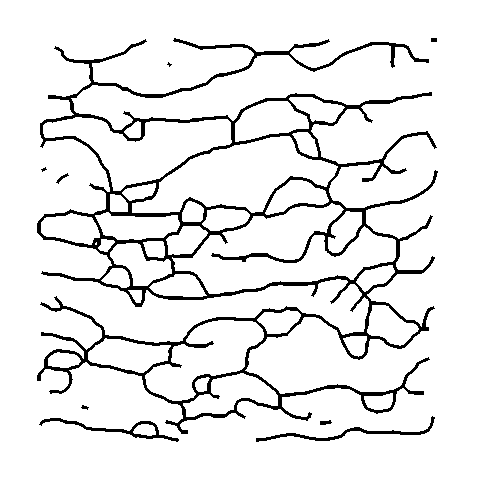

Supplement: Supplemental Information 4 [file peerj-07-7813-s004.zip › Supplemental-4/E-07-1.bmp]

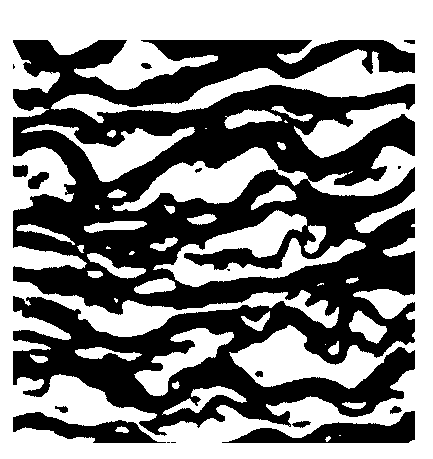

Supplement: Supplemental Information 4 [file peerj-07-7813-s004.zip › Supplemental-4/E-07.bmp]

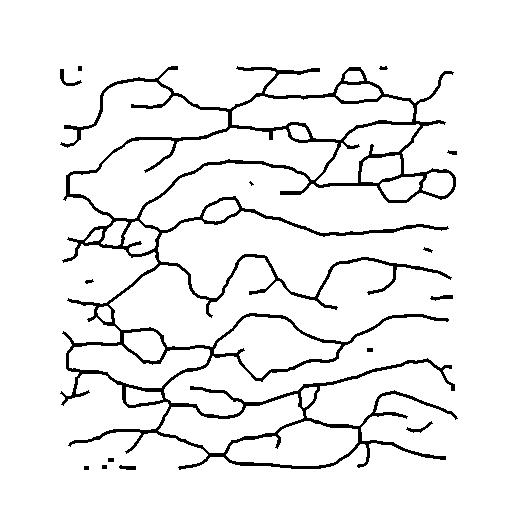

Supplement: Supplemental Information 4 [file peerj-07-7813-s004.zip › Supplemental-4/E-08-1.bmp]

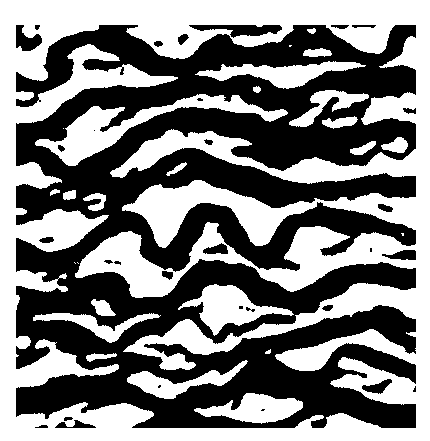

Supplement: Supplemental Information 4 [file peerj-07-7813-s004.zip › Supplemental-4/E-08.bmp]

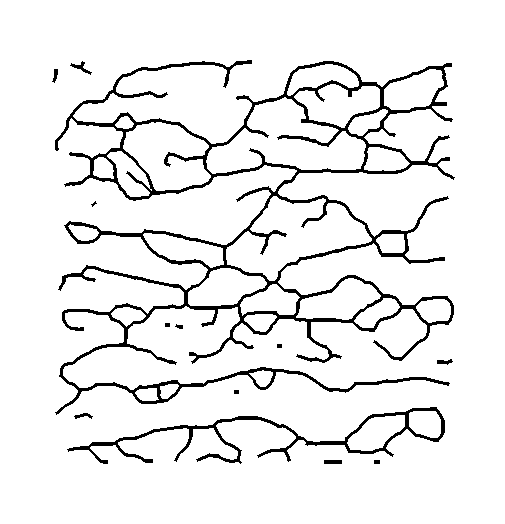

Supplement: Supplemental Information 4 [file peerj-07-7813-s004.zip › Supplemental-4/E-09-1.bmp]

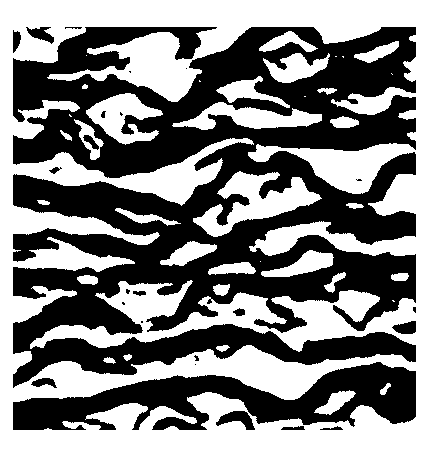

Supplement: Supplemental Information 4 [file peerj-07-7813-s004.zip › Supplemental-4/E-09.bmp]

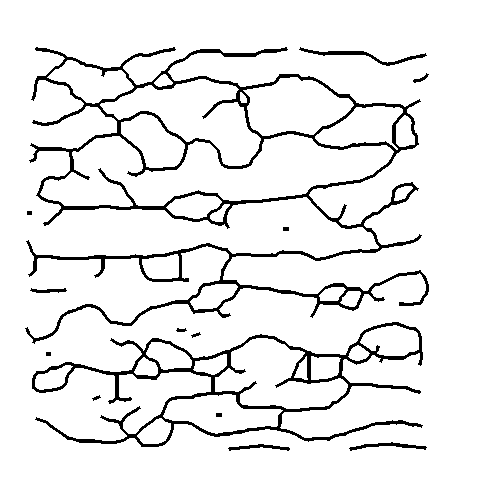

Supplement: Supplemental Information 4 [file peerj-07-7813-s004.zip › Supplemental-4/E-10-1.bmp]

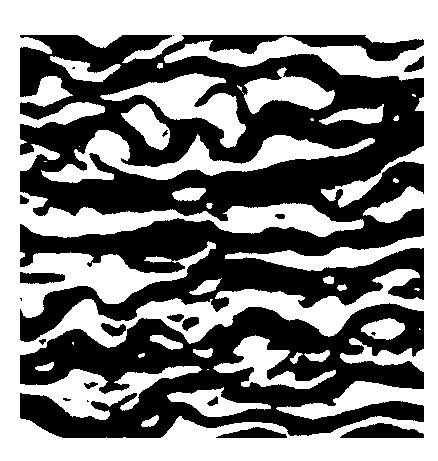

Supplement: Supplemental Information 4 [file peerj-07-7813-s004.zip › Supplemental-4/E-10.bmp]

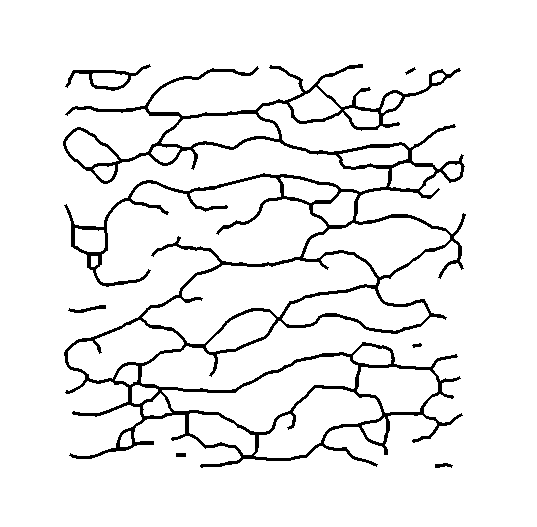

Supplement: Supplemental Information 4 [file peerj-07-7813-s004.zip › Supplemental-4/E-11-1.bmp]

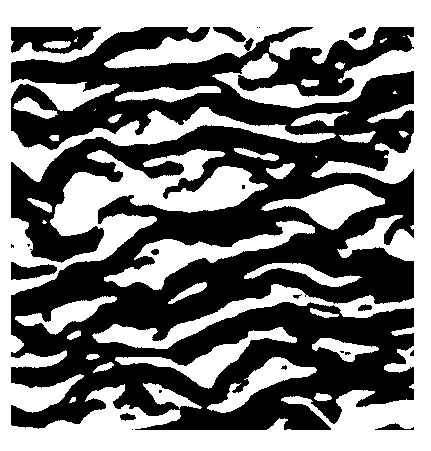

Supplement: Supplemental Information 4 [file peerj-07-7813-s004.zip › Supplemental-4/E-11.bmp]

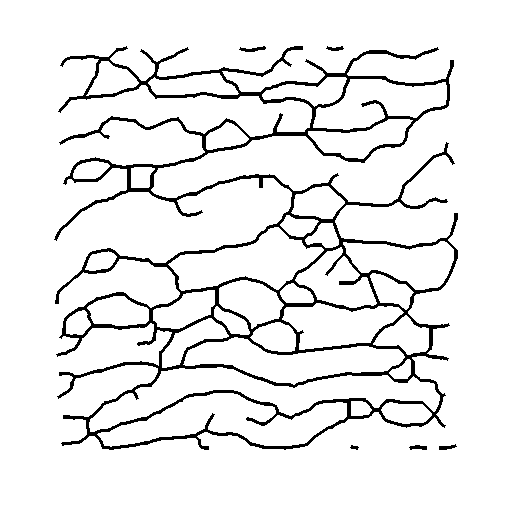

Supplement: Supplemental Information 4 [file peerj-07-7813-s004.zip › Supplemental-4/E-12-1.bmp]

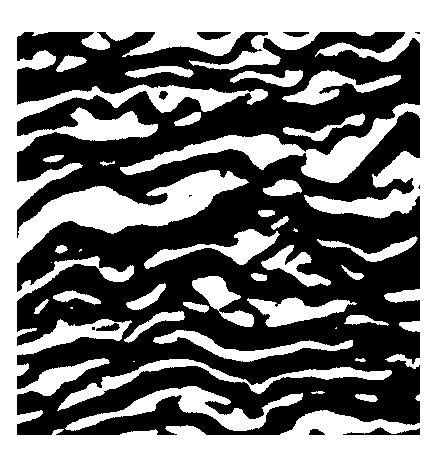

Supplement: Supplemental Information 4 [file peerj-07-7813-s004.zip › Supplemental-4/E-12.bmp]

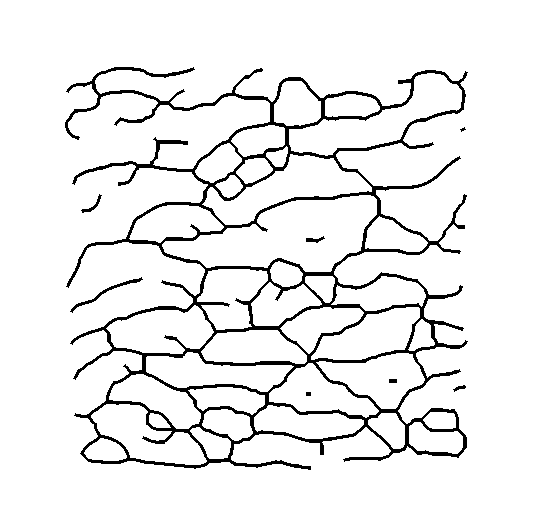

Supplement: Supplemental Information 4 [file peerj-07-7813-s004.zip › Supplemental-4/E-13-1.bmp]

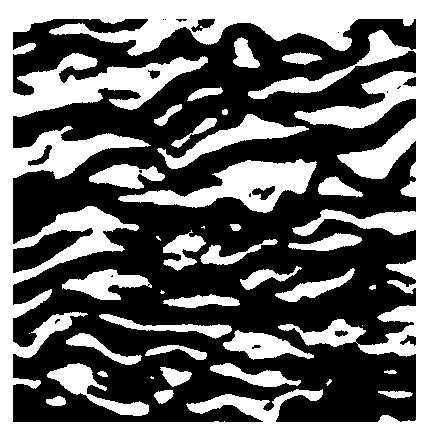

Supplement: Supplemental Information 4 [file peerj-07-7813-s004.zip › Supplemental-4/E-13.bmp]

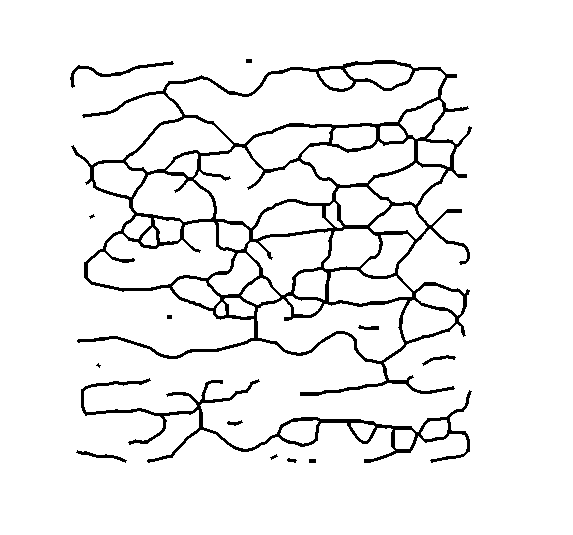

Supplement: Supplemental Information 4 [file peerj-07-7813-s004.zip › Supplemental-4/E-14-1.bmp]

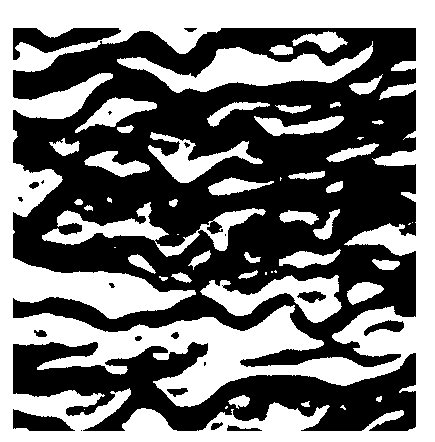

Supplement: Supplemental Information 4 [file peerj-07-7813-s004.zip › Supplemental-4/E-14.bmp]

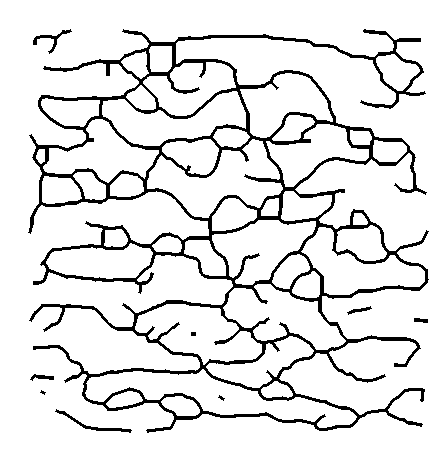

Supplement: Supplemental Information 4 [file peerj-07-7813-s004.zip › Supplemental-4/F-03-1.bmp]

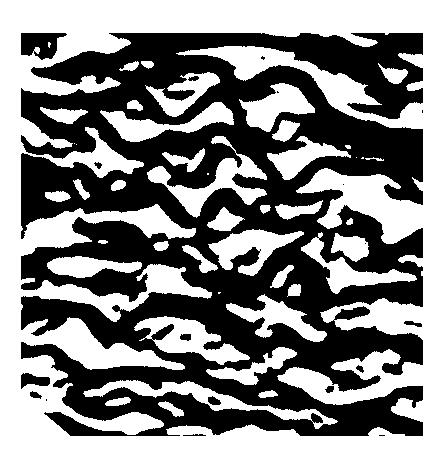

Supplement: Supplemental Information 4 [file peerj-07-7813-s004.zip › Supplemental-4/F-03.bmp]

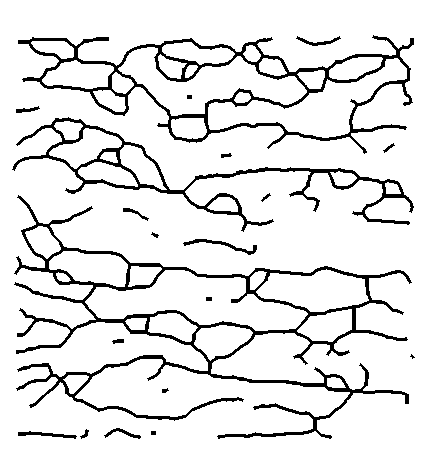

Supplement: Supplemental Information 4 [file peerj-07-7813-s004.zip › Supplemental-4/F-04-1.bmp]

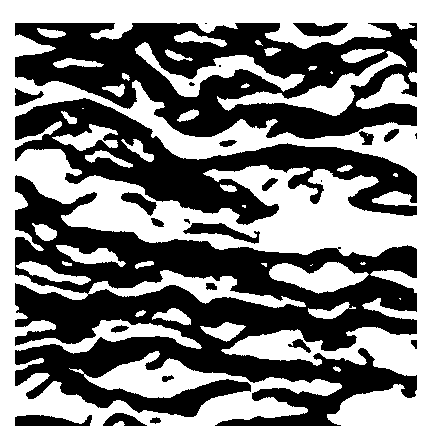

Supplement: Supplemental Information 4 [file peerj-07-7813-s004.zip › Supplemental-4/F-04.bmp]

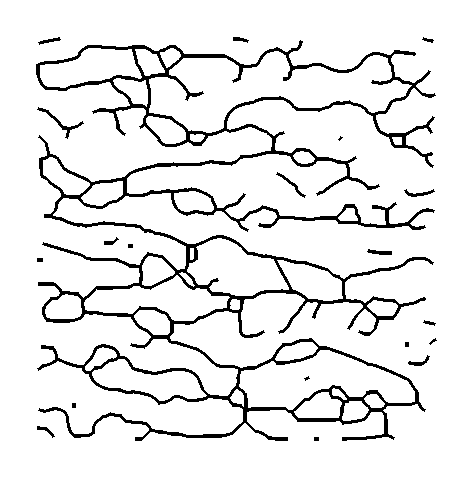

Supplement: Supplemental Information 4 [file peerj-07-7813-s004.zip › Supplemental-4/F-05-1.bmp]

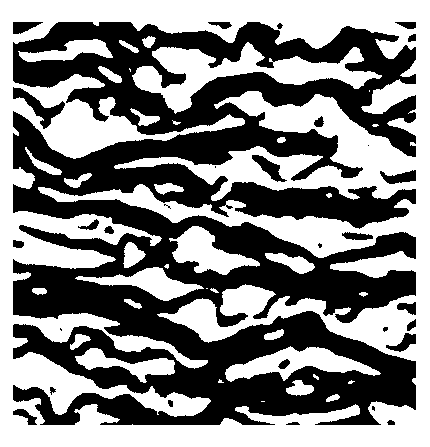

Supplement: Supplemental Information 4 [file peerj-07-7813-s004.zip › Supplemental-4/F-05.bmp]

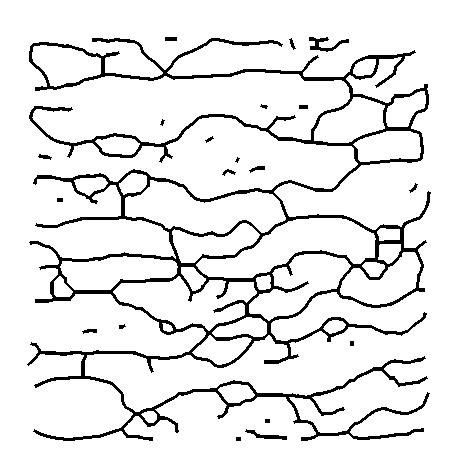

Supplement: Supplemental Information 4 [file peerj-07-7813-s004.zip › Supplemental-4/F-06-1.bmp]

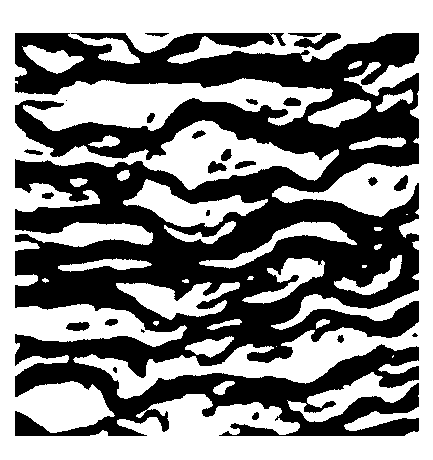

Supplement: Supplemental Information 4 [file peerj-07-7813-s004.zip › Supplemental-4/F-06.bmp]

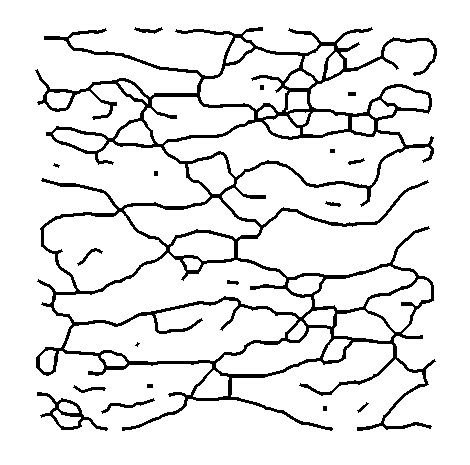

Supplement: Supplemental Information 4 [file peerj-07-7813-s004.zip › Supplemental-4/F-07-1.bmp]

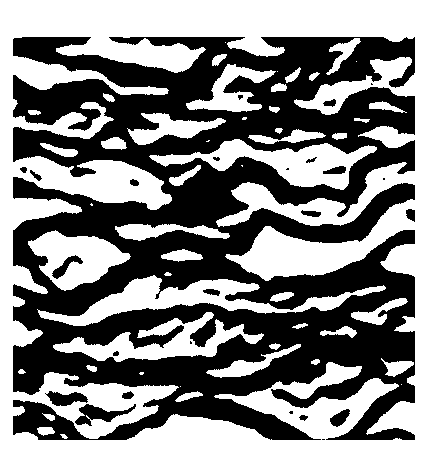

Supplement: Supplemental Information 4 [file peerj-07-7813-s004.zip › Supplemental-4/F-07.bmp]

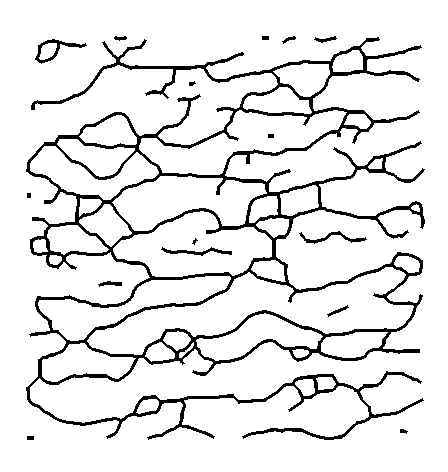

Supplement: Supplemental Information 4 [file peerj-07-7813-s004.zip › Supplemental-4/F-08-1.bmp]

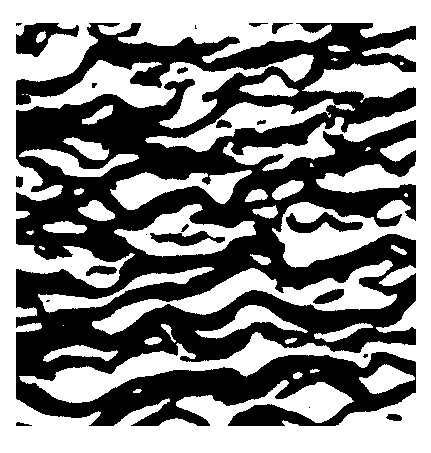

Supplement: Supplemental Information 4 [file peerj-07-7813-s004.zip › Supplemental-4/F-08.bmp]

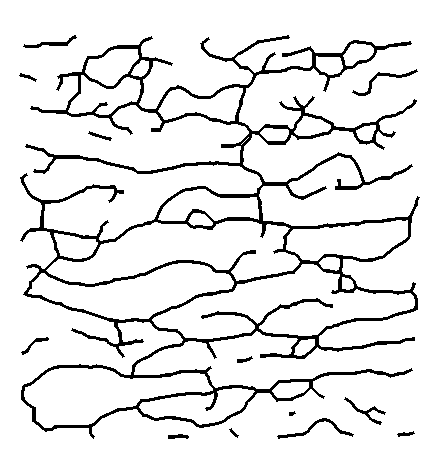

Supplement: Supplemental Information 4 [file peerj-07-7813-s004.zip › Supplemental-4/F-09-1.bmp]

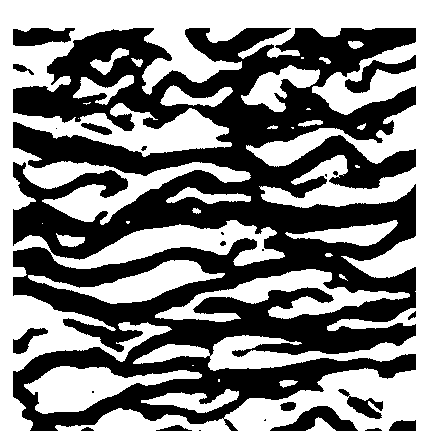

Supplement: Supplemental Information 4 [file peerj-07-7813-s004.zip › Supplemental-4/F-09.bmp]

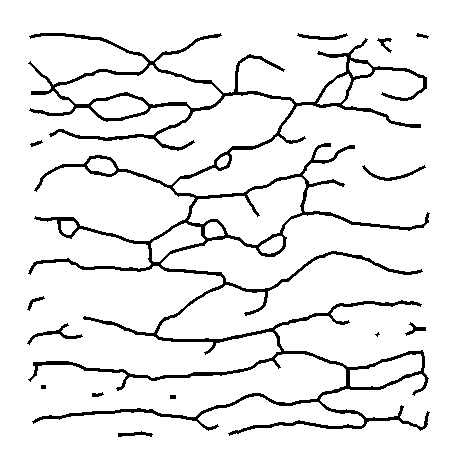

Supplement: Supplemental Information 4 [file peerj-07-7813-s004.zip › Supplemental-4/F-10-1.bmp]

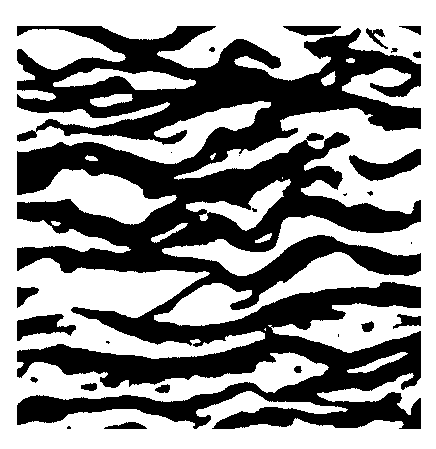

Supplement: Supplemental Information 4 [file peerj-07-7813-s004.zip › Supplemental-4/F-10.bmp]

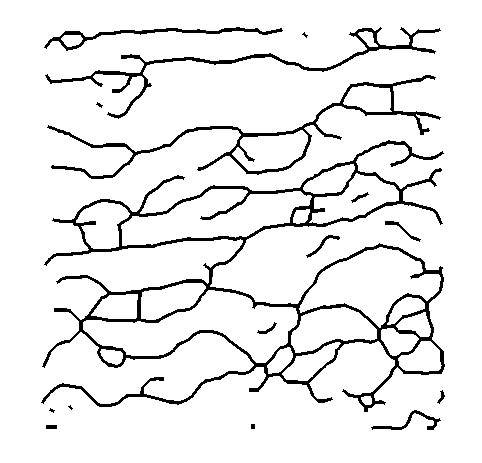

Supplement: Supplemental Information 4 [file peerj-07-7813-s004.zip › Supplemental-4/F-11-1.bmp]

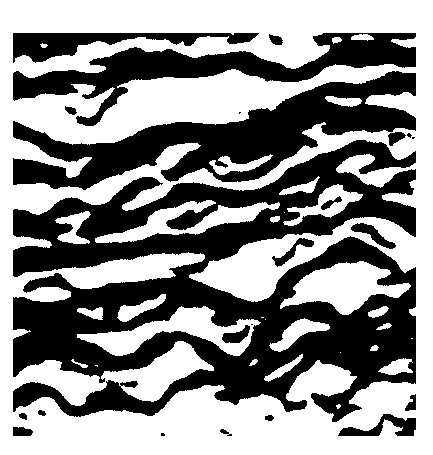

Supplement: Supplemental Information 4 [file peerj-07-7813-s004.zip › Supplemental-4/F-11.bmp]

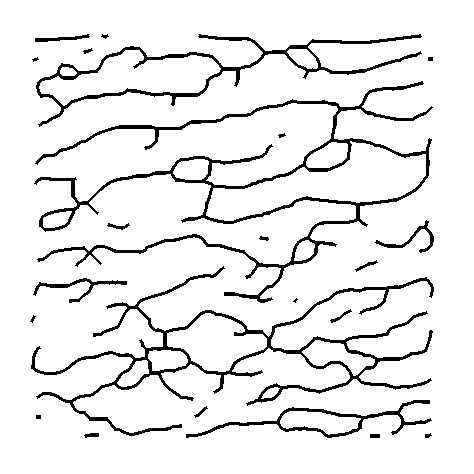

Supplement: Supplemental Information 4 [file peerj-07-7813-s004.zip › Supplemental-4/F-12-1.bmp]

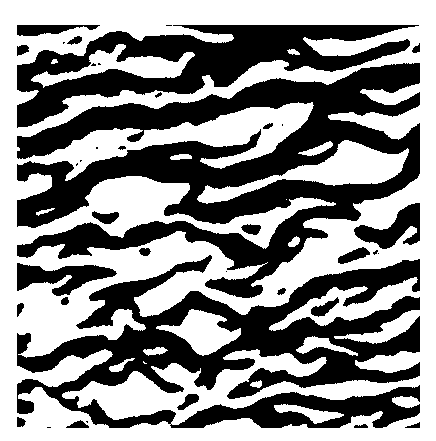

Supplement: Supplemental Information 4 [file peerj-07-7813-s004.zip › Supplemental-4/F-12.bmp]

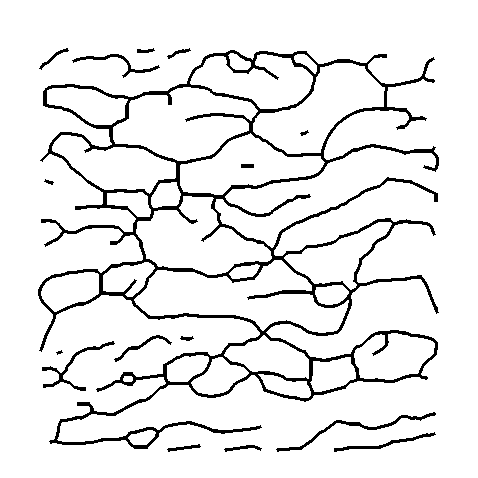

Supplement: Supplemental Information 4 [file peerj-07-7813-s004.zip › Supplemental-4/F-13-1.bmp]

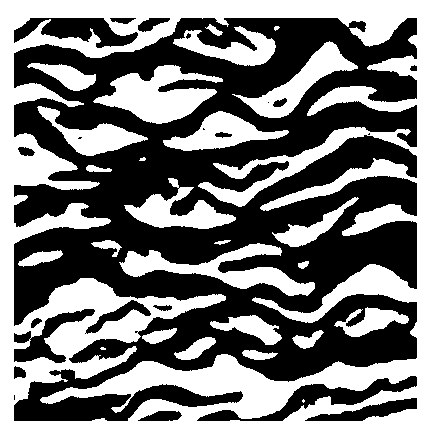

Supplement: Supplemental Information 4 [file peerj-07-7813-s004.zip › Supplemental-4/F-13.bmp]
